# Sinuhirtone A, An Uncommon 17,19-Dinorxeniaphyllanoid, and Nine Related New Terpenoids from the Hainan Soft Coral *Sinularia hirta*

**DOI:** 10.3390/md20040272

**Published:** 2022-04-18

**Authors:** Zi-Hui Chen, Si-Qi Lu, Guan-Ying Han, Xu-Wen Li, Yue-Wei Guo

**Affiliations:** 1 State Key Laboratory of Drug Research, Shanghai Institute of Materia Medica, Chinese Academy of Sciences, 555 Zu Chong Zhi Road, Zhangjiang Hi-Tech Park, Shanghai 201203, China; s18-chenzihui@simm.ac.cn (Z.-H.C.); siqi.lu@hengrui.com (S.-Q.L.); 2 University of Chinese Academy of Sciences, No. 19A Yuquan Road, Beijing 100049, China; 3 Jinzhou Medical University, Jinzhou 121001, China; 4 Drug Discovery Shandong Laboratory, Bohai Rim Advanced Research Institute for Drug Discovery, Yantai 264117, China

**Keywords:** soft coral, *Sinularia hirta*, xeniaphyllanes, norditerpenoids, stereochemistry

## Abstract

Chemical investigation of the Hainan soft coral *Sinularia hirta* resulted in the isolation and identification of a library of sixteen structurally diverse terpenoids, including a dinorditerpenoid with an uncommon 17,19-dinorxeniaphyllane skeleton, namely sinuhirtone A (**7**), six new xeniaphyllane-type diterpenoids (**1**–**6**), one new norxeniaphyllanoid (**8**), two new norcaryophyllene-type sesquiterpenoids (**9** and **10**), together with six known related compounds (**11**–**16**). Compounds **1**–**3** are three new furanone-containing xeniaphyllane-type diterpenoids. The structures of the new compounds, including their absolute configurations, were determined by extensive spectroscopic analysis and a series of quantum chemical calculations, including quantum mechanical-nuclear magnetic resonance (QM–NMR), time-dependent density functional theory–electronic circular dichroism (TDDFT–ECD), and optical rotatory dispersion (ORD) methods. A plausible biosynthetic connection between new compounds **1**–**9** was also proposed. New compounds **2**–**4**, **7**, and **8** were evaluated for in vitro cytotoxicity against four cancer cell lines.

## 1. Introduction

Soft corals of the genus *Sinularia* (phylum Cnidaria, class Anthozoa, subclass Octocorallia, order Alcyonacea, family Alcyoniidae) are rich sources of various sesquiterpenoids and diterpenoids with diverse structural features [1,2,3]. Many of these metabolites merit further investigation due to their potent biological activities, such as anti-inflammatory [4], cytotoxic [5], immunosuppressive [6], anti-bacterial [7], and antifouling [8] effects. Among all the species of *Sinularia*, *S. hirta* (Andaman and Nicobar Islands, India) has only been chemically investigated by Anjaneyulu and co-workers, with two steroids, two cembranoids, and two sesquiterpenoids having been reported [9,10]. Hence, *S. hirta* deserves a more-in-depth investigation, both chemically and biologically.

**Figure 1:**
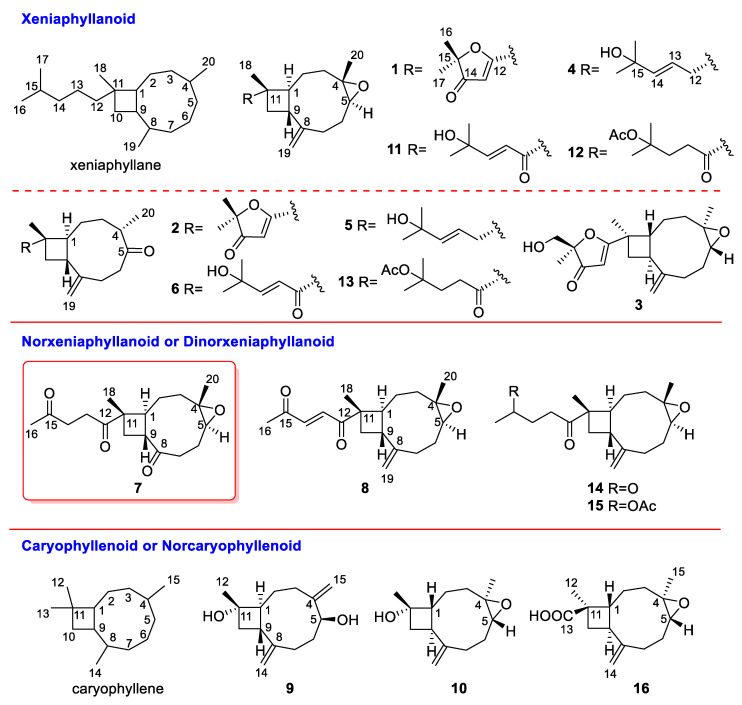
Structures of compounds **1**–**16**, xeniaphyllane, and caryophyllene.

During our continuing efforts to explore bioactive secondary metabolites from South China Sea invertebrates [11,12,13,14,15], *S. hirta* was collected off the coast of Yalong Bay, Hainan, China, and chemically investigated, resulting in the initial isolation of two new norhumulene-type norsesquiterpenoids, sinuhirtins A and B, together with two known norcaryophyllene-type sesquiterpenoids [16]. In order to accumulate more such structurally interesting compounds for biological study, a further systematic chemical investigation of this specimen was carried out this time, resulting in the isolation of a novel dinorditerpenoid, namely sinuhirtone A (**7**); six new xeniaphyllanoids, namely sinuhirfuranones A–C (**1**–**3**) and sinuhirtins C–E (**4**–**6**); a norxeniaphyllanoid, namely sinuhirtone B (**8**); two new norcaryophyllenoids, namely sinuhirtins F (**9**) and G (**10**); together with six known related compounds (**11**–**16**). It is worth mentioning that sinuhirtone A features an uncommon 17,19-dinorxeniaphyllane-type skeleton, and sinuhirfuranones A–C represent the first example of marine diterpenoids possessing the 2,2-dimethylfuran-3-one group (Figure 1). Herein, we report the isolation, structural elucidation, and plausible biosynthetic pathway of the new isolates and their bioactivity evaluation.

## 2. Results and Discussion

The frozen body of soft coral *S. hirta* was cut into pieces and exhaustively extracted with acetone. The Et_2_O-soluble portion of the acetone extract was purified repeatedly by a combination of silica gel, Sephadex LH-20, and reversed-phase (RP)-HPLC to yield sixteen pure terpenoids: **1** (3.2 mg), **2** (1.1 mg), **3** (0.9 mg), **4** (1.3 mg), **5** (1.6 mg), **6** (1.5 mg), **7** (1.1 mg), **8** (1.5 mg) **9** (4.1 mg), **10** (1.6 mg), **11** (1.7 mg), **12** (1.3 mg), **13** (2.0 mg), **14** (2.2 mg), **15** (1.4 mg), and **16** (1.5 mg). By comparing their NMR spectroscopic data and optical rotation with those reported in the literature, the known compounds were readily identified as nanolobatin B (**11**) [17], gibberosin D (**12**) [18], gibberosin Q (**13**) [19], gibberosin A (**14**) [18], nanolobatin C (**15**) [17], and (1*R*,4*R*,5*R*,9*S*,11*R*)-4,5-epoxy-caryophyll-8(14)-en-12-oic acid (**16**) [20], respectively.

**Figure 2:**
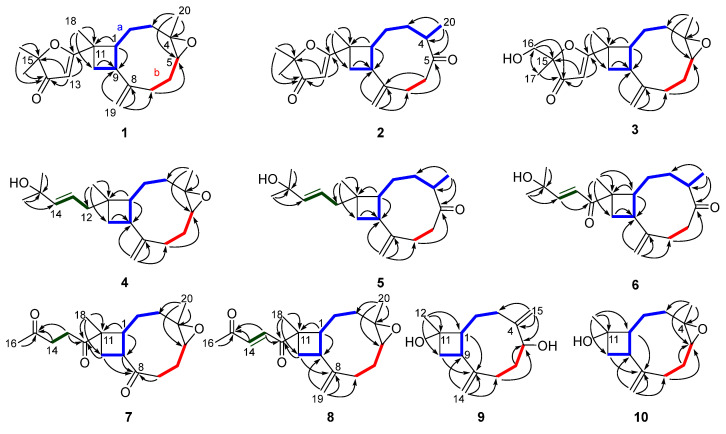
Selected ^1^H–^1^H COSY (bold lines) and key HMBC (single arrows) correlations of **1**–**10**. Fragments **a** (blue) and **b** (red) represent two different spin systems, respectively.

Sinuhirfuranone A (**1**) was obtained as a colorless oil. Its molecular formula, C_20_H_28_O_3_, was deduced by HRESIMS ion peak at *m/z* 317.2115 ([M + H]^+^, calcd 317.2111), which suggested seven degrees of unsaturation. The IR spectrum of **1** showed typical absorption indicative of carbonyl group (*ν*_max_ 1701 cm^−1^). Its ^1^H NMR spectrum (Table 1) displayed signals for four singlet methyls at *δ*_H_ 1.22, 1.36, 1.37, and 1.37 and three singlet olefinic protons at *δ*_H_ 4.96, 5.07, and 5.30. The ^13^C NMR, DEPT, and HSQC spectra indicated 20 carbon signals in **1**, including four methyls (*δ*_C_ 17.1, 17.4, 23.0, and 23.1), five sp^3^ methylenes (*δ*_C_ 27.7, 29.8, 30.1, 35.7, and 38.9), three sp^3^ methines (*δ*_C_ 48.1, 48.4, and 63.8), three sp^3^ quaternary carbons (*δ*_C_ 39.0, 59.5, and 88.7), one trisubstituted double bond (*δ*_C_ 98.6 and 196.2), one terminal double bond (*δ*_C_ 114.5 and 150.5), and one ketone group (*δ*_C_ 207.6) (Table 2). One ketone and two double bonds accounted for three degrees of unsaturation, suggesting the presence of a tetracyclic ring system for **1**. The ^1^H–^1^H COSY spectrum analysis of **1** defined two spin systems **a** and **b** (Figure 2) by clear cross peaks of H_2_-10 (*δ*_H_ 2.26, 1.87)/H-9 (*δ*_H_ 2.79)/H-1 (*δ*_H_ 2.45)/H_2_-2 (*δ*_H_ 1.76, 1.60)/H_2_-3 (*δ*_H_ 2.12, 0.97) (**a**) and H-5 (*δ*_H_ 2.85)/H_2_-6 (*δ*_H_ 2.16, 1.34)/H_2_-7 (*δ*_H_ 2.35, 2.17) (**b**), respectively. Based on HMBC experiment, fragments **a** and **b** could be connected by inserting the 8,19-exomethylene double bond and the oxygenated quaternary carbon C-4 (*δ*_C_ 59.5), as indicated by the clear correlations from H_2_-19 to C-7/C-8/C-9 and H_3_-20 to C-3/C-4/C-5 (Figure 2). Further HMBC correlations from H_3_-16 and H_3_-17 to C-14/C-15, from H_3_-18 to C-1/C-10/C-11/C-12, and from H-13 to C-12/C-14/C-15 constructed a xeniaphyllane skeleton. The presence of an epoxide group at C-4 and C-5 was deduced by the typical up-fielded ^13^C NMR signals appearing at *δ*_C_ 59.5 (C-4) and *δ*_C_ 63.8 (C-5). The remaining cycle was deduced to be a furanone ring by the presence of an ether bridge between C-12 and C-15, as evidenced by the typical chemical shifts of an oxygenated carbon at *δ*_C_ 88.7 and a ketone carbonyl at *δ*_C_ 196.2 [12].

**Figure 3:**
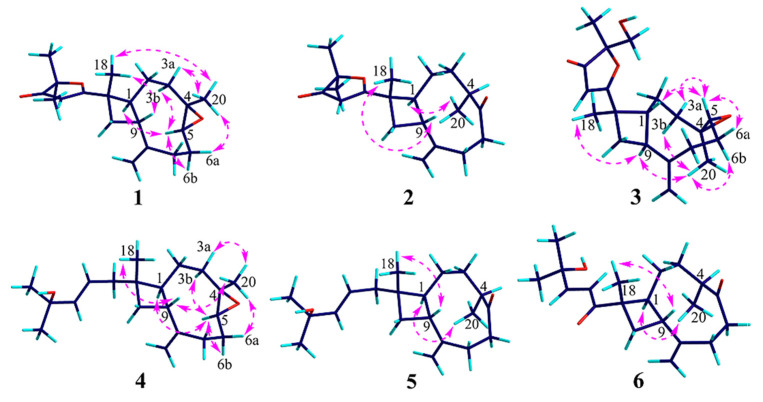
Key NOESY correlations of compounds **1**–**6**.

**Figure 4:**
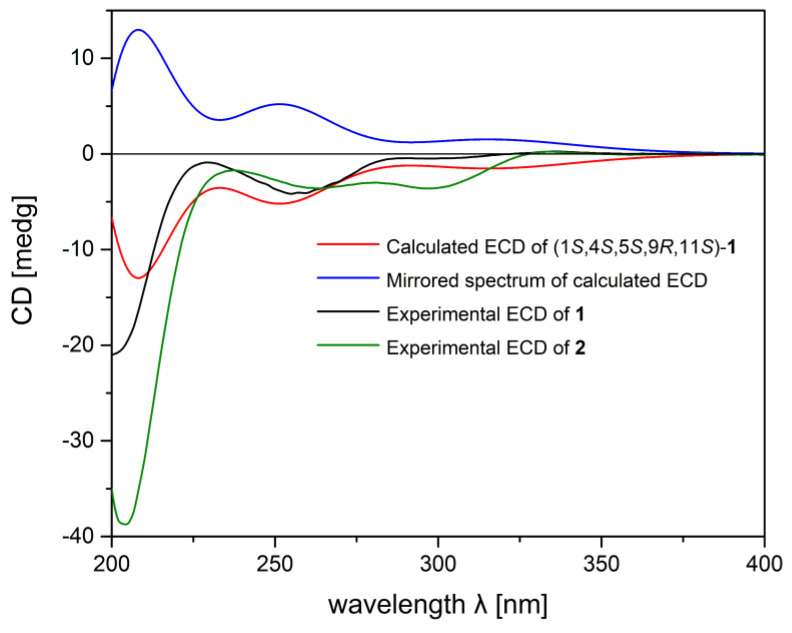
Experimental ECD spectrum of **1** (black) and **2** (green); calculated ECD spectrum of (1*S*,4*S*,5*S*,9*R*,11*S*)-**1** (red) and its enantiomer (blue).

The relative configuration (RC) of compound **1** was deduced by NOESY experiment. The crucial NOE correlations (Figure 3) of H_3_-18 (*δ*_H_ 1.36)/H-9, H_3_-18/H_3_-20 (*δ*_H_ 1.22), and H-1/H-5 proved that H_3_-18, H-9, and H_3_-20 should be the relative *β*-orientation, while H-1 and H-5 should be the relative *α*-orientation. A *trans* configuration for H-5 and H_3_-20 in the trisubstituted epoxide was supported by the clear NOE correlations of H-5/H-3b (*δ*_H_ 0.97), H_3_-20/H-3a (*δ*_H_ 2.12), and H_3_-20/H-6a (*δ*_H_ 2.16) and the lack of correlation of H-5/H_3_-20. Thus, the RC of **1** was determined to be 1S*,4S*,5S*,9R*,11S*. Given the conformational flexibility of the core cyclononane ring in **1** that makes the assignment of RC using NOE interactions between remote protons potentially unreliable, a further QM–NMR calculation was performed to further confirm the RC for **1**, which could be a showcase for the other cyclononane-containing compounds (**2**–**10**). Two possible configurations, **1a** (1S*,4S*,5S*,9R*,11S*) and **1b** (1S*,4R*,5R*,9R*,11S*), were calculated, and **1a** showed a better result than **1b**, verifying the RC of **1** to be 1S*,4S*,5S*,9R*,11S* (Figure S81). The absolute configuration (AC) of compound **1** was further established by TDDFT-ECD calculation due to the presence of an α,β-unsaturated ketone group near the chiral centers. As shown in Figure 4, the Boltzmann-averaged ECD spectrum of (1*S*,4*S*,5*S*,9*R*,11*S*)-**1** showed a highly matched ECD curve to the experimental curve of **1**, allowing the assignment of the AC of **1**.

Sinuhirfuranone B (**2**) was obtained as colorless oil, and its molecular formula C_20_H_28_O_3_, the same as that of **1**, was determined by the HRESIMS ion peak at *m/z* 317.2118 ([M + H]^+^, calcd 317.2111). In fact, the NMR spectra of **2** (Table 1 and Table 2) showed great similarities to those of **1**, with the main differences on the ^1^H and ^13^C NMR chemical shifts of C-4, C-5 and its adjacent carbons and protons, suggesting that the epoxide at C-4 (*δ*_C_ 59.5) and C-5 (*δ*_C_ 63.8) in **1** was replaced by a ketone group at C-5 (*δ*_C_ 216.6) in **2**. Then, the RC at C-1, C-4, C-9, and C-11 of **2** was elucidated by NOESY experiment. The clear NOE correlations of H-1 (*δ*_H_ 2.09)/H_3_-20 (*δ*_H_ 1.02) and H-9 (*δ*_H_ 2.56)/H_3_-18 (*δ*_H_ 1.29) indicated the relative *α*-orientation of H-1/H_3_-20 and the relative β-orientation of H-9/H_3_-18, respectively. As a result, the RC of **2** was elucidated to be 1S*,4S*,9R*,11S*. Finally, the AC of C-11 in **2** was determined to be *S* by showing the same positive cotton effect (CE) around 220 nm and negative CE around 260 nm in its CD spectrum as that of compound **1** (Figure 4).

**Figure 5:**
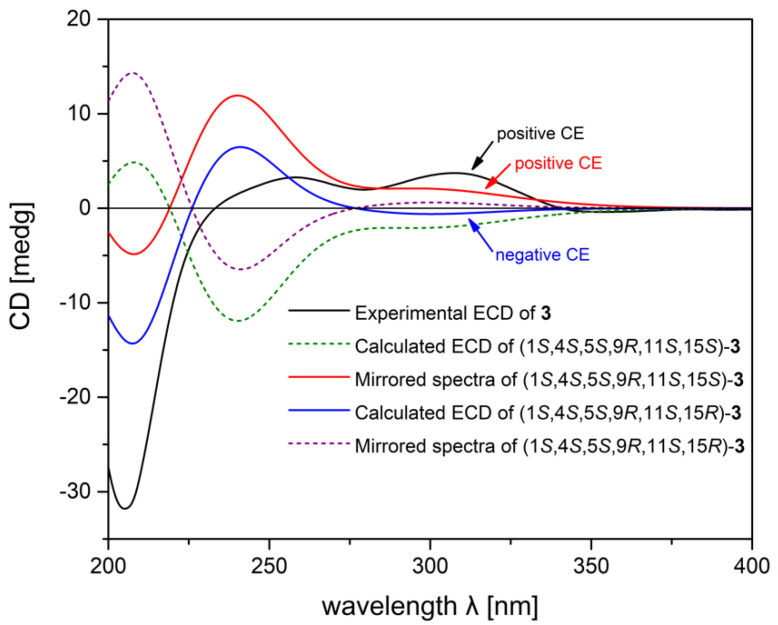
Experimental ECD spectra of **3** (black), calculated ECD spectrum of (1*S*,4*S*,5*S*,9*R*,11*S*,15*S*)-**3** (dashed green), mirrored spectra of (1*S*,4*S*,5*S*,9*R*,11*S*,15*S*)-**3** (red), calculated ECD spectrum of (1*S*,4*S*,5*S*,9*R*,11*S*,15*R*)-**3** (blue), and mirrored spectra of (1*S*,4*S*,5*S*,9*R*,11*S*,15*R*)-**3** (dashed purple).

Sinuhirfuranone C (**3**) is a colorless oil, and its molecular formula C_20_H_28_O_4_ was established by the HRESIMS ion peak at *m/z* 333.2051 ([M + H]^+^, calcd 333.2060), indicating seven degrees of unsaturation. The 1D and 2D NMR data of **3** (Table 1 and Table 2) were reminiscent of compound **1**. A detailed comparison of the NMR data of these two compounds indicated that they differed only by the substitution of a primary hydroxyl group at C-16 (*δ*_C_ 65.9) in **3** rather than a methyl group in **1**, which was further confirmed by the observation of HMBC correlations from H_3_-17 (*δ*_H_ 1.37) to C-14 (*δ*_C_ 206.1)/C-15 (*δ*_C_ 90.5)/C-16 (Figure 2). Based on the extremely similar chemical shifts of the segment C-1–C-11 with C-18–C-20 and compatible NOE correlations with **1** (Figure 3), the RC of C-1, C-4, C-5, C-9, and C-11 in **3** was deduced to be 1S*,4S*,5S*,9R*,11S*, the same as those in compound **1**. Given that the remaining chiral center C-15 is independent from other chiral centers, QM-NMR method was applied, and two possible configurations, **3a** (1S*,4S*,5S*,9R*,11S*,15R*) and **3b** (1S*,4S*,5S*,9R*,11S*,15S*), were calculated (Figure S85). However, it failed since the difference on carbon chemical shifts between the two configurations was too slight. Further, TDDFT-ECD calculation was performed to resolve this problem, and (1*S*,4*S*,5*S*,9*R*,11*S*,15*R*)-**3** and (1*S*,4*S*,5*S*,9*R*,11*S*,15*S*)-**3** were both calculated. Interestingly, as shown in Figure 5, the mirrored spectrum of (1*S*,4*S*,5*S*,9*R*,11*S*,15*S*)-**3** showed a highly matched ECD curve to the experimental curve of **3**, allowing the determination of the AC of **3** to be 1*R*,4*R*,5*R*,9*S*,11*R*,15*R*.

Sinuhirtins C–E (**4**–**6**) were isolated as colorless oil. Their molecular formulas were deduced from the HRESIMS data to be C_20_H_32_O_2_ (*m/z* 305.2482, [M + H]^+^, calcd 305.2475), C_20_H_32_O_2_ (*m/z* 327.2297, [M + Na]^+^, calcd 327.2295), and C_20_H_30_O_3_ (*m/z* 319.2275, [M + H]^+^, calcd 319.2268), respectively. They showed NMR data similar to those of co-occurring xeniaphyllane nanolobatin B (**11**) [17]. In fact, compounds **4**–**6** possessed the same skeleton as **11**, differing from each other by the oxidative patterns and double-bond positions on the skeleton. Specifically, sinuhirtin C (**4**) was the decarbonylated product of **11** at C-12 position, as indicated by an aliphatic methylene at *δ*_C_ 46.3 in **4** instead of a carbonyl group at *δ_C_* 203.7 in **11**. The NMR data of sinuhirtin D (**5**) and **4** only differed on C-4, C-5, and its adjacent carbons and protons. In fact, the epoxide group at C-4 and C-5 in **4** was substituted by the ketone group at C-5 in **5**, which was further supported by the observation of HMBC correlations from H_3_-20 (*δ*_H_ 1.00) to C-3 (*δ*_C_ 29.2)/C-4 (*δ*_C_ 47.2)/C-5 (*δ*_C_ 218.4). Sinuhirtin E (**6**) was the 12-carbonylation derivative of **5** since the chemical shift at C-12 was shifted downfield from *δ*_C_ 44.3 in **5** to *δ*_C_ 203.8 in **6**.

**Figure 6:**
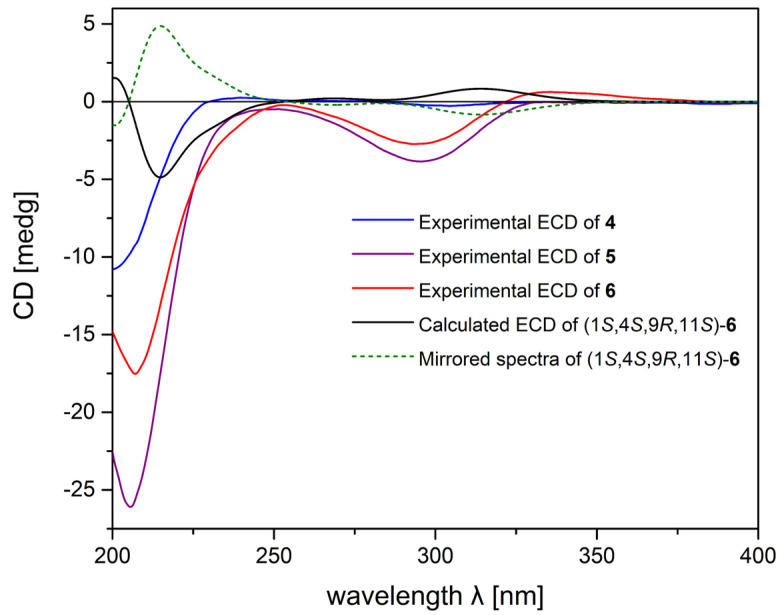
Experimental ECD spectra of **4** (blue), **5** (purple), **6** (red), calculated ECD spectrum of (1*S*,4*S*,9*R*,11*S*)-**6** (black), and mirrored spectra of (1*S*,4*S*,9*R*,11*S*)-**6** (dashed green).

The RC of compound **4** was assigned to be the same as **1** of 1S*,4S*,5S*,9R*,11S* through analysis of their NMR data and interpretation of their NOESY spectra, whereas the RCs of compounds **5** and **6** were determined to be the same as **2** of 1S*,4S*,9R*,11S* on the basis of the careful analysis of their NOESY spectra. To determine their ACs, TDDFT-ECD calculation was applied on compound **6** since the α,β-unsaturated ketone group was near its chiral center C-11 (Figure 6). On the basis of the structural similarities of compounds **4**–**6**, their ECD curves were compared with each other, resulting in the determination of the same absolute stereochemistry at C-1, C-9, and C-11 as 1*S*,9*R*,11*S*.

Sinuhirtone A (**7**) was isolated as colorless oil with the chemical formula of C_18_H_26_O_4_ as determined by the HRESIMS (*m/z* 307.1911 [M + H]^+^, calcd 307.1904), suggesting six degrees of unsaturation. The ^13^C NMR and HSQC spectra disclosed 18 carbon resonances, including three methyls (*δ*_C_ 16.4, 17.6, and 30.1), seven sp^3^ methylenes (*δ*_C_ 25.0, 27.4, 30.5, 31.4, 37.0, 37.6, and 38.5), three sp^3^ methines (*δ*_C_ 45.8, 51.3, and 61.8), two sp^3^ quaternary carbons (*δ*_C_ 48.2, and 58.9), and three carbonyls (*δ*_C_ 207.2, 212.5, and 213.3). The NMR data of **7** were strongly reminiscent of those of co-occurring gibberosin A (**14**). The overall comparison of the ^13^C NMR data of **7** and **14** revealed that the differences between them mainly happened at C-8 (*δ*_C_ 213.3 for **7**, *δ*_C_ 150.8 for **14**) and its neighboring carbons C-7 (*δ*_C_ 37.6 for **7**, *δ*_C_ 29.4 for **14**), C-6 (*δ* 25.0 for **7**, *δ*_C_ 30.2 for **14**), and C-9 (*δ*_C_ 51.3 for **7**, *δ*_C_ 47.4 for **14**), indicating that the terminal double bond at C-8 in **14** was oxidized to be a ketone carbonyl group in **7**. It is worth noting that sinuhirtone A, featuring an uncommon 17,19-dinorxeniaphyllane-type skeleton, is the first example of dinorditerpene from marine soft coral.

Sinuhirtone B (**8**) was also isolated as colorless oil. Its molecular formula, C_19_H_26_O_3_, was established by HREIMS at *m/z* 302.1883 ([M]^+^, calcd 302.1876), which suggested seven degrees of unsaturation. The ^1^H and ^13^C NMR data of **8** were almost identical to those of **14** except that C-13 and C-14 were both shifted downfield from *δ*_C_ 30.6 and 36.8 in **14** to *δ*_C_ 132.7 and 137.8 in **8**, respectively, indicating that C-13 and C-14 were oxidized to be a double bond, which was in agreement with the 2 mass-units loss of molecular weight in **8** (Table 2 and Table 3).

**Figure 7:**
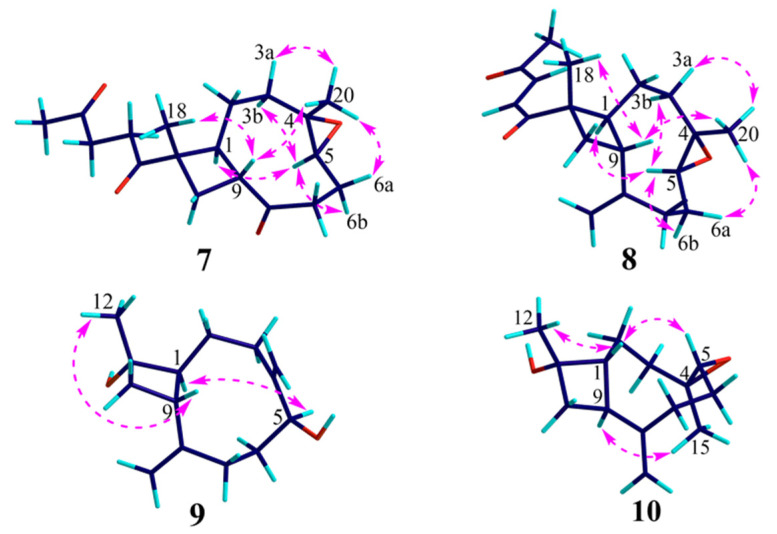
Key NOESY correlations of compounds **7**–**10**.

**Figure 8:**
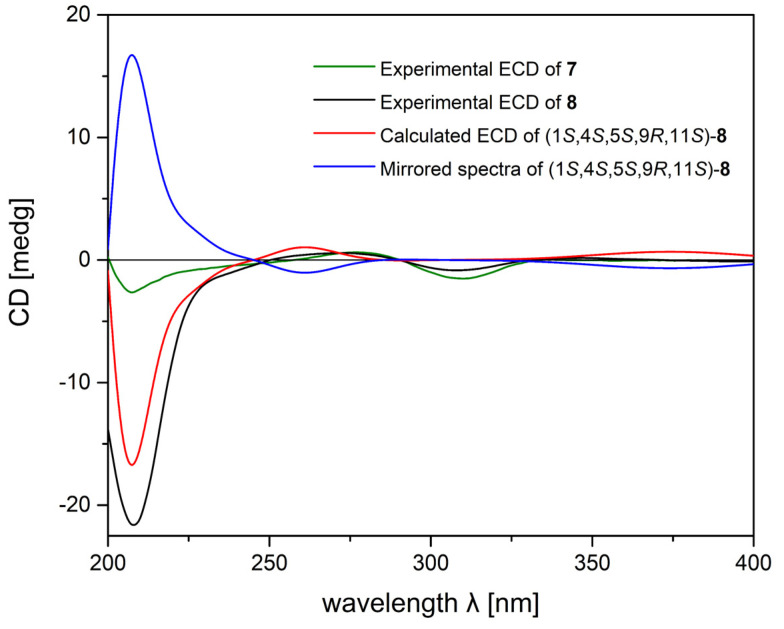
Experimental ECD spectra of **7** (green), **8** (black), calculated ECD spectrum of (1*S*,4*S*,5*S*,9*R*,11*S*)-**8** (red), and mirrored spectra of (1*S*,4*S*,5*S*,9*R*,11*S*)-**8** (blue).

The RCs of **7** and **8** were both assigned to be 1S*,4S*,5S*,9R*,11S*, which was confirmed by the NOESY correlations of H_3_-18 (*δ*_H_ 1.34)/H-9 (*δ*_H_ 3.08)/H_3_-20 (*δ*_H_ 1.30) and H-1 (*δ*_H_ 2.64)/H-5 (*δ*_H_ 2.76) in **7**, H_3_-18 (*δ*_H_ 1.34)/H-9 (*δ*_H_ 2.75)/H_3_-20 (*δ*_H_ 1.22) and H-1 (*δ*_H_ 2.47)/H-5 (*δ*_H_ 2.88) in **8**, respectively (Figure 7). Based on the assignment of RCs of compounds **7** and **8**, we further decided to perform the TDDFT-ECD calculation to assign the AC of **8** as well as compare the ECD curves of **7** and **8**. As shown in Figure 8, both the ECD spectra of **7** and **8** displayed the positive CEs at around 260 nm and negative CEs at 310 nm, which highly matched the calculated ECD of (1*S*,4*S*,5*S*,9*R*,11*S*)-**8**.

Sinuhirtin F (**9**) was isolated as colorless oil. Its molecular formula was deduced to be C_14_H_22_O_2_ based on the HRESIMS pseudo-molecular ion peak at *m/z* 223.1689 ([M + H]^+^, calcd 223.1693), indicating four degrees of unsaturation. Its ^1^H NMR spectrum (Table 3) displayed signals for one singlet methyl at *δ*_H_ 1.24 and four singlet olefinic protons at *δ*_H_ 4.79, 4.83, 4.96, and 5.05. The ^13^C NMR, DEPT, and HSQC spectra indicated 14 carbon signals in **9**, including one methyl (*δ*_C_ 21.6), five sp^3^ methylenes (*δ*_C_ 29.9, 32.3, 32.5, 33.2, and 40.3), three sp^3^ methines (*δ*_C_ 40.0, 58.1, and 75.7), one sp^3^ quaternary carbon (*δ*_C_ 70.7), and two terminal double bonds (*δ*_C_ 110.0, 114.6, 150.9, and 151.1) (Table 2). Two double bonds accounts for two out of the four degrees of unsaturation, suggesting a bicyclic ring system for **9**. The planar structure of **9** was further elucidated by detailed 2D NMR analysis. The ^1^H–^1^H COSY correlations of H-10 (*δ*_H_ 2.00)/H-9 (*δ*_H_ 1.88)/H-1 (*δ*_H_ 1.86)/H_2_-2 (*δ*_H_ 1.97, 1.56)/H_2_-3 (*δ*_H_ 2.57, 1.95) and H-5 (*δ*_H_ 4.12)/H_2_-6 (*δ*_H_ 1.95, 1.79)/H_2_-7 (*δ*_H_ 2.35, 2.09) established two structure fragments in **9** (Figure 2). The connection of these fragments, bearing in mind two terminal double bonds and one methyl, were deduced by the careful analysis of the clear HMBC correlations from H_2_-14 (*δ*_H_ 4.83, 4.79) to C-7 (*δ*_C_ 33.2)/C-8 (*δ*_C_ 151.1)/C-9 (*δ*_C_ 40.0), from H_2_-15 (*δ*_H_ 5.05, 4.96) to C-3 (*δ*_C_ 32.5)/C-4 (*δ*_C_ 150.9)/C-5 (*δ*_C_ 75.7), and from H_3_-12 to C-1 (*δ*_C_ 58.1)/C-10 (*δ*_C_ 40.3)/C-11 (*δ*_C_ 70.7), constructing a norcaryophyllene skeleton. Thus, the planar structure of **9** was established as shown in Figure 1.

To determine the RC of **9** by NOESY correlations is risky since there are two overlapped proton signals at H-1 (*δ*_H_ 1.86) and H-9 (*δ*_H_ 1.87). To tackle this problem, bearing in mind the existence of four chiral centers, eight possible configurations (**9a**: 1R*,5S*,9S*,11S*; **9b**: 1R*,5S*,9R*,11S*; **9c**: 1R*,5R*,9S*,11S*; **9d**: 1R*,5R*,9R*,11S*; **9e**: 1S*,5S*,9S*,11S*; **9f**: 1S*,5S*,9R*,11S*; **9g**: 1S*,5R*,9S*,11S*; and **9h**: 1S*,5R*,9R*,11S*) were calculated by the same QM-NMR method as that for **1** and **3**. As a result, **9f** showed the highest DP4+ probability (96.73%) among the eight models, indicating the RC of **9** to be 1S*,5S*,9R*,11S*. With the solid establishment of RC of **9**, next step was to determine its AC. The initial efforts to resolve the AC of **9** by using TDDFT-ECD approach failed due to the lack of strong chromophore near the chiral centers in **9**. In turn, we applied the theoretical specific rotation calculation since it has been proven to be a convincible method for elucidating ACs of natural products [21,22]. The specific rotations of **9** in MeOH solution were measured at the sodium D line and at 589 nm ([α] −40.5). The specific rotations at 589 nm were calculated using TDDFT at the B3LYP/6-311+G* levels with the results for 1*S*,5*S*,9*R*,11*S*. The calculated conformationally averaged rotation ([α] −50.5) was in good agreement with experimental rotation value, indicating the AC of **9** to be 1*S*,5*S*,9*R*,11*S*. (Table S11)

Sinuhirtin G (**10**) was also a colorless oil, the molecular formula of which was determined by the HRESIMS (*m/z* 223.1688 [M + H]^+^, calcd 223.1693). The NMR data of **10** (Table 2 and Table 3) showed great similarities to those of nanonorcaryophyllene A, a known norcaryophyllenoid isolated from the Taiwan soft coral *Sinularia nanolobata* [17]. The overall comparison of the ^13^C NMR data of **10** and **17** revealed that the differences between them mainly happened at C-9 (*δ*_C_ 48.2 for **10**, *δ*_C_ 44.9 for **17**), C-10 (*δ*_C_ 40.5 for **10**, *δ*_C_ 42.6 for **17**), C-11 (*δ* 73.6 for **10**, *δ*_C_ 71.1 for **17**), and C-12 (*δ*_C_ 28.3 for **10**, *δ*_C_ 21.5 for **17**), indicating **10** to be likely the epimer of **17**. The RC of **10** was deduced to be 1R*,4R*,5R*,9S*,11S* based on the apparent NOESY correlations of H_3_-12 (*δ*_H_ 1.27)/H-1 (*δ*_H_ 1.87)/H-5 (*δ*_H_ 2.83) and H-9 (*δ*_H_ 2.88)/H_3_-15 (*δ*_H_ 1.25) (Figure 7). Then, the AC of **10** was assigned as 1*R*,4*R*,5*R*,9*S*,11*S* using the same optical rotation calculation as that of **9**.

**Scheme 1:**
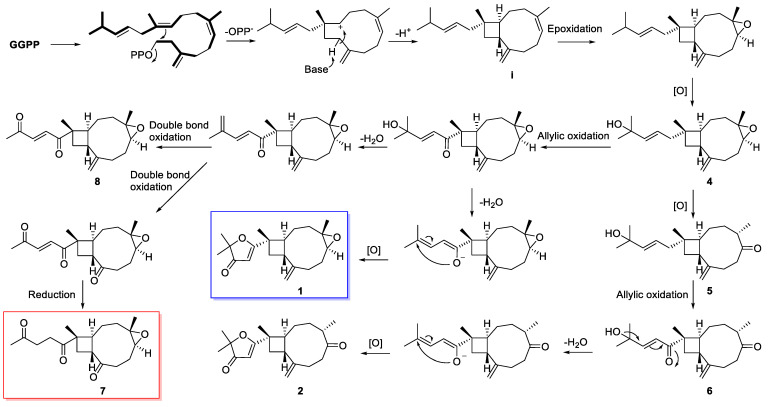
Plausible biosynthetic connection between compounds **1**–**9** (OPP: diphosphate).

Sinuhirtone A (**7**) features the first 17,19-dinorxeniaphyllane skeleton, and sinuhirfuranones A–C (**1**–**3**) possess the rare 2,2-dimethylfuran-3-one group, which are structurally different from other xeniaphyllanoids. However, they do share the same 4/9 bicyclic ring system, indicating that they should be biosynthetic related. Therefore, a plausible biosynthetic connection between **1** and **9** was proposed. As shown in Scheme 1, geranylgeranyl pyrophosphate (GGPP) could be envisaged as an early linear diterpene precursor to form the xeniaphyllane-type skeleton (**i**) under double-bond migration and ring closure steps, while new compounds were generated after a series of different reactions, including epoxidation, allylic oxidation, elimination, double-bond oxidation, and/or reduction, and so on.

In bioassay, new compounds **2**–**4**, **7**, and **8** were evaluated for in vitro cytotoxicity against four cancer cell lines, including Capan-1, A549, HT-29, and SNU-398, while showing no obvious cytotoxicity towards these cancer cell lines.

## 3. Materials and Methods

### 3.1. General Experimental Procedures

Optical rotations were measured on a PerkinElmer 241MC polarimeter. IR spectrum was recorded on a Nicolet iS50 spectrometer (Thermo Fisher Scientific, Madison, WI, USA). ^1^H and ^13^C NMR spectra were acquired on a Bruker AVANCE III 500 and 600 spectrometer. Chemical shifts are reported with the residual CHCl_3_ (*δ*_H_ 7.26 ppm) as the internal standard for ^1^H NMR spectrometry and CDCl_3_ (*δ*_C_ 77.2 ppm) for ^13^C NMR spectrometry. The LREIMS and HREIMS data were recorded on a Finnigan-MAT-95 mass spectrometer (Finnigan-MAT, San Jose, CA, USA). HRESIMS spectra were recorded on an Agilent G6250 Q-TOF (Agilent, Santa Clara, CA, USA). All solvents used for column chromatography and HPLC were of analytical grade (Shanghai Chemical Reagents Co., Ltd., Shanghai, China) and chromatographic grade (Dikma Technologies Inc., Beijing, China), respectively. Sephadex LH-20 (Pharmacia, Peapack, NJ, USA) was also used for column chromatography. Commercial silica gel (Qingdao Haiyang Chemical Co., Ltd., Qingdao, China, 100–200 and 300–400 mesh) was used for column chromatography, and precoated silica gel GF254 plates (Sinopharm Chemical Reagent Co., Shanghai, China) were used for analytical TLC. Reversed-phase (RP) HPLC was performed on an Agilent 1260 series liquid chromatograph equipped with a DAD G1315D detector at 210 nm (Agilent, Santa Clara, CA, USA). An Agilent semipreparative XDB-C18 column (5 μm, 250 × 9.4 mm) was employed for the purification.

### 3.2. Biological Material

The soft coral *S. hirta*, a voucher specimen No. YAL-50, was collected by scuba at a depth of −15 m to −20 m off Yalong bay, Sanya, China, in 2006 and identified by Professor Xiu-Bao Li from Hainan University, Hainan, China. The voucher sample *S.* *hirta* has been deposited at the Shanghai Institute of Materia Medica, CAS, under registration of No. YAL-50.

### 3.3. Extraction and Isolation

The frozen animals (210 g, dry weight) were cut into pieces and extracted exhaustively with acetone at room temperature (3 × 3.0 L). The organic extract was evaporated to give a brown residue, which was partitioned between Et_2_O and H_2_O. The Et_2_O solution was concentrated under reduced pressure to give a dark-brown residue (9.8 g), which was fractionated by silica gel column chromatography and eluting with a step gradient (0–100% diethyl ether (EE) in petroleum ether (PE)), yielding twelve fractions (Fr. A–L). Fr. D was further purified by Sephadex LH-20 [PE/CH_2_Cl_2_/MeOH (2:1:1)], followed by RP-HPLC [MeCN-H_2_O (80:20), 3.0 mL/min], to give compounds **2** (1.1 mg), **5** (1.6 mg), **8** (1.5 mg), **10** (1.6 mg), and **12** (1.3 mg). Fr. E was further purified by silica gel CC (300–400 mesh) and eluted with PE-EE (80:20), followed by RP-HPLC [MeCN-H_2_O (70:30), 3.0 mL/min] to give compounds **1** (3.2 mg), **3** (0.9 mg), **6** (1.5 mg), **7** (1.1 mg), and **9** (4.1 mg). Purification of Fr. H and Fr. J by Sephadex LH-20 [PE/CH_2_Cl_2_/MeOH (2:1:1)] and RP-HPL C [MeCN-H_2_O (60:40), 3.0 mL/min] yielded compounds **4** (1.3 mg), **11** (1.7 mg), **13** (2.0 mg), **14** (2.2 mg), **15** (1.4 mg), and **16** (1.5 mg).

### 3.4. Spectroscopic Data of Compounds

Sinuhirfuranone A (**1**): colorless oil; [*α*${]}_{D}^{20}$ +26.2 (*c* 0.10, MeOH); UV (MeOH) *λ*_max_ (log *ε*) 265 (3.14); IR (KBr) *ν*_max_: 2973, 2931, 2928, 2861, 1701, 1582, 1195, 1180, 1076 cm^−1^; ^1^H and ^13^C NMR data, see Table 1 and Table 2; HRMS (ESI) *m/z*: [M + H]^+^ Calcd. for C_20_H_29_O_3_, 317.2111; Found 317.2115.

Sinuhirfuranone B (**2**): colorless oil; [*α*${]}_{D}^{20}$ −14.0 (*c* 0.10, MeOH); UV (MeOH) *λ*_max_ (log *ε*) 264 (2.64); IR (KBr) *ν*_max_: 2988, 2870, 2928, 1701, 1380, 1142 cm^−1^; ^1^H and ^13^C NMR data, see Table 1 and Table 2; HRMS (ESI) *m/z*: [M + H]^+^ Calcd. for C_20_H_29_O_3_, 317.2111; Found 317.2118.

Sinuhirfuranone C (**3**): colorless oil; [*α*${]}_{D}^{20}$ +42.2 (*c* 0.10, MeOH); UV (MeOH) *λ*_max_ (log *ε*) 265 (2.65); IR (KBr) *ν*_max_: 3440, 2978, 2932, 2869, 1698, 1574, 1455, 1381, 1142 cm^−1^; ^1^H and ^13^C NMR data, see Table 1 and Table 2; HRMS (ESI) *m/z*: [M + H]^+^ Calcd. for C_20_H_29_O_4_, 333.2060; Found 333.2051.

Sinuhirtin C (**4**): colorless oil; [*α*${]}_{D}^{20}$ −4.3 (*c* 0.10, MeOH); IR (KBr) *ν*_max_: 3399, 2925, 2854, 1180, 1142, 1132, 1076 cm^−1^; ^1^H and ^13^C NMR data, see Table 1 and Table 2; HRMS (ESI) *m/z*: [M + H]^+^ Calcd. for C_20_H_33_O_2_, 305.2475; Found 305.2482.

Sinuhirtin D (**5**): colorless oil; [*α*${]}_{D}^{20}$ −15.0 (*c* 0.10, MeOH); IR (KBr) *ν*_max_: 3363, 2988, 2870, 1709, 1379, 1142 cm^−1^; ^1^H and ^13^C NMR data, see Table 1 and Table 2; HRMS (ESI) *m/z*: [M + Na]^+^ Calcd. for C_20_H_32_NaO_2_, 327.2295; Found 327.2297.

Sinuhirtin E (**6**): colorless oil; [*α*${]}_{D}^{20}$ −18.6 (*c* 0.10, MeOH); IR (KBr) *ν*_max_: 3446 2989, 2932, 2869, 1688, 1627, 1380, 1196, 1142, 1076 cm^−1^; ^1^H and ^13^C NMR data, see Table 1 and Table 2; HRMS (ESI) *m/z*: [M + H]^+^ Calcd. for C_20_H_31_O_3_, 319.2268; Found 319.2275.

Sinuhirtone A (**7**): colorless oil; [*α*${]}_{D}^{20}$ +63.0 (*c* 0.10, MeOH); IR (KBr) *ν*_max_: 2978, 2932, 2869, 1699, 1381, 1142 cm^−1^; ^1^H and ^13^C NMR data, see Table 2 and Table 3; HRMS (ESI) *m/z*: [M + H]^+^ Calcd. for C_18_H_27_O_4_, 307.1904; Found 307.1911.

Sinuhirtone B (**8**): colorless oil; [*α*${]}_{D}^{20}$ +53.7 (*c* 0.10, MeOH); UV (MeOH) *λ*_max_ (log *ε*) 276 (2.31), 231 (2.67); IR (KBr) *ν*_max_: 2977, 2934, 2869, 1703, 1682, 1455, 1384, 1142, 1076 cm^−1^; ^1^H and ^13^C NMR data, see Table 2 and Table 3; HRMS (EI) *m/z*: [M]^+^ Calcd. for C_19_H_26_O_3_, 302.1876; Found 302.1883.

Sinuhirtin F (**9**): colorless oil; [*α*${]}_{D}^{20}$ −40.5 (*c* 0.10, MeOH); IR (KBr) *ν*_max_: 3342, 2959, 2920, 1257, 1180, 1147, 1138, 1078 cm^−1^; ^1^H and ^13^C NMR data, see Table 2 and Table 3; HRMS (ESI) *m/z*: [M + H]^+^ Calcd. for C_14_H_23_O_2_, 223.1693; Found 223.1689.

Sinuhirtin G (**10**): colorless oil; [*α*${]}_{D}^{20}$ −97.3 (*c* 0.10, MeOH); IR (KBr) *ν*_max_: 3439, 2978, 2931, 2869, 1454, 1383, 1227, 1142, 1075 cm^−1^; ^1^H and ^13^C NMR data, see Table 2 and Table 3; HRMS (ESI) *m/z*: [M + H]^+^ Calcd. for C_14_H_23_O_2_, 223.1693; Found 223.1688.

### 3.5. QM-NMR Calculational Section

Conformational search was performed by using the torsional sampling (MCMM) approach and OPLS_2005 force field within an energy window of 21 kJ/mol. Conformers above 1% Boltzmann populations were re-optimized at the B3LYP/6-311G(d,p) level with the IEFPCM solvent model for chloroform. Frequency analysis was also carried out to confirm that the re-optimized geometries were at the energy minima. Subsequently, NMR calculations were performed at the PCM/mPW1PW91/6-31G(d) level, as recommended for DP4+. NMR shielding constants were calculated by using the GIAO method. Finally, shielding constants were averaged over the Boltzmann distribution obtained for each stereoisomer and correlated with the experimental data [12].

### 3.6. TDDFT-ECD Calculational Section

Conformational search was carried out by using the torsional sampling (MCMM) method and OPLS_2005 force field within an energy window of 21 kJ/mol. Conformers above 1% Boltzmann populations were re-optimized at the MPW1PW91/6-31G(d) level with the IEFPCM solvent model for MeCN. Frequency analysis was also carried out to confirm that the re-optimized geometries were at the energy minima. ECD spectra were obtained by TDDFT calculations showed with the identical functional basis set and solvent model as the energy optimization. At last, the Boltzmann-averaged ECD spectra of the compounds were obtained with SpecDis 1.62 [23].

### 3.7. Optical Rotation Calculational Section

The specific optical rotation calculations for compounds were carried out by using Information Gaussian 09. The conformers were further optimized at the B3LYP/6-311G(d,p) level with the IEFPCM solvent model for chloroform, all of which were subjected to specific optical rotation calculations at the B3LYP/6-311+G(d) level in chloroform with SMD model. The calculated specific optical rotation values of these conformers were averaged according to the Boltzmann distribution theory and their relative Gibbs free energy. The predicted ORD curves were generated from specific rotations calculated at four different wavelengths [24].

### 3.8. Bioassay

Cytotoxicity assays were carried out by using Capan-1 (human pancreatic cancer), A549 (human lung cancer), HT-29 (human colon carcinoma), and SNU-398 (human hepatocellular carcinoma), following a previously described procedure for a modification of the MTT colorimetric method [25,26]. To measure the cytotoxicity of tested compounds, five concentrations with three replications were performed on each cell line. Vincristine was used as a positive control for the bioassays using Capan-1, A549, HT-29, and SNU-398 cell lines, respectively.

## 4. Conclusions

In summary, the systematic chemical investigation of *S. hirta* of South China Sea yielded a series of terpenoids **1**–**10** with different skeletons, which increased the chemical diversity and complexity of marine terpenoids. The stereochemistry of these compounds was assigned by QM-NMR, TDDFT-ECD, and optical rotation calculations, which provided different approaches to deal with the challenges of stereochemistry determination of the complex molecules without single crystals. Structurally, sinuhirtone A (**7**) represents the first example of 17,19-dinorxeniaphyllane-type skeleton, whereas sinuhirfuranones A–C (**1**–**3**) possess the 2,2-dimethylfuran-3-one group, which is rare among marine diterpenoids. Although some new compounds did not show obvious anti-cancer activities, our proposed biogenetic pathway of these interesting compounds should impel further study on their accumulation for more extensive biological evaluation to understand the role they play on the life cycle of soft coral and to find their possible medicinal application.

## Figures and Tables

**Table 1:** ^1^H NMR data (*δ* in ppm, *J* in Hz) for compounds **1**–**6** in CDCl_3_.

No.	1 *^a^*	2 *^a^*	3 *^a^*	4 *^a^*	5 *^a^*	6 *^a^*
	*δ*_H_ Mult (*J* Hz)	*δ*_H_ Mult (*J* Hz)	*δ*_H_ Mult (*J* Hz)	*δ*_H_ Mult (*J* Hz)	*δ*_H_ Mult (*J* Hz)	*δ*_H_ Mult (*J* Hz)
1	2.45 t (9.6)	2.09 m	2.48 t (9.6)	1.89 t (9.6)	1.90 t (7.8)	2.12 m
2a	1.76 dt (14.4, 3.6)	1.66 m	1.77 m	1.64 m	1.51 m	1.73 m
2b	1.60 m	1.37 m	1.60 m	1.43 m	1.24 m	1.37 m
3a	2.12 dt (13.2, 3.6)	1.84 m	2.10 m	2.06 dt (13.2, 3.6)	1.88 m	1.92 m
3b	0.97 td (13.2, 5.4)	1.62 m	0.98 td (13.2, 4.8)	0.96 td (13.2, 4.8)	1.68 m	1.67 m
4		2.52 m			2.52 m	2.54 m
5	2.85 dd (10.8, 4.2)		2.86 dd (10.2, 4.2)	2.88 dd (10.2, 4.2)		
6a	2.16 ddd (13.2, 7.8, 4.8)	2.51 m	2.27 m	2.13 ddd (15.0, 7.8, 4.2)	2.53 m	2.53 m
6b	1.34 overlap		1.32 m	1.32 m		
7a	2.35 m	2.49 m	2.37 ddd (13.2, 8.4, 4.2)	2.33 ddd (13.2, 8.4, 4.2)	2.45 m	2.51 m
7b	2.17 m		2.17 m	2.25 ddt (12.0, 7.8, 4.2)		2.48 m
9	2.79 q (9.6)	2.56 m	2.81 q (9.6)	2.64 q (9.6)	2.50 m	2.53 m
10a	2.26 m	2.16 t (10.8)	2.29 m	1.65 m	1.61 m	2.06 t (10.2)
10b	1.87 dd (10.8, 8.4)	1.80 dd (10.8, 8.4)	1.89 dd (10.8,8.4)		1.52 m	1.77 dd (11.4, 8.4)
12				2.00 d (6.6)	1.91 m	
13	5.30 s	5.26 s	5.37 s	5.59 overlap	5.55 ddd (15.6, 7.8, 6.6)	6.47 d (15.0)
14				5.61 overlap	5.61 d (15.6)	6.92 d (15.0)
16a	1.37 s	1.38 s	3.81 d (12.0)	1.30 s	1.33 s	1.40 s
16b			3.69 d (12.0)			
17	1.37 s	1.37 s	1.37 s	1.30 s	1.39 s	1.40 s
18	1.36 s	1.29 s	1.39 s	1.03 s	0.98 s	1.21 s
19a	5.07 s	4.96 s	5.07 s	4.97 s	4.86 s	4.91 s
19b	4.96 s	4.95 s	4.96 s	4.86 s	4.82 s	4.91 s
20	1.22 s	1.02 s	1.21 s	1.19 s	1.00 d (6.6)	1.03 d (6.6)

*^a^* Recorded at 600 MHz. Chemical shifts (ppm) refer to CHCl_3_ (*δ*_H_ 7.26). Assignments were deduced by analysis of 1D and 2D NMR spectra.

**Table 2:** ^13^C NMR and DEPT spectroscopic data for compounds **1**–**10** in CDCl_3_.

No.	1 *^a^*	2 *^a^*	3 *^a^*	4 *^a^*	5 *^a^*	6 *^a^*	7 *^a^*	8 *^a^*	9 *^a^*	10 *^a^*
	*δ*_C_ Mult.	*δ*_C_ Mult.	*δ*_C_ Mult.	*δ*_C_ Mult.	*δ*_C_ Mult.	*δ*_C_ Mult.	*δ*_C_ Mult.	*δ*_C_ Mult.	*δ*_C_ Mult.	*δ*_C_ Mult.
1	48.4 CH	49.0 CH	48.7 CH	48.8 CH	44.6 CH	45.7 CH	45.8 CH	45.5 CH	58.1 CH	52.7 CH
2	27.7 CH_2_	27.1 CH_2_	27.7 CH_2_	27.9 CH_2_	26.2 CH_2_	27.0 CH_2_	27.4 CH_2_	28.3 CH_2_	29.8 CH_2_	26.1 CH_2_
3	38.9 CH_2_	30.2 CH_2_	38.8 CH_2_	39.0 CH_2_	29.2 CH_2_	29.5 CH_2_	38.5 CH_2_	38.7 CH_2_	32.5 CH_2_	39.0 CH_2_
4	59.5 qC	48.2 CH	59.5 qC	59.9 qC	47.2 CH	48.1 CH	58.9 qC	59.5 qC	150.9 qC	59.8 qC
5	63.8 CH	216.6 qC	63.7 CH	64.0 CH	218.4 qC	217.5 qC	61.8 CH	63.6 CH	75.7 CH	63.4 CH
6	30.1 CH_2_	41.8 CH_2_	30.1 CH_2_	30.3 CH_2_	43.4 CH_2_	41.9 CH_2_	25.0 CH_2_	30.1 CH_2_	32.3 CH_2_	29.9 CH_2_
7	29.8 CH_2_	32.3 CH_2_	29.8 CH_2_	29.9 CH_2_	31.4 CH_2_	32.1 CH_2_	37.6 CH_2_	29.9 CH_2_	33.2 CH_2_	30.8 CH_2_
8	150.5 qC	151.9 qC	150.4 qC	151.9 qC	153.3 qC	152.2 qC	213.3 qC	150.5 qC	151.1 qC	151.4 qC
9	48.1 CH	42.7 CH	48.1 CH	48.8 CH	44.0 CH	42.2 CH	51.3 CH	47.4 CH	40.0 CH	48.2 CH
10	35.7 CH_2_	35.0 CH_2_	35.6 CH_2_	38.1 CH_2_	35.7 CH_2_	34.4 CH_2_	31.4 CH_2_	35.2 CH_2_	40.3 CH_2_	40.5 CH_2_
11	39.0 qC	38.9 qC	39.2 qC	37.3 qC	38.1 qC	47.7 qC	48.2 qC	48.2 qC	70.7 qC	73.6 qC
12	196.2 qC	196.2 qC	198.1 qC	46.3 CH_2_	44.3 CH_2_	203.8 qC	212.5 qC	203.2 qC	21.7 CH_3_	28.3 CH_3_
13	98.6 CH	98.5 CH	100.4 CH	123.1 CH	123.4 CH	120.7 CH	30.5 CH_2_	132.7 CH		
14	207.6 qC	207.8 qC	206.1 qC	140.7 CH	141.5 CH	153.3 CH	37.0 CH_2_	137.8 CH	110.0 CH_2_	113.0 CH_2_
15	88.7 qC	88.7 qC	90.5 qC	70.9 qC	70.4 qC	71.1 qC	207.2 qC	197.9 qC	114.6 CH_2_	16.9 CH_3_
16	23.0 CH_3_	23.0 CH_3_	65.9 CH_2_	30.2 CH_3_	33.0 CH_3_	29.3 CH_3_	30.1 CH_3_	29.3 CH_3_		
17	23.1 CH_3_	23.1 CH_3_	18.4 CH_3_	30.1 CH_3_	29.4 CH_3_	29.4 CH_3_				
18	17.4 CH_3_	17.4 CH_3_	17.4 CH_3_	19.8 CH_3_	21.0 CH_3_	16.6 CH_3_	17.6 CH_3_	16.7 CH_3_		
19	114.5 CH_2_	112.9 CH_2_	114.6 CH_2_	113.1 CH_2_	112.0 CH_2_	112.7 CH_2_		114.4 CH_2_		
20	17.1 CH_3_	16.2 CH_3_	17.1 CH_3_	17.2 CH_3_	15.2 CH_3_	15.6 CH_3_	16.4 CH_3_	17.2 CH_3_		

*^a^* Recorded at 125 MHz in CDCl_3_. Chemical shifts (ppm) refer to CHCl_3_ (*δ*_C_ 77.2). Assignments were deduced by analysis of 1D and 2D NMR spectra.

**Table 3:** ^1^H NMR data (*δ* in ppm, *J* in Hz) for compounds **7**–**10** in CDCl_3_.

No.	7 *^a^*	8 *^b^*	9 *^a^*	10 *^a^*
	*δ*_H_ Mult (*J* Hz)	*δ*_H_ Mult (*J* Hz)	*δ*_H_ Mult (*J* Hz)	*δ*_H_ Mult (*J* Hz)
1	2.64 m	2.47 t (9.5)	1.86 m	1.87 t (9.6)
2a	1.84 dt (15.0, 3.6)	1.83 m	1.97 m	1.74 dt (14.4, 4.2)
2b	1.64 m	1.59 m	1.56 m	1.65 m
3a	2.15 dt (13.2, 3.6)	2.12 m	2.57 dt (13.2, 4.2)	2.16 dt (13.2, 3.6)
3b	1.09 td (13.2, 4.8)	1.10 td (13.0, 5.0)	1.95 m	1.00 td (13.2, 4.8)
5	2.76 dd (10.2, 4.2)	2.88 dd (10.5, 4.5)	4.12 dd (9.6, 3.6)	2.83 dd (10.2, 4.2)
6a	2.40 m	2.25 m	1.95 m	2.24 m
6b	1.46 m	1.33 m	1.79 m	1.35 m
7a	2.57 m	2.30 m	2.35 ddd (14.4, 10.2, 4.2)	2.35 m
7b		2.13 m	2.09 m	2.09 m
9	3.08 q (9.0)	2.75 q (9.5)	1.88 m	2.88 q (9.6)
10a	2.63 m	2.15 m	2.00 m	2.02 m
10b	1.79 dd (11.4, 7.8)	1.90 dd (11.0, 8.0)		1.93 m
12			1.24 s	1.27 s
13	2.65 m	6.98 s		
14a	2.73 m	6.98 s	4.83 s	5.02 s
14b			4.79 s	4.93 s
15a			5.05 s	1.25 s
15b			4.96 s	
16	2.19 s	2.36 s		
18	1.34 s	1.34 s		
19a		5.00 s		
19b		4.93 s		
20	1.30 s	1.22 s		

*^a^* Recorded at 600 MHz. *^b^* Recorded at 500 MHz. Chemical shifts (ppm) refer to CHCl_3_ (*δ*_H_ 7.26). Assignments were deduced by analysis of 1D and 2D NMR spectra.

## Data Availability

Data are contained within the article or Supplementary Material.

## Supplementary Materials

The following are available online at https://www.mdpi.com/article/10.3390/md20040272/s1: 1D and 2D NMR, HRMS, and IR spectra of **1**–**10**; DP4+ calculation of compounds **3** and **9**; TDDFT-ECD calculation of compounds **1**, **3**, **6**, and **8**; ORD calculation of compounds **9** and **10**.
