# Supplementary material for: Sinuhirtone A, An Uncommon 17,19-Dinorxeniaphyllanoid, and Nine Related New Terpenoids from the Hainan Soft Coral Sinularia hirta
Source: Mar Drugs. 2022 Apr 18;20(4):272. doi: 10.3390/md20040272 (PMC9030993; doi:10.3390/md20040272)
Supplement: Supplementary file 1 [file marinedrugs-20-00272-s001.zip › marinedrugs-1686906-supplementary.pdf]

Supporting Information

Sinuhirtone A, A Novel 17,19-Dinorxeniaphyllanoid, and Three Uncommon Diterpenoids from the Hainan Soft Coral *Sinularia hirta*

Zi-Hui Chen,[1,2] Si-Qi Lu,[1,3] Guan-Ying Han,[3,*] Xu-Wen Li,[1,2,4,*] and Yue-Wei Guo[1,2,4,*]

1 State Key Laboratory of Drug Research, Shanghai Institute of Materia Medica, Chinese Academy of Sciences, 555 Zu Chong Zhi Road, Zhangjiang Hi-Tech Park, Shanghai, 201203, China; s18-chenzihui@simm.ac.cn (Z.-H.C.); siqi.lu@hengrui.com (S.-Q.L.)

2 University of Chinese Academy of Sciences, No. 19A Yuquan Road, Beijing 100049, China

3 Jinzhou Medical University, Jinzhou 121001, China

4 Drug Discovery Shandong Laboratory, Bohai Rim Advanced Research Institute for Drug Discovery, Yantai, Shandong 264117, China

* Correspondence: hanguanying@fjbiopharma.com (G.-Y.H.); xwli@simm.ac.cn (X.-W.L.); ywguo@simm.ac.cn (Y.-W.G.); Tel.: +86-21-50805813 (Y.-W.G.)

Contents

1. NMR, MS, and IR spectra for compounds **1–10** 3

2. Computational Section..85

2.1 QM-NMR calculation for compounds **1**, **3**, and **9** 85

2.1.1 QM-NMR calculation for compound **1**..85

2.1.2 QM-NMR calculation for compound **3**..87

2.1.3 QM-NMR calculation for compound **9**..89

2.2 ECD calculation for compound **1**, **3**, **6**, and **8**..121

2.2.1 ECD calculation for (1*S*,4*S*,5*S*,9*R*,11*S*)-**1**...121

2.2.2 ECD calculation for (1*S*,4*S*,5*S*,9*R*,11*S*,15*S*)-**3**....................................125

2.2.3 ECD calculation for (1*S*,4*S*,5*S*,9*R*,11*S*,15*R*)-**3** 134

2.2.4 ECD calculation for (1*S*,4*S*,9*R*,11*S*)-**6**..143

2.2.5 ECD calculation for (1*S*,4*S*,5*S*,9*R*,11*S*)-**8**..147

2.3 Specific rotation calculation for compounds **9** and **10** 153

1. NMR, MS, and IR spectra for compounds 1–10

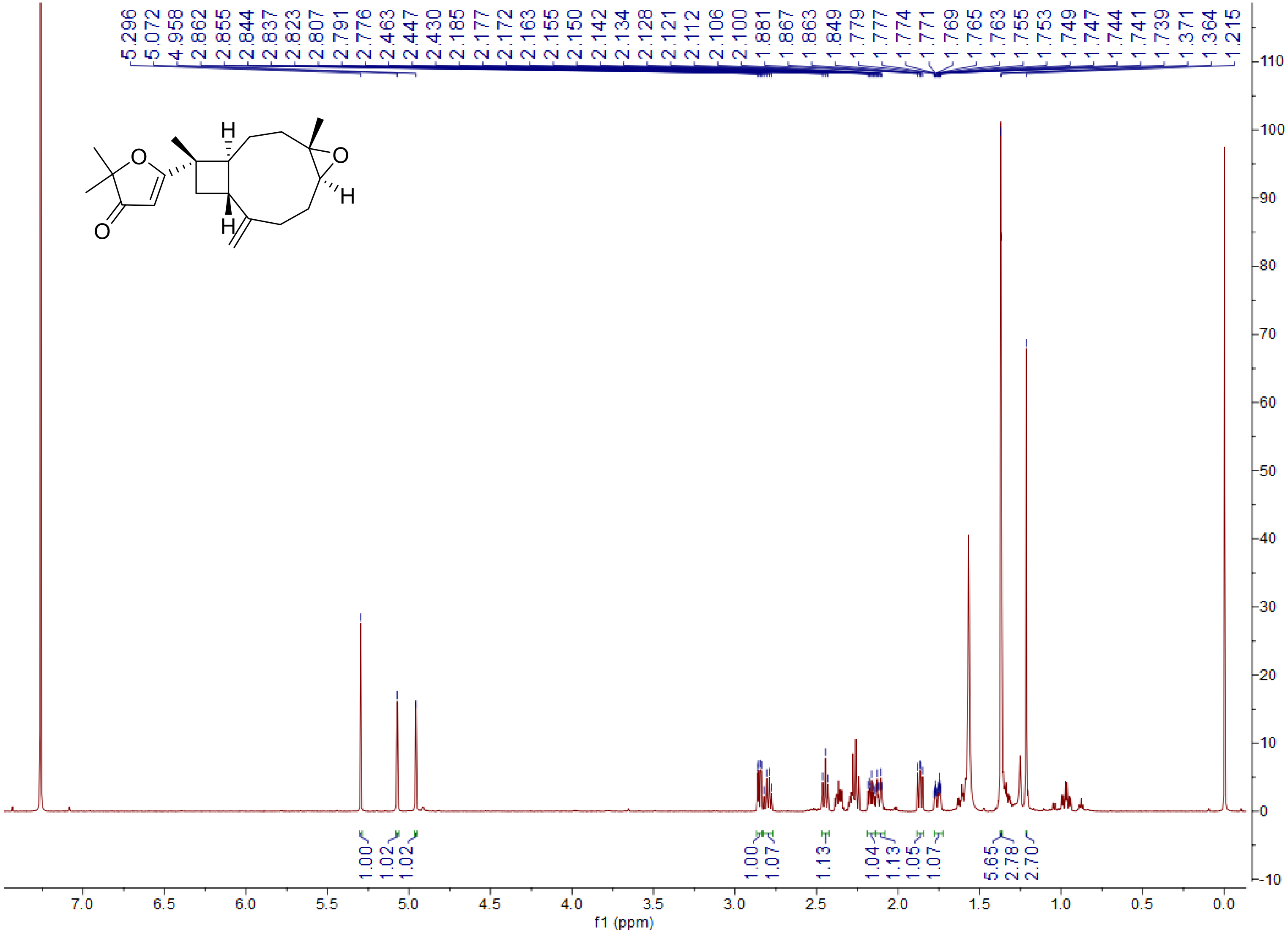

Figure S1. ^{1}H NMR spectrum (600 MHz) of sinuhirfuranone A (**1**) in $CDCl_3$.

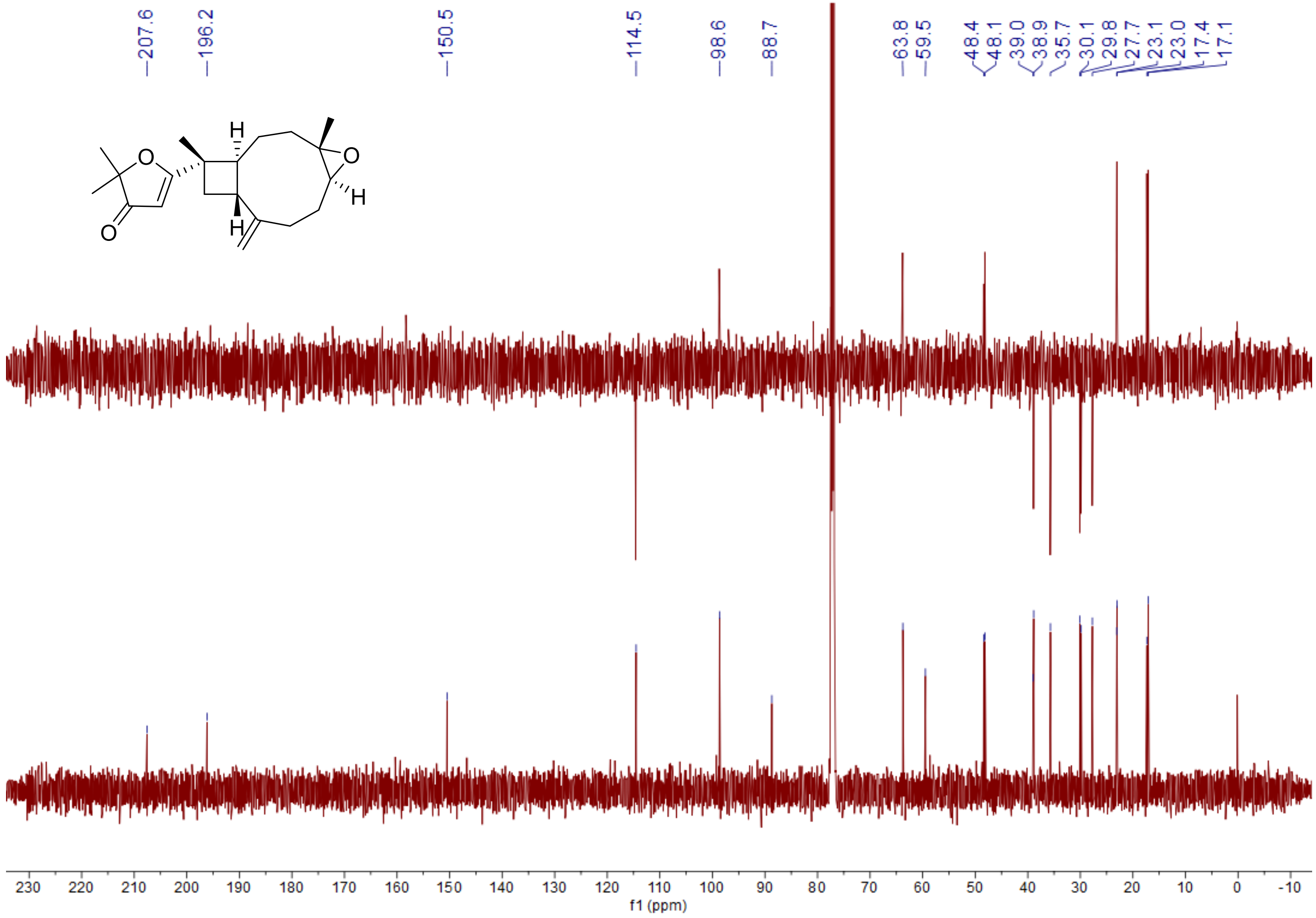

Figure S2. ^{13}C NMR spectrum (125 MHz) of sinuhirfuranone A (**1**) in $CDCl_3$.

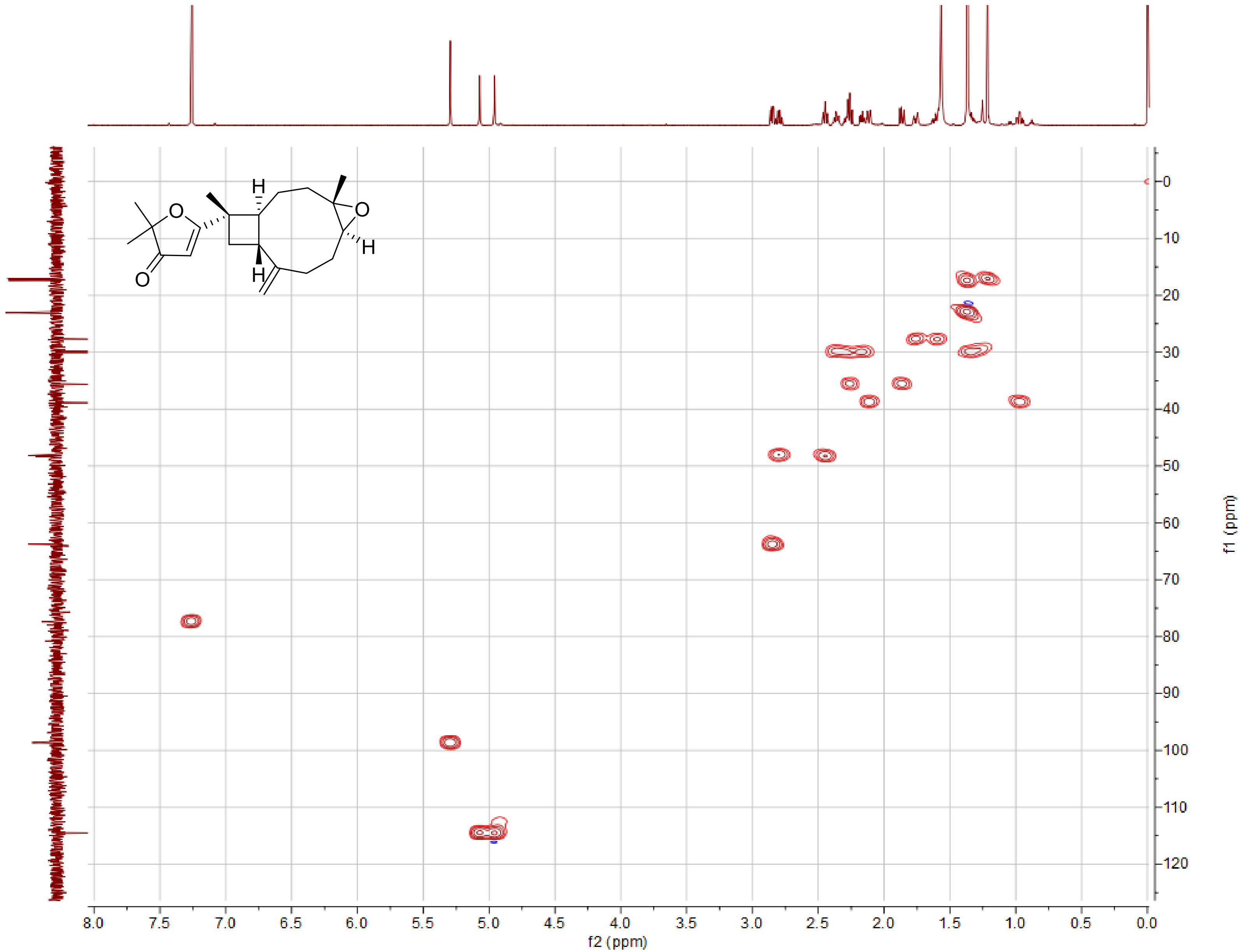

Figure S3. HSQC spectrum (500 MHz) of sinuhirfuranone A (**1**) in $CDCl_3$.

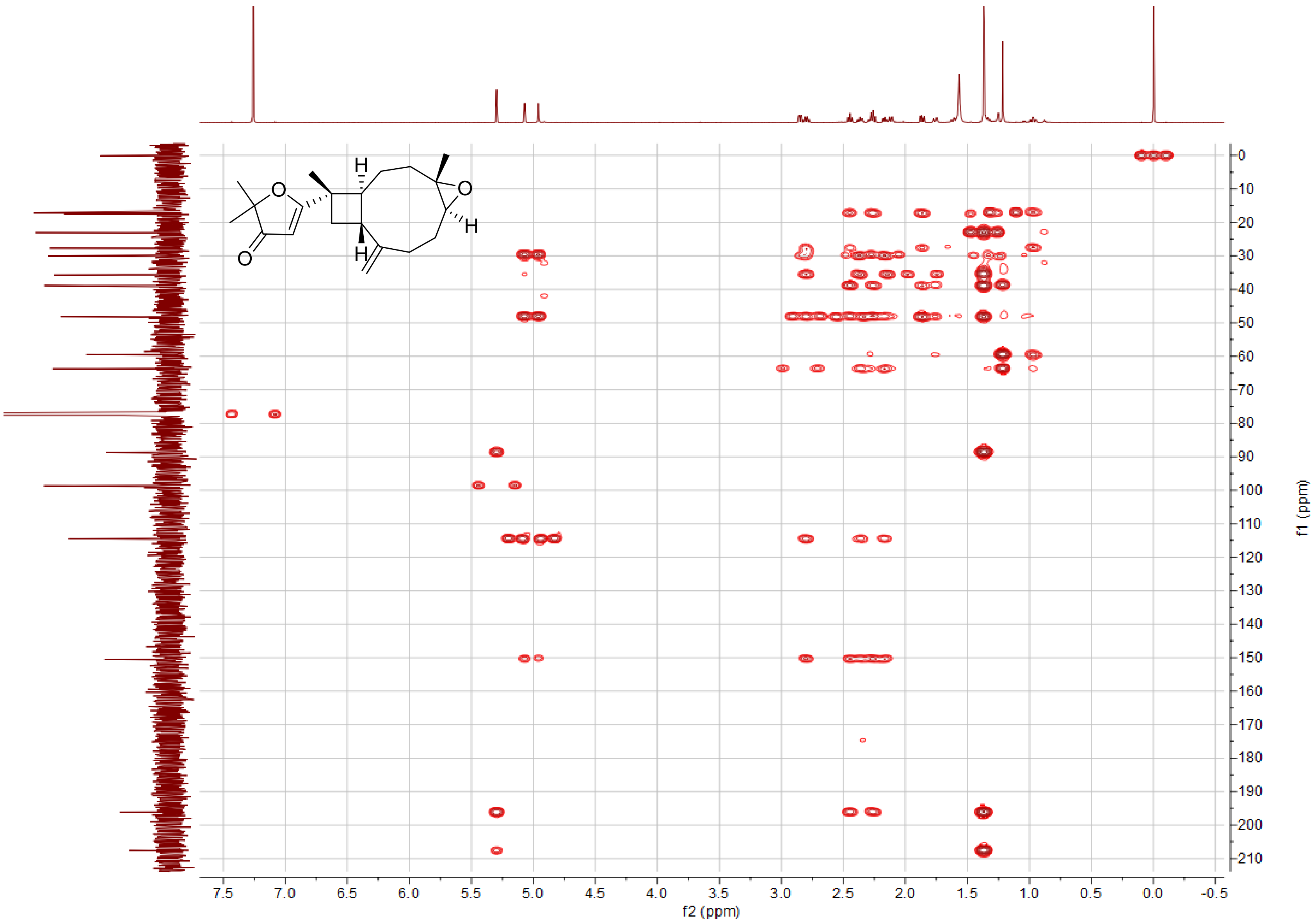

Figure S4. HMBC spectrum (500 MHz) of sinuhirfuranone A (**1**) in $CDCl_3$.

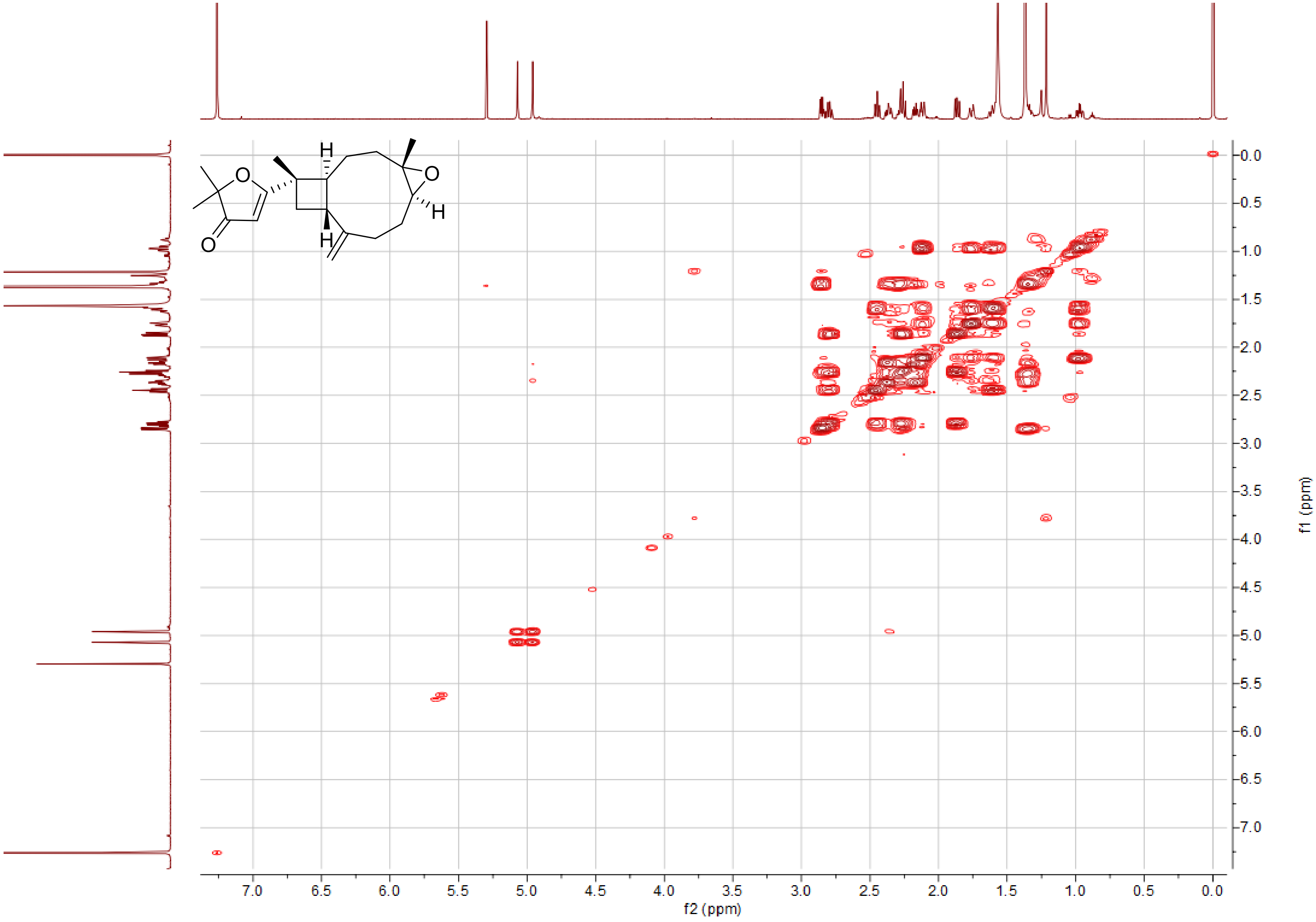

Figure S5. COSY spectrum (500 MHz) of sinuhirfuranone A (**1**) in $CDCl_3$.

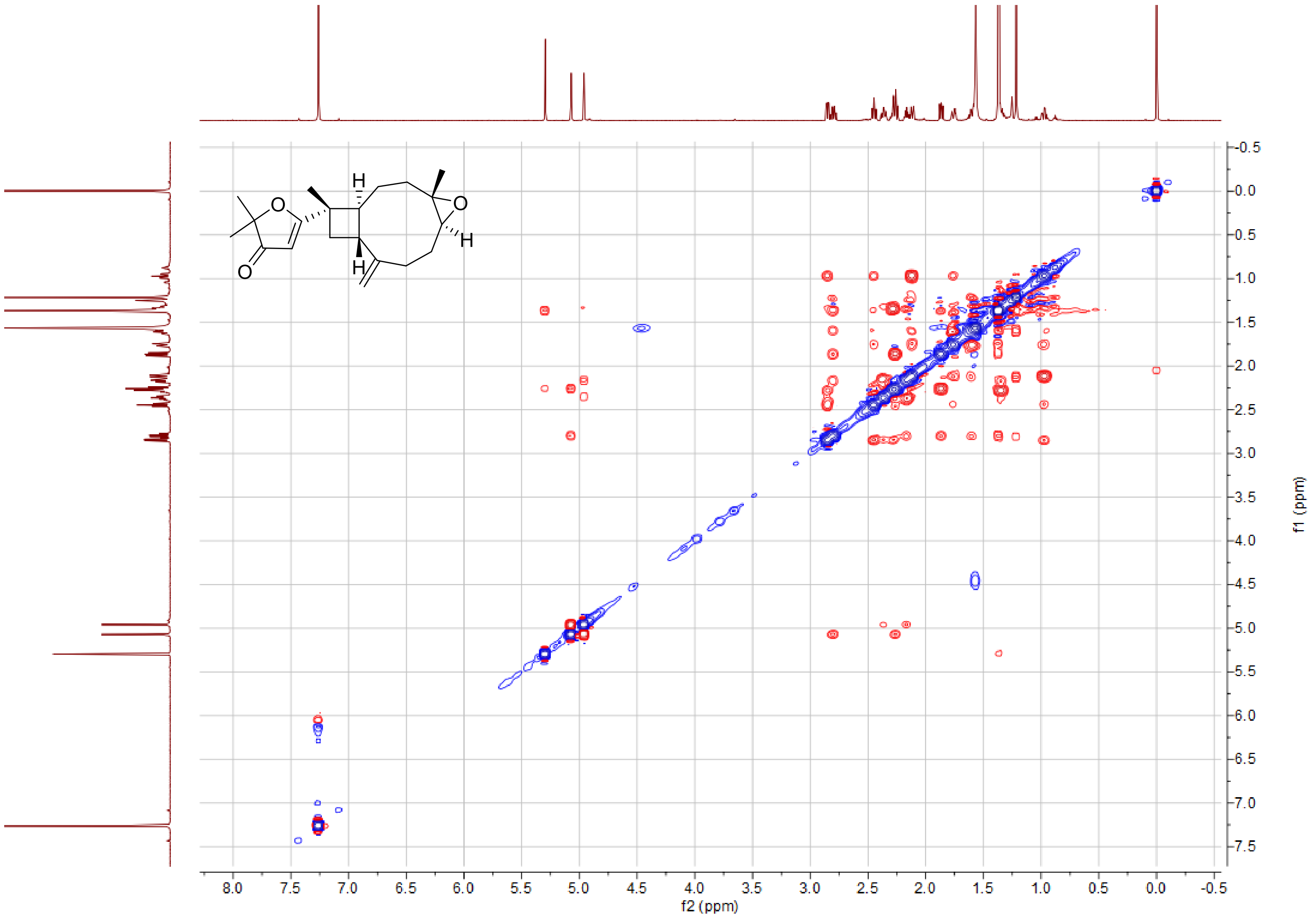

Figure S6. NOESY spectrum (500 MHz) of sinuhirfuranone A (**1**) in $CDCl_3$.

Fragmentor Voltage	Collision Energy	Ionization Mode
125	0	ESI

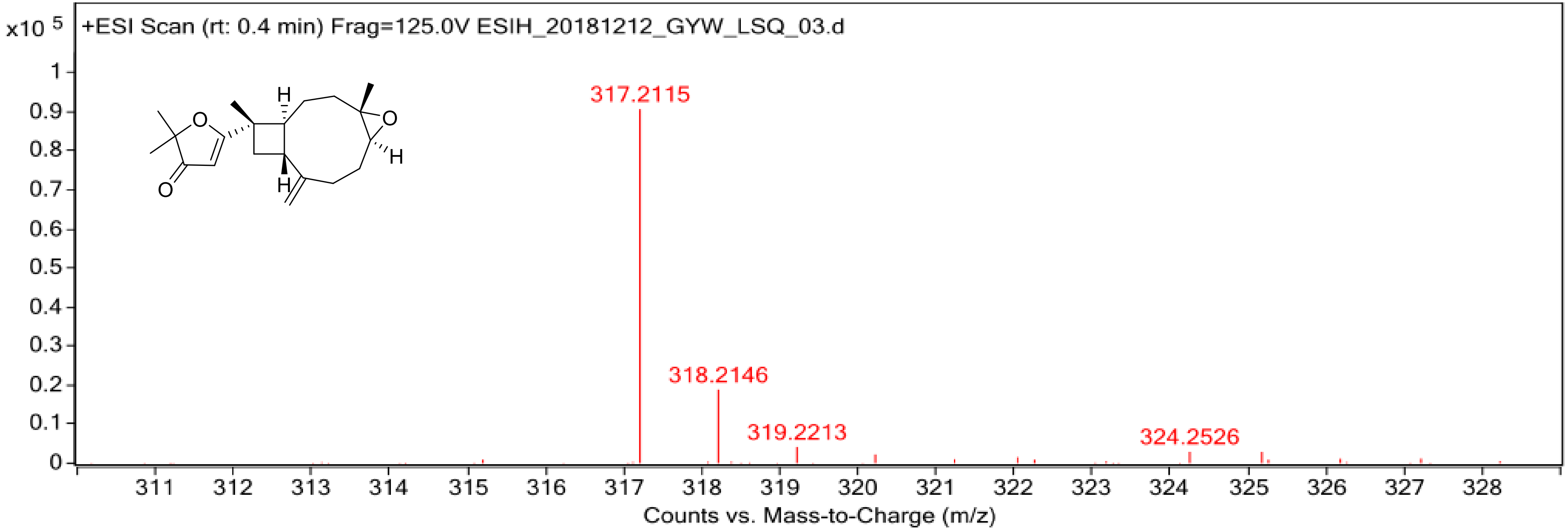

Formula Calculator Results

m/z	Calc m/z	Diff (mDa)	Diff (ppm)	Ion Formula	Ion
317.2115	317.2111	-0.42	-1.33	C20 H29 O3	(M+H)+

Figure S7. HRESIMS spectrum of sinuhirfuranone A (**1**) in MeOH.

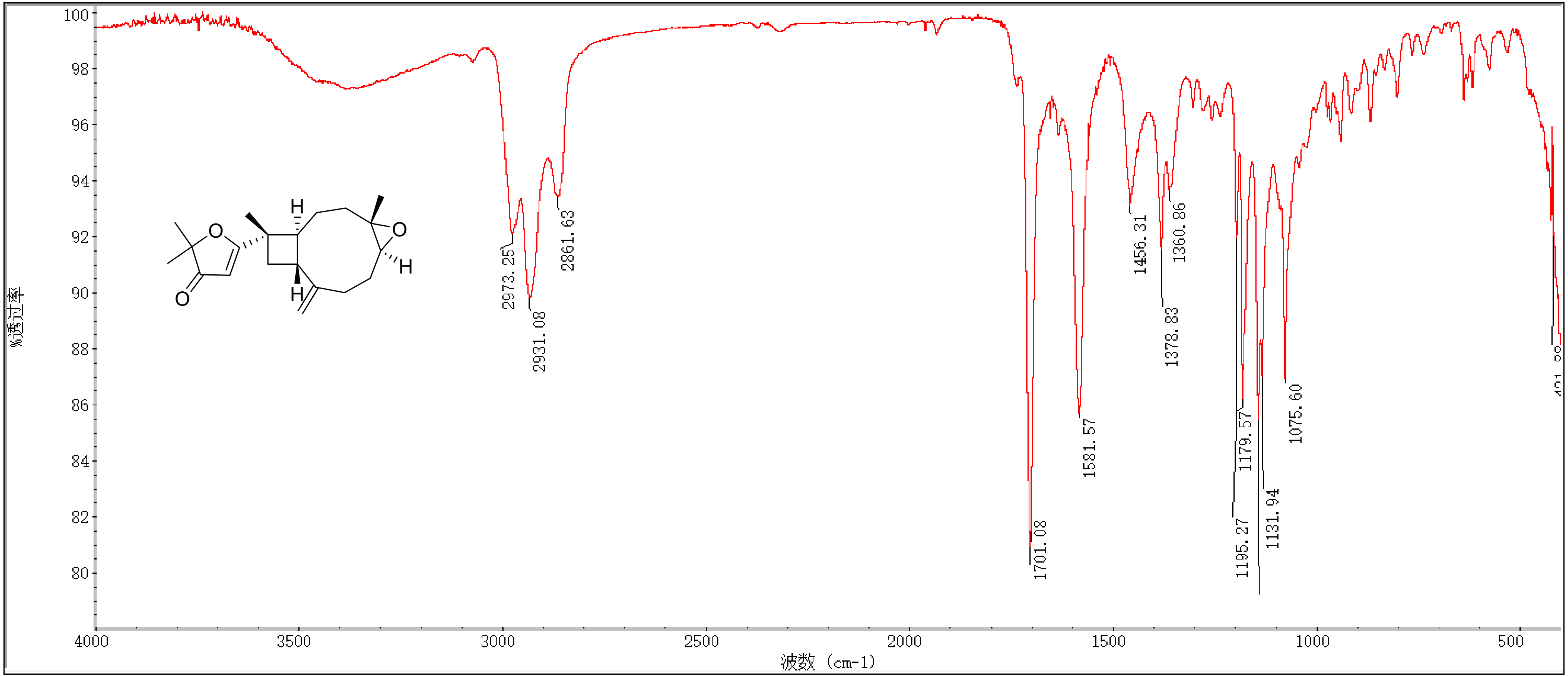

Figure S8. IR spectrum of sinuhirfuranone A (**1**).

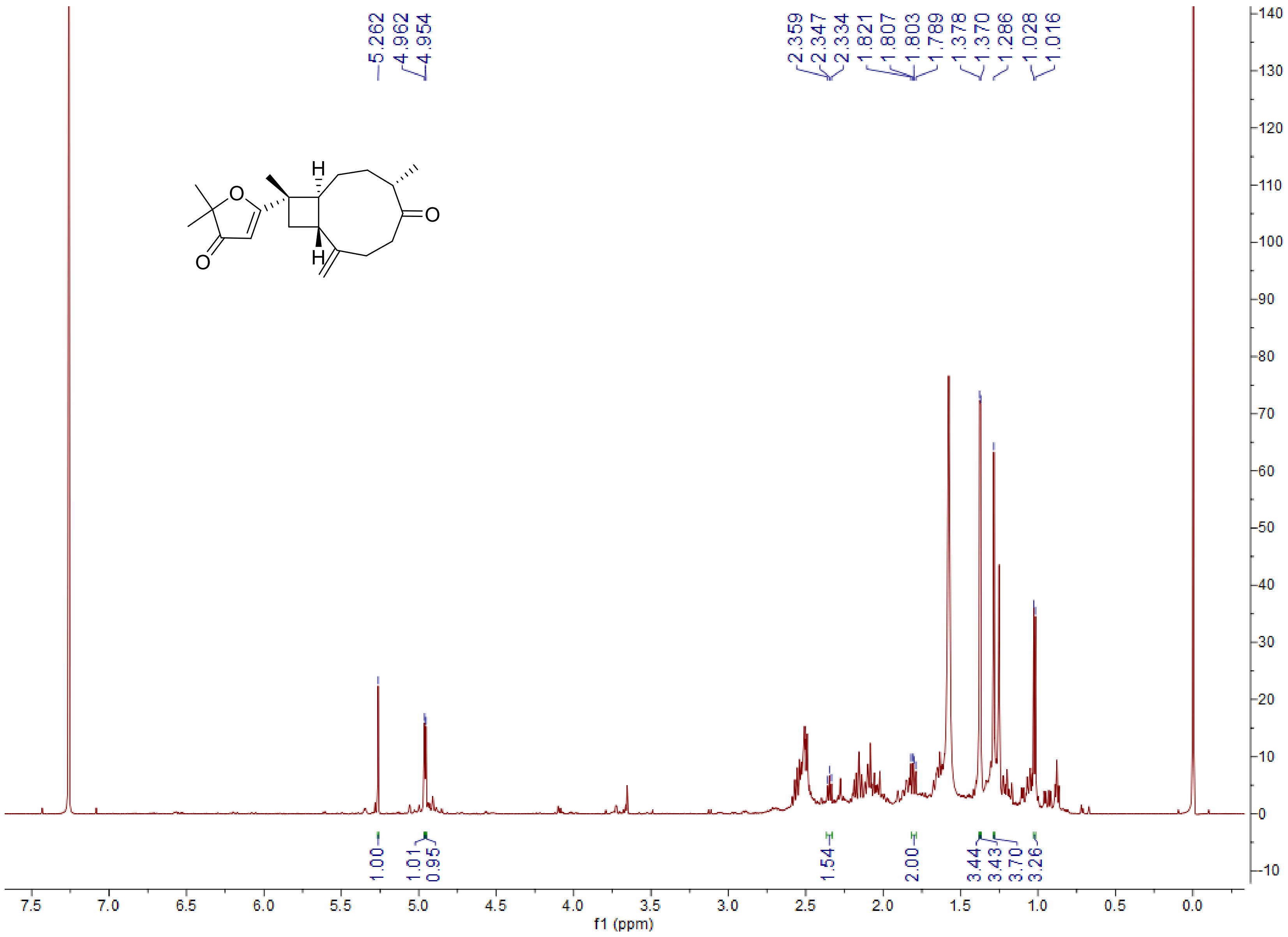

Figure S9. ^{1}H NMR spectrum (600 MHz) of sinuhirfuranone B (**2**) in $CDCl_3$.

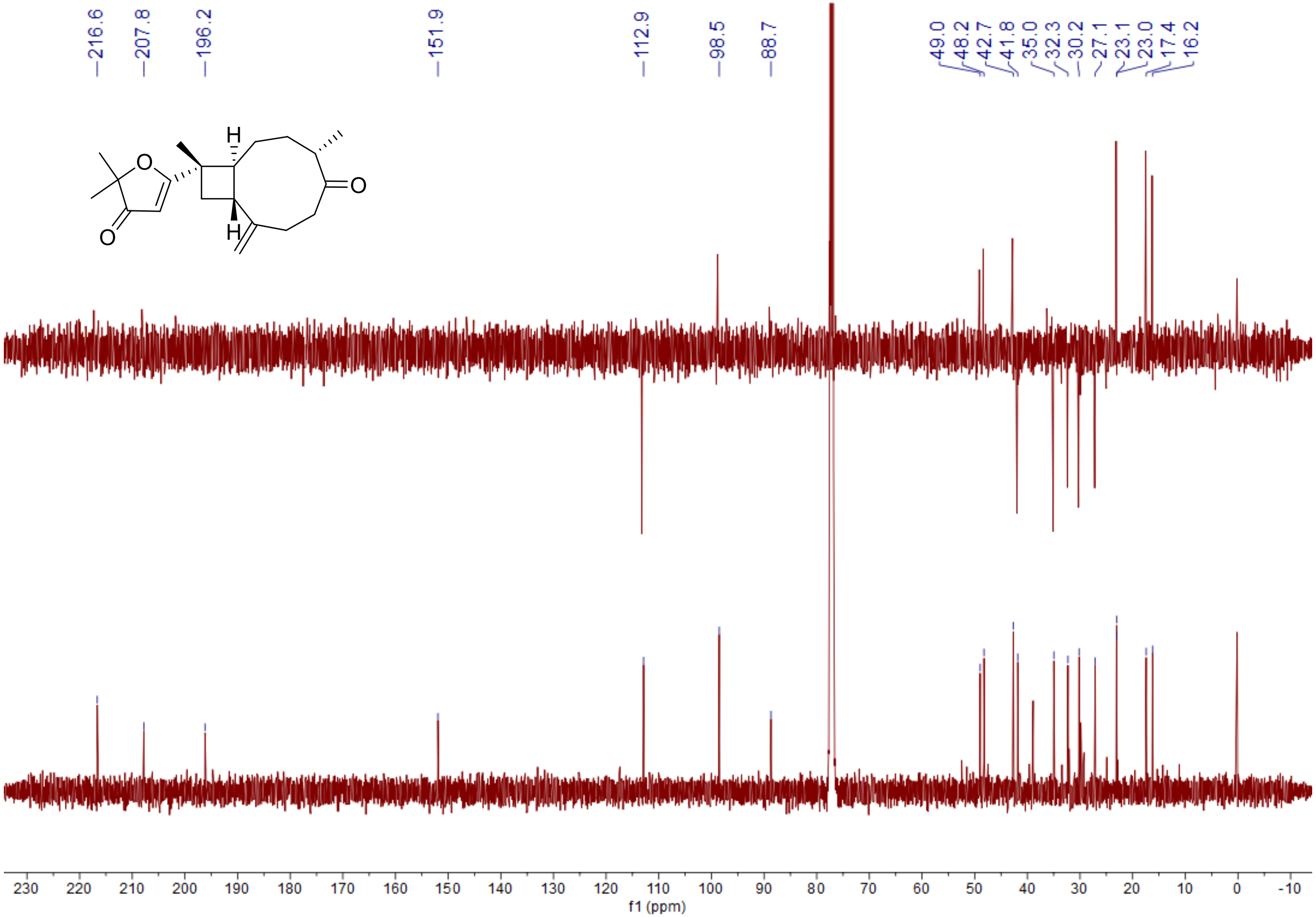

Figure S10. ^{13}C NMR spectrum (125 MHz) of sinuhirfuranone B (**2**) in $CDCl_3$.

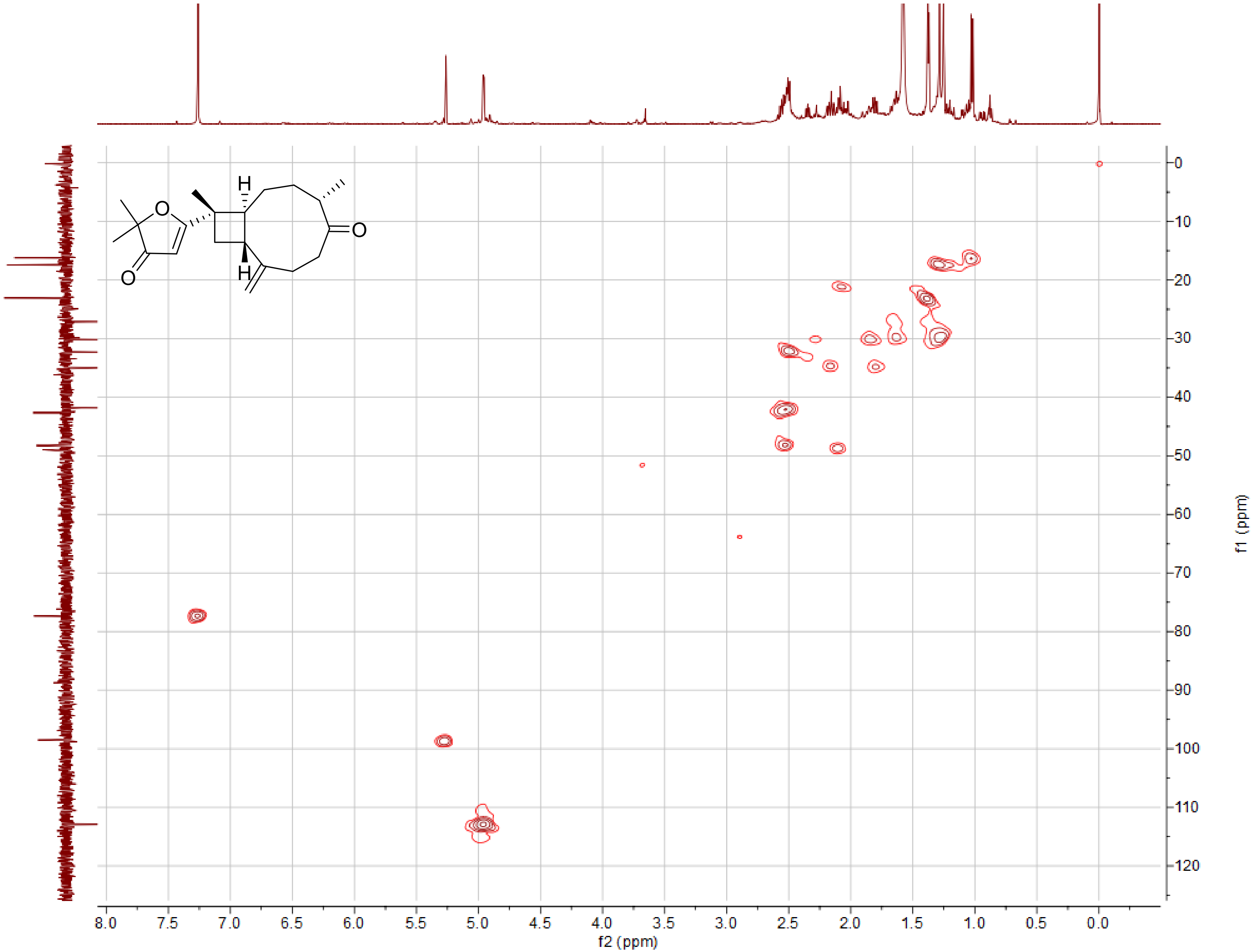

Figure S11. HSQC spectrum (500 MHz) of sinuhirfuranone B (**2**) in $CDCl_3$.

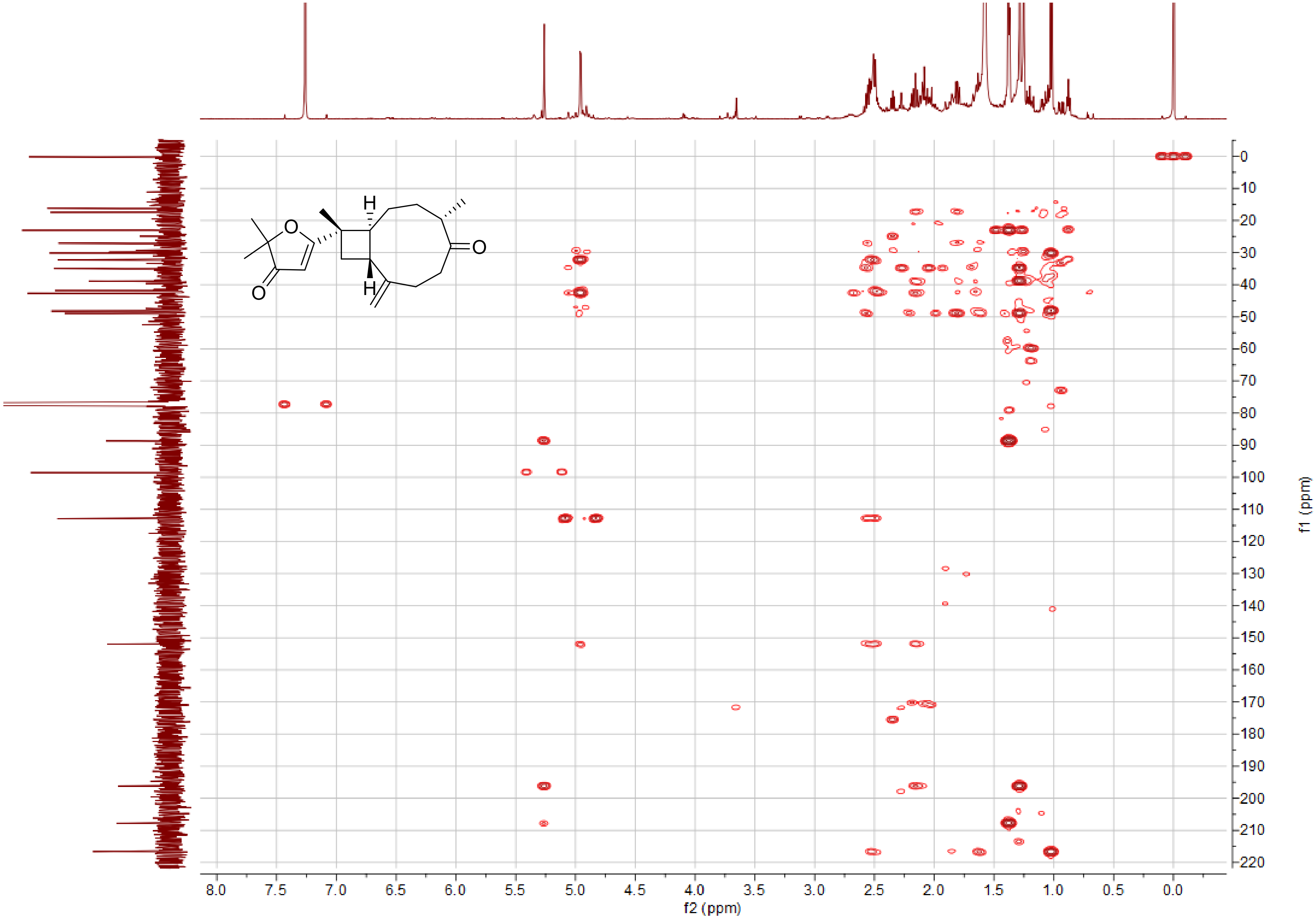

Figure S12. HMBC spectrum (500 MHz) of sinuhirfuranone B (**2**) in $CDCl_3$.

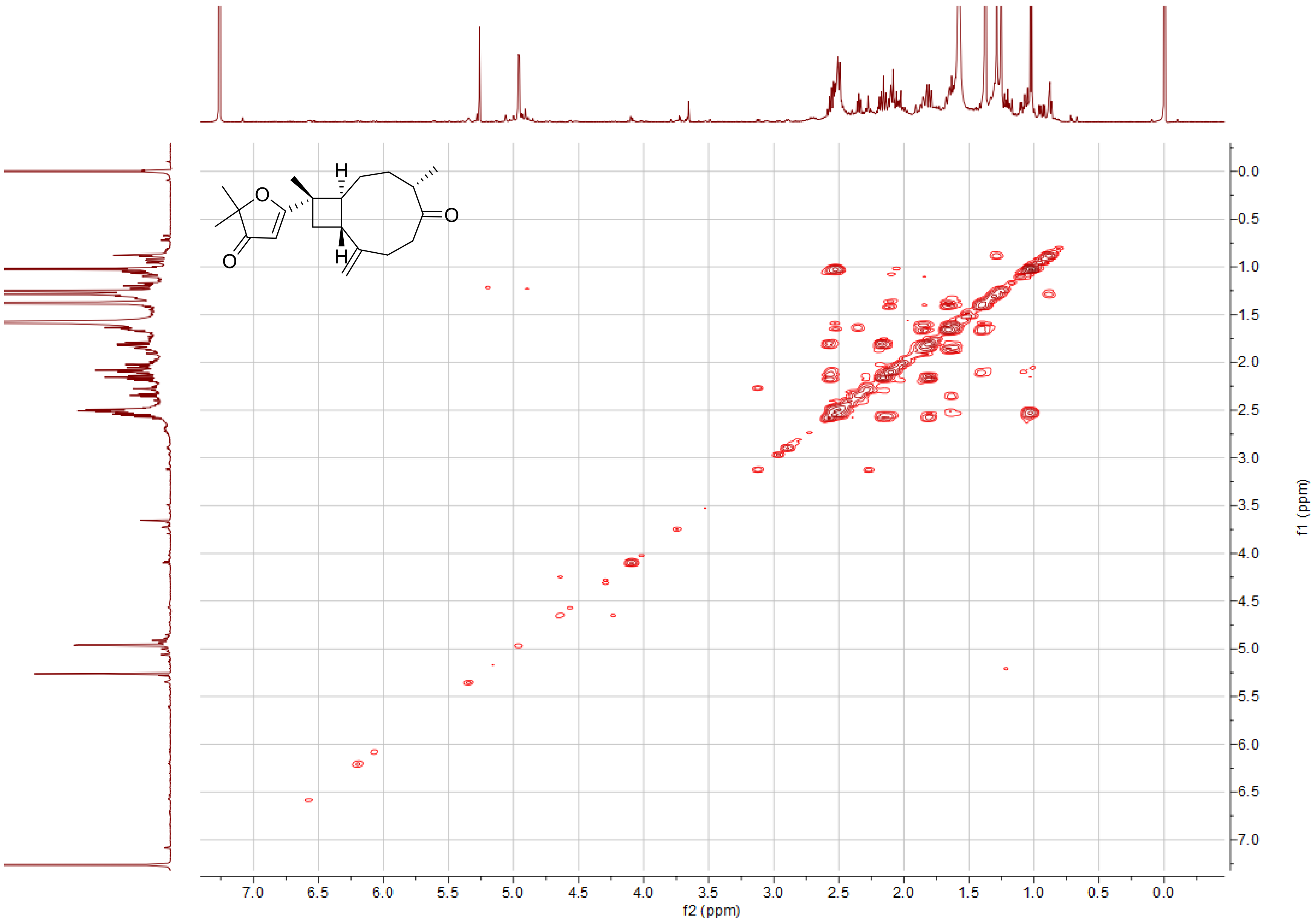

Figure S13. COSY spectrum (500 MHz) of sinuhirfuranone B (**2**) in $CDCl_3$.

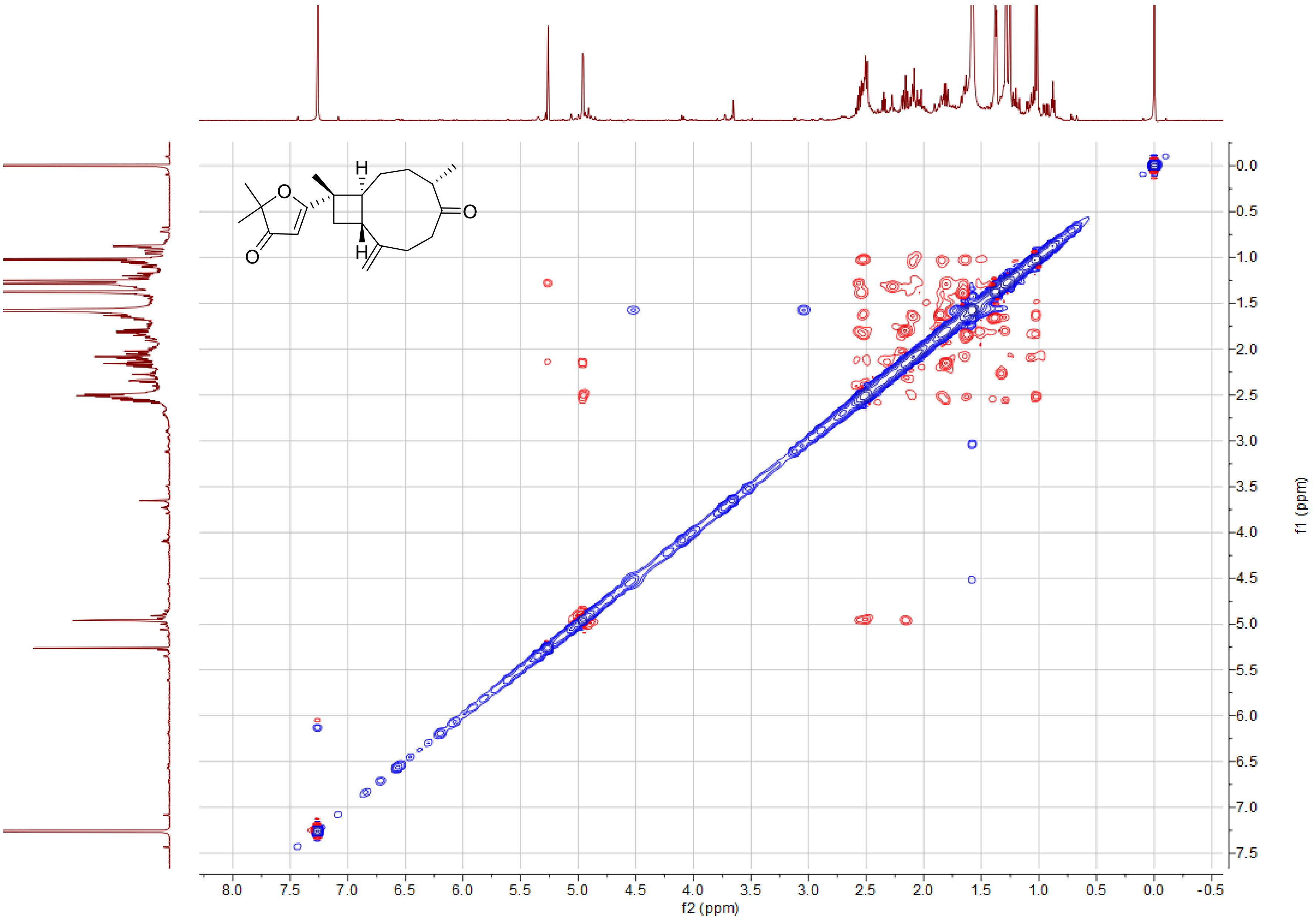

Figure S14. NOESY spectrum (500 MHz) of sinuhirfuranone B (**2**) in $CDCl_3$.

Fragmentor Voltage	Collision Energy	Ionization Mode
135	0	ESI

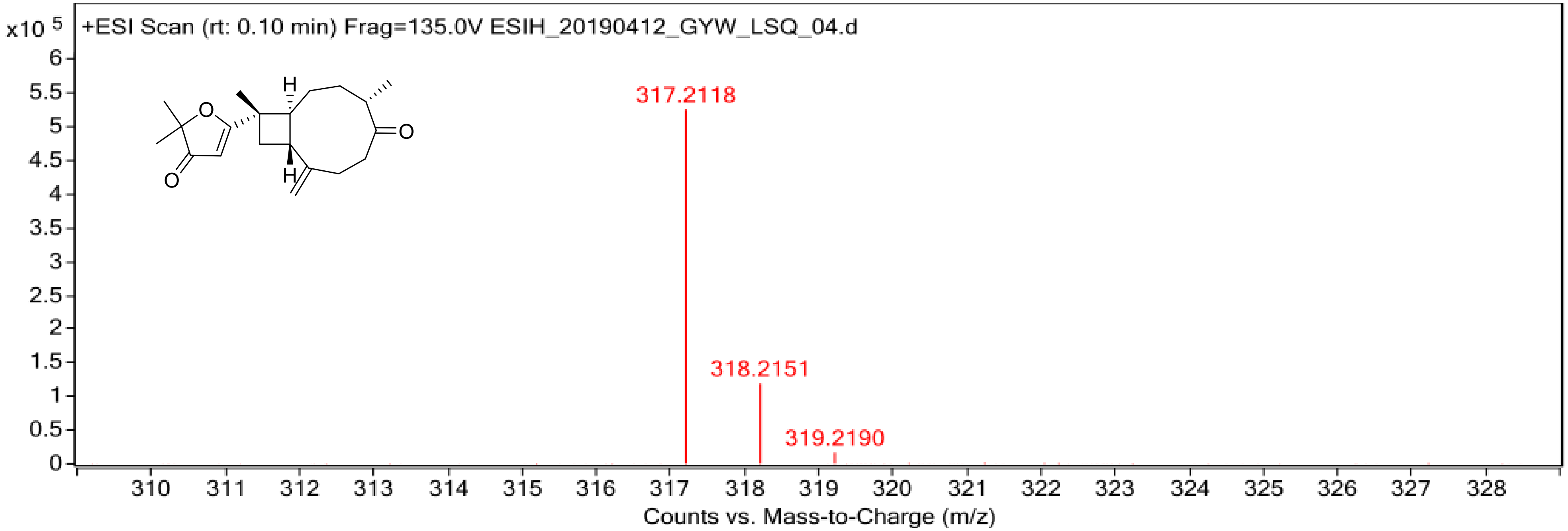

Formula Calculator Results

m/z	Calc m/z	Diff (mDa)	Diff (ppm)	Ion Formula	Ion
317.2118	317.2111	-0.71	-2.23	C20 H29 O3	(M+H)+

Figure S15. HRESIMS spectrum of sinuhirfuranone B (**2**) in MeOH.

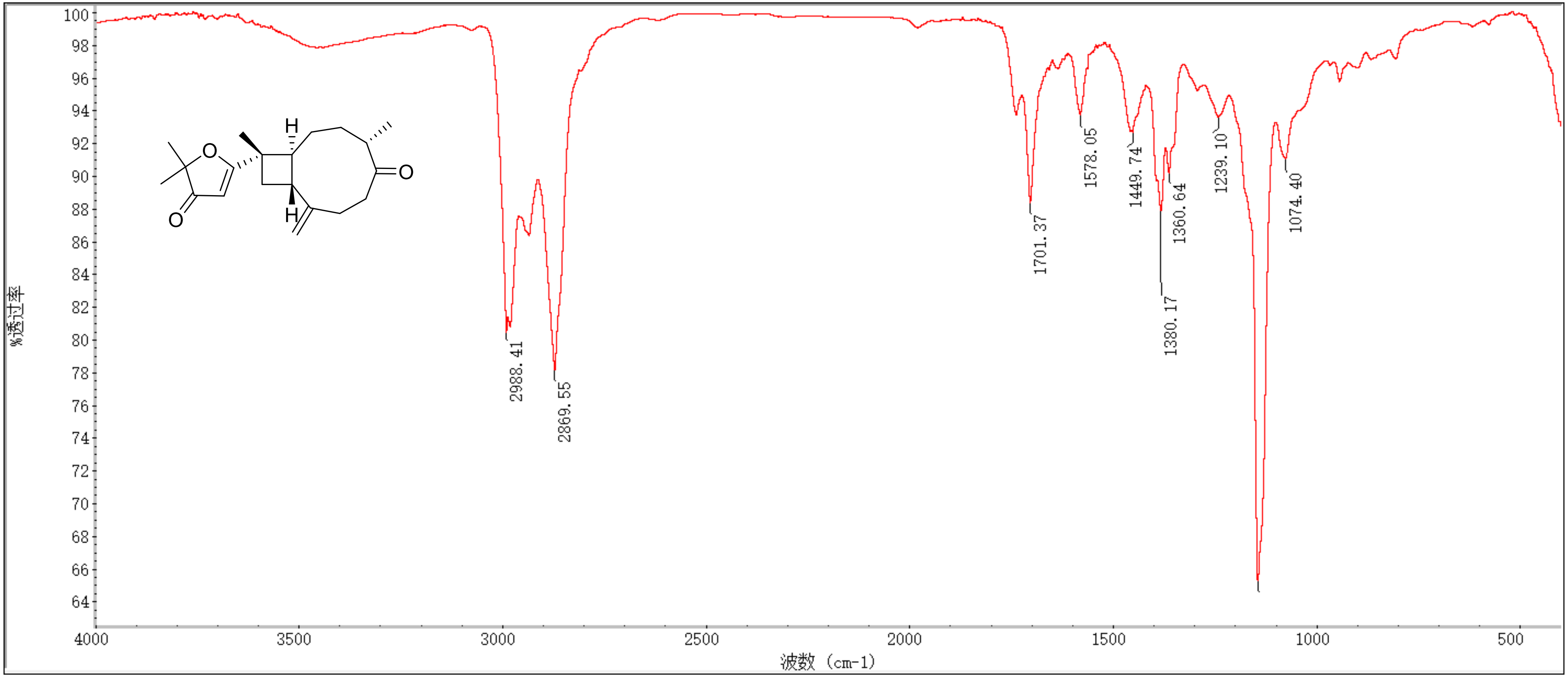

Figure S16. IR spectrum of sinuhirfuranone B (**2**).

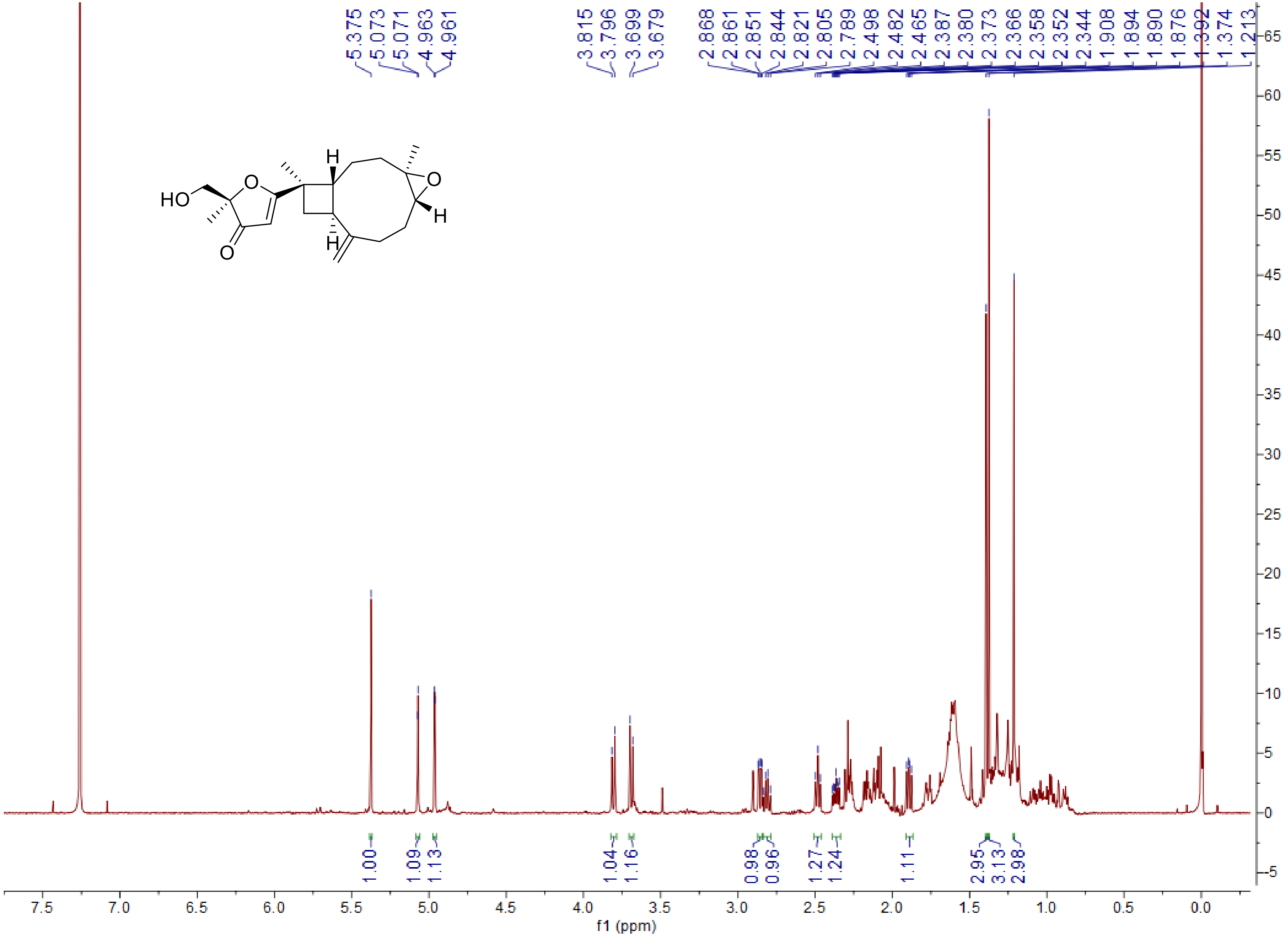

Figure S17. ^{1}H NMR spectrum (600 MHz) of sinuhirfuranone C (**3**) in $CDCl_3$.

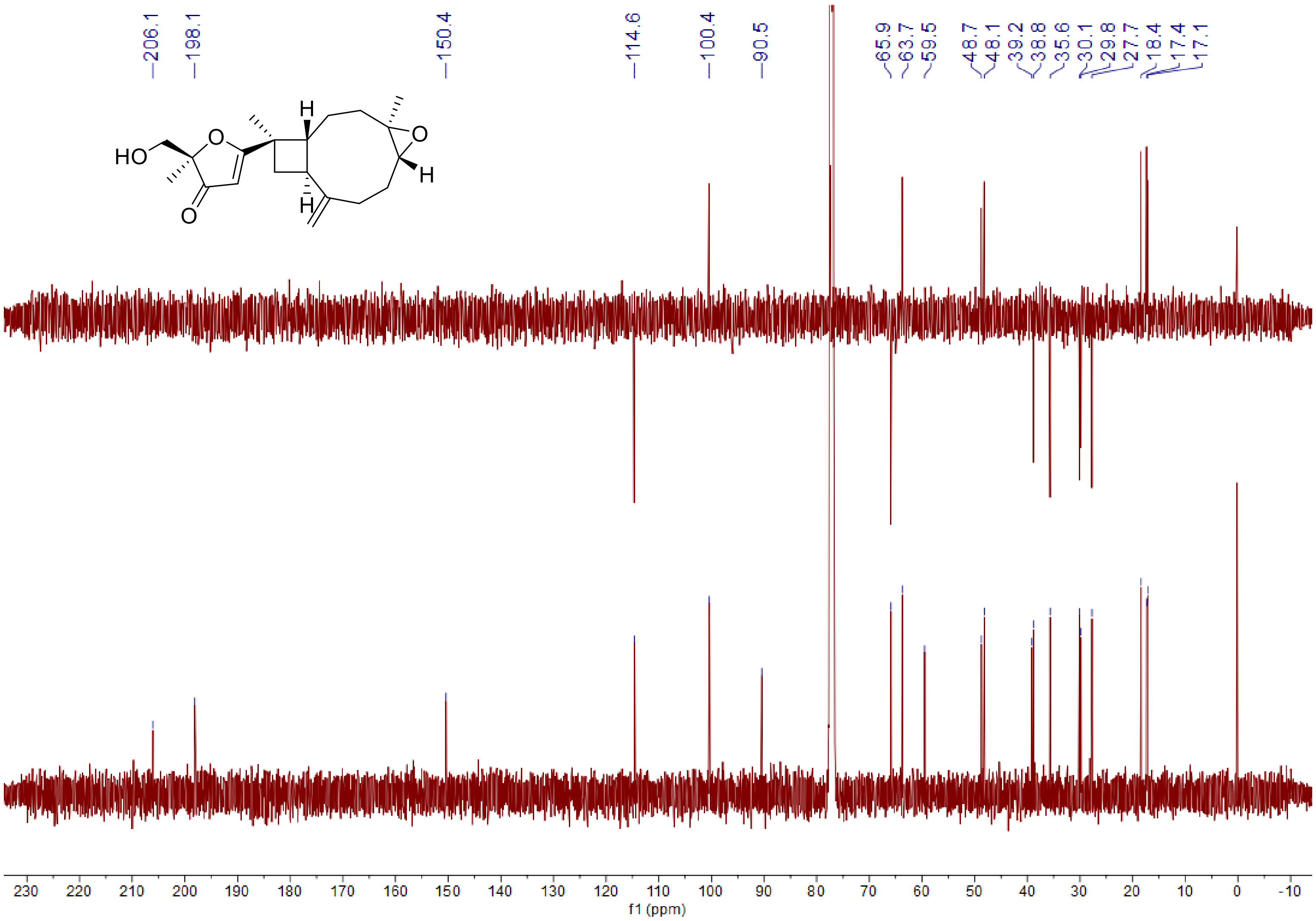

Figure S18. ^{13}C NMR spectrum (125 MHz) of sinuhirfuranone C (**3**) in $CDCl_3$.

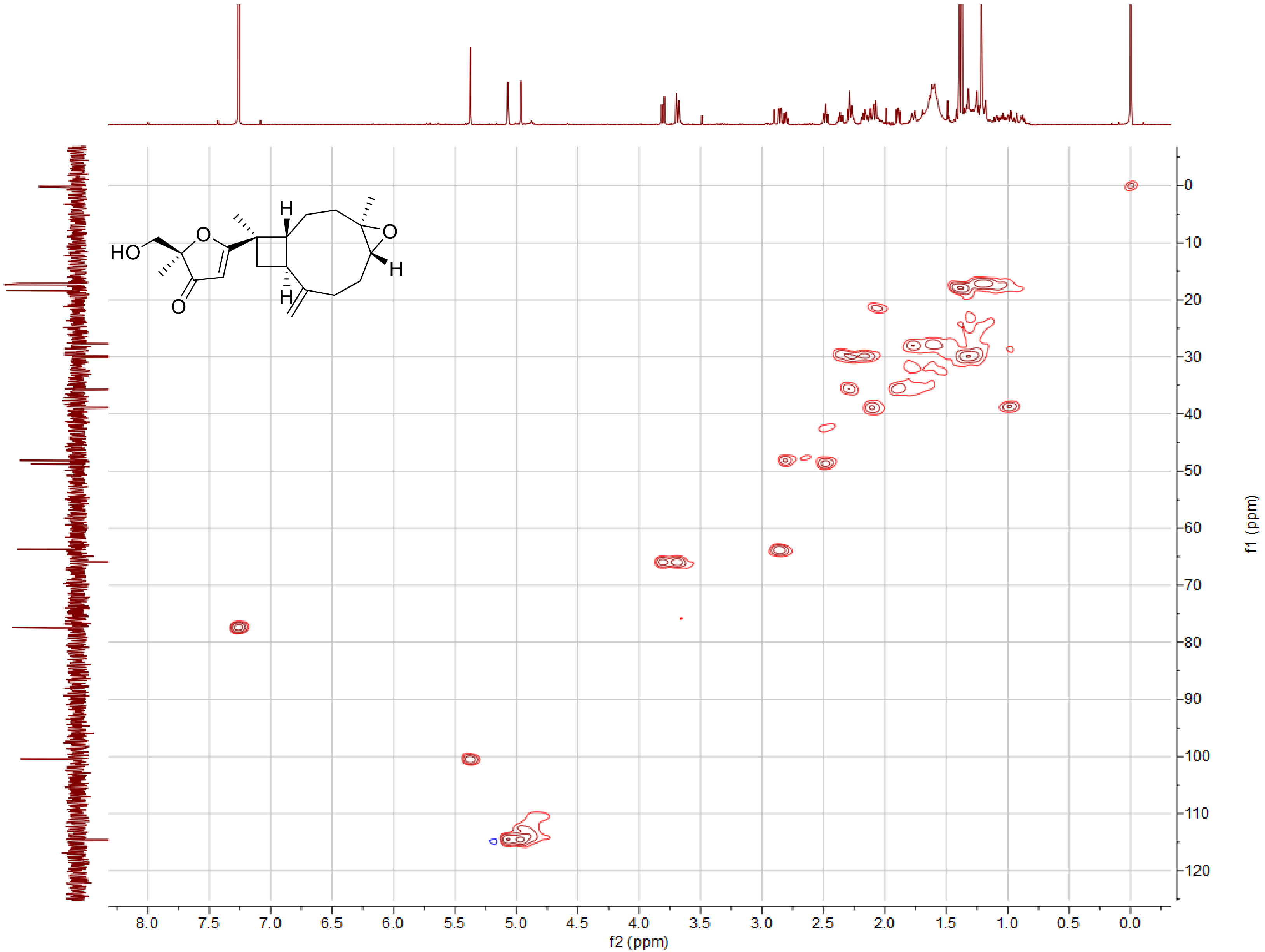

Figure S19. HSQC spectrum (500 MHz) of sinuhirfuranone C (**3**) in $CDCl_3$.

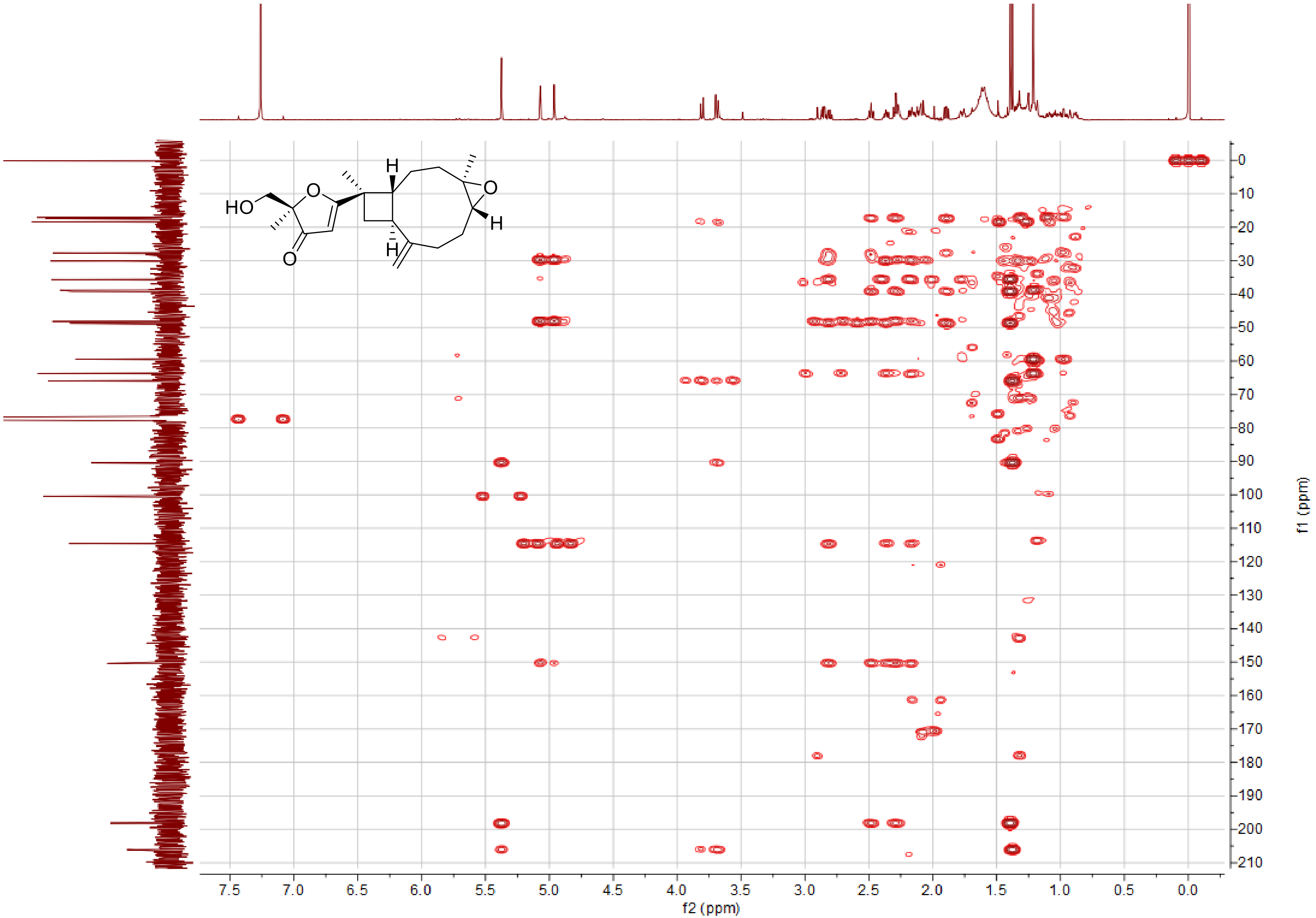

Figure S20. HMBC spectrum (500 MHz) of sinuhirfuranone C (**3**) in $CDCl_3$.

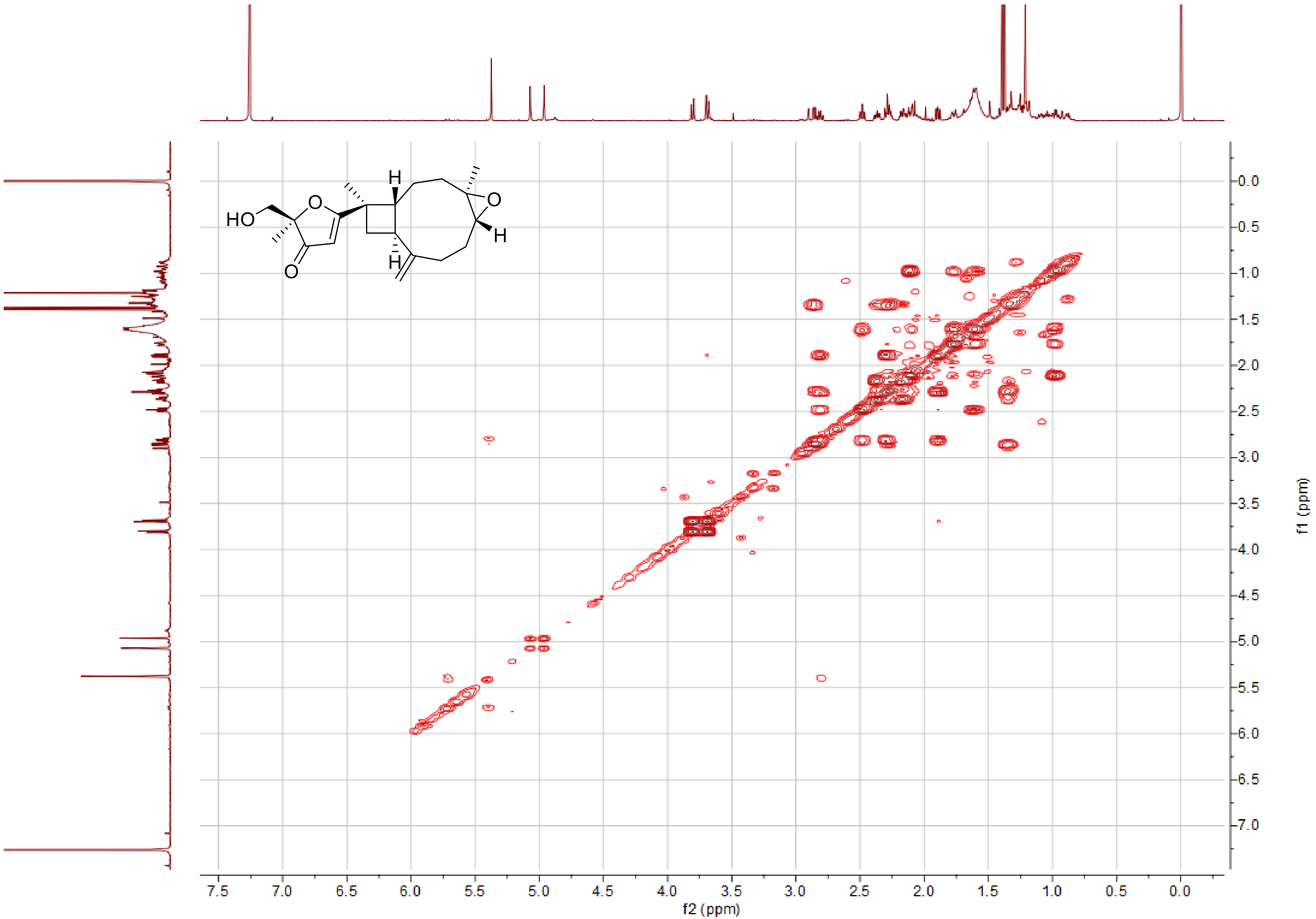

Figure S21. COSY spectrum (500 MHz) of sinuhirfuranone C (**3**) in $CDCl_3$.

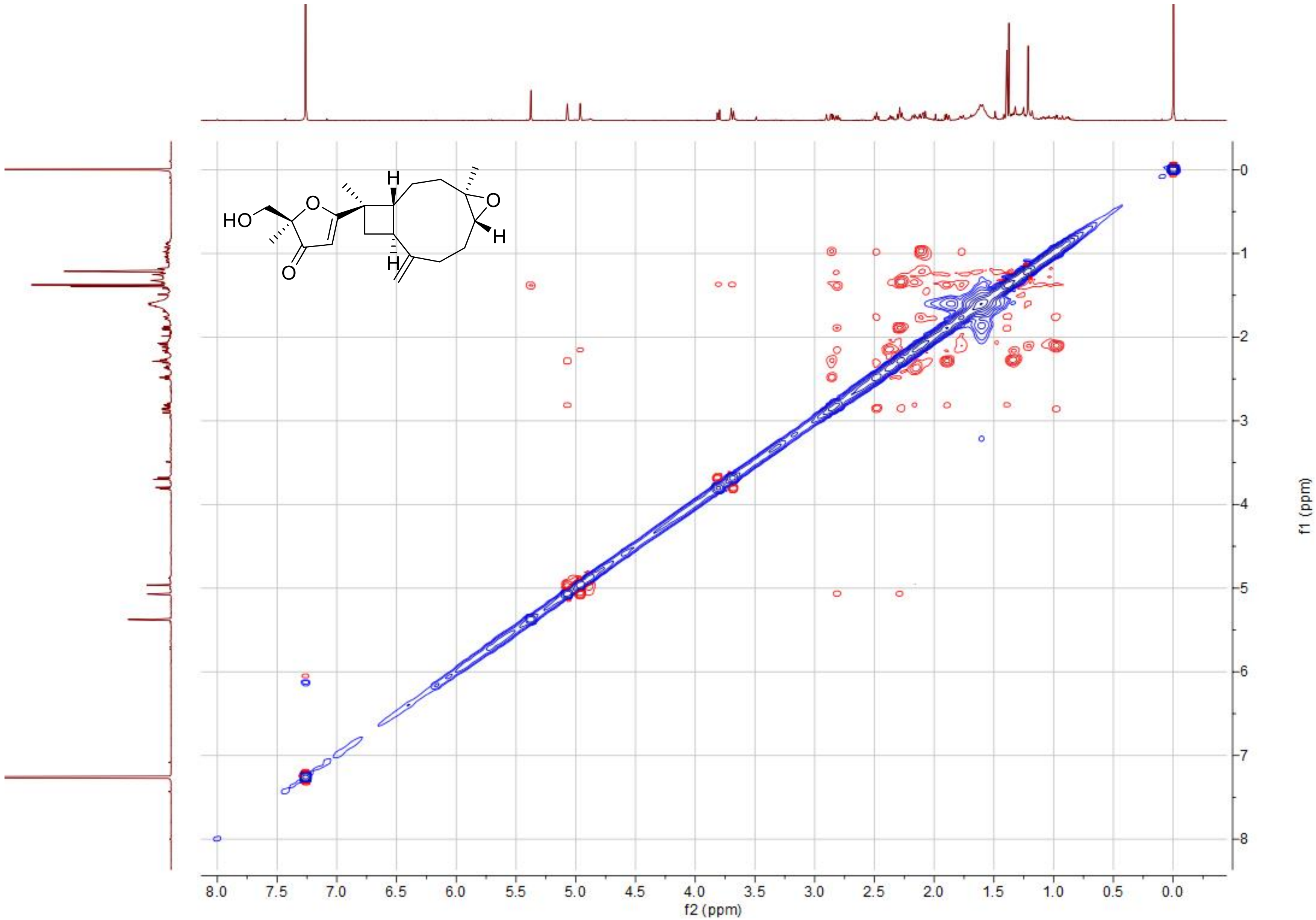

Figure S22. NOESY spectrum (500 MHz) of sinuhirfuranone C (**3**) in $CDCl_3$.

Fragmentor Voltage	Collision Energy	Ionization Mode
135	0	ESI

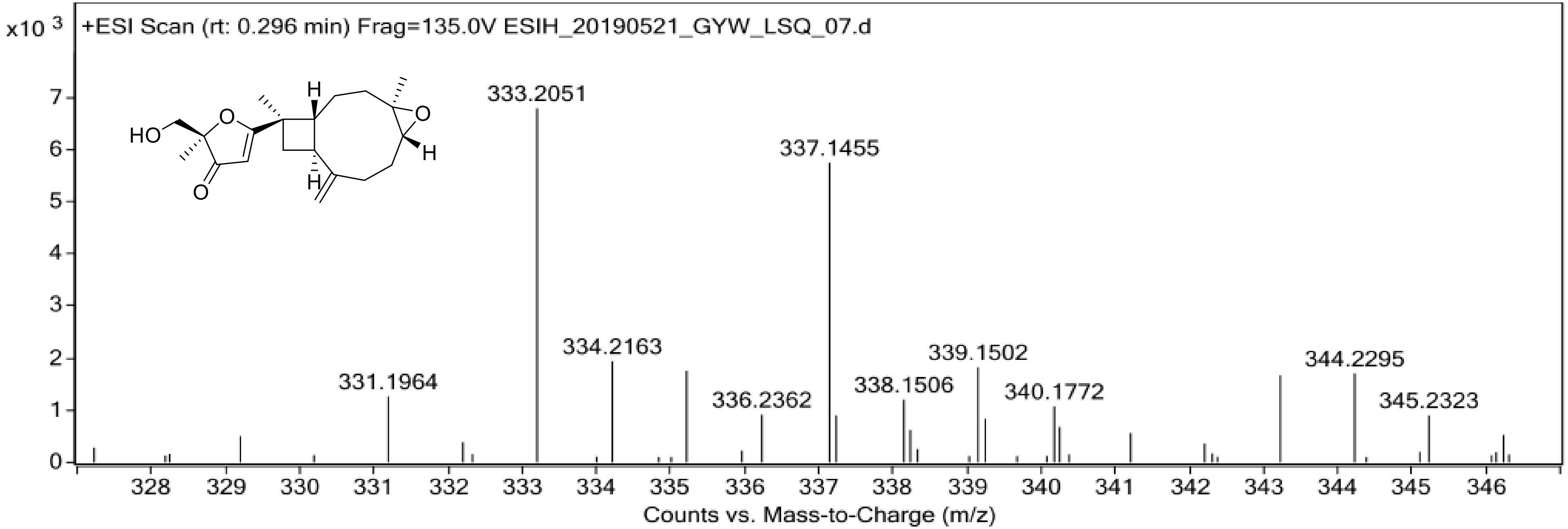

Formula Calculator Results

m/z	Calc m/z	Diff (mDa)	Diff (ppm)	Ion Formula	Ion
333.2051	333.206	0.92	2.78	C20 H29 O4	(M+H)+

Figure S23. HRESIMS spectrum of sinuhirfuranone C (**3**) in MeOH.

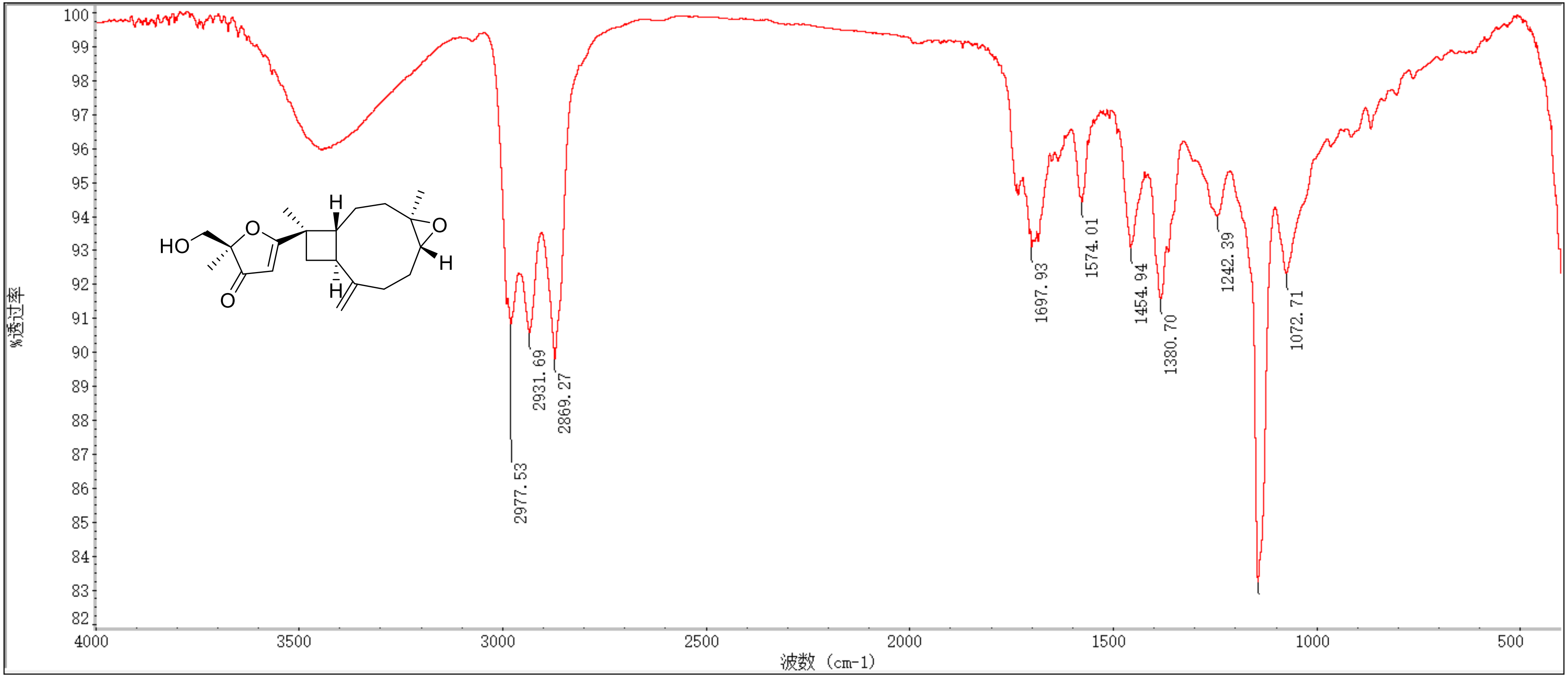

Figure S24. IR spectrum of sinuhirfuranone C (**3**).

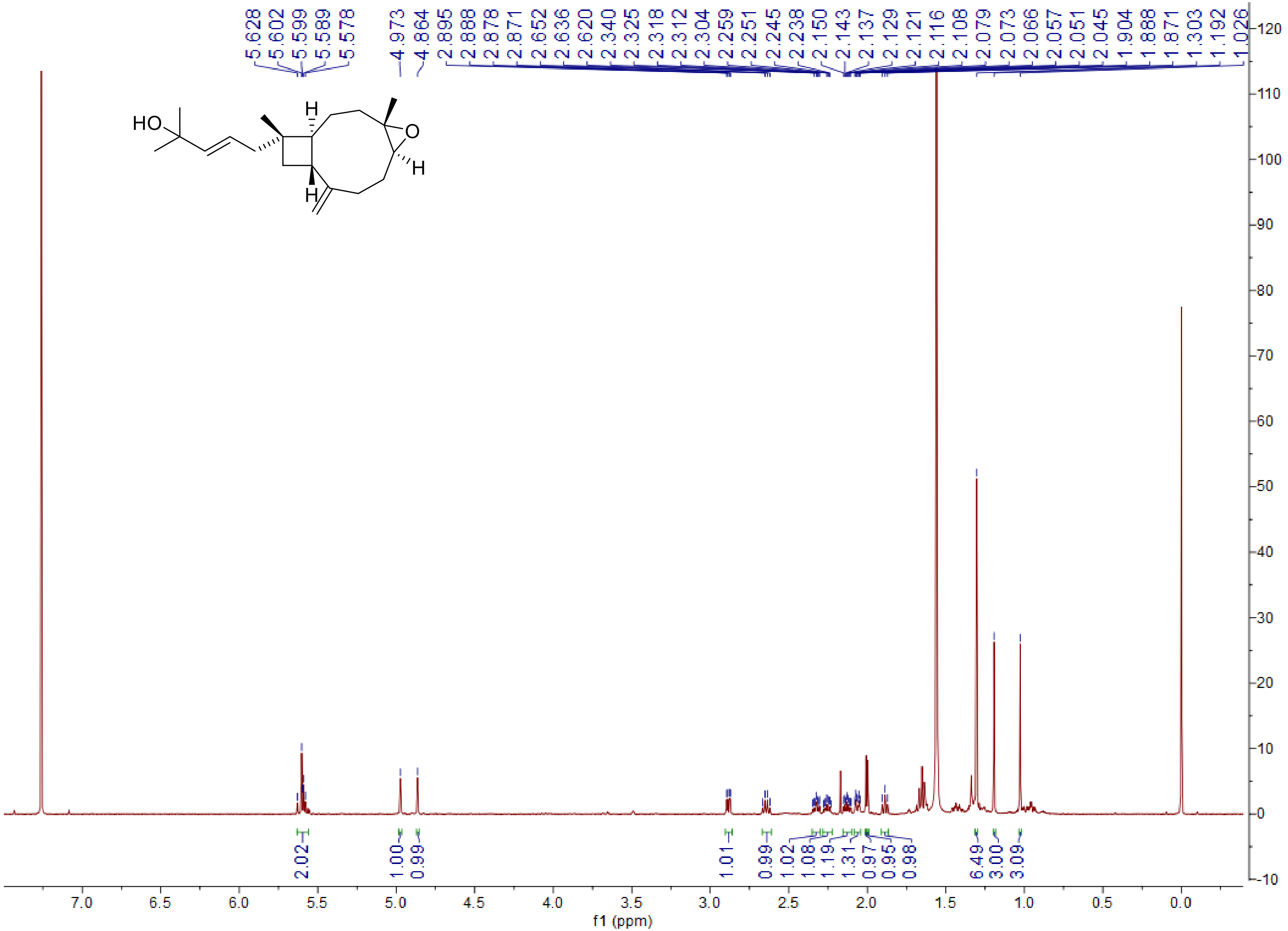

Figure S25. ^{1}H NMR spectrum (600 MHz) of sinuhirtin C (**4**) in $CDCl_3$.

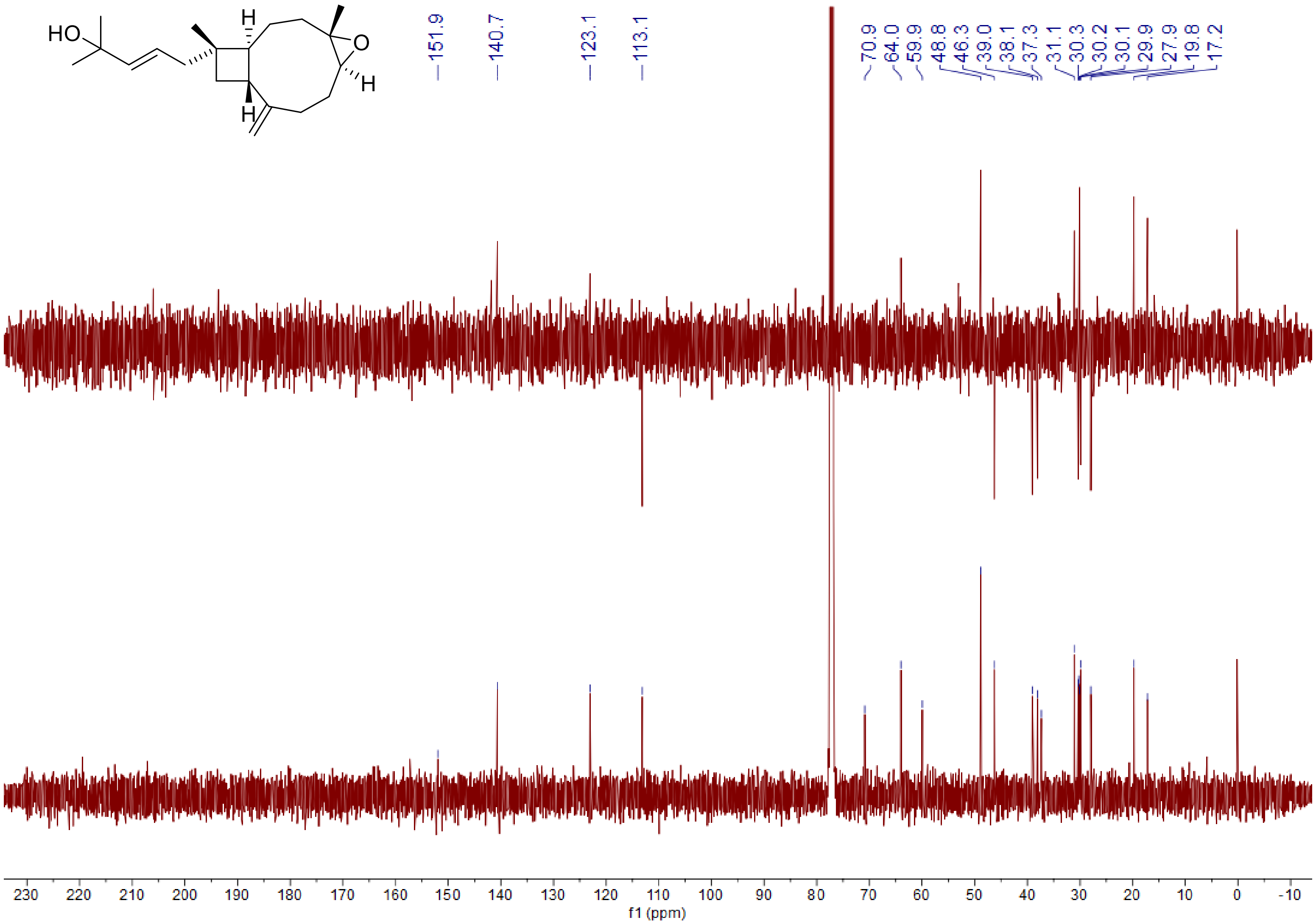

Figure S26. ^{13}C NMR spectrum (125 MHz) of sinuhirtin C (**4**) in $CDCl_3$.

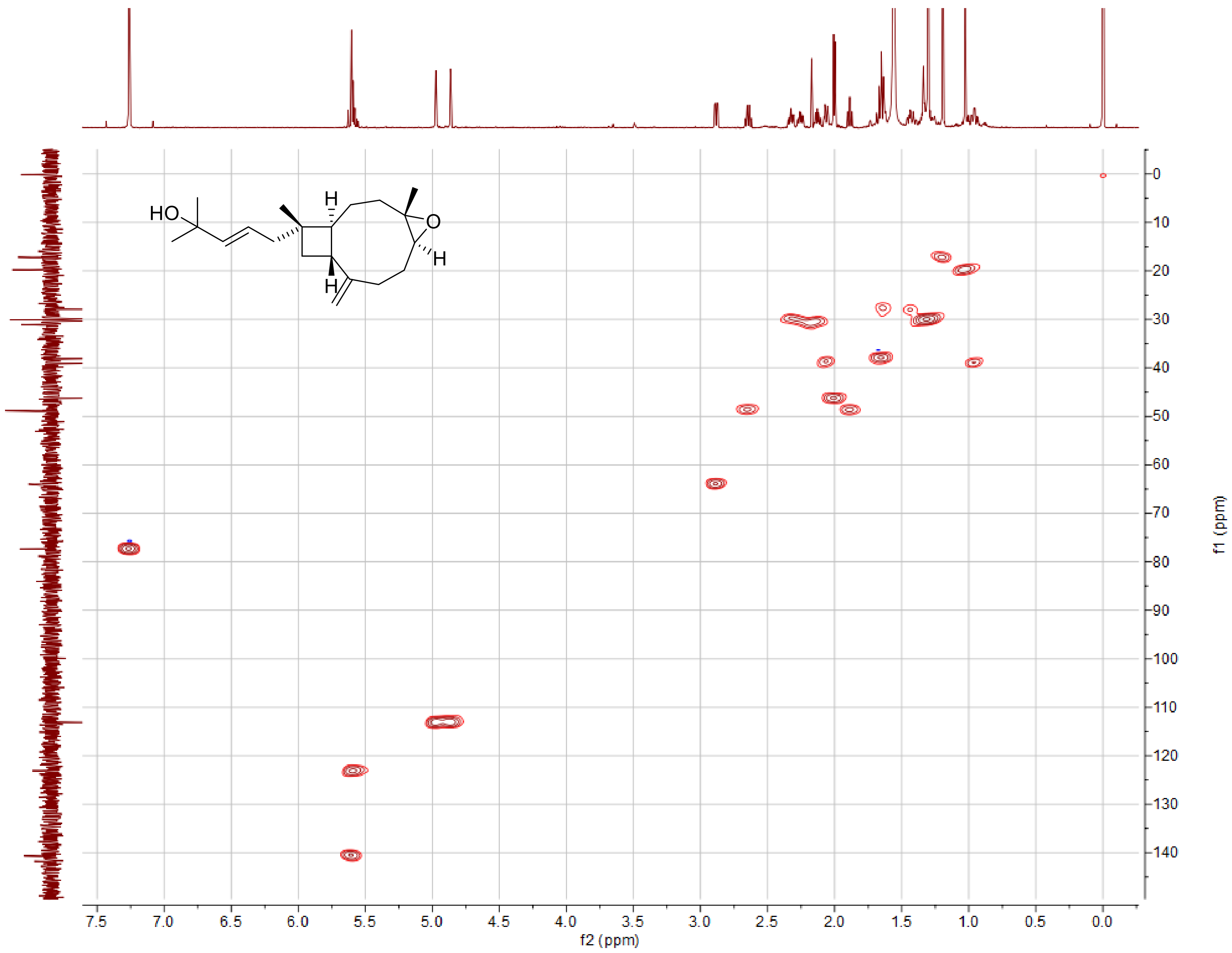

Figure S27. HSQC spectrum (500 MHz) of sinuhirtin C (**4**) in $CDCl_3$.

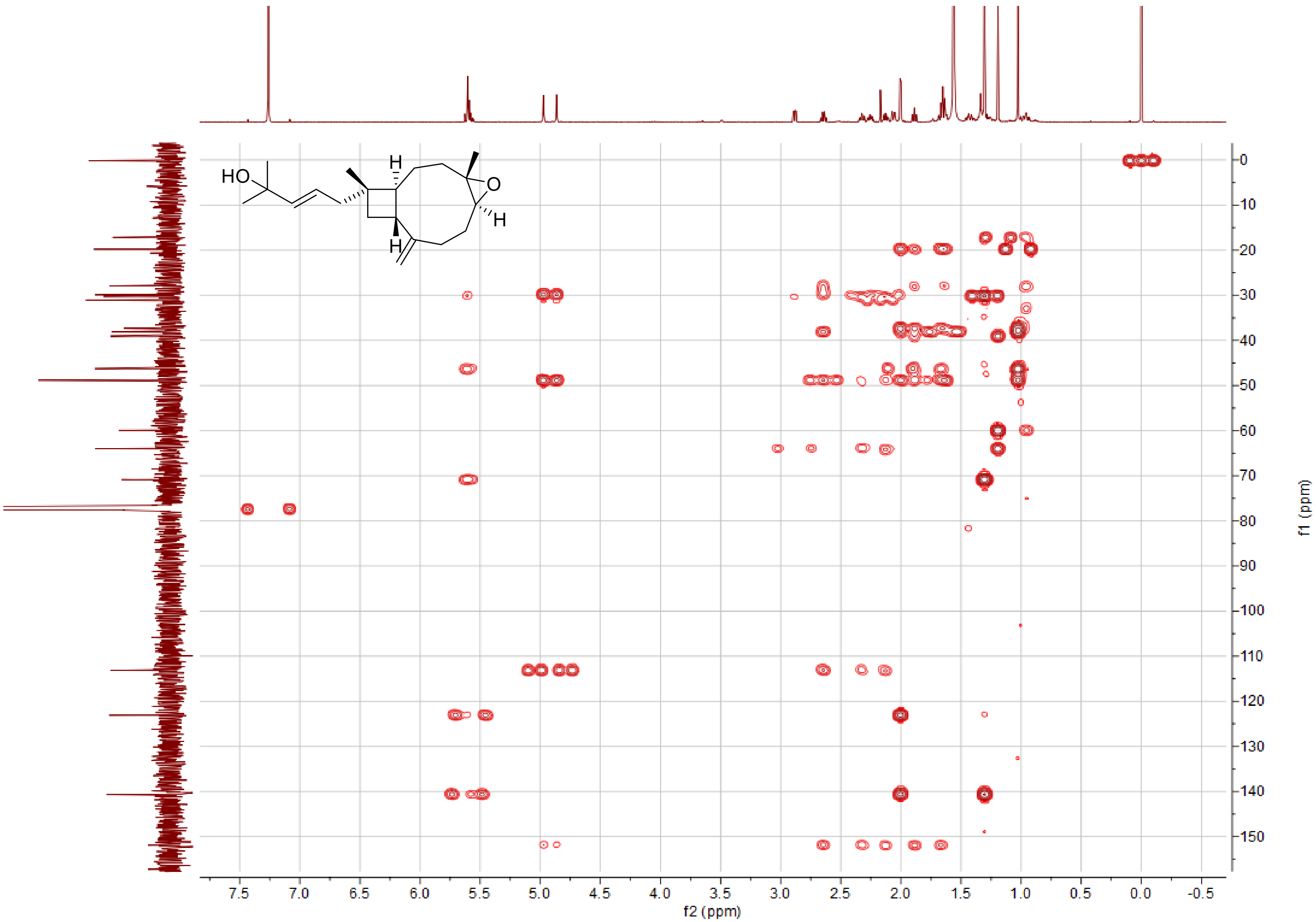

Figure S28. HMBC spectrum (500 MHz) of sinuhirtin C (**4**) in $CDCl_3$.

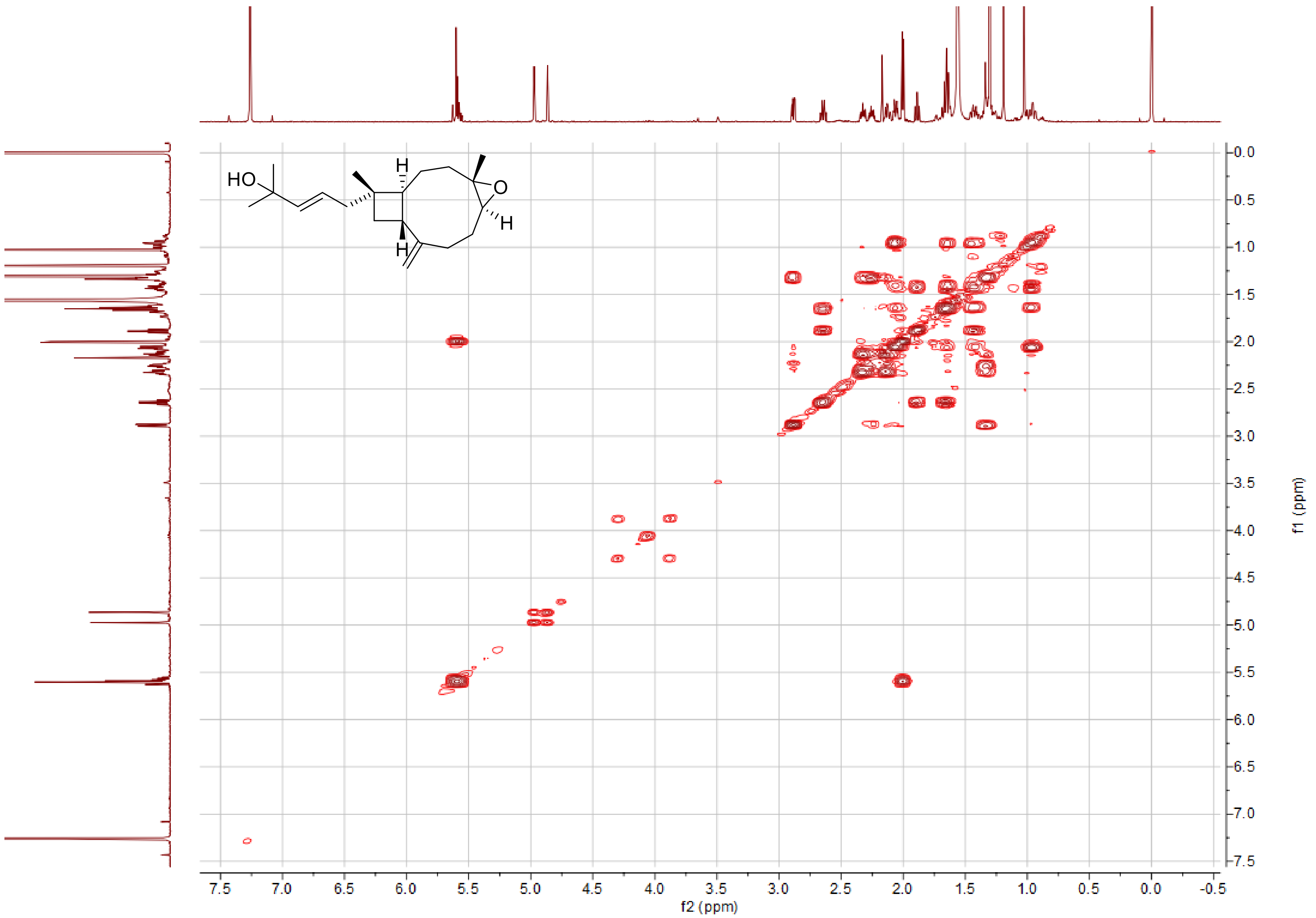

Figure S29. COSY spectrum (500 MHz) of sinuhirtin C (**4**) in $CDCl_3$.

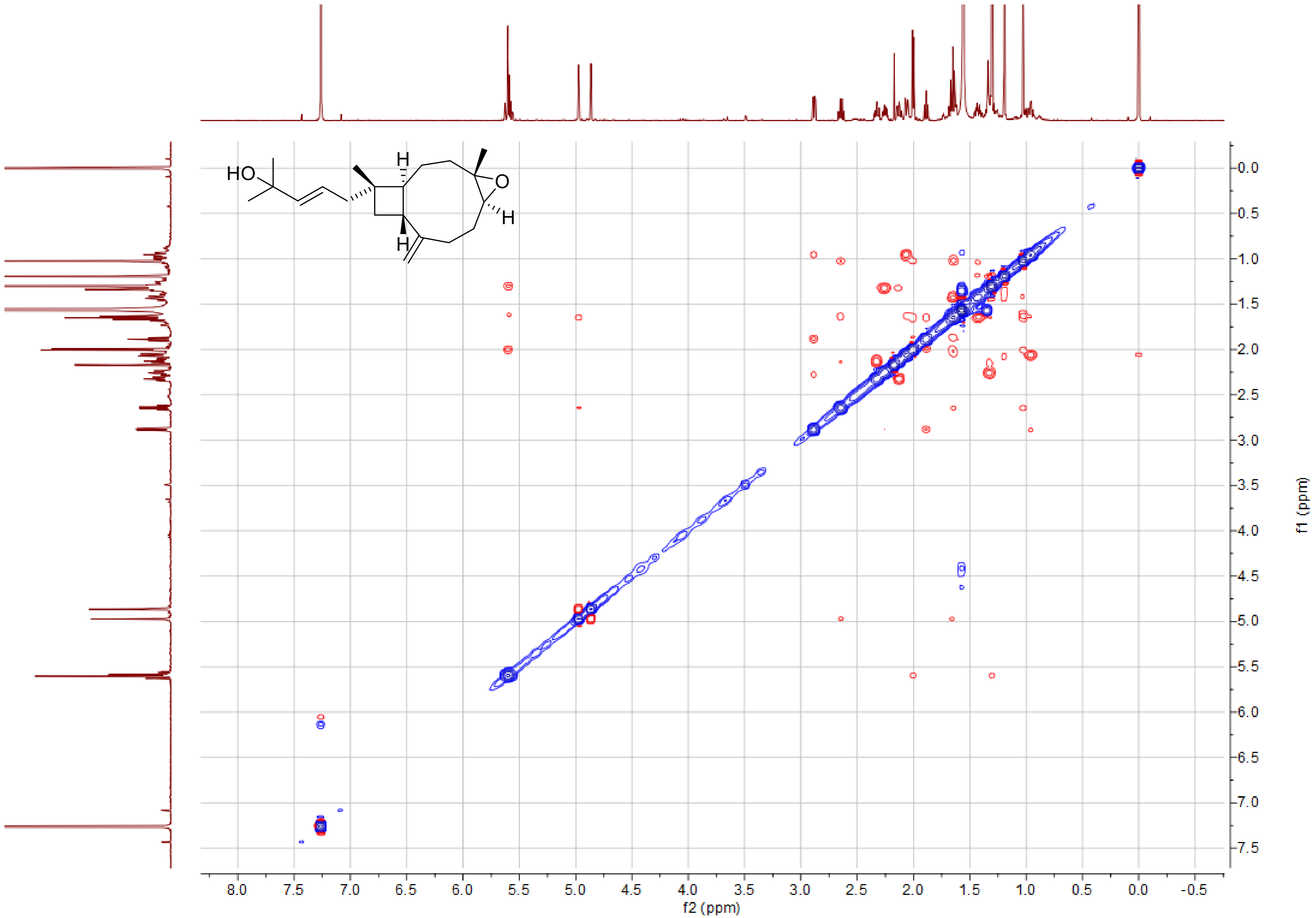

Figure S30. NOESY spectrum (500 MHz) of sinuhirtin C (**4**) in $CDCl_3$.

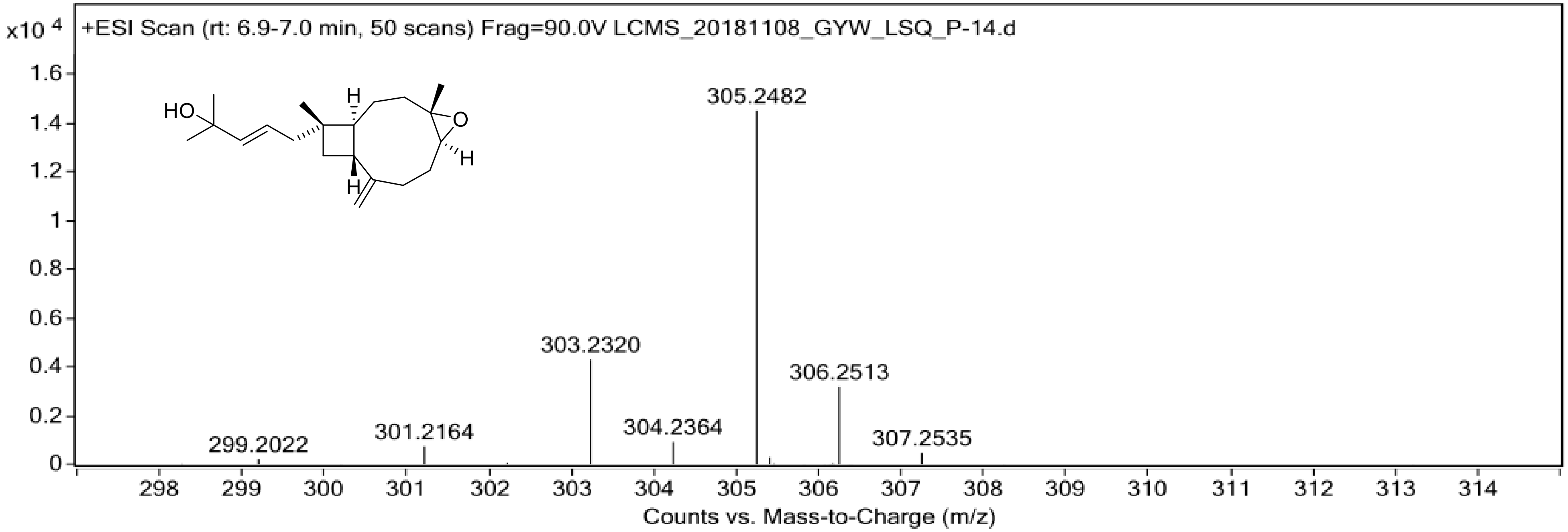

Formula Calculator Results

m/z	Calc m/z	Diff (mDa)	Diff (ppm)	Ion Formula	Ion
305.2482	305.2475	-0.65	-2.14	C20 H33 O2	(M+H)+

Figure S31. HRESIMS spectrum of sinuhirtin C (**4**) in MeOH.

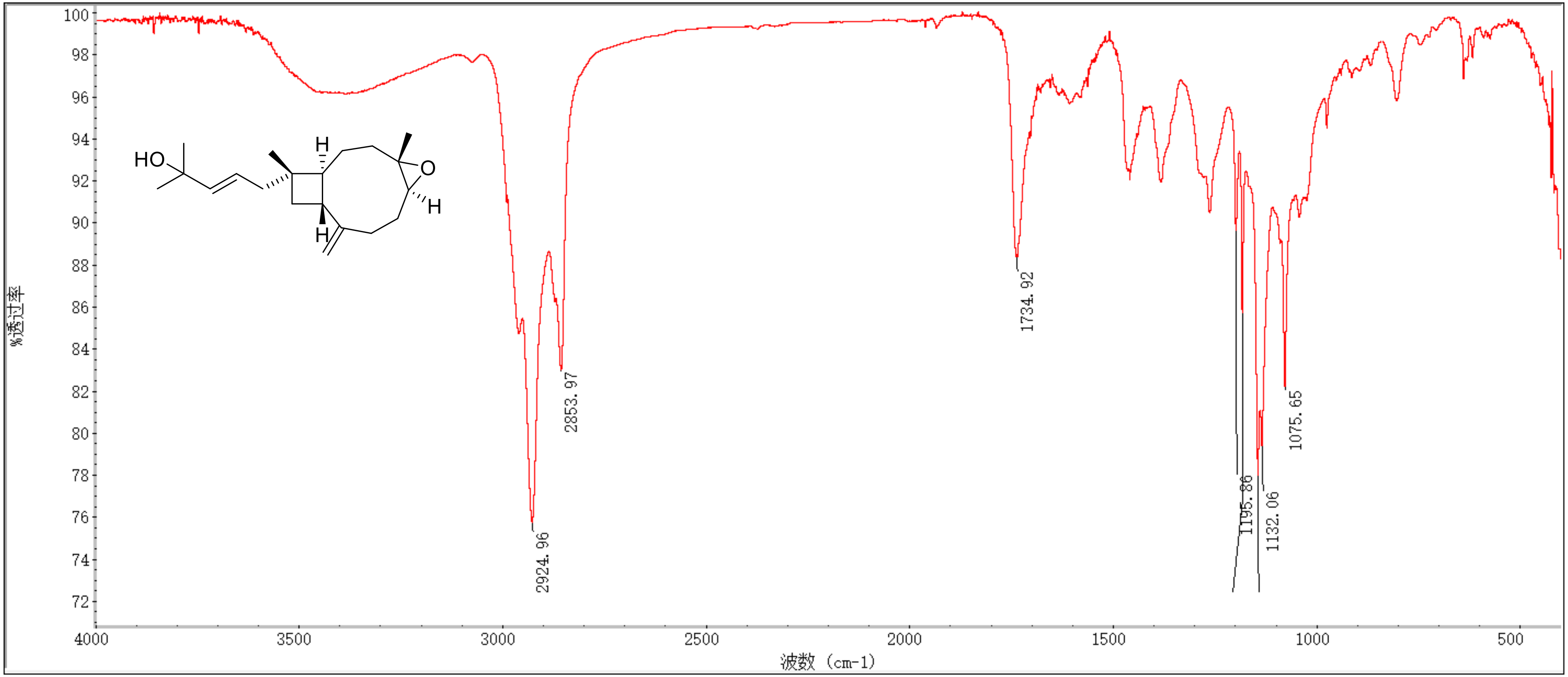

Figure S32. IR spectrum of sinuhirtin C (**4**).

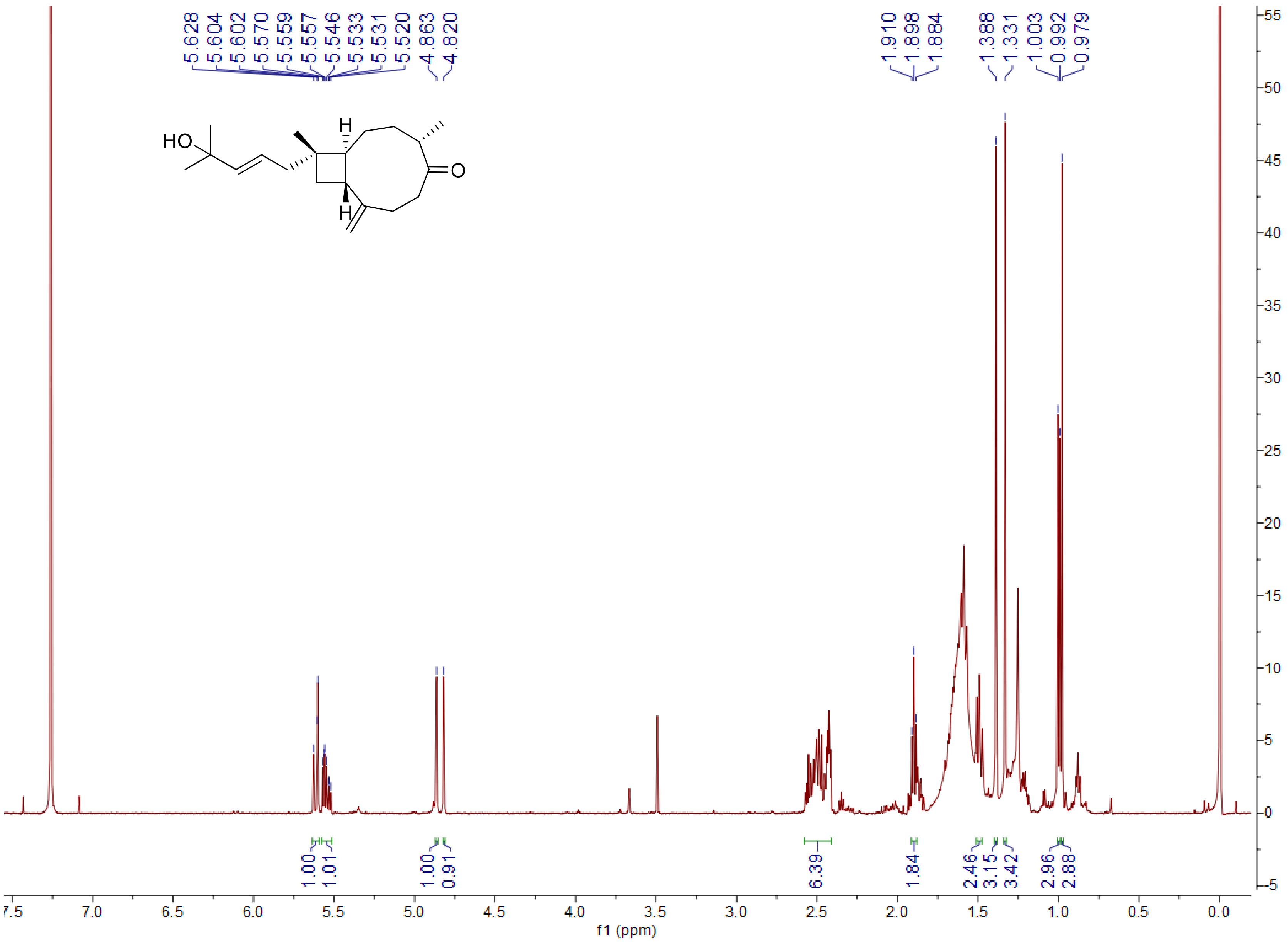

Figure S33. ^{1}H NMR spectrum (600 MHz) of sinuhirtin D (**5**) in $CDCl_3$.

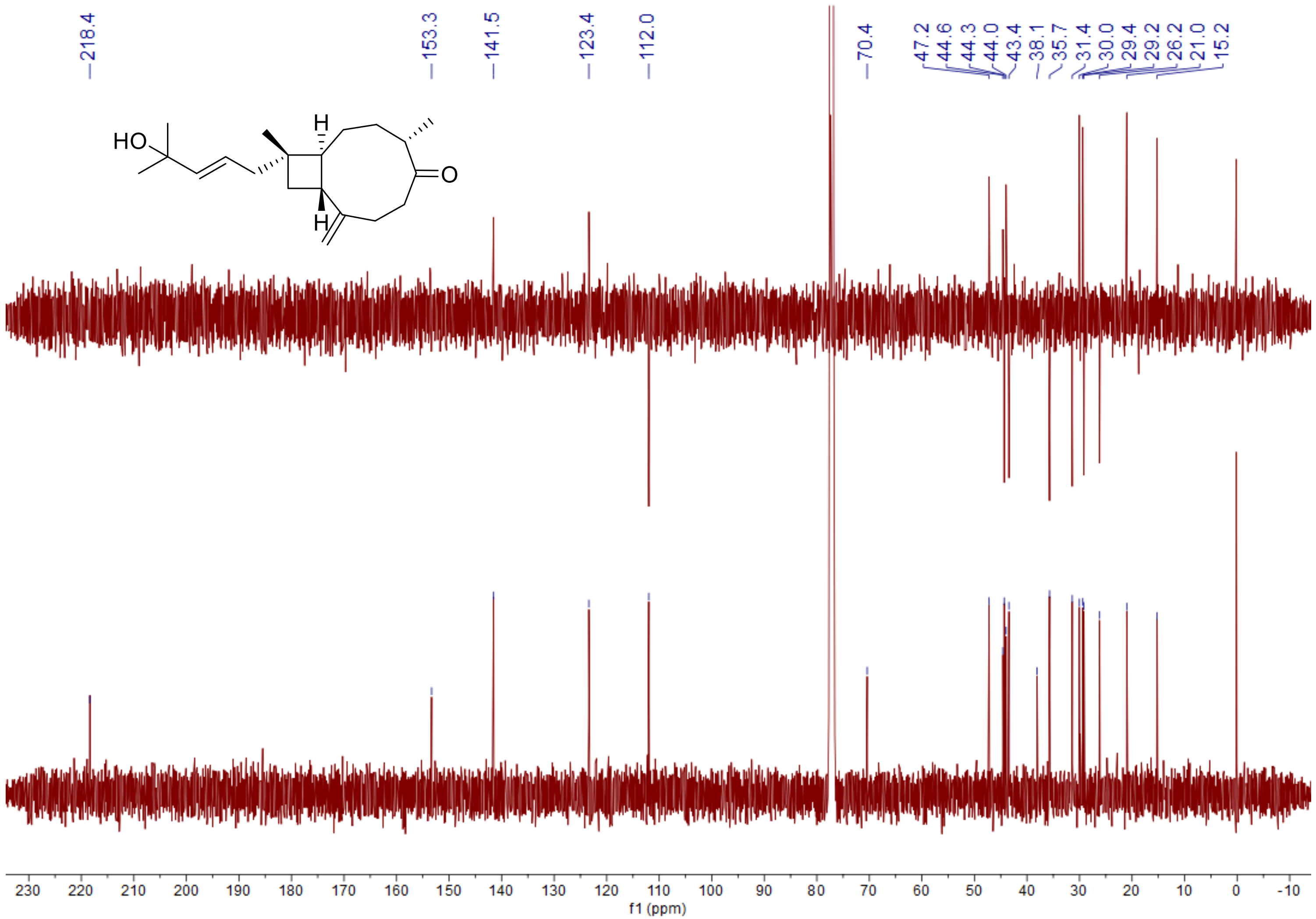

Figure S34. ^{13}C NMR spectrum (125 MHz) of sinuhirtin D (**5**) in $CDCl_3$.

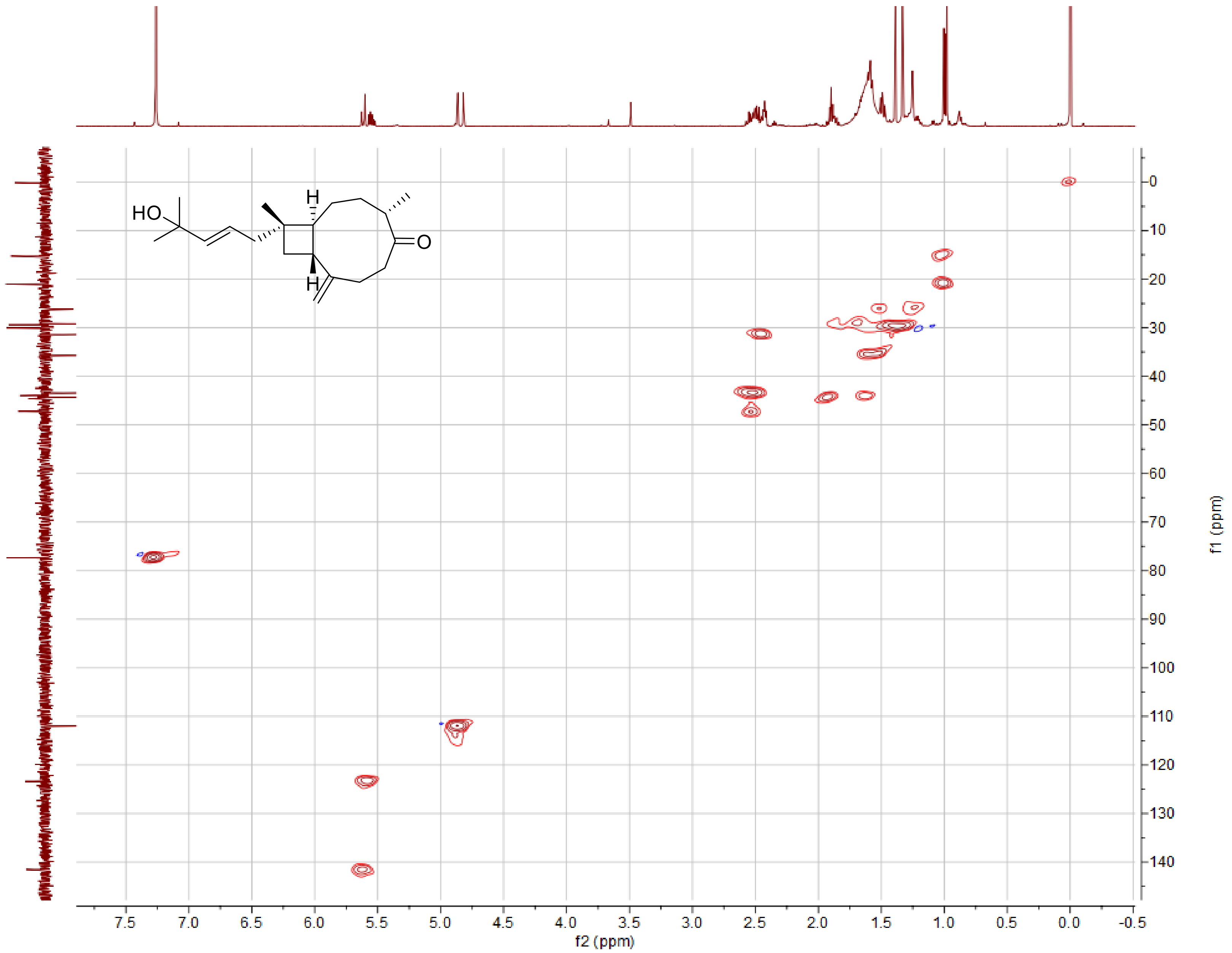

Figure S35. HSQC spectrum (500 MHz) of sinuhirtin D (**5**) in $CDCl_3$.

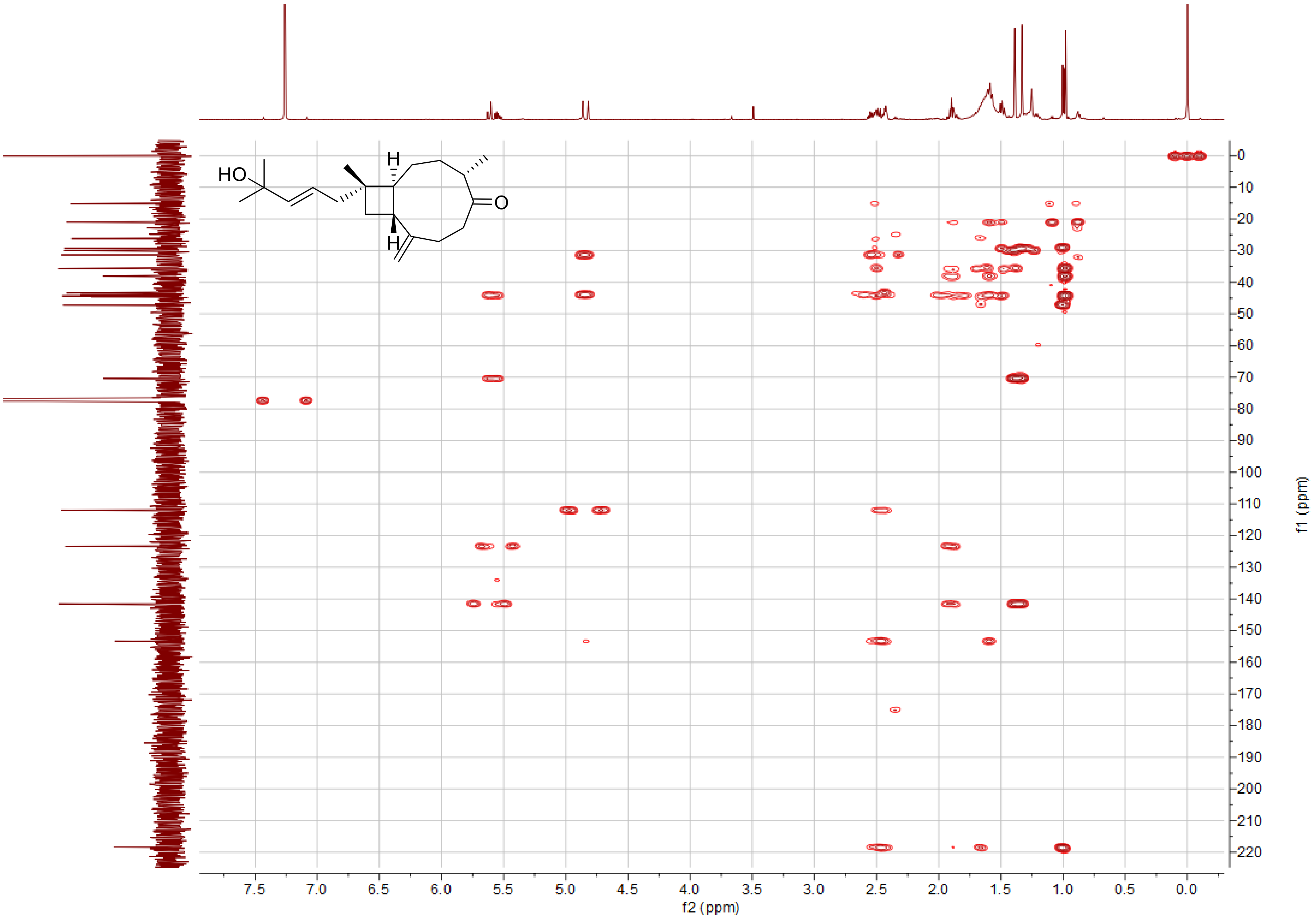

Figure S36. HMBC spectrum (500 MHz) of sinuhirtin D (**5**) in $CDCl_3$.

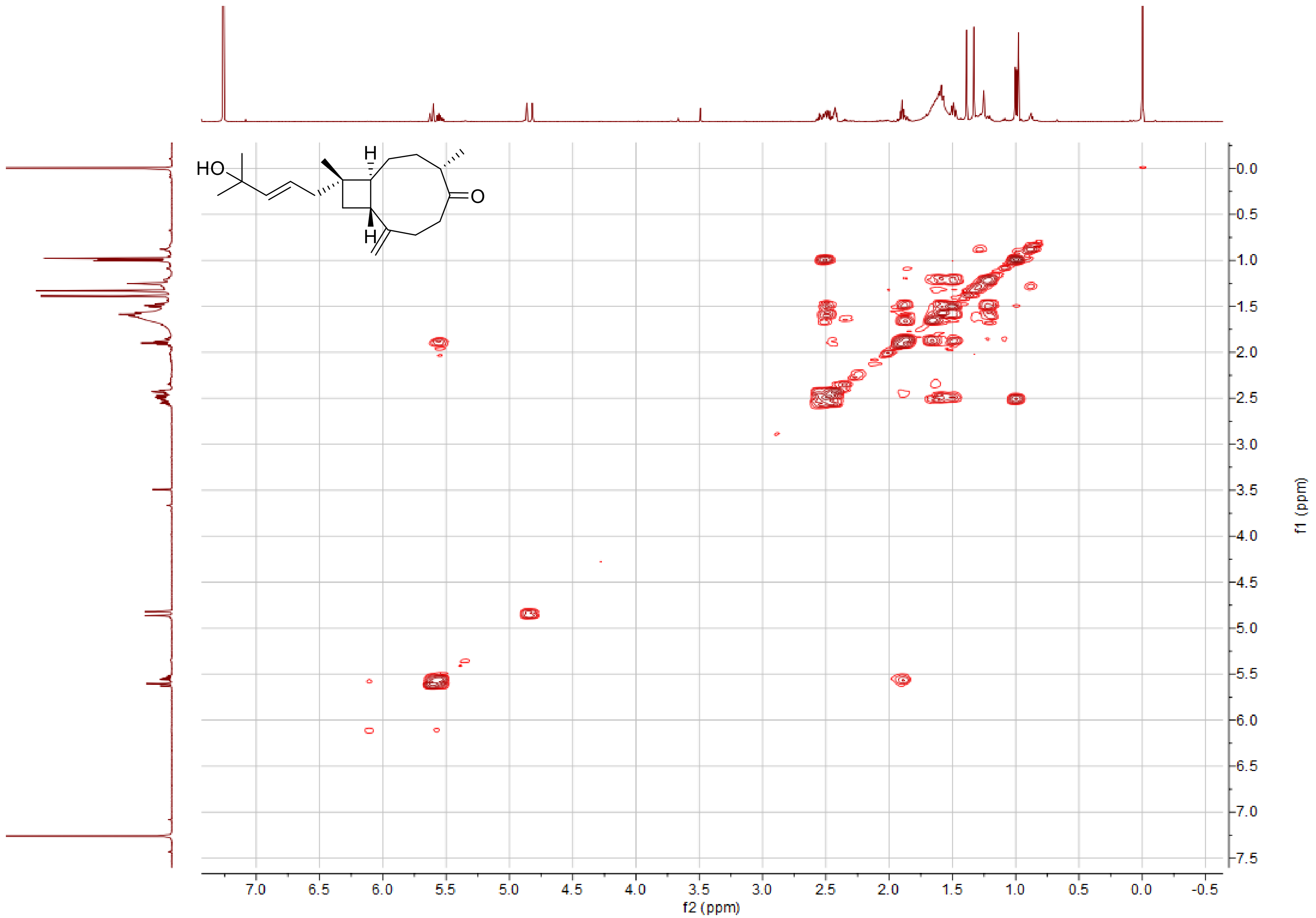

Figure S37. COSY spectrum (500 MHz) of sinuhirtin D (**5**) in $CDCl_3$.

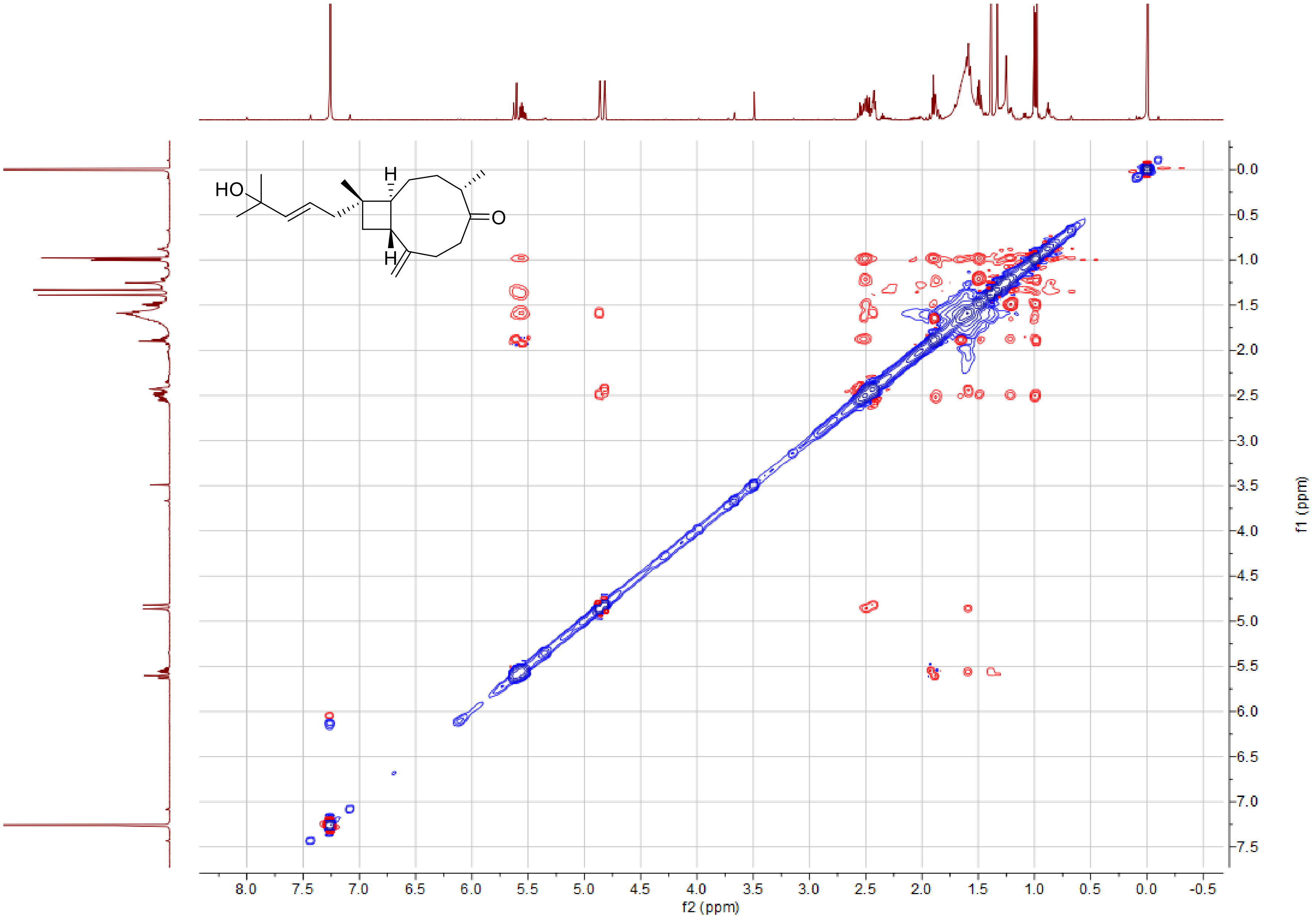

Figure S38. NOESY spectrum (500 MHz) of sinuhirtin D (**5**) in $CDCl_3$.

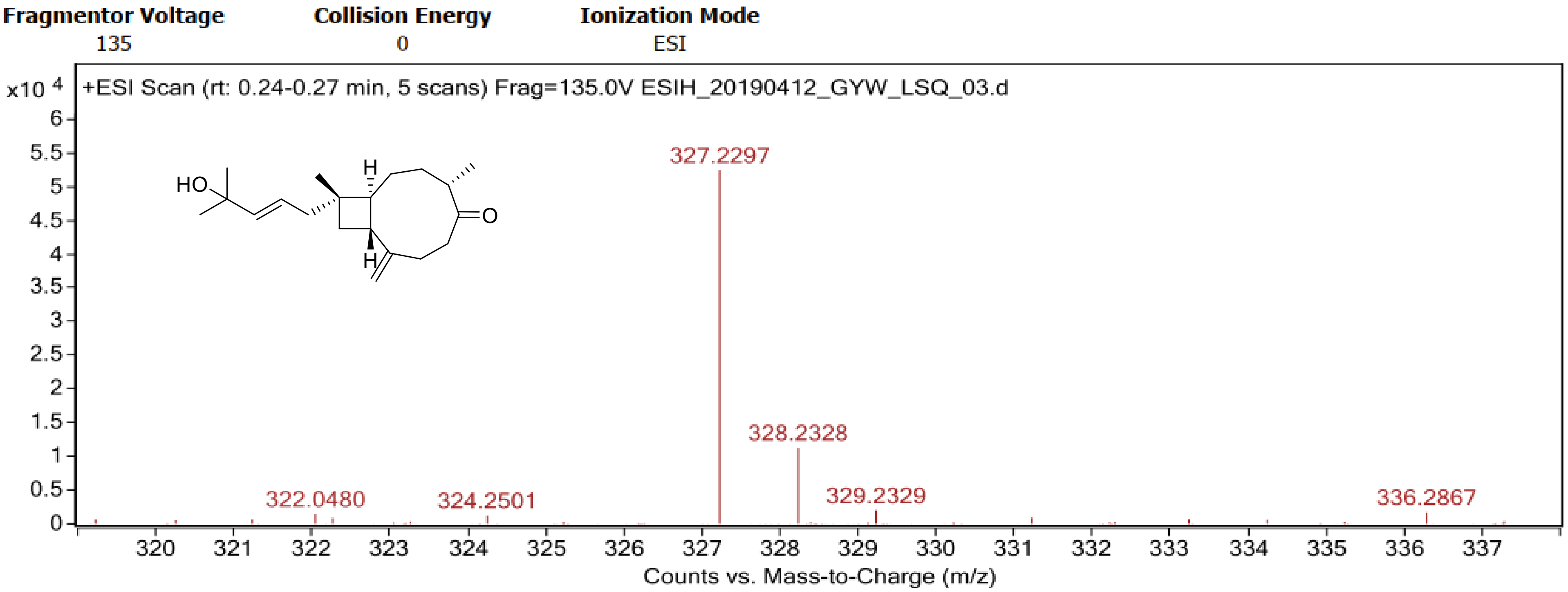

Formula Calculator Results

m/z	Calc m/z	Diff (mDa)	Diff (ppm)	Ion Formula	Ion
327.2297	327.2295	-0.21	-0.63	C20 H32 Na O2	(M+Na)+

Figure S39. HRESIMS spectrum of sinuhirtin D (**5**) in MeOH.

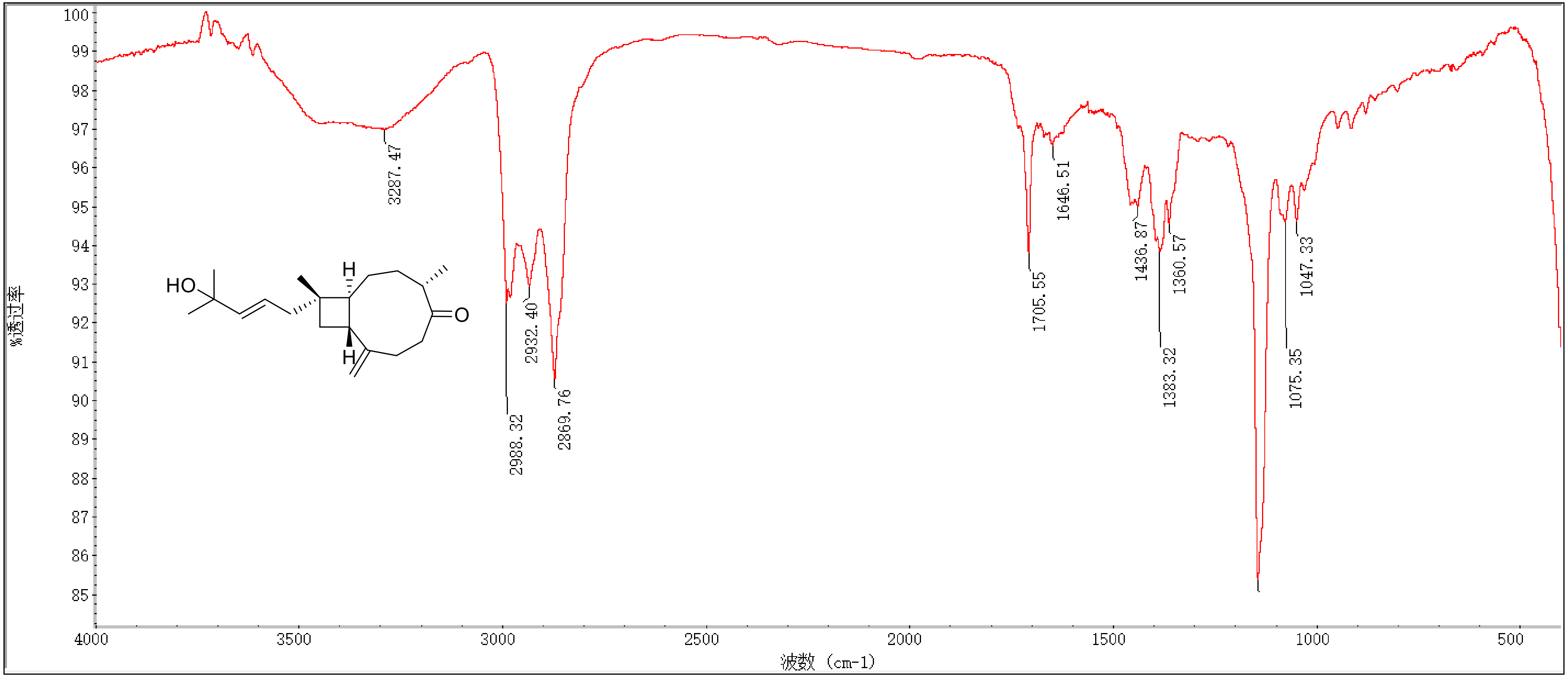

Figure S40. IR spectrum of sinuhirtin D (**5**).

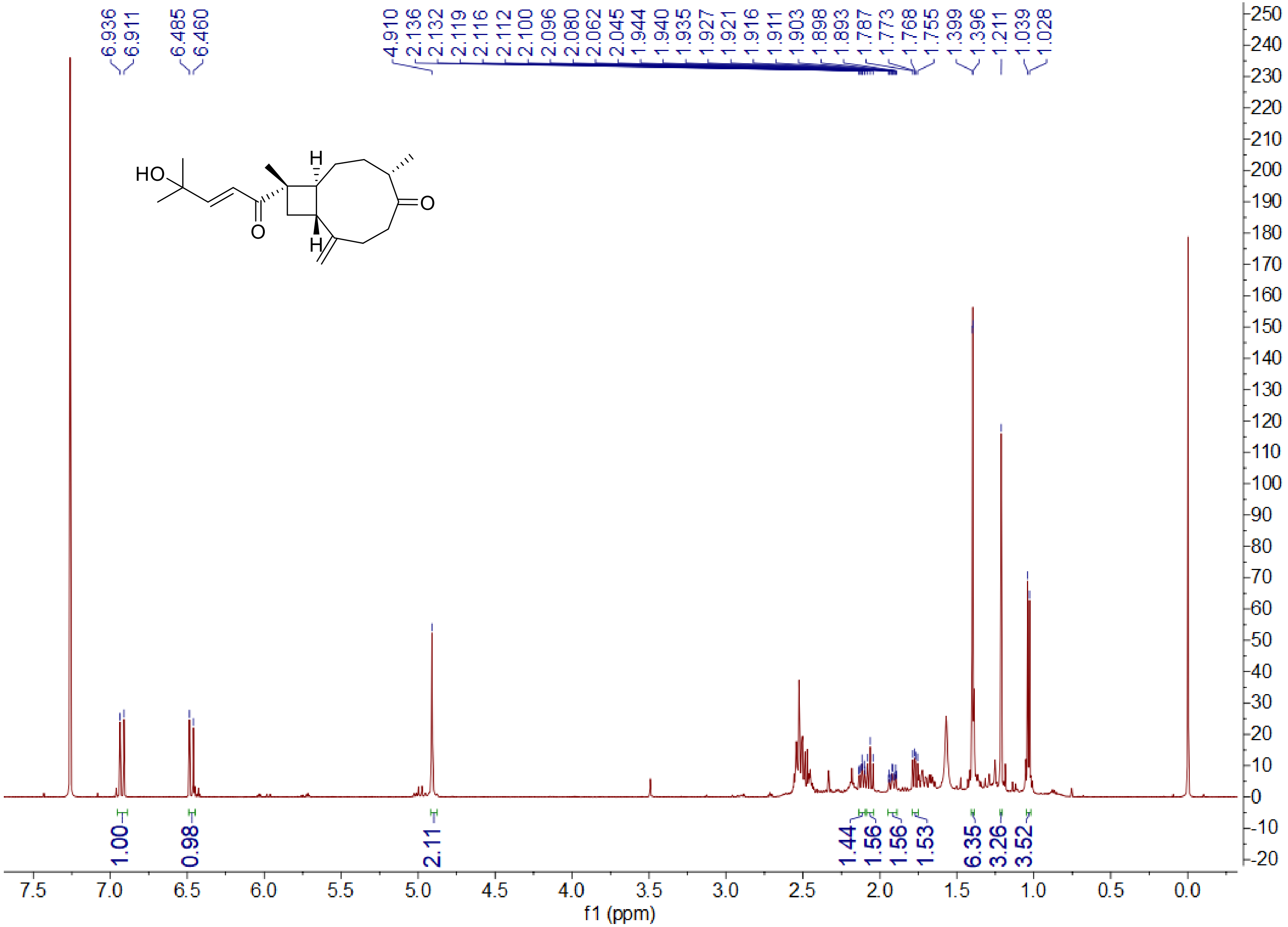

Figure S41. ^{1}H NMR spectrum (600 MHz) of sinuhirtin E (**6**) in $CDCl_3$.

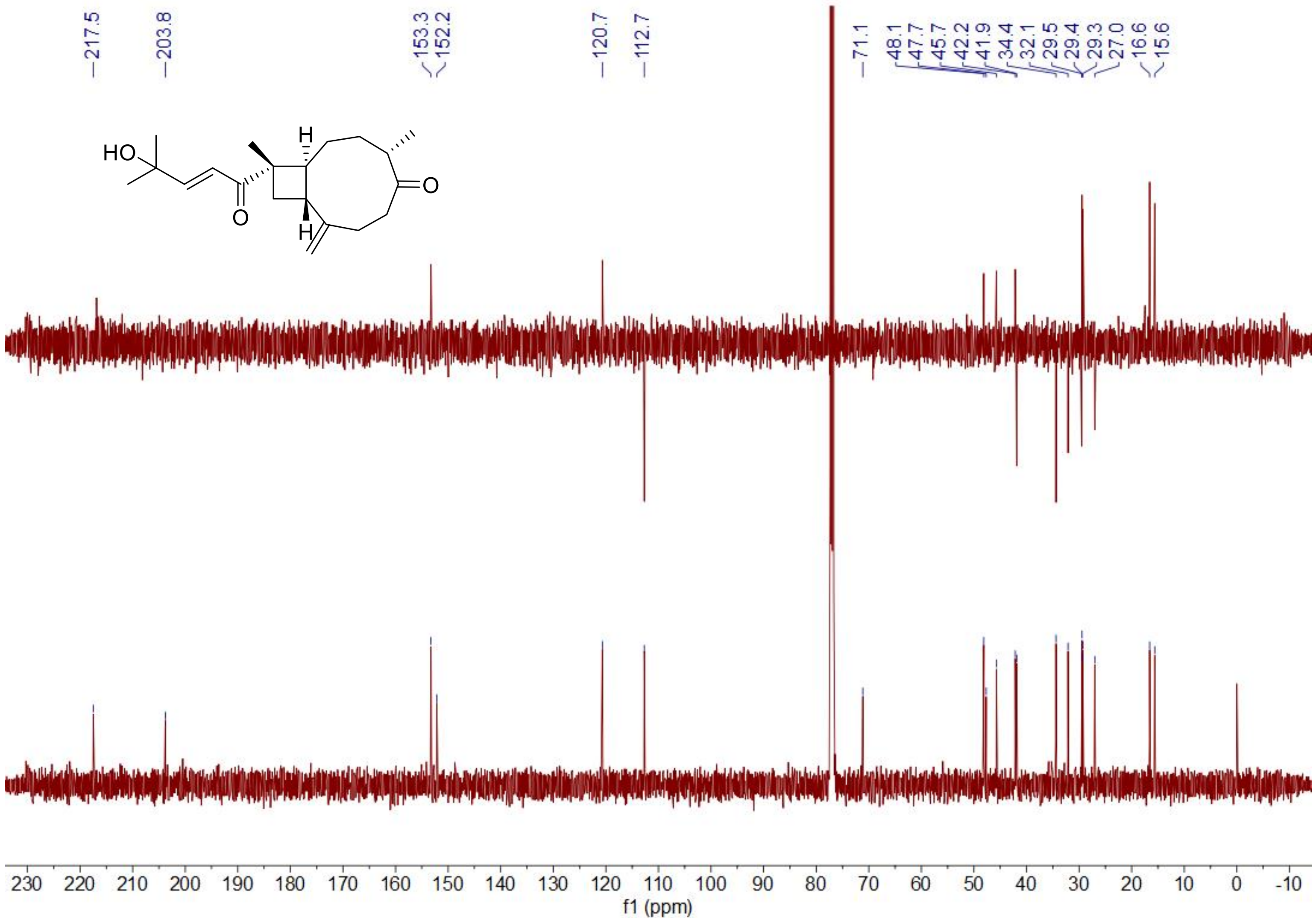

Figure S42. ^{13}C NMR spectrum (125 MHz) of sinuhirtin E (**6**) in $CDCl_3$.

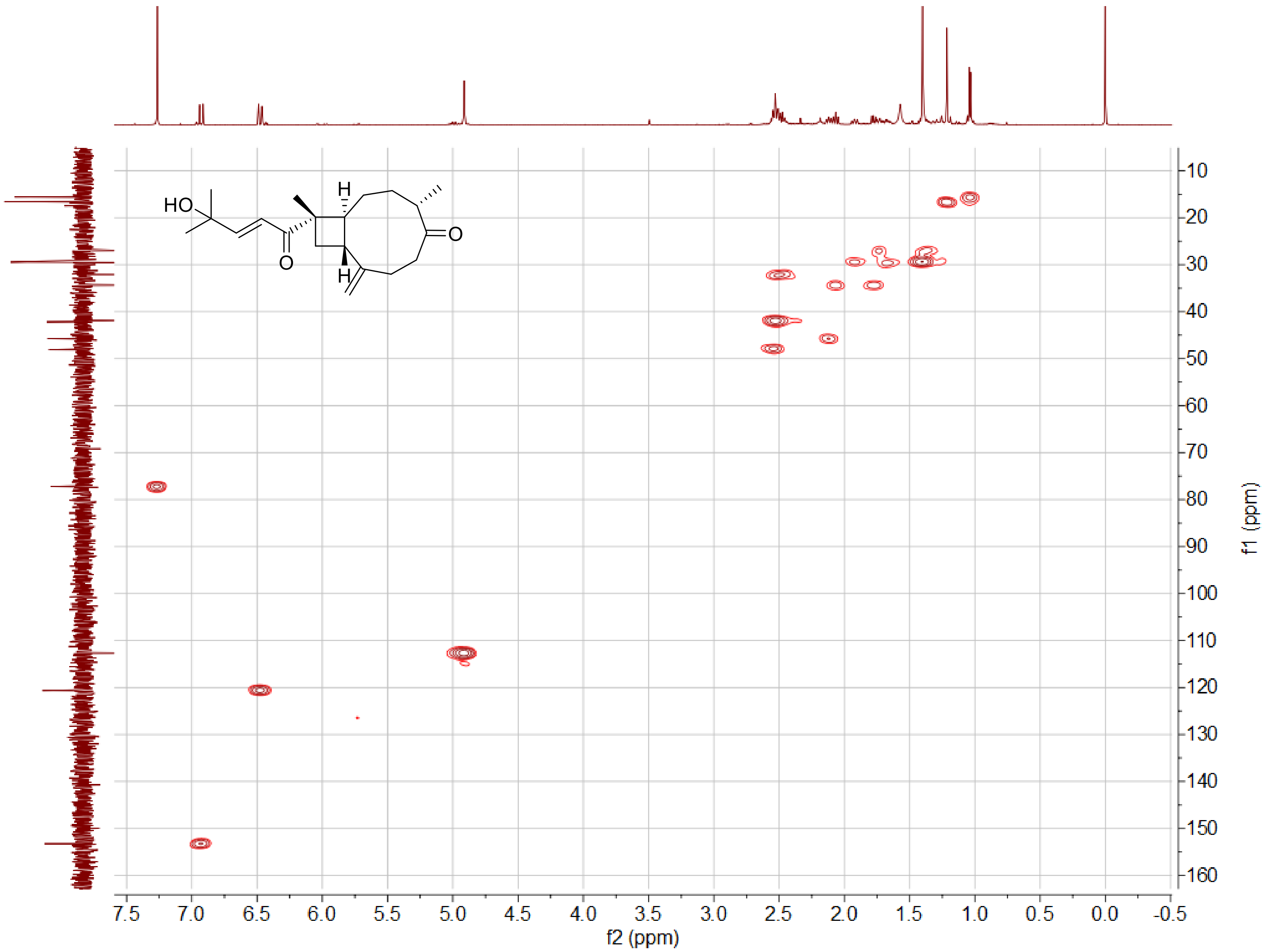

Figure S43. HSQC spectrum (500 MHz) of sinuhirtin E (**6**) in $CDCl_3$.

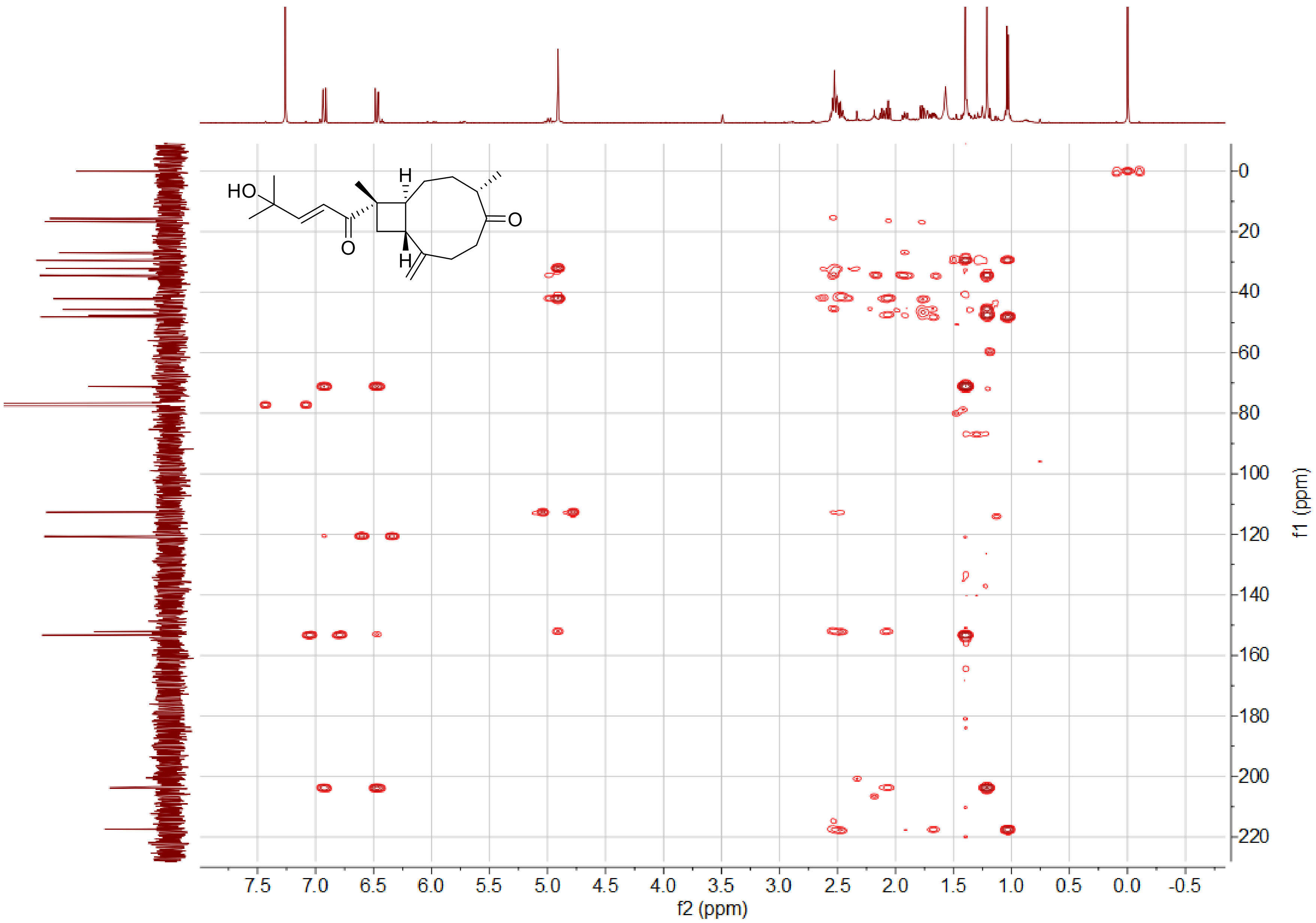

Figure S44. HMBC spectrum (500 MHz) of sinuhirtin E (**6**) in $CDCl_3$.

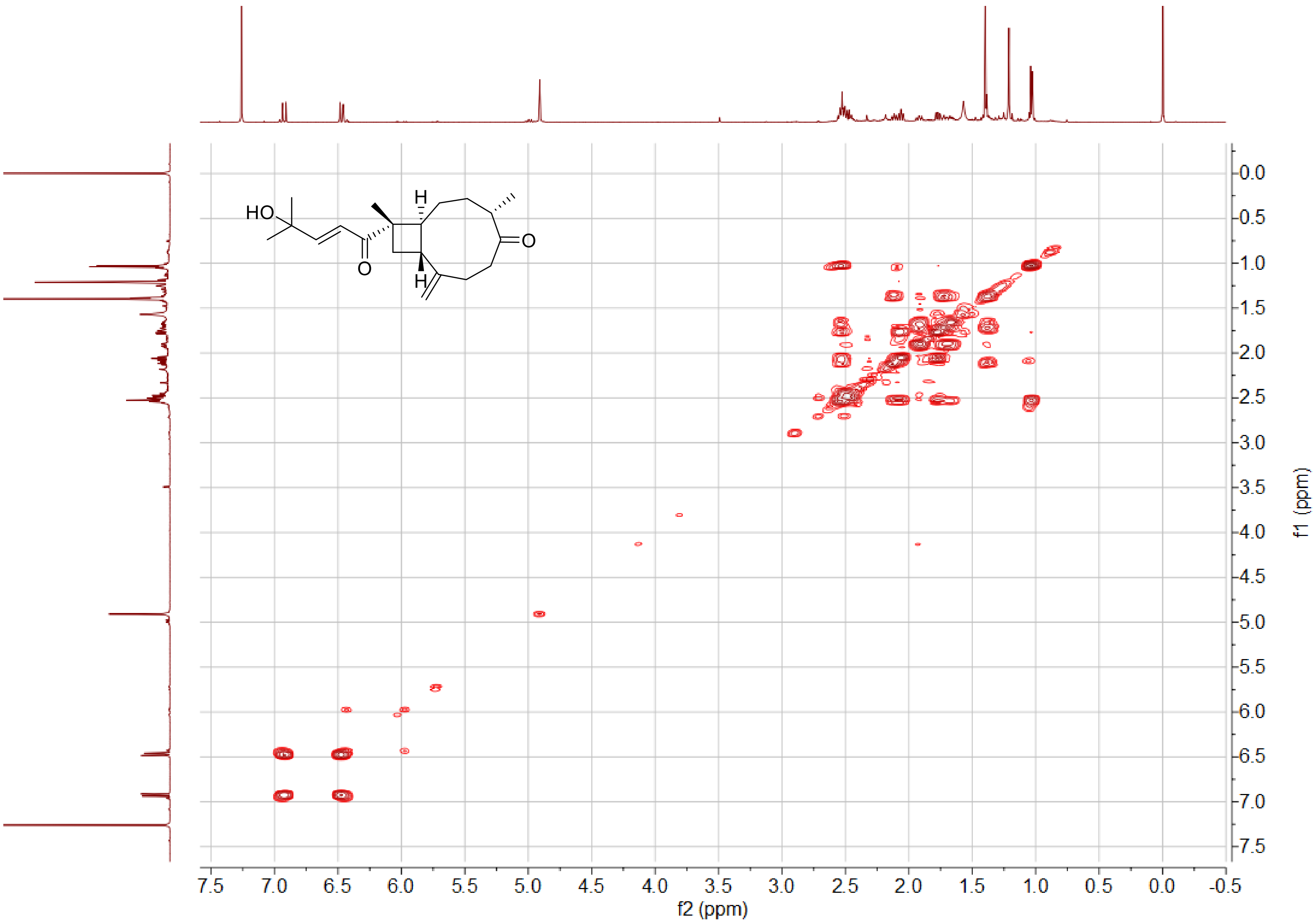

Figure S45. COSY spectrum (500 MHz) of sinuhirtin E (**6**) in $CDCl_3$.

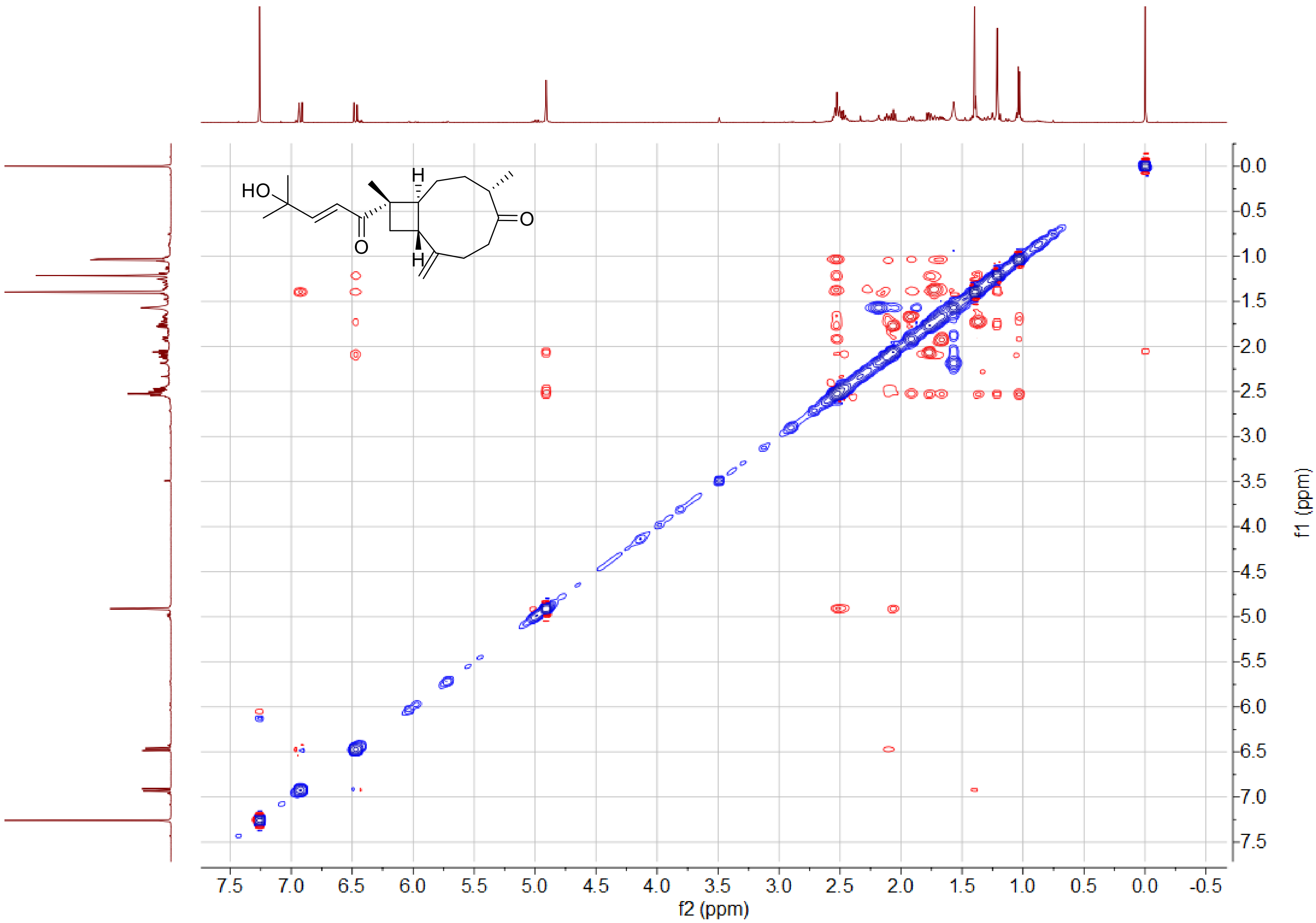

Figure S46. NOESY spectrum (500 MHz) of sinuhirtin E (**6**) in $CDCl_3$.

Fragmentor Voltage	Collision Energy	Ionization Mode
125	0	ESI

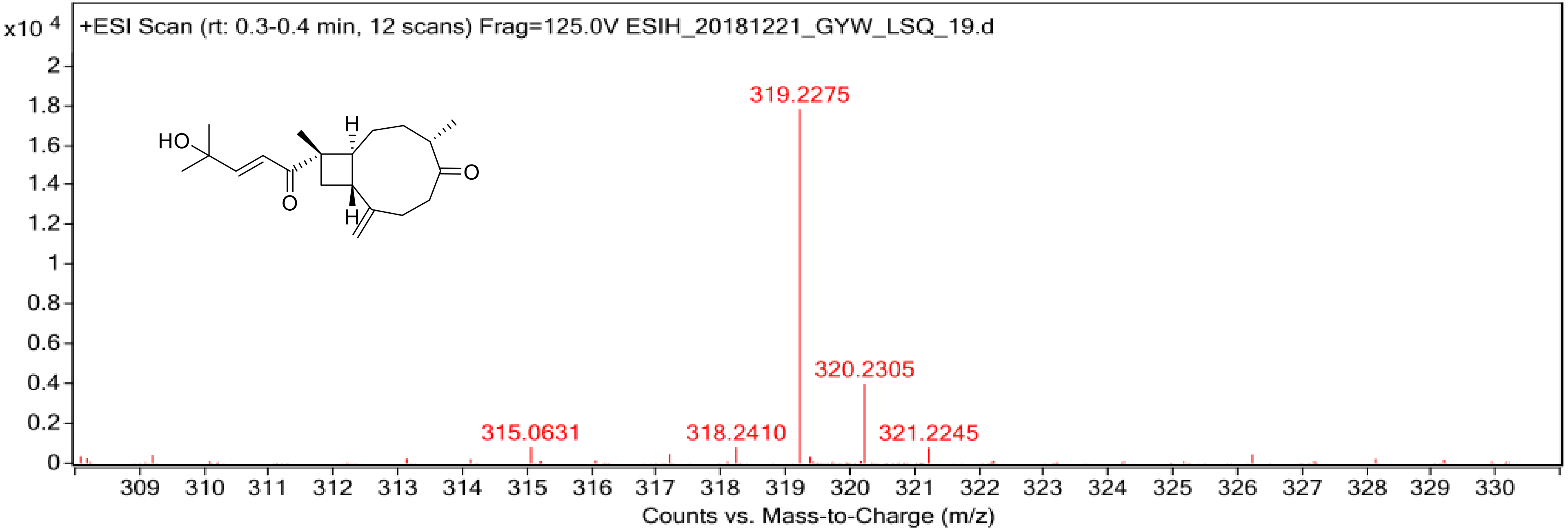

Formula Calculator Results

m/z	Calc m/z	Diff (mDa)	Diff (ppm)	Ion Formula	Ion
319.2275	319.2268	-0.71	-2.23	C20 H31 O3	(M+H)+

Figure S47. HRESIMS spectrum of sinuhirtin E (**6**) in MeOH.

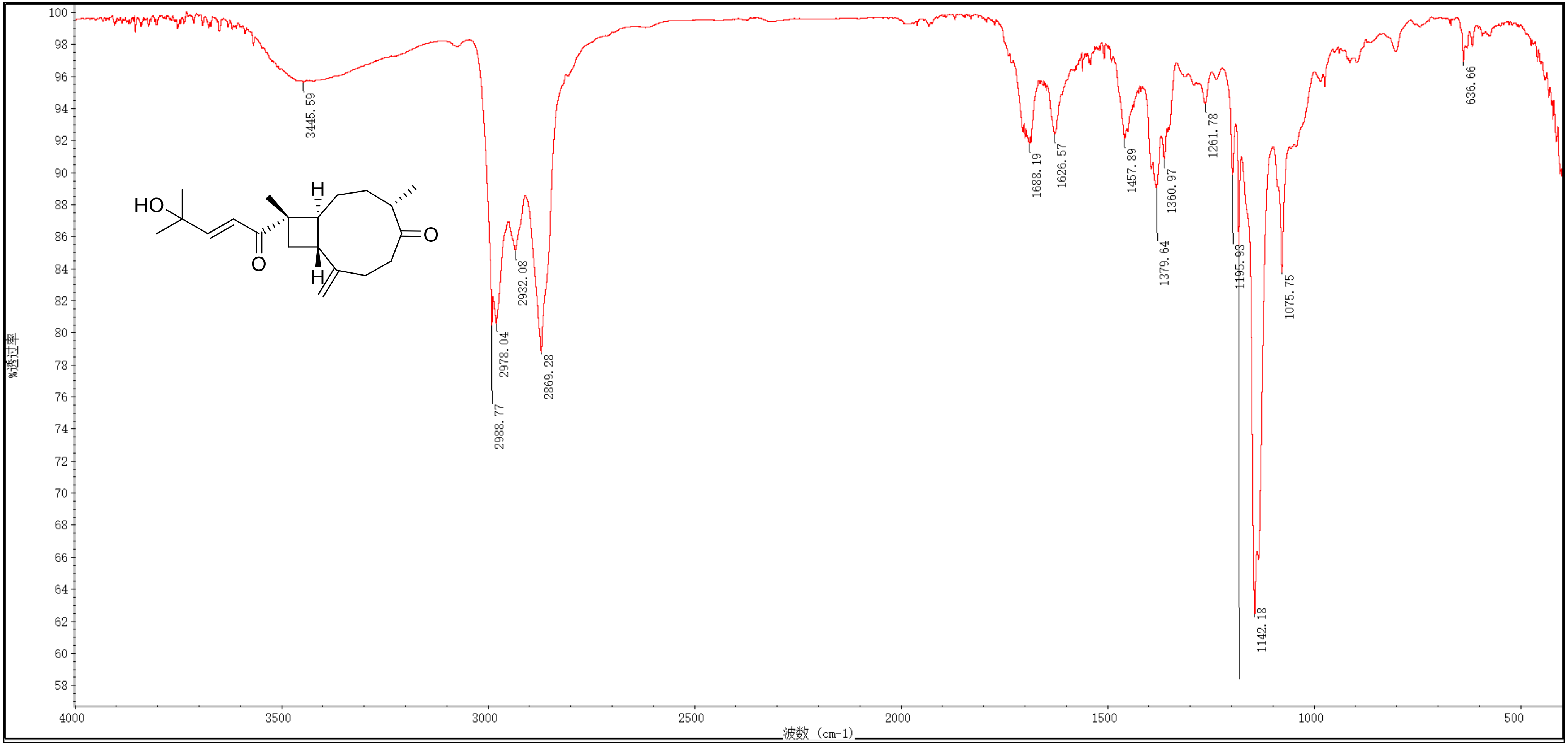

Figure S48. IR spectrum of sinuhirtin E (**6**).

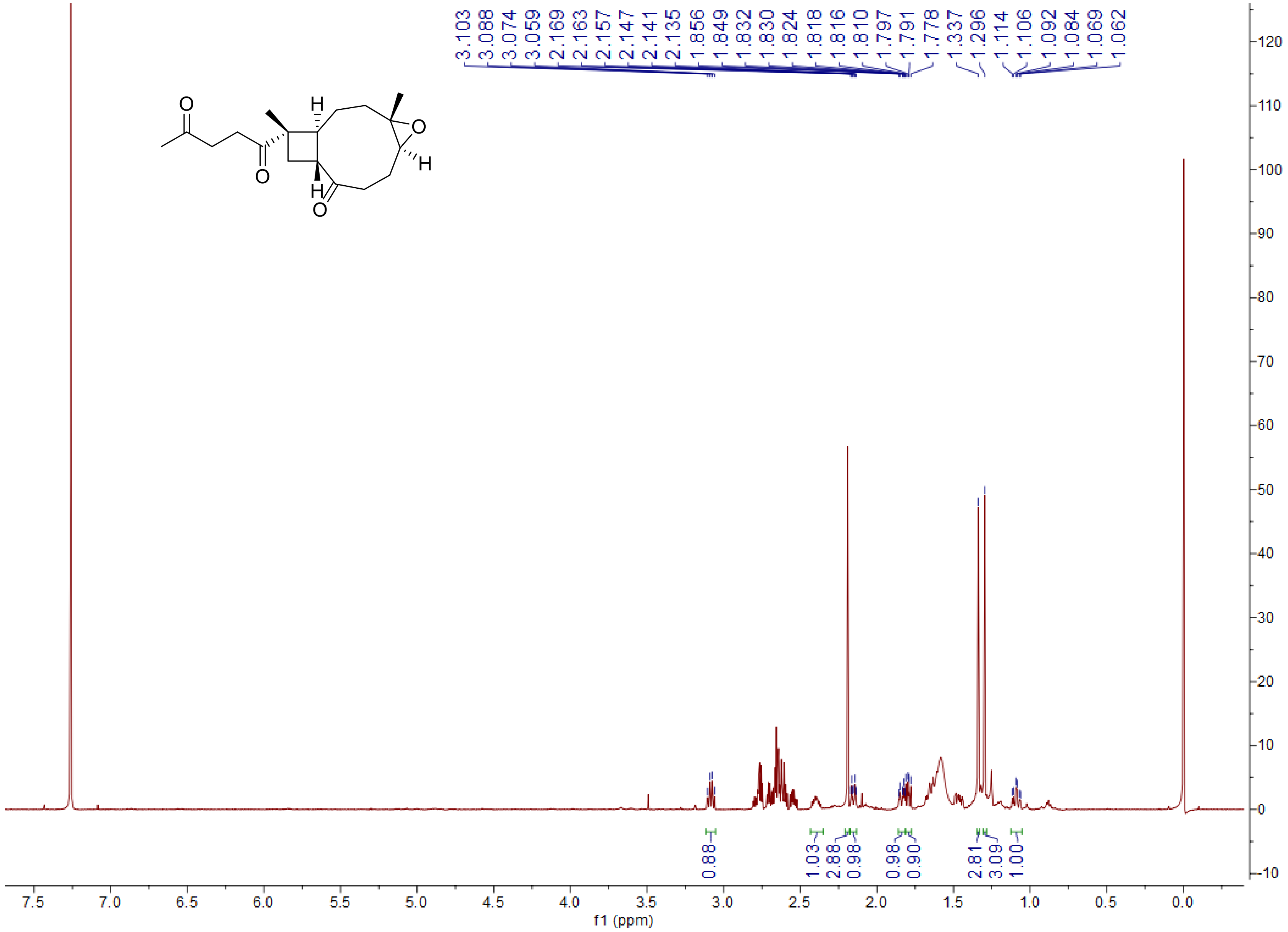

Figure S49. ^{1}H NMR spectrum (600 MHz) of sinuhirtone A (**7**) in $CDCl_3$.

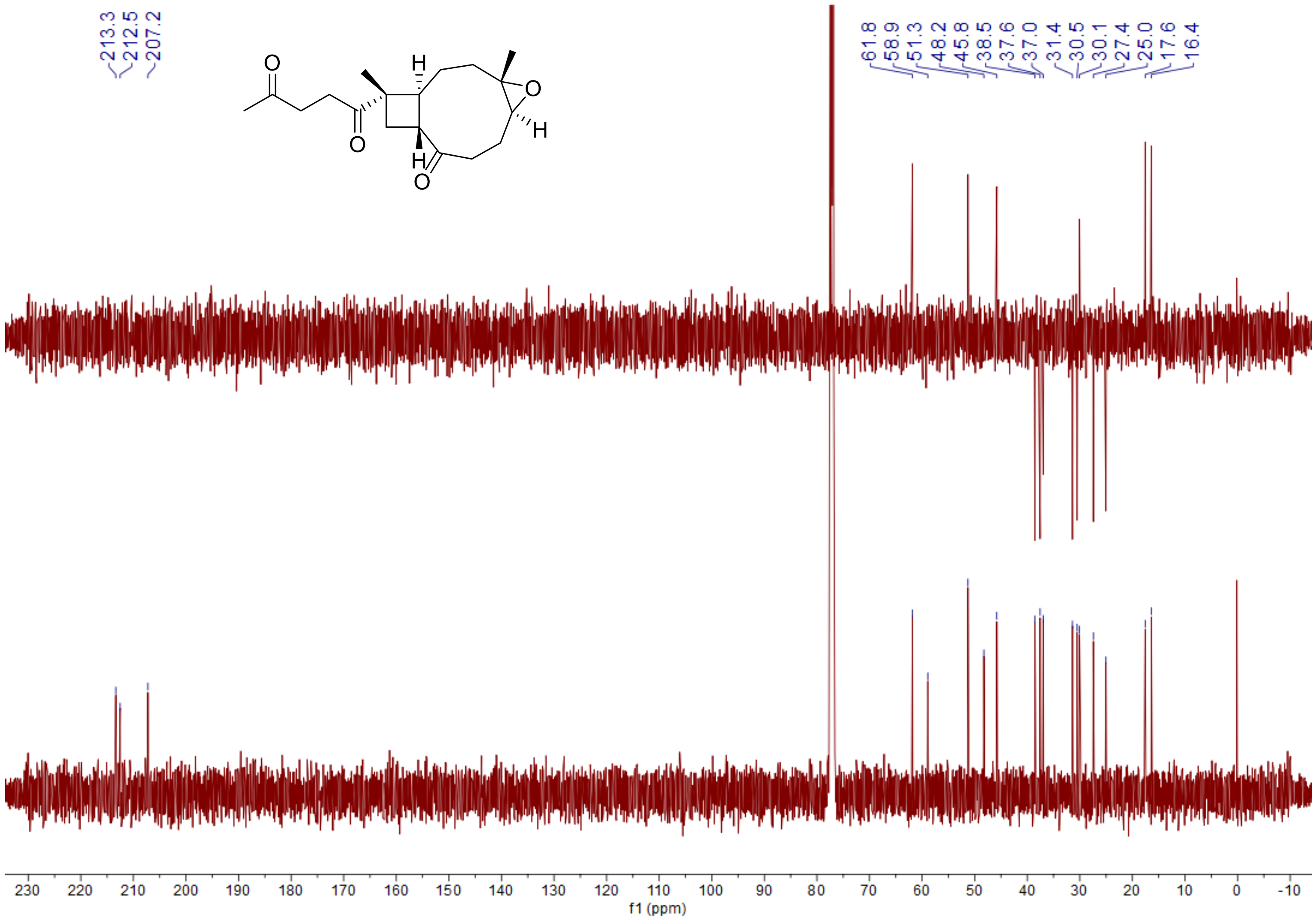

Figure S50. ^{13}C NMR spectrum (125 MHz) of sinuhirtone A (**7**) in $CDCl_3$.

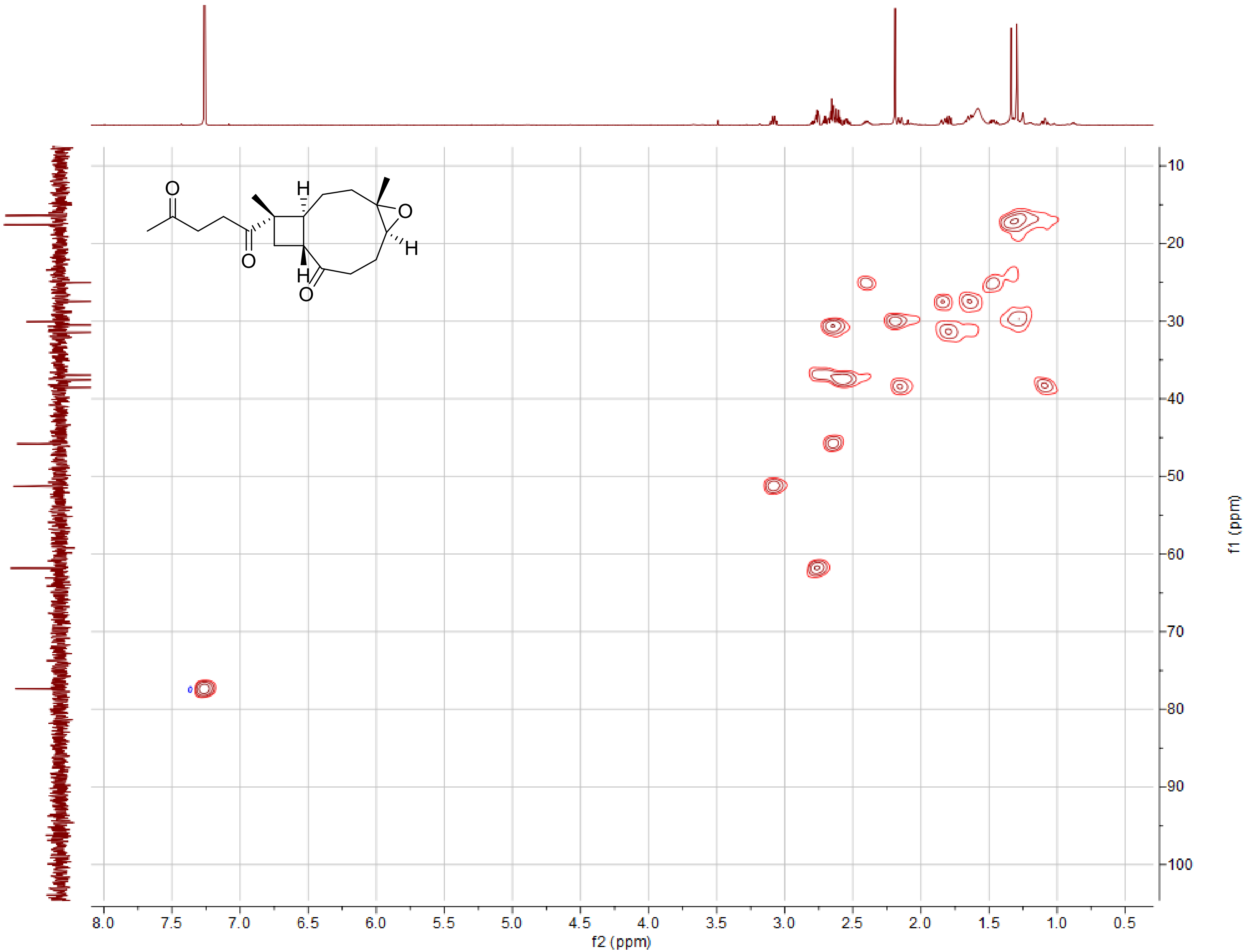

Figure S51. HSQC spectrum (500 MHz) of sinuhirtone A (**7**) in $CDCl_3$.

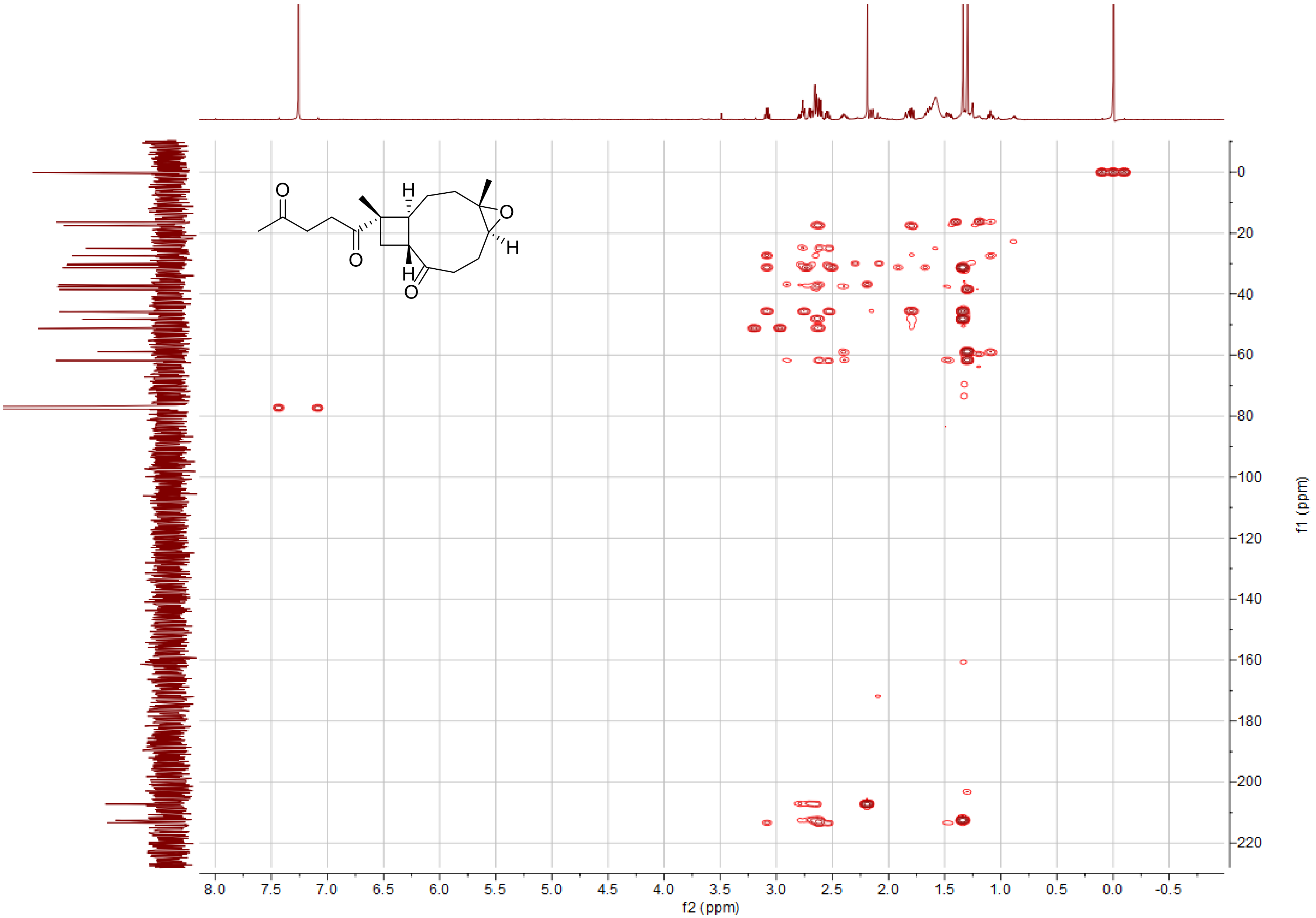

Figure S52. HMBC spectrum (500 MHz) of sinuhirtone A (**7**) in $CDCl_3$.

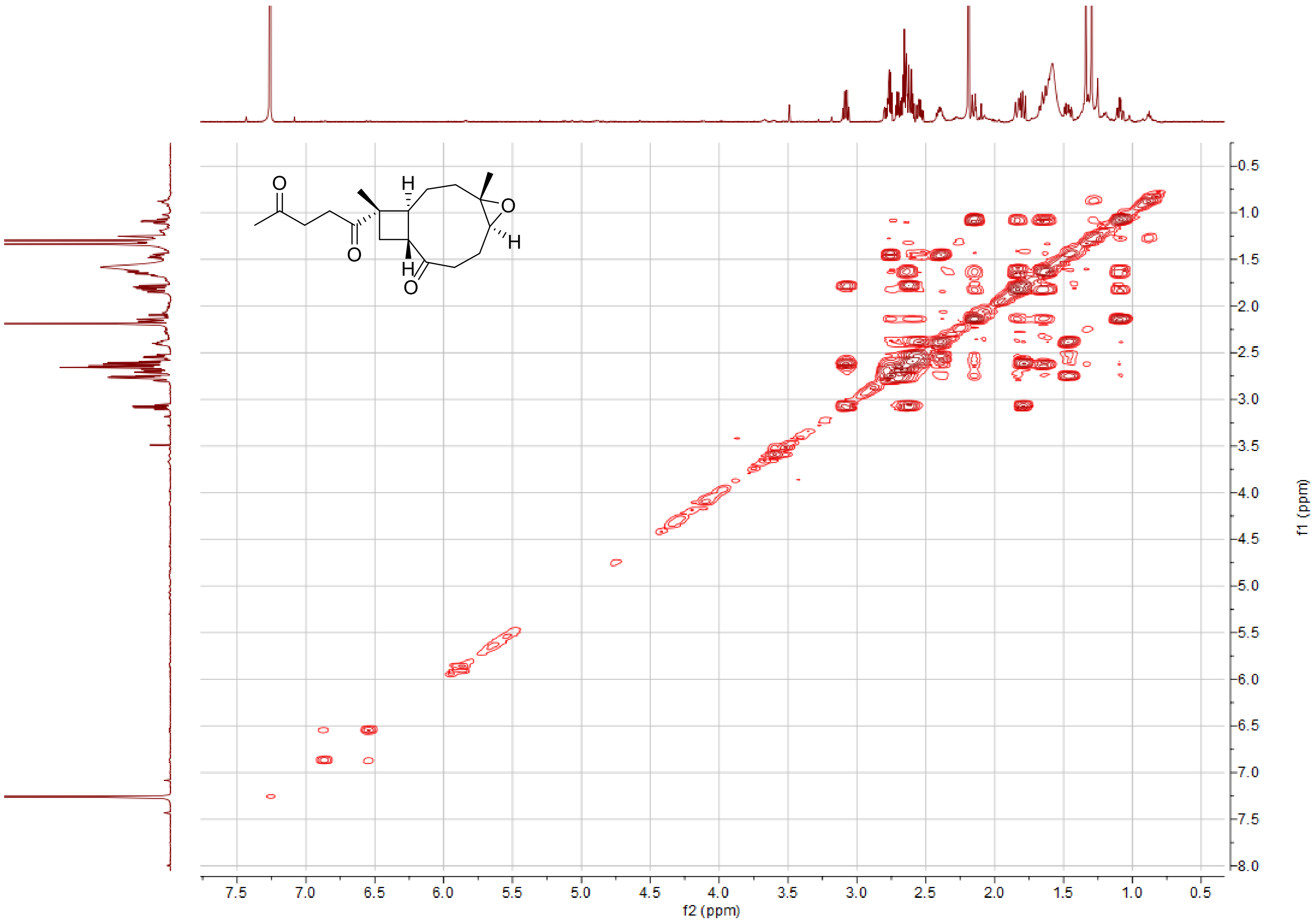

Figure S53. COSY spectrum (500 MHz) of sinuhirtone A (**7**) in $CDCl_3$.

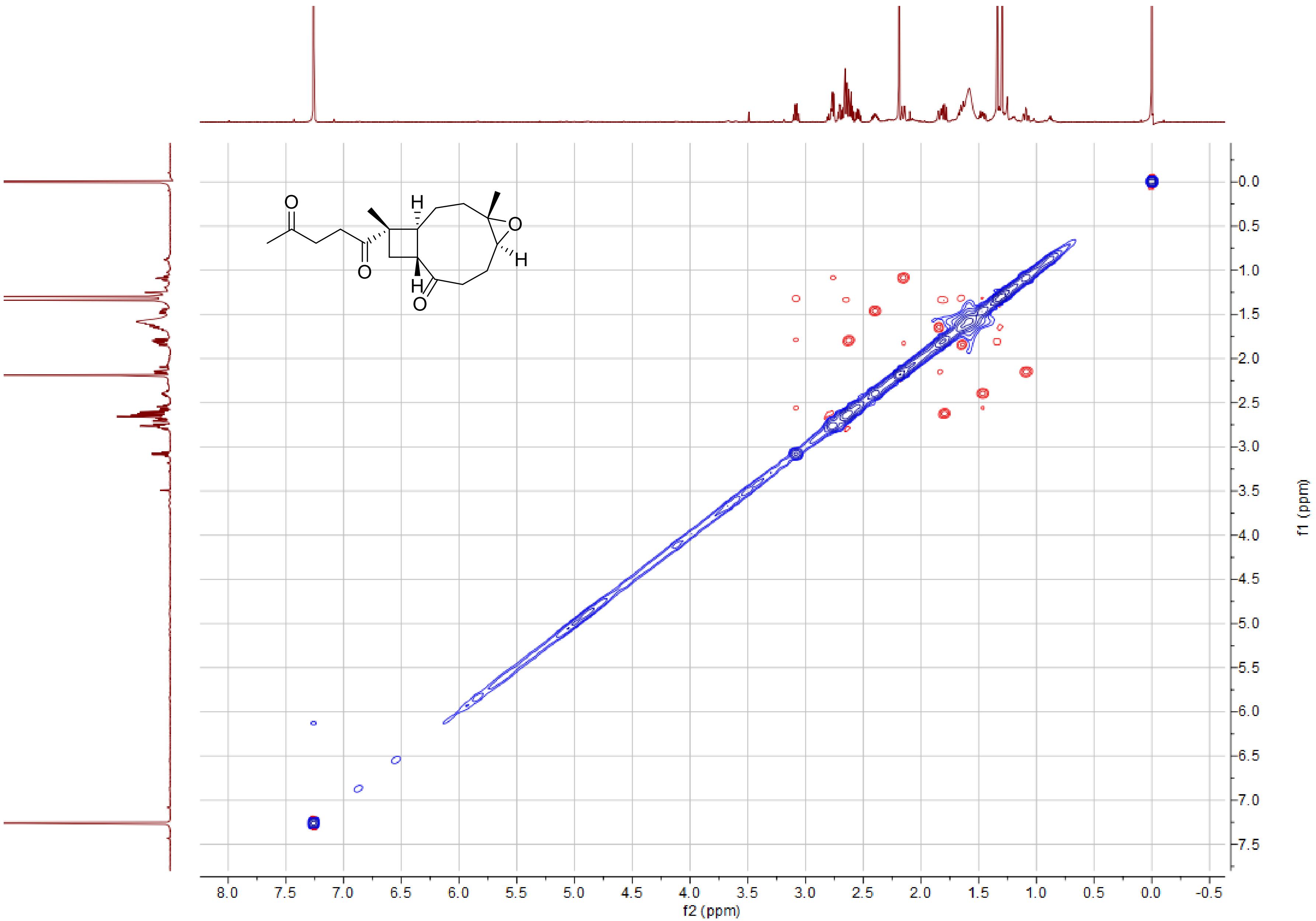

Figure S54. NOESY spectrum (500 MHz) of sinuhirtone A (**7**) in $CDCl_3$.

Fragmentor Voltage	Collision Energy	Ionization Mode
135	0	ESI

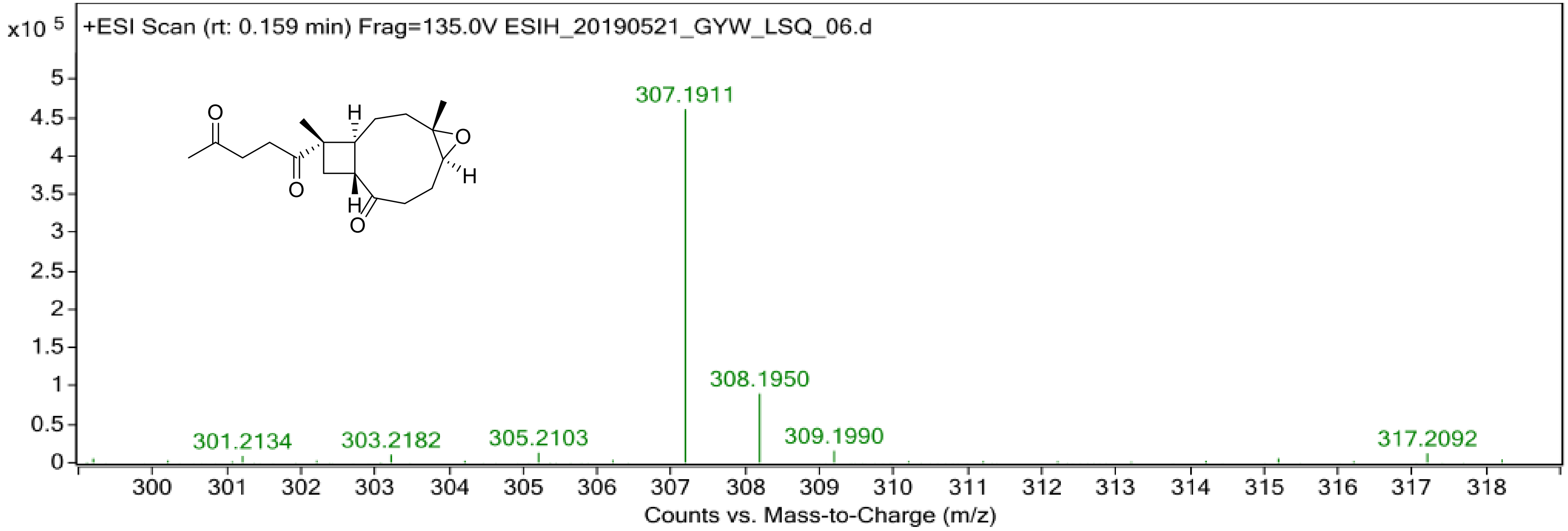

Formula Calculator Results

m/z	Calc m/z	Diff (mDa)	Diff (ppm)	Ion Formula	Ion
307.1911	307.1904	-0.71	-2.32	C18 H27 O4	(M+H)+

Figure S55. HRESIMS spectrum of sinuhirtone A (**7**) in MeOH.

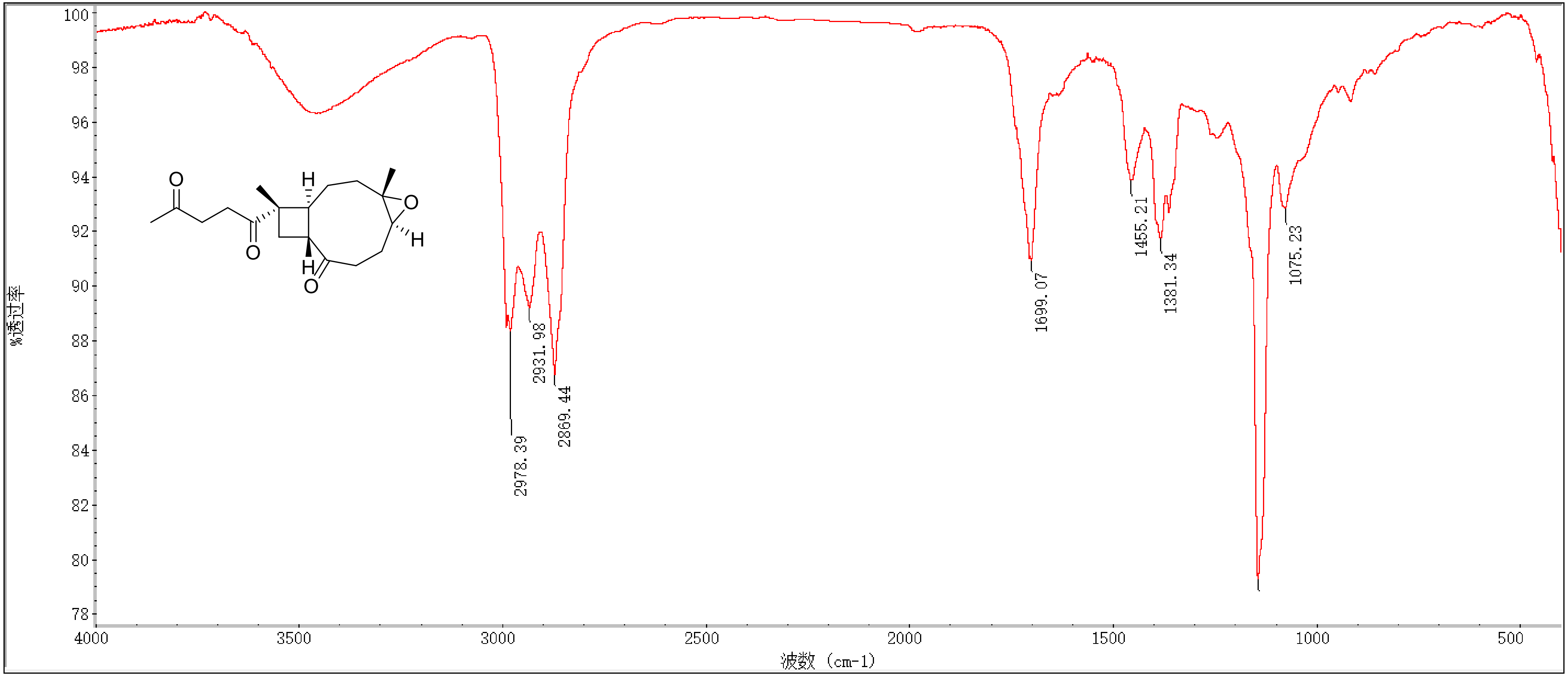

Figure S56. IR spectrum of sinuhirtone A (**7**).

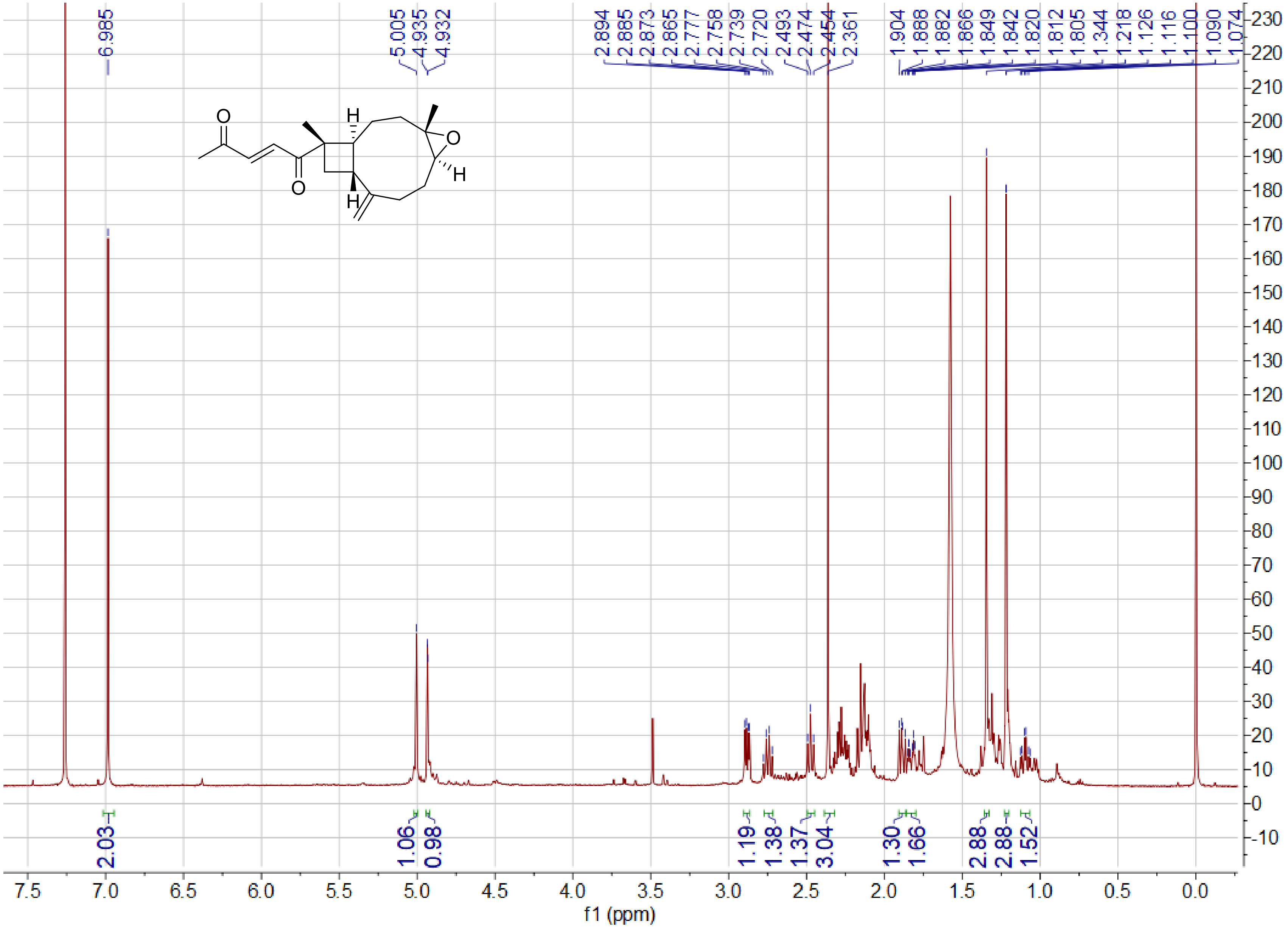

Figure S57. ^{1}H NMR spectrum (500 MHz) of sinuhirtone B (**8**) in $CDCl_3$.

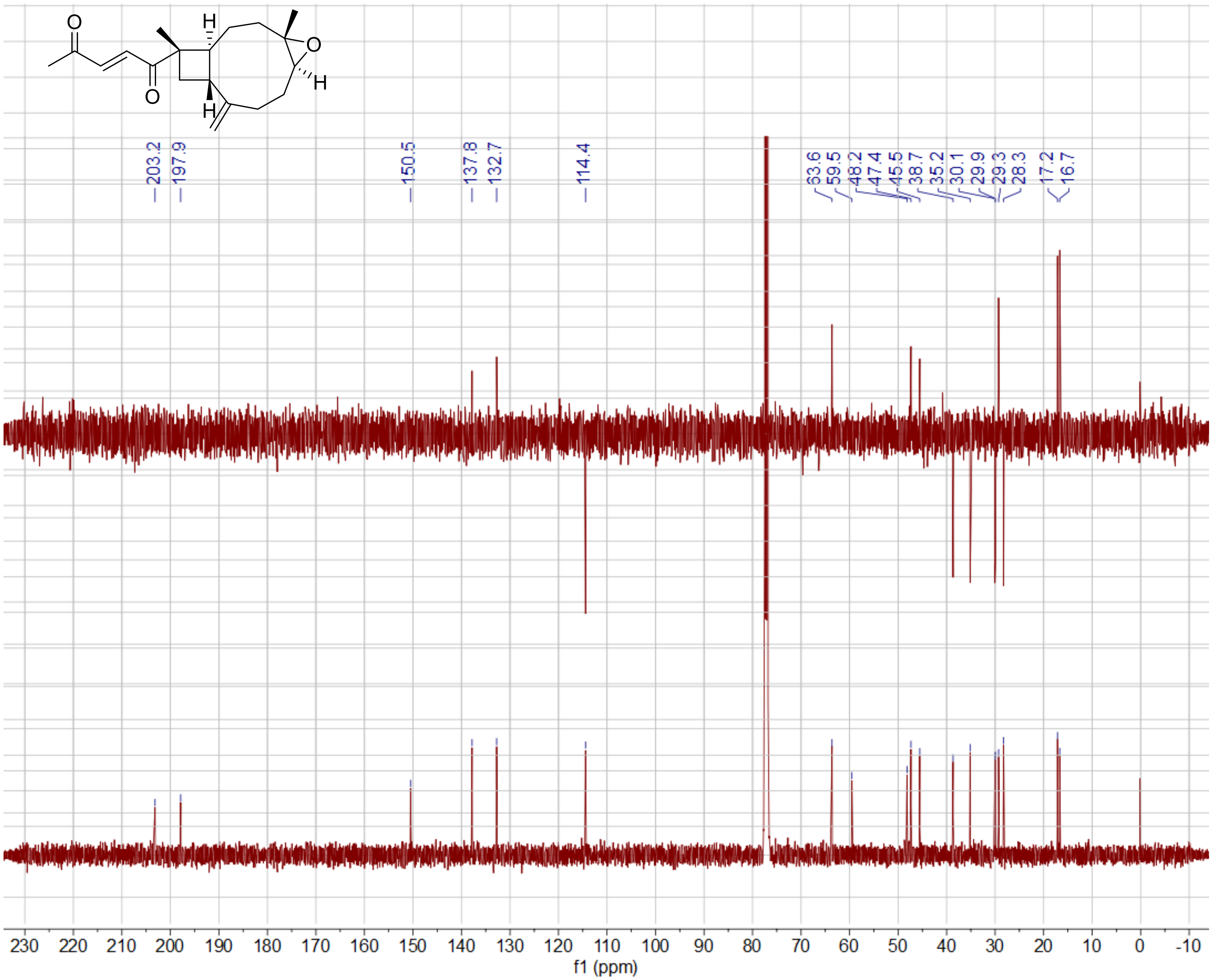

Figure S58. ^{13}C NMR spectrum (125 MHz) of sinuhirtone B (**8**) in $CDCl_3$.

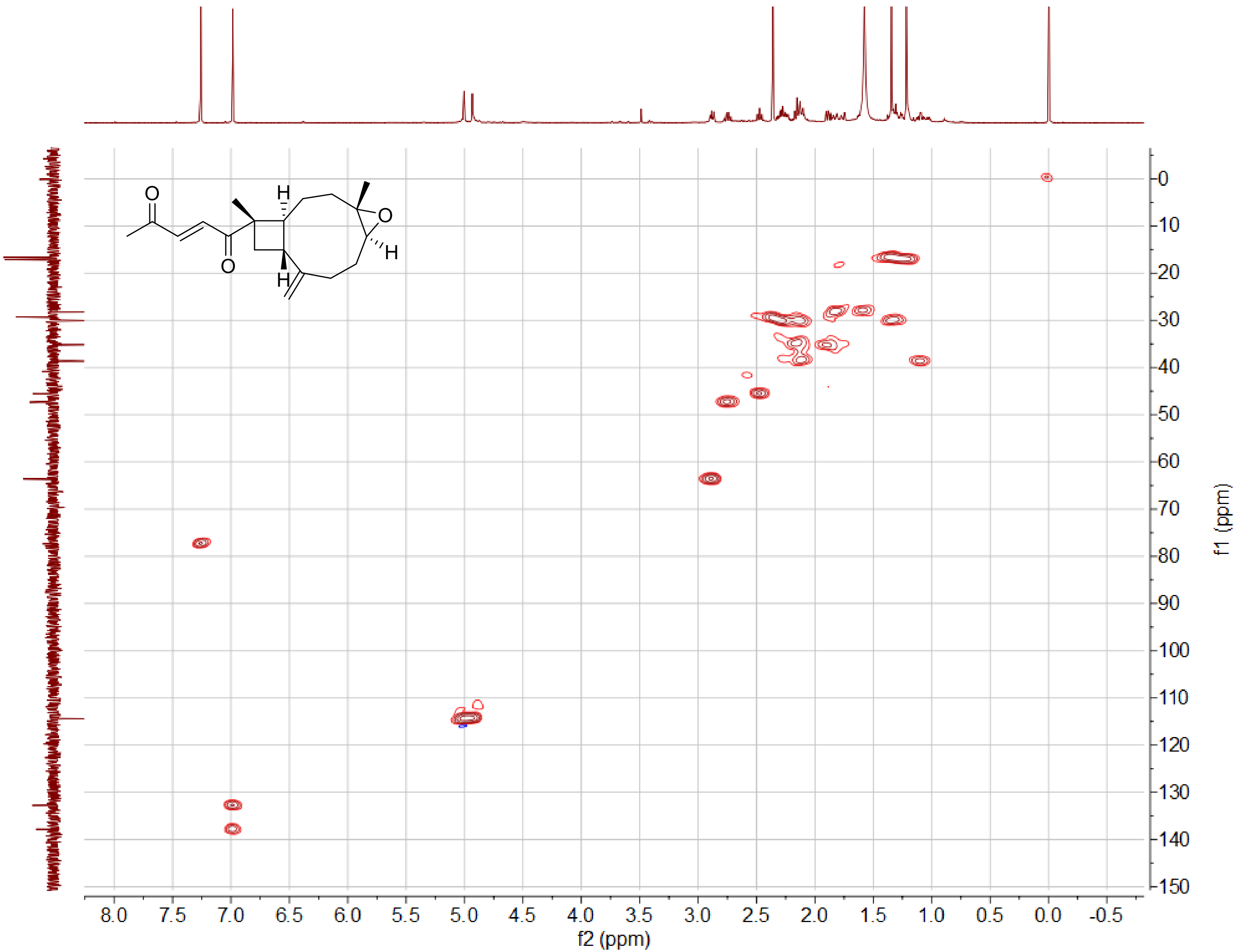

Figure S59. HSQC spectrum (500 MHz) of sinuhirtone B (**8**) in $CDCl_3$.

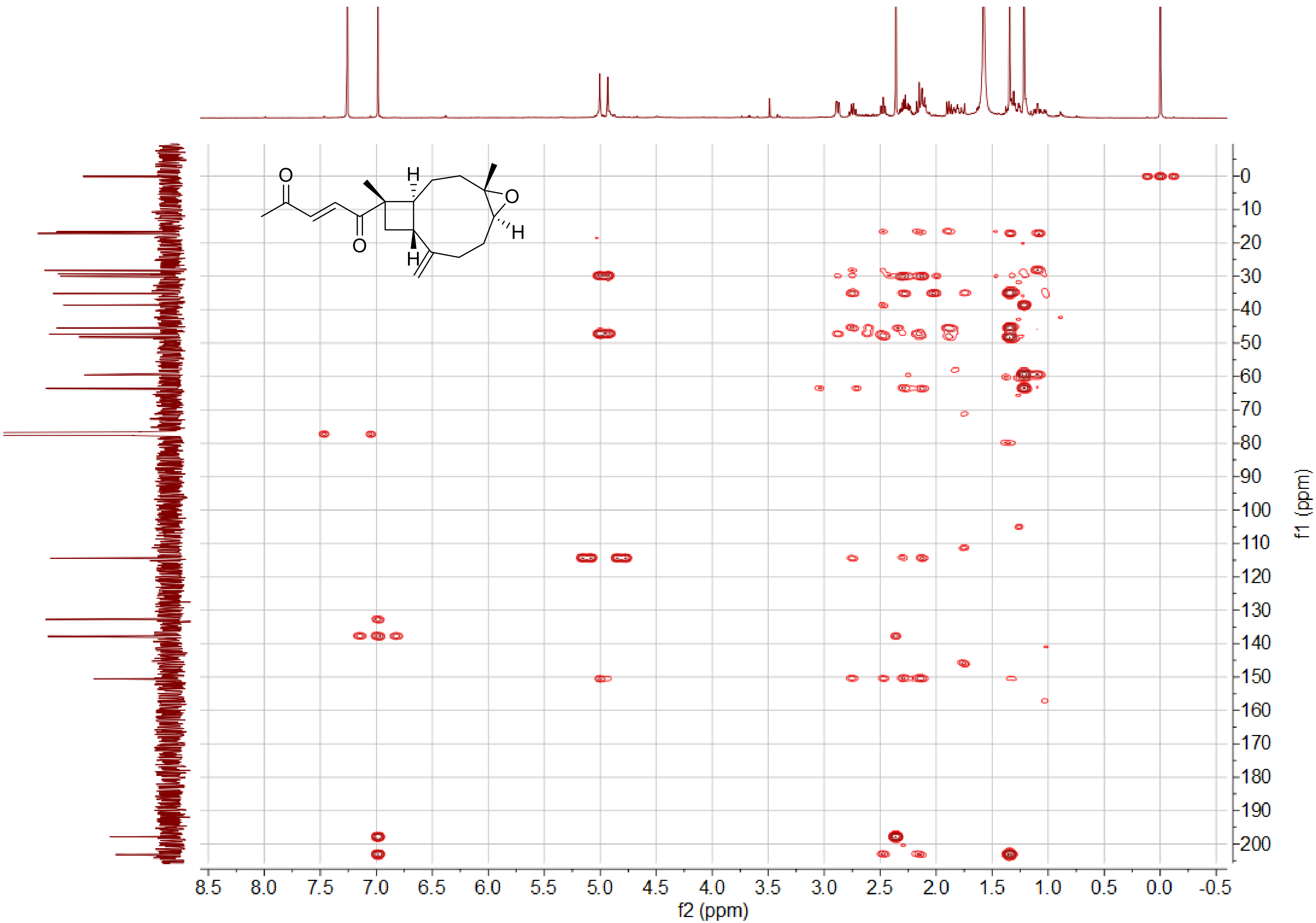

Figure S60. HMBC spectrum (500 MHz) of sinuhirtone B (**8**) in $CDCl_3$.

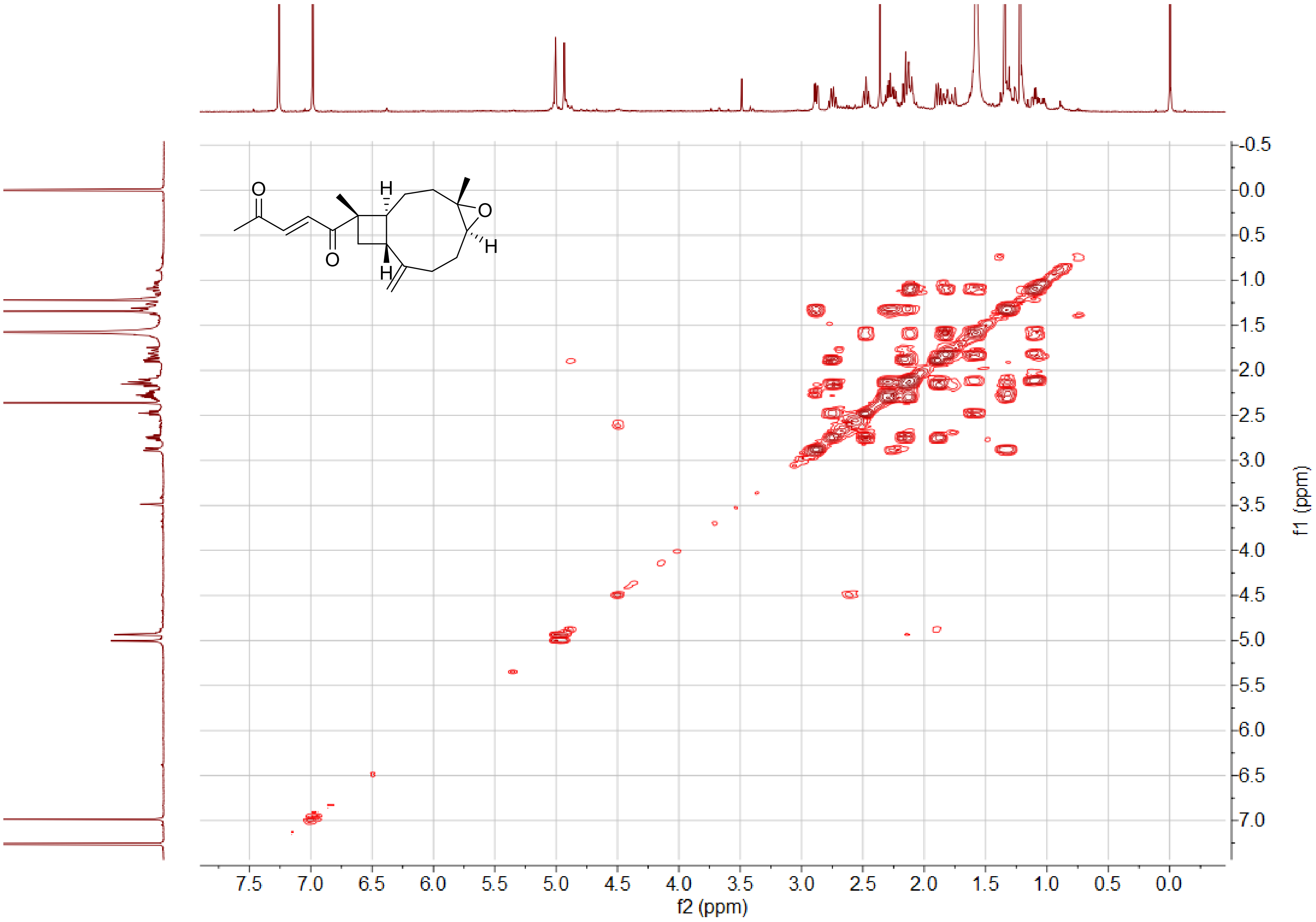

Figure S61. COSY spectrum (500 MHz) of sinuhirtone B (**8**) in $CDCl_3$.

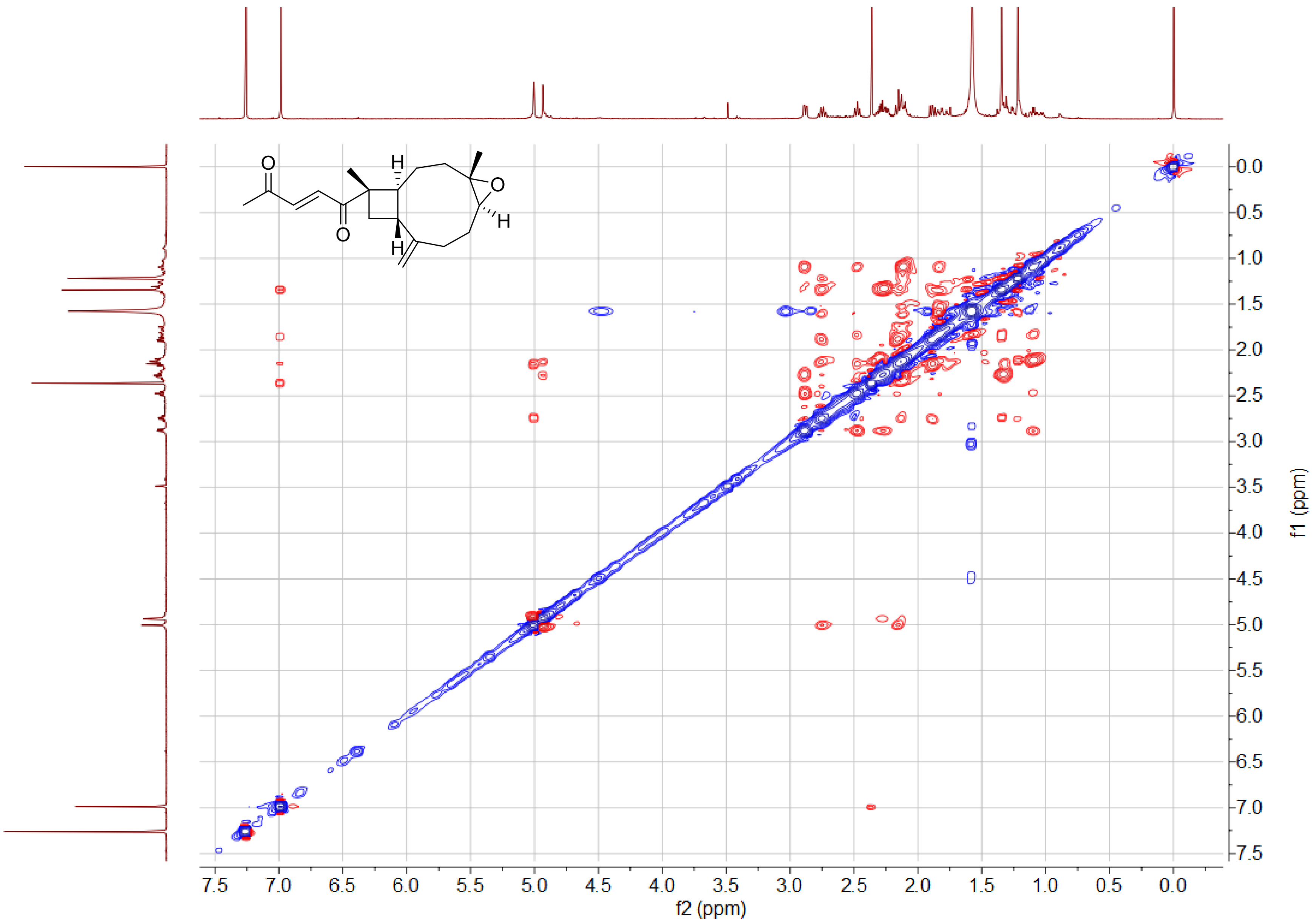

Figure S62. NOESY spectrum (500 MHz) of sinuhirtone B (**8**) in $CDCl_3$.

EIH-2019040307-GYW-LSQ_A8-L3-DEE2-c1#1 RT: 4.75
T: + c EI Full ms [49.50-800.50]
m/z= 88-314

m/z	Intensity	Relative	Theo. Mass	Delta (mmu)	RDB equiv.	Composition
89.0388	139816.0	3.80	89.0386	0.23	5.5	C_7H_5
91.0542	3684163.0	100.00	91.0542	-0.05	4.5	C_7H_7
92.0610	596071.0	16.18	92.0621	-1.01	4.0	C_7H_8
93.0342	62769.0	1.70	93.0335	0.72	4.5	$C_6H_5O_1$
93.0696	2991752.0	81.21	93.0699	-0.27	3.5	C_7H_9
94.0402	91030.0	2.47	94.0413	-1.12	4.0	$C_6H_6O_1$
94.0764	659197.0	17.89	94.0777	-1.32	3.0	C_7H_{10}
95.0489	901471.0	24.47	95.0491	-0.22	3.5	$C_6H_7O_1$
95.0853	1895043.0	51.44	95.0855	-0.23	2.5	C_7H_{11}
96.0566	468783.0	12.72	96.0570	-0.37	3.0	$C_6H_8O_1$
96.0911	330812.0	8.98	96.0934	-2.27	2.0	C_7H_{12}
97.0282	785935.0	21.33	97.0284	-0.22	3.5	$C_5H_5O_2$
97.0642	891630.0	24.20	97.0648	-0.57	2.5	$C_6H_9O_1$
97.0999	667484.0	18.12	97.1012	-1.25	1.5	C_7H_{13}
98.0358	916492.0	24.88	98.0362	-0.44	3.0	$C_5H_6O_2$
98.0709	141013.0	3.83	98.0726	-1.73	2.0	$C_6H_{10}O_1$
98.1066	90901.0	2.47	98.1090	-2.38	1.0	C_7H_{14}
99.0435	289926.0	7.87	99.0441	-0.58	2.5	$C_5H_7O_2$
99.0800	63190.0	1.72	99.0804	-0.42	1.5	$C_6H_{11}O_1$
99.1173	168756.0	4.58	99.1168	0.43	0.5	C_7H_{15}
100.0520	52669.0	1.43	100.0519	0.17	2.0	$C_5H_8O_2$
101.0606	156131.0	4.24	101.0597	0.92	1.5	$C_5H_9O_2$
103.0560	379015.0	10.29	103.0542	1.80	5.5	C_8H_7
104.0647	220779.0	5.99	104.0621	2.67	5.0	C_8H_8
112.0912	116831.0	3.17	112.0883	2.89	2.0	$C_7H_{12}O_1$
116.0638	275585.0	7.48	116.0621	1.70	6.0	C_9H_8
117.0717	1073563.0	29.14	117.0699	1.78	5.5	C_9H_9
118.0778	392481.0	10.65	118.0777	0.13	5.0	C_9H_{10}
119.0494	99383.0	2.70	119.0491	0.29	5.5	$C_8H_7O_1$
119.0859	2021974.0	54.88	119.0855	0.34	4.5	C_9H_{11}
120.0565	59079.0	1.60	120.0570	-0.45	5.0	$C_8H_8O_1$
120.0922	723974.0	19.65	120.0934	-1.11	4.0	C_9H_{12}
121.0641	812771.0	22.06	121.0648	-0.69	4.5	$C_8H_9O_1$
121.1004	1228399.0	33.34	121.1012	-0.82	3.5	C_9H_{13}
122.0716	971557.0	26.37	122.0726	-1.06	4.0	$C_8H_{10}O_1$
123.0432	75168.0	2.04	123.0441	-0.82	4.5	$C_7H_7O_2$
123.0787	990916.0	26.90	123.0804	-1.78	3.5	$C_8H_{11}O_1$
123.1154	295785.0	8.03	123.1168	-1.43	2.5	C_9H_{15}
124.0510	99415.0	2.70	124.0519	-0.85	4.0	$C_7H_8O_2$
124.0870	514525.0	13.97	124.0883	-1.30	3.0	$C_8H_{12}O_1$
124.1222	105404.0	2.86	124.1247	-2.48	2.0	C_9H_{16}
125.0587	916913.0	24.89	125.0597	-0.99	3.5	$C_7H_9O_2$
125.0946	239943.0	6.51	125.0961	-1.50	2.5	$C_8H_{13}O_1$
125.1315	243116.0	6.60	125.1325	-1.02	1.5	C_9H_{17}
126.1018	49011.0	1.33	126.1039	-2.16	2.0	$C_8H_{14}O_1$
126.1379	53608.0	1.46	126.1403	-2.44	1.0	C_9H_{18}
127.0528	187370.0	5.09	127.0542	-1.47	7.5	$C_{10}H_7$
127.0734	96113.0	2.61	127.0754	-1.95	2.5	$C_7H_{11}O_2$
127.1465	91807.0	2.49	127.1481	-1.63	0.5	C_9H_{19}
128.0612	625627.0	16.98	128.0621	-0.88	7.0	$C_{10}H_8$
129.0547	334049.0	9.07	129.0546	0.12	2.5	$C_6H_9O_3$
129.0688	845597.0	22.95	129.0699	-1.09	6.5	$C_{10}H_9$
130.0768	373382.0	10.13	130.0777	-0.95	6.0	$C_{10}H_{10}$
131.0488	69988.0	1.90	131.0491	-0.33	6.5	$C_9H_7O_1$
131.0850	1949719.0	52.92	131.0855	-0.53	5.5	$C_{10}H_{11}$

EIH-2019040307-GYW-LSQ_A8-L3-DEE2-c1#1 RT: 4.75
T: + c EI Full ms [49.50-800.50]
m/z= 88-314

m/z	Intensity	Relative	Theo. Mass	Delta (mmu)	RDB equiv.	Composition
132.0543	62025.0	1.68	132.0570	-2.64	6.0	$C_9H_8O_1$
132.0914	465190.0	12.63	132.0934	-2.00	5.0	$C_{10}H_{12}$
133.0650	318155.0	8.64	133.0648	0.16	5.5	$C_9H_9O_1$
133.1012	1216745.0	33.03	133.1012	-0.02	4.5	$C_{10}H_{13}$
134.0718	236415.0	6.42	134.0726	-0.81	5.0	$C_9H_{10}O_1$
134.1085	310806.0	8.44	134.1090	-0.52	4.0	$C_{10}H_{14}$
135.0432	130525.0	3.54	135.0441	-0.87	5.5	$C_8H_7O_2$
135.0802	759389.0	20.61	135.0804	-0.27	4.5	$C_9H_{11}O_1$
135.1163	470693.0	12.78	135.1168	-0.50	3.5	$C_{10}H_{15}$
136.0512	52831.0	1.43	136.0519	-0.67	5.0	$C_8H_8O_2$
136.0880	372281.0	10.10	136.0883	-0.31	4.0	$C_9H_{12}O_1$
136.1218	103947.0	2.82	136.1247	-2.83	3.0	$C_{10}H_{16}$
137.0594	145448.0	3.95	137.0597	-0.32	4.5	$C_8H_9O_2$
137.0952	455349.0	12.36	137.0961	-0.91	3.5	$C_9H_{13}O_1$
137.1325	142049.0	3.86	137.1325	0.03	2.5	$C_{10}H_{17}$
138.0672	90383.0	2.45	138.0675	-0.33	4.0	$C_8H_{10}O_2$
138.1044	333337.0	9.05	138.1039	0.53	3.0	$C_9H_{14}O_1$
139.0530	57816.0	1.57	139.0542	-1.26	8.5	$C_{11}H_7$
139.0758	157070.0	4.26	139.0754	0.49	3.5	$C_8H_{11}O_2$
139.1104	136805.0	3.71	139.1117	-1.31	2.5	$C_9H_{15}O_1$
139.1473	75071.0	2.04	139.1481	-0.81	1.5	$C_{10}H_{19}$
140.0824	237321.0	6.44	140.0832	-0.73	3.0	$C_8H_{12}O_2$
141.0699	360757.0	9.79	141.0699	-0.00	7.5	$C_{11}H_9$
141.0902	54029.0	1.47	141.0910	-0.80	2.5	$C_8H_{13}O_2$
141.1637	73096.0	1.98	141.1638	-0.05	0.5	$C_{10}H_{21}$
142.0772	319903.0	8.68	142.0777	-0.48	7.0	$C_{11}H_{10}$
143.0850	864923.0	23.48	143.0855	-0.57	6.5	$C_{11}H_{11}$
144.0921	403035.0	10.94	144.0934	-1.30	6.0	$C_{11}H_{12}$
145.0639	193974.0	5.27	145.0648	-0.93	6.5	$C_{10}H_9O_1$
145.1013	1987627.0	53.95	145.1012	0.10	5.5	$C_{11}H_{13}$
146.0733	124083.0	3.37	146.0726	0.68	6.0	$C_{10}H_{10}O_1$
146.1082	638058.0	17.32	146.1090	-0.82	5.0	$C_{11}H_{14}$
147.0801	551689.0	14.97	147.0804	-0.34	5.5	$C_{10}H_{11}O_1$
147.1167	986449.0	26.78	147.1168	-0.12	4.5	$C_{11}H_{15}$
148.0878	260467.0	7.07	148.0883	-0.49	5.0	$C_{10}H_{12}O_1$
148.1234	225537.0	6.12	148.1247	-1.22	4.0	$C_{11}H_{16}$
149.0242	1020343.0	27.70	149.0233	0.88	6.5	$C_8H_5O_3$
149.0595	152052.0	4.13	149.0597	-0.20	5.5	$C_9H_9O_2$
149.0959	751620.0	20.40	149.0961	-0.20	4.5	$C_{10}H_{13}O_1$
149.1335	154933.0	4.21	149.1325	0.99	3.5	$C_{11}H_{17}$
150.0673	243569.0	6.61	150.0675	-0.27	5.0	$C_9H_{10}O_2$
150.1036	324305.0	8.80	150.1039	-0.36	4.0	$C_{10}H_{14}O_1$
151.0748	483545.0	13.12	151.0754	-0.51	4.5	$C_9H_{11}O_2$
151.1114	304623.0	8.27	151.1117	-0.30	3.5	$C_{10}H_{15}O_1$
151.1485	99836.0	2.71	151.1481	0.34	2.5	$C_{11}H_{19}$
152.0618	161149.0	4.37	152.0621	-0.24	9.0	$C_{12}H_8$
152.0820	130525.0	3.54	152.0832	-1.14	4.0	$C_9H_{12}O_2$
153.0710	170634.0	4.63	153.0699	1.12	8.5	$C_{12}H_9$
153.0901	90221.0	2.45	153.0910	-0.89	3.5	$C_9H_{13}O_2$
153.1637	63352.0	1.72	153.1638	-0.05	1.5	$C_{11}H_{21}$
154.0768	98994.0	2.69	154.0777	-0.89	8.0	$C_{12}H_{10}$
155.0856	241076.0	6.54	155.0855	0.09	7.5	$C_{12}H_{11}$
155.1791	60309.0	1.64	155.1794	-0.28	0.5	$C_{11}H_{23}$
156.0934	178403.0	4.84	156.0934	0.09	7.0	$C_{12}H_{12}$
157.1016	582410.0	15.81	157.1012	0.39	6.5	$C_{12}H_{13}$

EIH-2019040307-GYW-LSQ_A8-L3-DEE2-c1#1 RT: 4.75
T: + c EI Full ms [49.50-800.50]
m/z= 88-314

m/z	Intensity	Relative	Theo. Mass	Delta (mmu)	RDB equiv.	Composition
158.0705	93782.0	2.55	158.0726	-2.12	7.0	$C_{11}H_{10}O_1$
158.1068	332981.0	9.04	158.1090	-2.17	6.0	$C_{12}H_{14}$
159.0789	356904.0	9.69	159.0804	-1.50	6.5	$C_{11}H_{11}O_1$
159.1158	871495.0	23.66	159.1168	-1.02	5.5	$C_{12}H_{15}$
160.0859	216765.0	5.88	160.0883	-2.34	6.0	$C_{11}H_{12}O_1$
160.1218	247745.0	6.72	160.1247	-2.87	5.0	$C_{12}H_{16}$
161.0610	61118.0	1.66	161.0597	1.25	6.5	$C_{10}H_9O_2$
161.0952	544405.0	14.78	161.0961	-0.94	5.5	$C_{11}H_{13}O_1$
161.1314	450816.0	12.24	161.1325	-1.05	4.5	$C_{12}H_{17}$
162.1028	206697.0	5.61	162.1039	-1.14	5.0	$C_{11}H_{14}O_1$
163.0733	177432.0	4.82	163.0754	-2.04	5.5	$C_{10}H_{11}O_2$
163.1107	362958.0	9.85	163.1117	-1.08	4.5	$C_{11}H_{15}O_1$
163.1478	153962.0	4.18	163.1481	-0.35	3.5	$C_{12}H_{19}$
164.0826	466193.0	12.65	164.0832	-0.62	5.0	$C_{10}H_{12}O_2$
164.1175	213560.0	5.80	164.1196	-2.04	4.0	$C_{11}H_{16}O_1$
165.0698	176299.0	4.79	165.0699	-0.08	9.5	$C_{13}H_9$
165.0904	286592.0	7.78	165.0910	-0.59	4.5	$C_{10}H_{13}O_2$
165.1244	75848.0	2.06	165.1274	-2.94	3.5	$C_{11}H_{17}O_1$
165.1626	66686.0	1.81	165.1638	-1.16	2.5	$C_{12}H_{21}$
166.0756	64518.0	1.75	166.0777	-2.15	9.0	$C_{13}H_{10}$
166.0973	80574.0	2.19	166.0988	-1.52	4.0	$C_{10}H_{14}O_2$
167.0855	149074.0	4.05	167.0855	0.01	8.5	$C_{13}H_{11}$
169.1007	251112.0	6.82	169.1012	-0.51	7.5	$C_{13}H_{13}$
170.1068	116022.0	3.15	170.1090	-2.23	7.0	$C_{13}H_{14}$
171.0816	135154.0	3.67	171.0804	1.13	7.5	$C_{12}H_{11}O_1$
171.1172	439065.0	11.92	171.1168	0.35	6.5	$C_{13}H_{15}$
172.0869	116410.0	3.16	172.0883	-1.39	7.0	$C_{12}H_{12}O_1$
172.1227	132402.0	3.59	172.1247	-1.91	6.0	$C_{13}H_{16}$
173.0965	320842.0	8.71	173.0961	0.37	6.5	$C_{12}H_{13}O_1$
173.1314	282836.0	7.68	173.1325	-1.10	5.5	$C_{13}H_{17}$
174.1029	176720.0	4.80	174.1039	-1.06	6.0	$C_{12}H_{14}O_1$
175.0778	67366.0	1.83	175.0754	2.41	6.5	$C_{11}H_{11}O_2$
175.1115	388014.0	10.53	175.1117	-0.20	5.5	$C_{12}H_{15}O_1$
175.1484	165616.0	4.50	175.1481	0.27	4.5	$C_{13}H_{19}$
176.0828	71639.0	1.94	176.0832	-0.40	6.0	$C_{11}H_{12}O_2$
176.1179	172997.0	4.70	176.1196	-1.63	5.0	$C_{12}H_{16}O_1$
177.0924	140269.0	3.81	177.0910	1.42	5.5	$C_{11}H_{13}O_2$
177.1272	184360.0	5.00	177.1274	-0.19	4.5	$C_{12}H_{17}O_1$
177.1635	140042.0	3.80	177.1638	-0.23	3.5	$C_{13}H_{21}$
178.0798	70409.0	1.91	178.0777	2.07	10.0	$C_{14}H_{10}$
178.1001	56586.0	1.54	178.0988	1.30	5.0	$C_{11}H_{14}O_2$
179.0837	129586.0	3.52	179.0855	-1.81	9.5	$C_{14}H_{11}$
179.1069	111684.0	3.03	179.1067	0.21	4.5	$C_{11}H_{15}O_2$
179.1439	103688.0	2.81	179.1430	0.81	3.5	$C_{12}H_{19}O_1$
181.1018	115763.0	3.14	181.1012	0.66	8.5	$C_{14}H_{13}$
182.1079	66071.0	1.79	182.1090	-1.10	8.0	$C_{14}H_{14}$
183.0808	69535.0	1.89	183.0804	0.40	8.5	$C_{13}H_{11}O_1$
183.1171	167461.0	4.55	183.1168	0.30	7.5	$C_{14}H_{15}$
184.1246	66136.0	1.80	184.1247	-0.10	7.0	$C_{14}H_{16}$
185.0966	174778.0	4.74	185.0961	0.52	7.5	$C_{13}H_{13}O_1$
185.1312	266068.0	7.22	185.1325	-1.23	6.5	$C_{14}H_{17}$
186.1033	115180.0	3.13	186.1039	-0.60	7.0	$C_{13}H_{14}O_1$
186.1389	177626.0	4.82	186.1403	-1.37	6.0	$C_{14}H_{18}$
187.1122	248425.0	6.74	187.1117	0.47	6.5	$C_{13}H_{15}O_1$
187.1483	669556.0	18.17	187.1481	0.21	5.5	$C_{14}H_{19}$

EIH-2019040307-GYW-LSQ_A8-L3-DEE2-c1#1 RT: 4.75
T: + c EI Full ms [49.50-800.50]
m/z= 88-314

m/z	Intensity	Relative	Theo. Mass	Delta (mmu)	RDB equiv.	Composition
188.1173	109224.0	2.96	188.1196	-2.30	6.0	$C_{13}H_{16}O_1$
188.1536	161861.0	4.39	188.1560	-2.36	5.0	$C_{14}H_{20}$
189.0697	61377.0	1.67	189.0699	-0.23	11.5	$C_{15}H_9$
189.0891	116151.0	3.15	189.0910	-1.95	6.5	$C_{12}H_{13}O_2$
189.1267	235961.0	6.40	189.1274	-0.69	5.5	$C_{13}H_{17}O_1$
189.1633	75395.0	2.05	189.1638	-0.51	4.5	$C_{14}H_{21}$
190.0965	62251.0	1.69	190.0988	-2.36	6.0	$C_{12}H_{14}O_2$
190.1335	108803.0	2.95	190.1352	-1.76	5.0	$C_{13}H_{18}O_1$
191.1079	102264.0	2.78	191.1067	1.27	5.5	$C_{12}H_{15}O_2$
191.1429	100969.0	2.74	191.1430	-0.15	4.5	$C_{13}H_{19}O_1$
191.1790	50436.0	1.37	191.1794	-0.45	3.5	$C_{14}H_{23}$
192.1122	61701.0	1.67	192.1145	-2.33	5.0	$C_{12}H_{16}O_2$
193.1016	62705.0	1.70	193.1012	0.38	9.5	$C_{15}H_{13}$
193.1229	55486.0	1.51	193.1223	0.62	4.5	$C_{12}H_{17}O_2$
195.1149	95595.0	2.59	195.1168	-1.94	8.5	$C_{15}H_{15}$
196.1236	59888.0	1.63	196.1247	-1.04	8.0	$C_{15}H_{16}$
197.0948	84070.0	2.28	197.0961	-1.28	8.5	$C_{14}H_{13}O_1$
197.1316	96728.0	2.63	197.1325	-0.92	7.5	$C_{15}H_{17}$
198.1029	69179.0	1.88	198.1039	-1.06	8.0	$C_{14}H_{14}O_1$
199.1123	171508.0	4.66	199.1117	0.57	7.5	$C_{14}H_{15}O_1$
199.1468	81804.0	2.22	199.1481	-1.34	6.5	$C_{15}H_{19}$
200.1177	81869.0	2.22	200.1196	-1.84	7.0	$C_{14}H_{16}O_1$
201.0915	54482.0	1.48	201.0910	0.50	7.5	$C_{13}H_{13}O_2$
201.1262	198701.0	5.39	201.1274	-1.19	6.5	$C_{14}H_{17}O_1$
202.1337	101908.0	2.77	202.1352	-1.48	6.0	$C_{14}H_{18}O_1$
203.1060	99156.0	2.69	203.1067	-0.68	6.5	$C_{13}H_{15}O_2$
203.1421	200999.0	5.46	203.1430	-0.97	5.5	$C_{14}H_{19}O_1$
204.1502	185914.0	5.05	204.1509	-0.71	5.0	$C_{14}H_{20}O_1$
205.1233	70377.0	1.91	205.1223	1.03	5.5	$C_{13}H_{17}O_2$
205.1583	290735.0	7.89	205.1587	-0.38	4.5	$C_{14}H_{21}O_1$
207.1168	56133.0	1.52	207.1168	-0.06	9.5	$C_{16}H_{15}$
207.1371	82419.0	2.24	207.1380	-0.81	4.5	$C_{13}H_{19}O_2$
209.0956	59273.0	1.61	209.0961	-0.48	9.5	$C_{15}H_{13}O_1$
209.1309	66266.0	1.80	209.1325	-1.58	8.5	$C_{16}H_{17}$
211.1114	101098.0	2.74	211.1117	-0.29	8.5	$C_{15}H_{15}O_1$
211.1485	64000.0	1.74	211.1481	0.33	7.5	$C_{16}H_{19}$
212.1203	51277.0	1.39	212.1196	0.77	8.0	$C_{15}H_{16}O_1$
213.1262	124536.0	3.38	213.1274	-1.24	7.5	$C_{15}H_{17}O_1$
213.1629	87567.0	2.38	213.1638	-0.83	6.5	$C_{16}H_{21}$
214.1349	60503.0	1.64	214.1352	-0.29	7.0	$C_{15}H_{18}O_1$
215.1066	58075.0	1.58	215.1067	-0.06	7.5	$C_{14}H_{15}O_2$
215.1435	137096.0	3.72	215.1430	0.45	6.5	$C_{15}H_{19}O_1$
216.1488	51957.0	1.41	216.1509	-2.03	6.0	$C_{15}H_{20}O_1$
217.1227	97991.0	2.66	217.1223	0.38	6.5	$C_{14}H_{17}O_2$
219.1371	48946.0	1.33	219.1380	-0.88	5.5	$C_{14}H_{19}O_2$
221.1330	51828.0	1.41	221.1325	0.50	9.5	$C_{17}H_{17}$
223.1101	57557.0	1.56	223.1117	-1.65	9.5	$C_{16}H_{15}O_1$
226.1346	49756.0	1.35	226.1352	-0.60	8.0	$C_{16}H_{18}O_1$
227.1436	104756.0	2.84	227.1430	0.56	7.5	$C_{16}H_{19}O_1$
228.1496	50500.0	1.37	228.1509	-1.30	7.0	$C_{16}H_{20}O_1$
229.1208	71057.0	1.93	229.1223	-1.48	7.5	$C_{15}H_{17}O_2$
229.1569	64809.0	1.76	229.1587	-1.84	6.5	$C_{16}H_{21}O_1$
231.1388	84006.0	2.28	231.1380	0.82	6.5	$C_{15}H_{19}O_2$
231.1729	77466.0	2.10	231.1743	-1.42	5.5	$C_{16}H_{23}O_1$
233.1539	53381.0	1.45	233.1536	0.31	5.5	$C_{15}H_{21}O_2$

EIH-2019040307-GYW-LSQ_A8-L3-DEE2-c1 4/4/2019 11:17:23 AM

EIH-2019040307-GYW-LSQ_A8-L3-DEE2-c1#1 RT: 4.75
T: + c EI Full ms [49.50-800.50]
m/z= 88-314

m/z	Intensity	Relative	Theo. Mass	Delta (mmu)	RDB equiv.	Composition
241.1595	278855.0	7.57	241.1587	0.85	7.5	$C_{17}H_{21}O_1$
245.1551	86110.0	2.34	245.1536	1.49	6.5	$C_{16}H_{21}O_2$
251.1456	71121.0	1.93	251.1430	2.59	9.5	$C_{18}H_{19}O_1$
255.1747	56198.0	1.53	255.1743	0.32	7.5	$C_{18}H_{23}O_1$
259.1721	136352.0	3.70	259.1693	2.82	6.5	$C_{17}H_{23}O_2$
269.1565	73517.0	2.00	269.1536	2.89	8.5	$C_{18}H_{21}O_2$
284.1757	67075.0	1.82	284.1771	-1.35	8.0	$C_{19}H_{24}O_2$
287.1655	74164.0	2.01	287.1642	1.33	7.5	$C_{18}H_{23}O_3$
302.1883	50792.0	1.38	302.1876	0.64	7.0	$C_{19}H_{26}O_3$

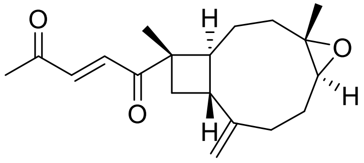

Figure S63. HREIMS spectrum of sinuhirtone B (**8**) in MeOH.

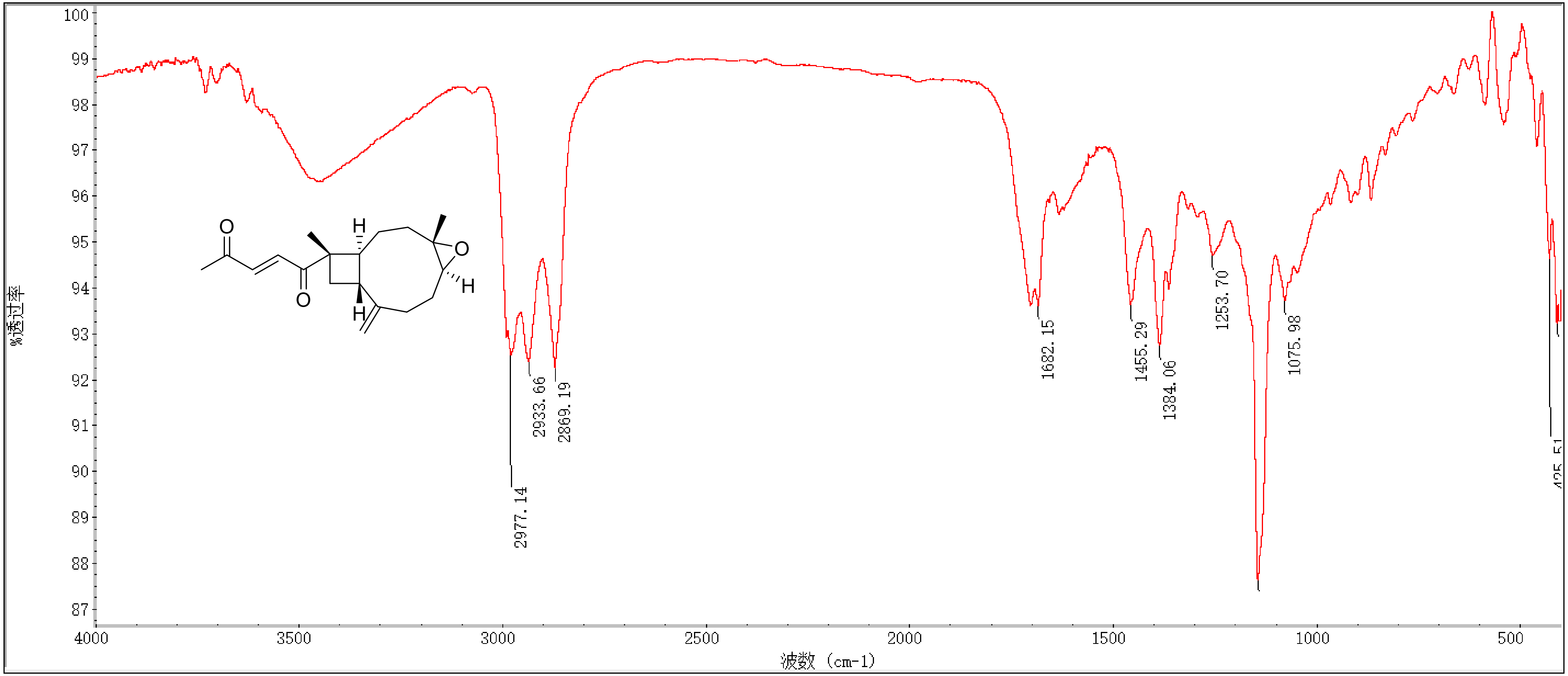

Figure S64. IR spectrum of sinuhirtone B (**8**).

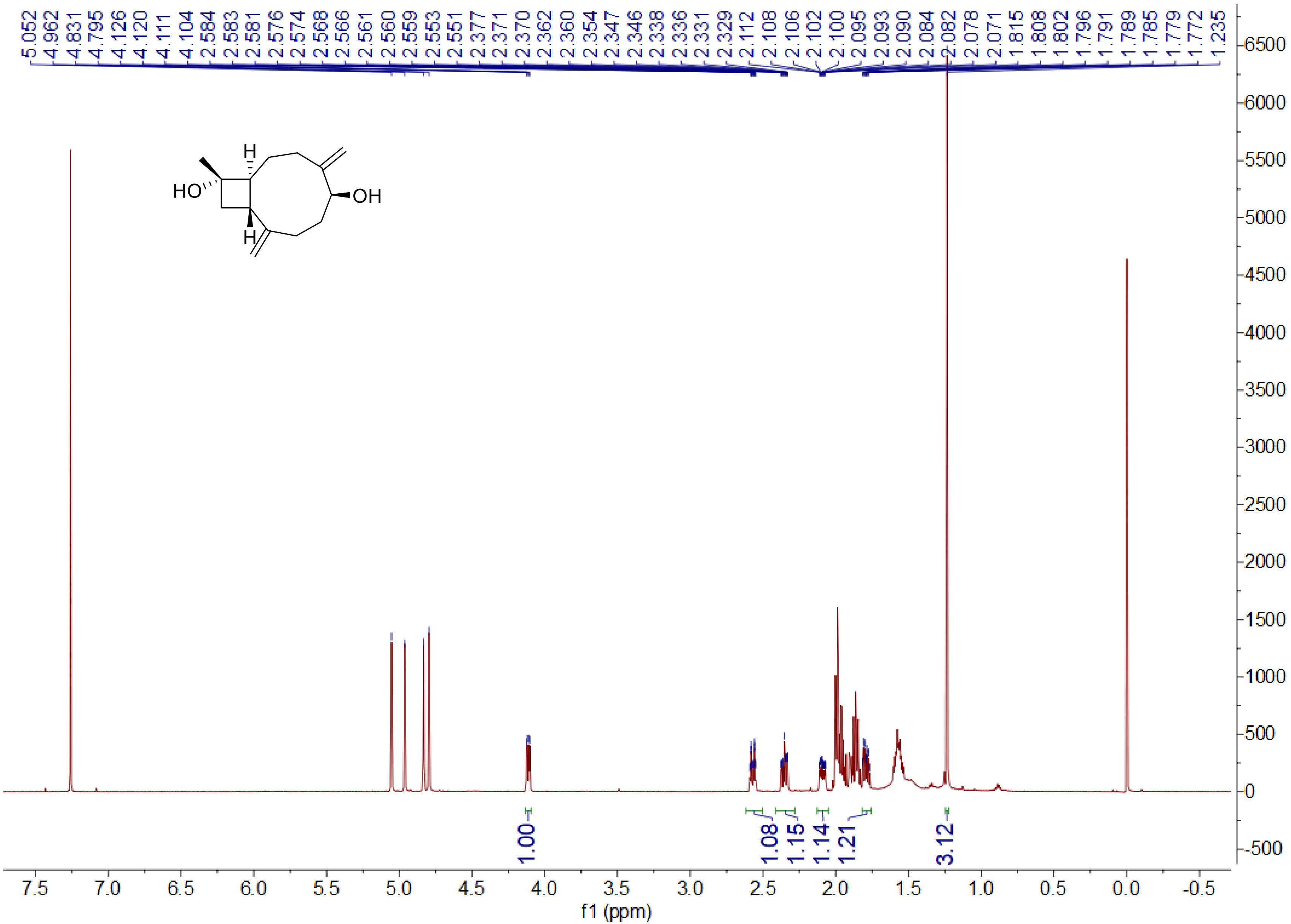

Figure S65. ^{1}H NMR spectrum (600 MHz) of sinuhirtin F (**9**) in $CDCl_3$.

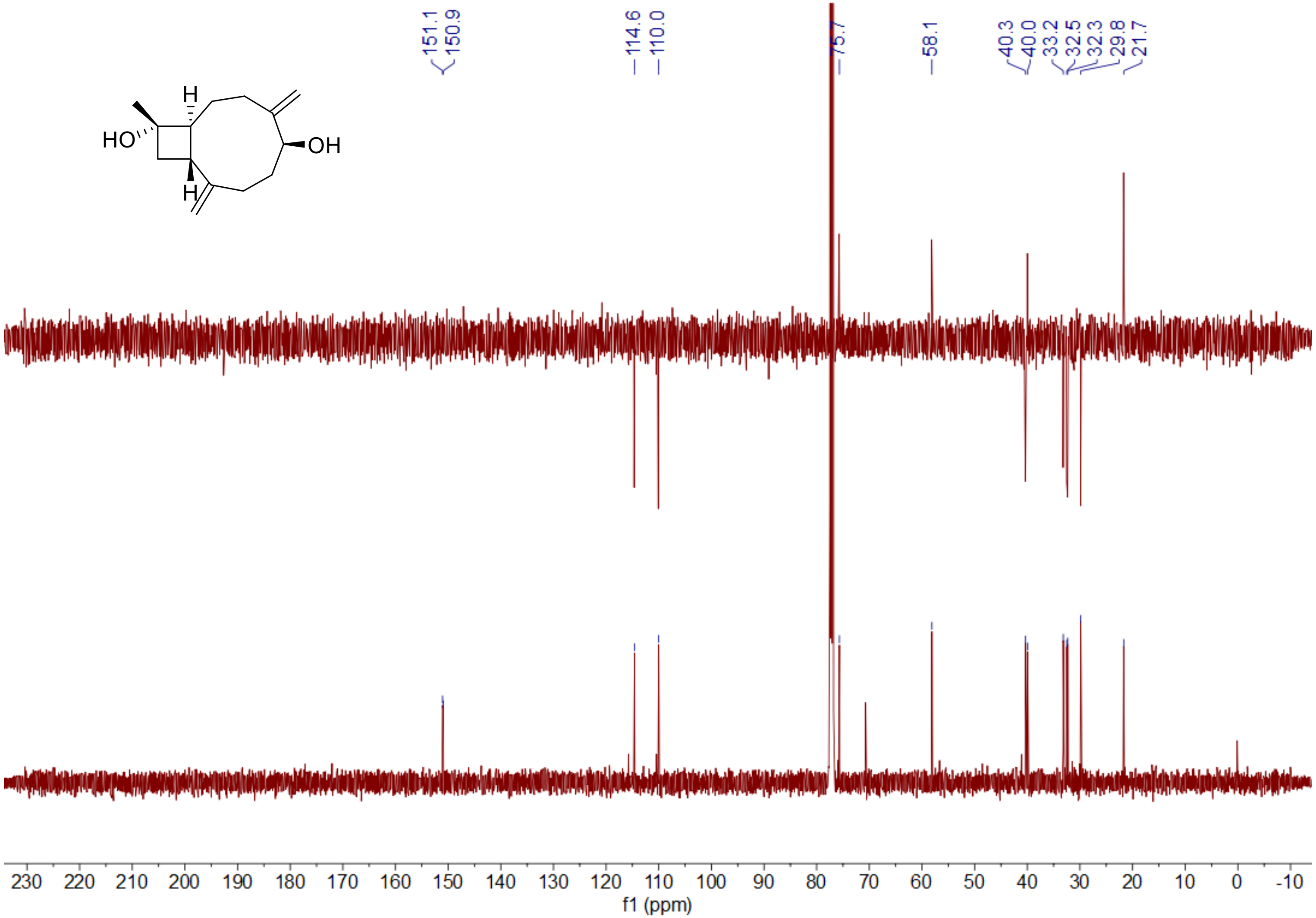

Figure S66. ^{13}C NMR spectrum (125 MHz) of sinuhirtin F (**9**) in $CDCl_3$.

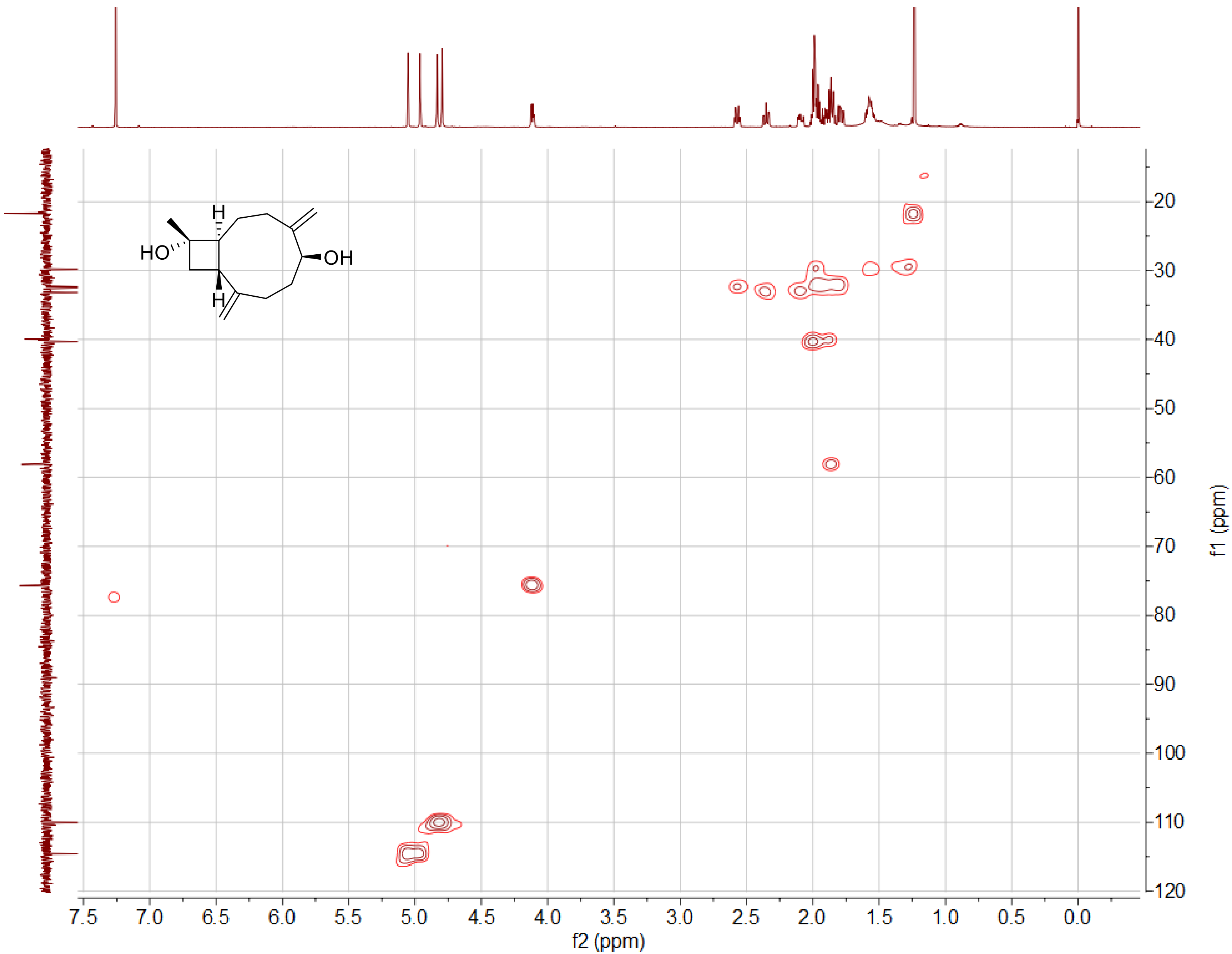

Figure S67. HSQC spectrum (500 MHz) of sinuhirtin F (**9**) in $CDCl_3$.

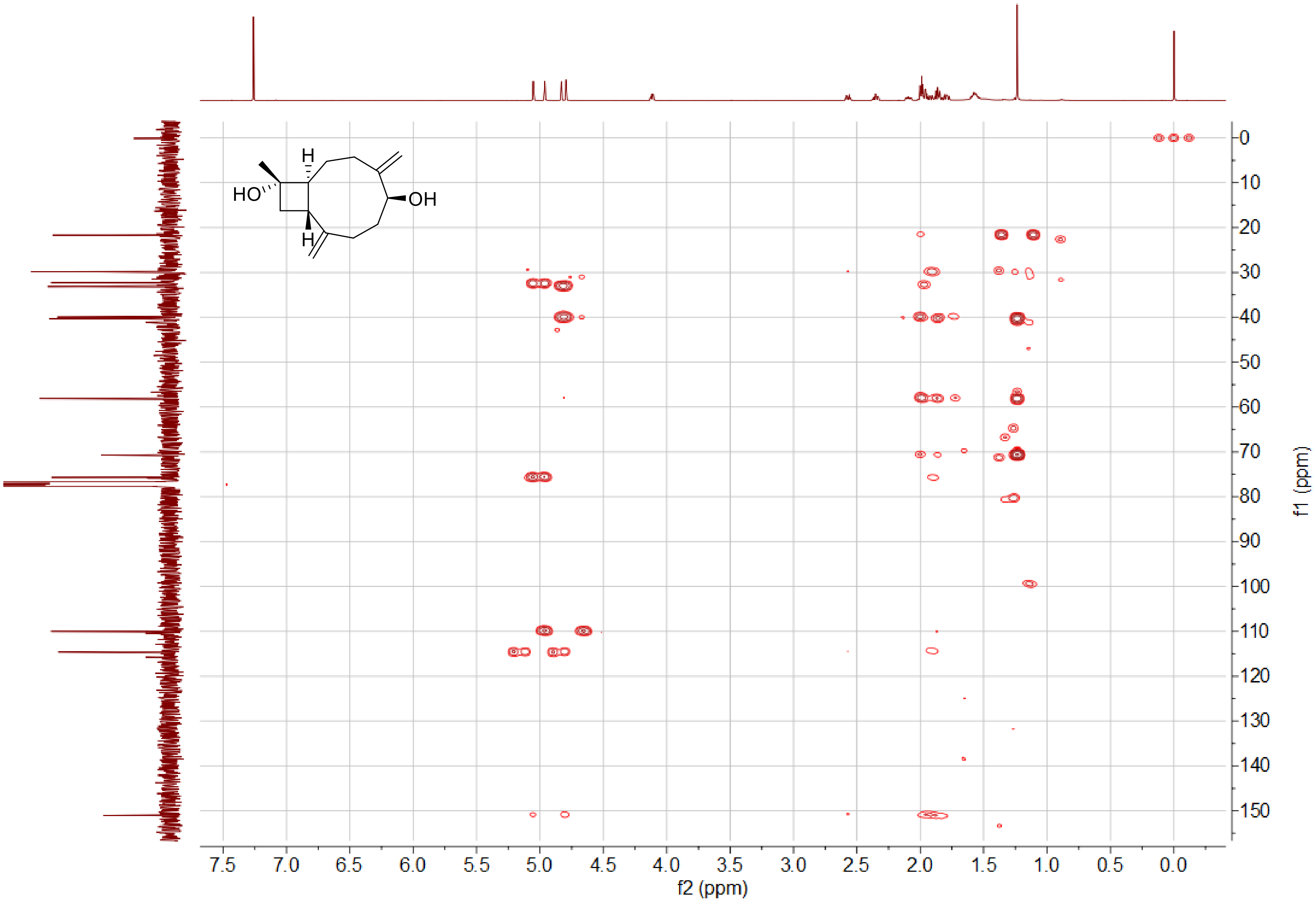

Figure S68. HMBC spectrum (500 MHz) of sinuhirtin F (**9**) in $CDCl_3$.

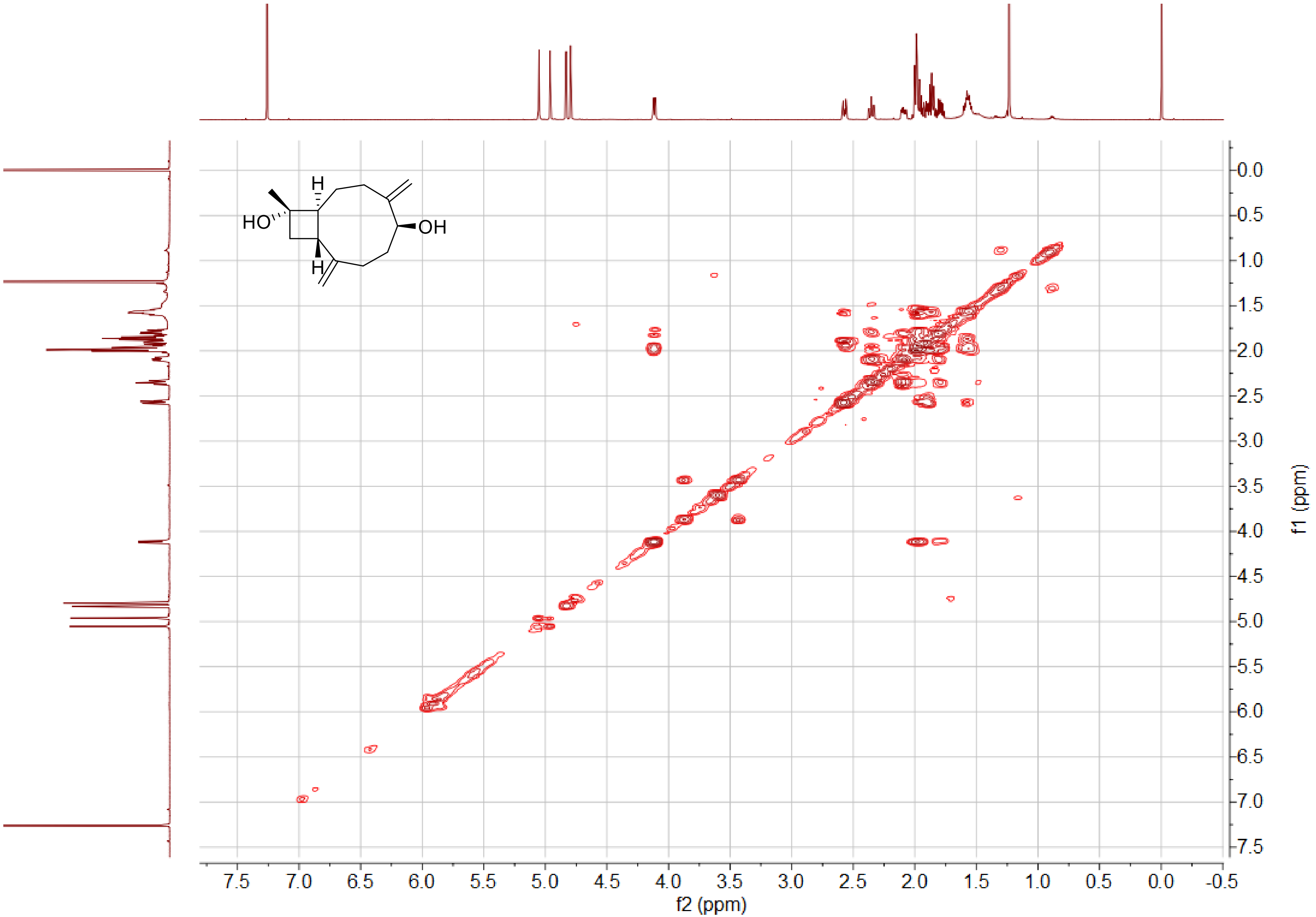

Figure S69. COSY spectrum (500 MHz) of sinuhirtin F (**9**) in $CDCl_3$.

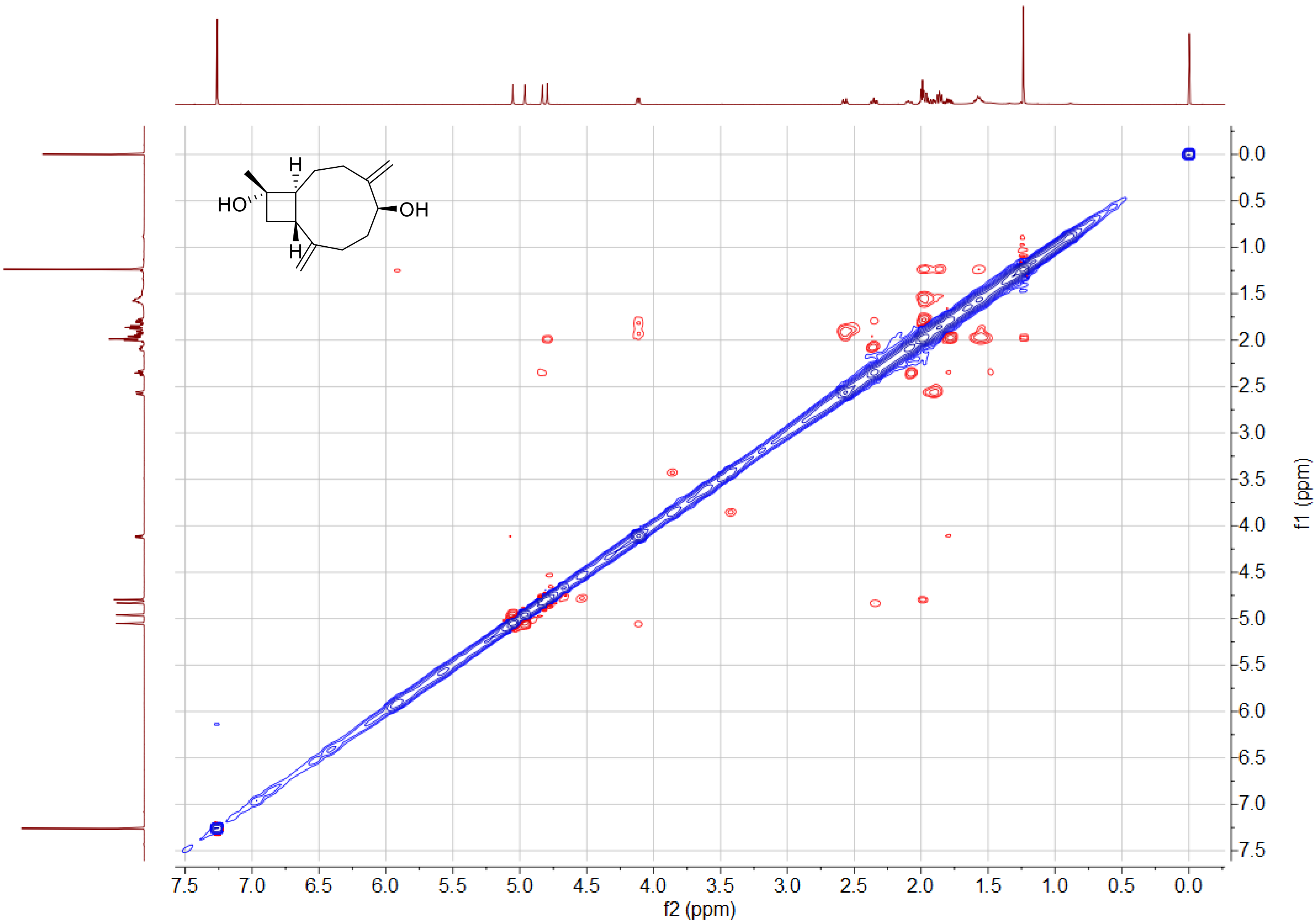

Figure S70. NOESY spectrum (500 MHz) of sinuhirtin F (**9**) in $CDCl_3$.

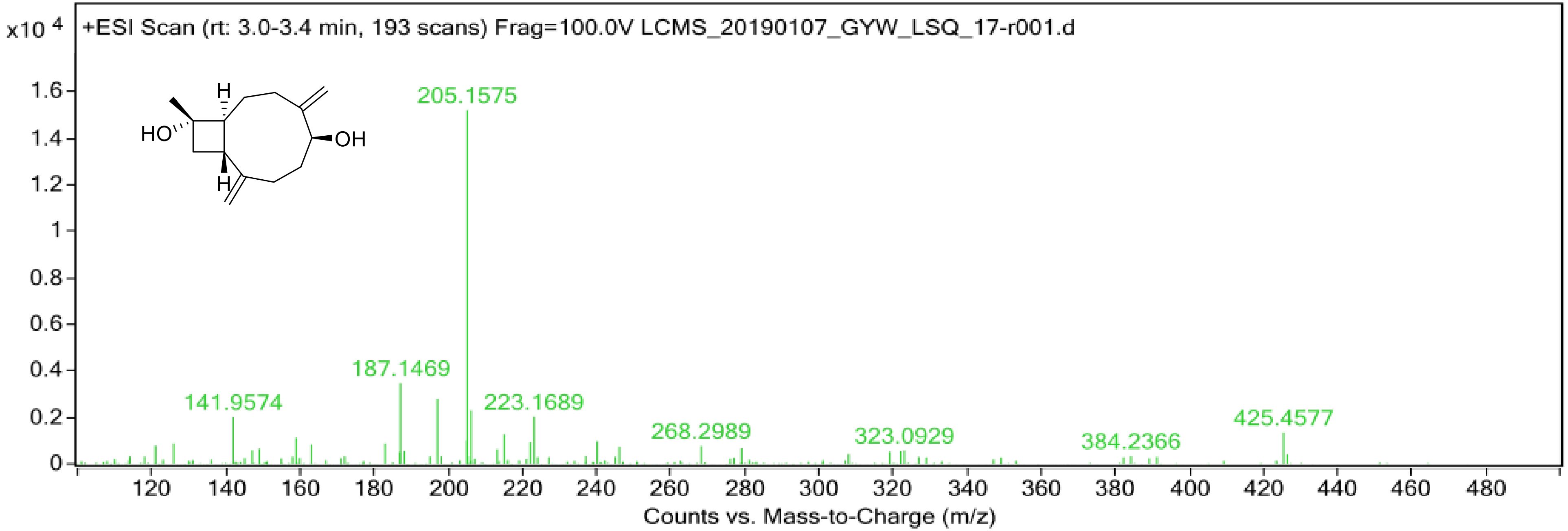

Formula Calculator Results

m/z	Calc m/z	Diff (mDa)	Diff (ppm)	Ion Formula	Ion
223.1689	223.1693	0.33	1.5	C14 H23 O2	(M+H)+

Figure S71. HRESIMS spectrum of sinuhirtin F (**9**) in MeOH.

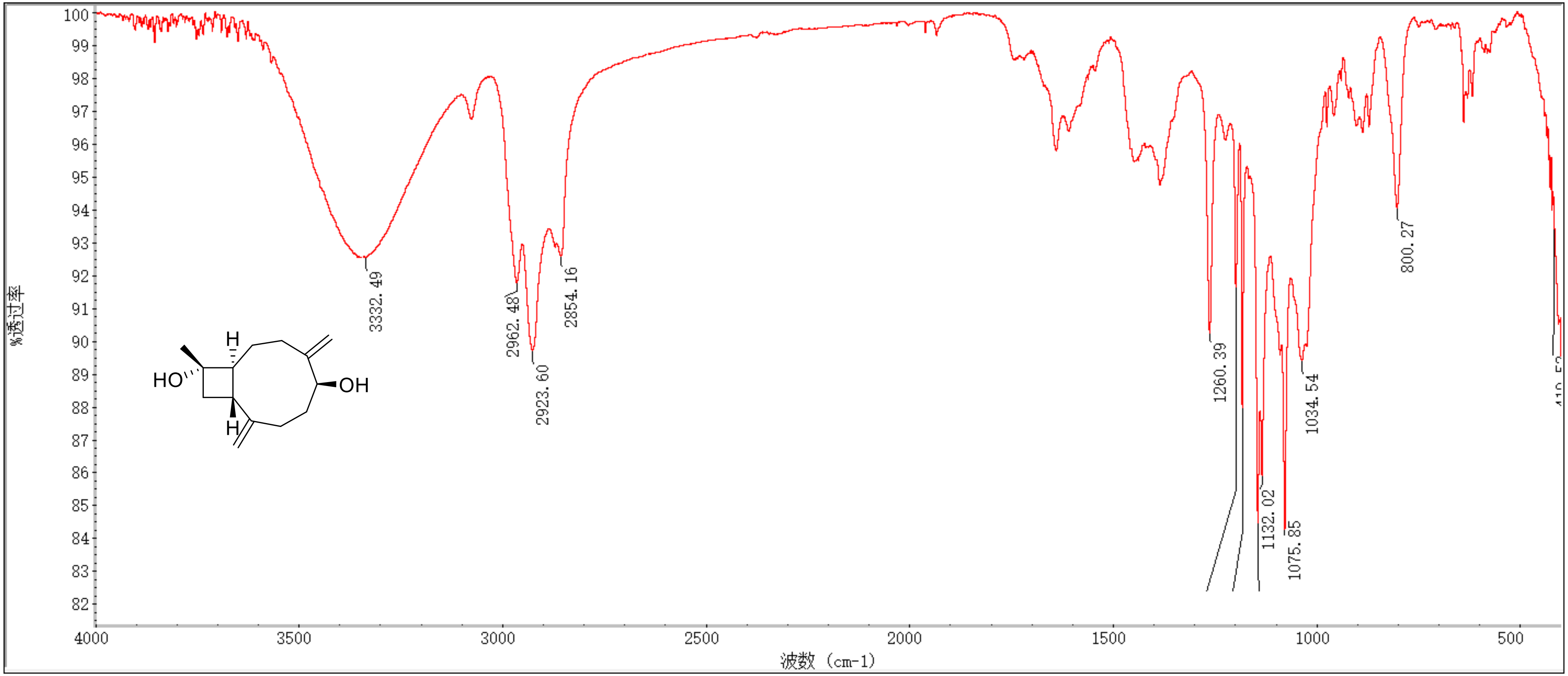

Figure S72. IR spectrum of sinuhirtin F (**9**).

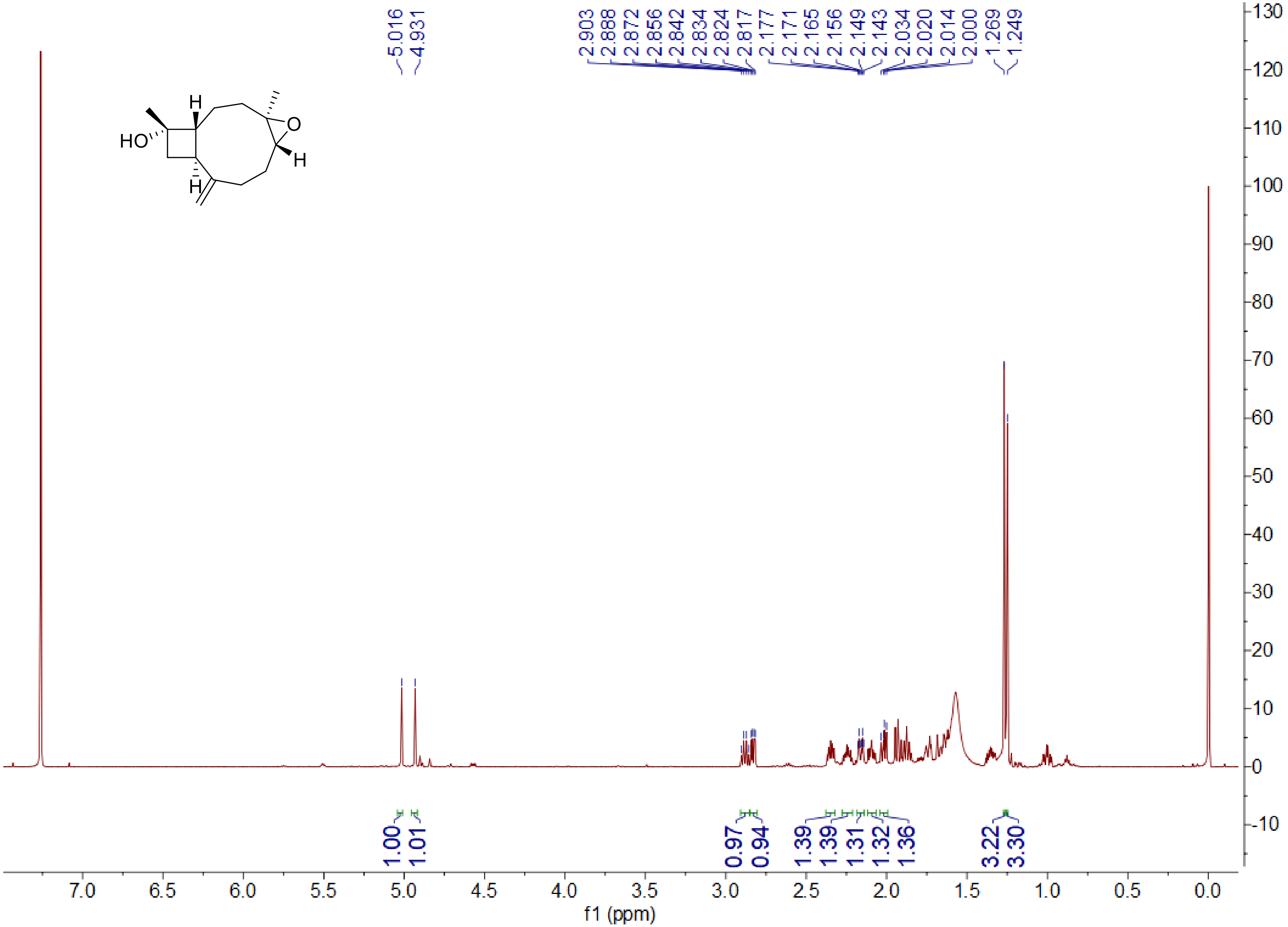

Figure S73. ^{1}H NMR spectrum (600 MHz) of sinuhirtin G (**10**) in $CDCl_3$.

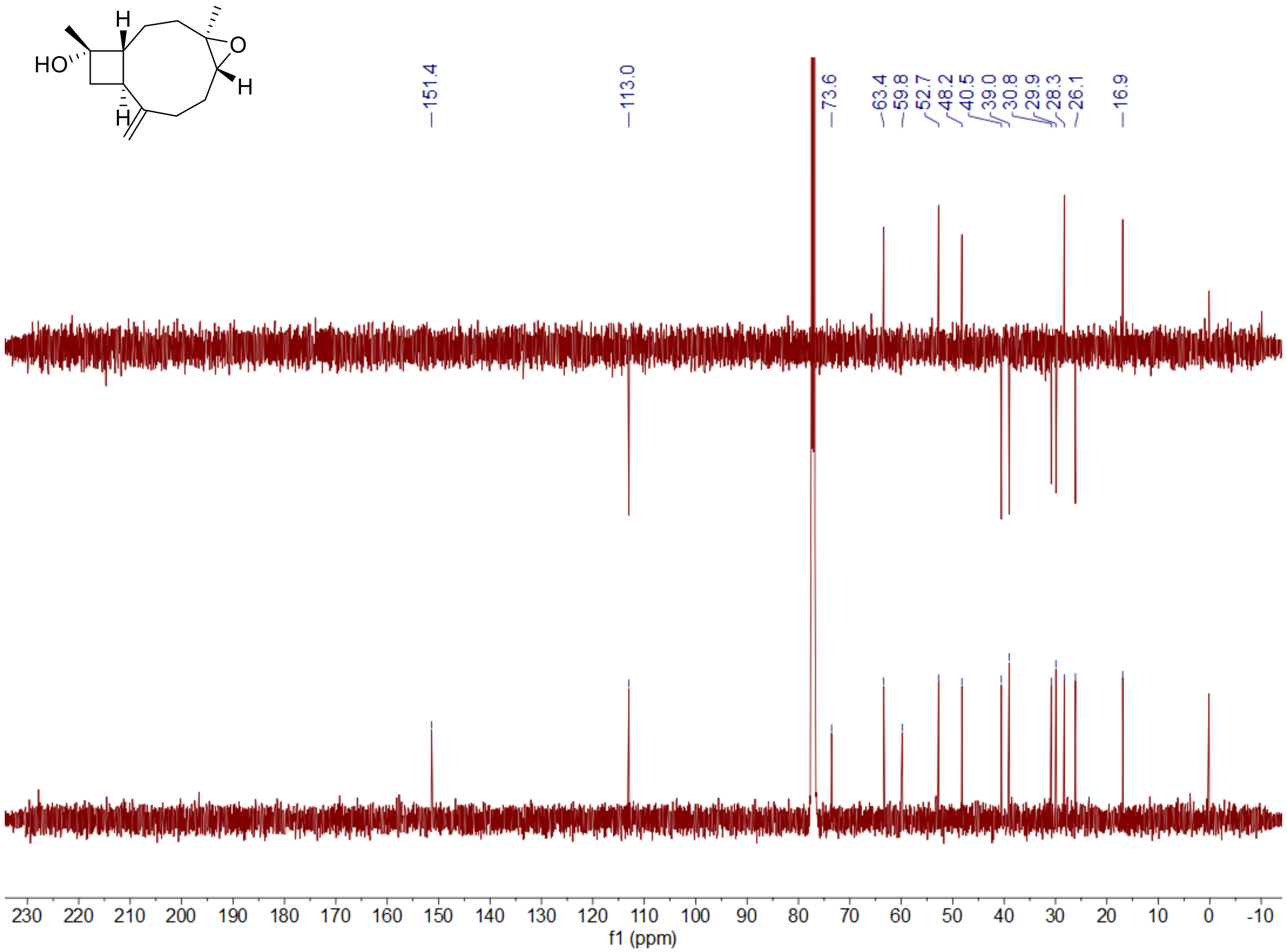

Figure S74. ^{13}C NMR spectrum (125 MHz) of sinuhirtin G (**10**) in $CDCl_3$.

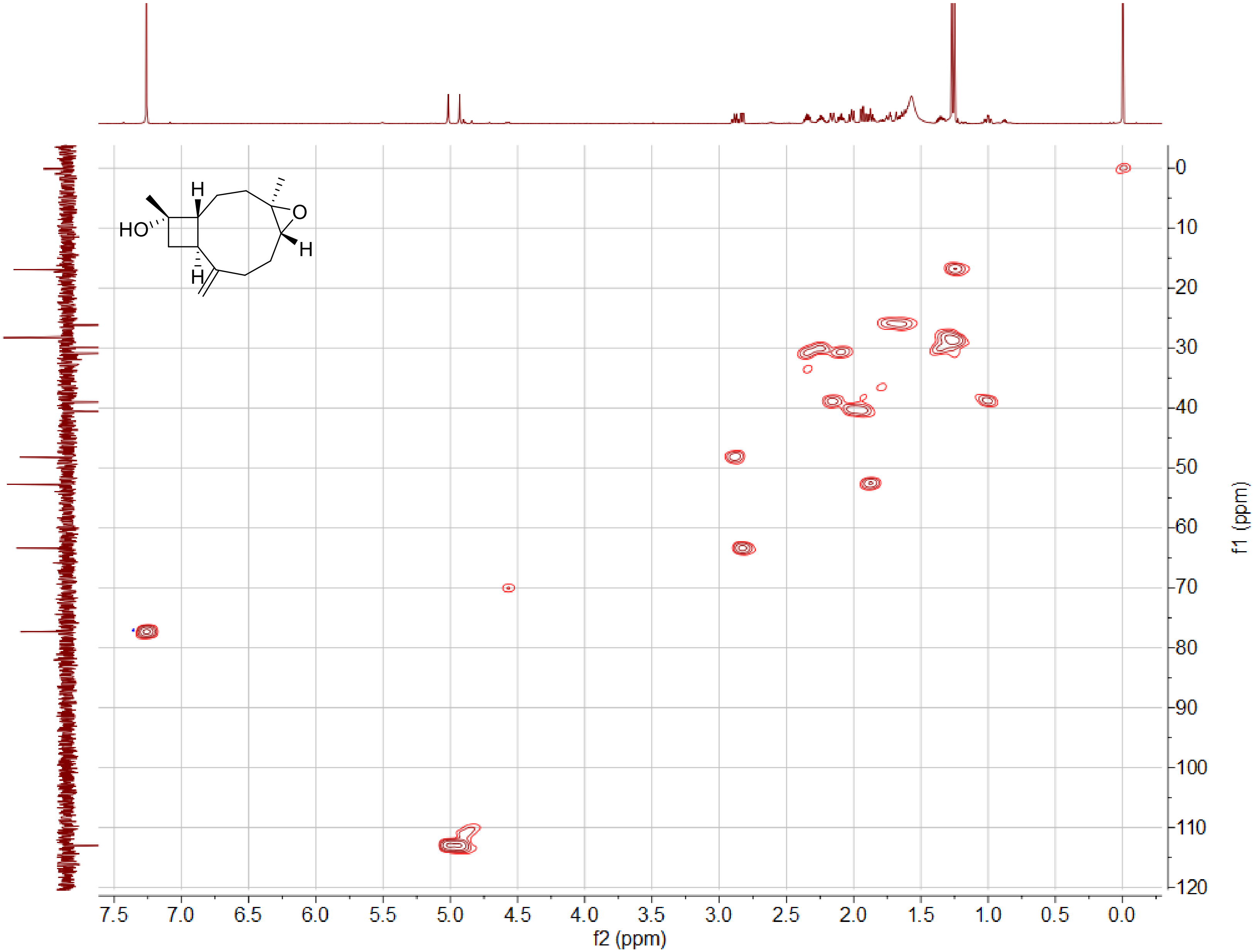

Figure S75. HSQC spectrum (500 MHz) of sinuhirtin G (**10**) in $CDCl_3$.

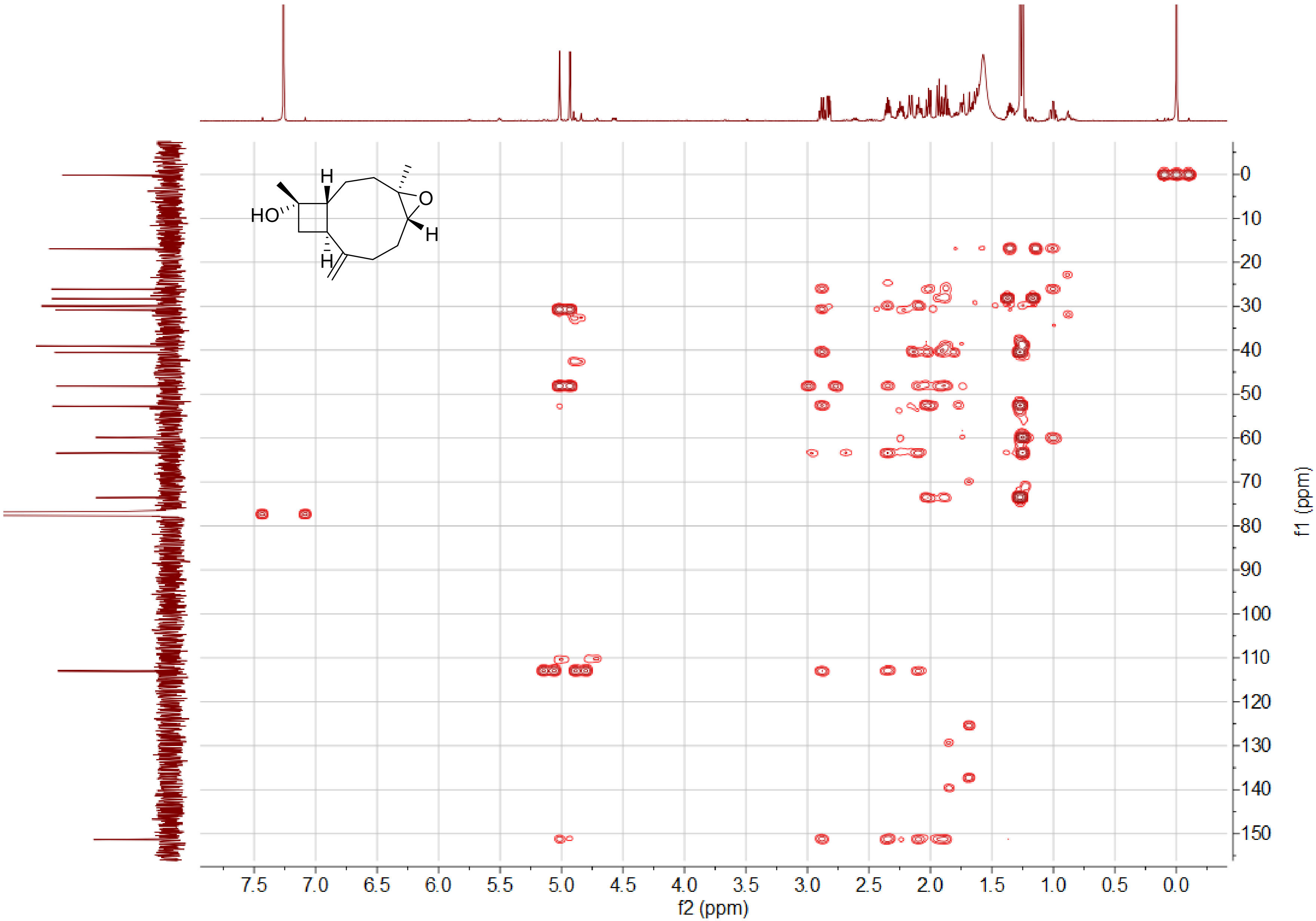

Figure S76. HMBC spectrum (500 MHz) of sinuhirtin G (**10**) in $CDCl_3$.

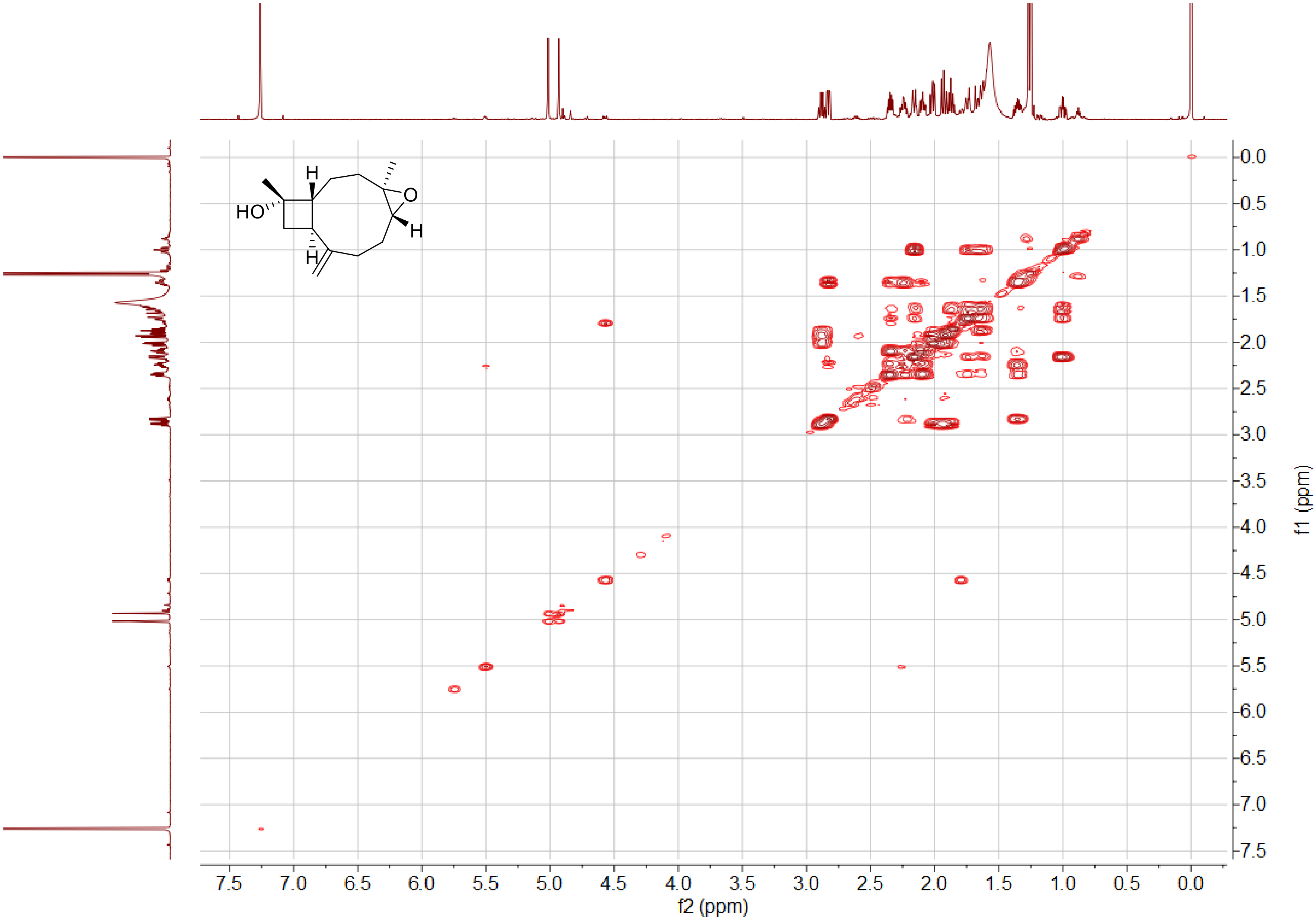

Figure S77. COSY spectrum (500 MHz) of sinuhirtin G (**10**) in $CDCl_3$.

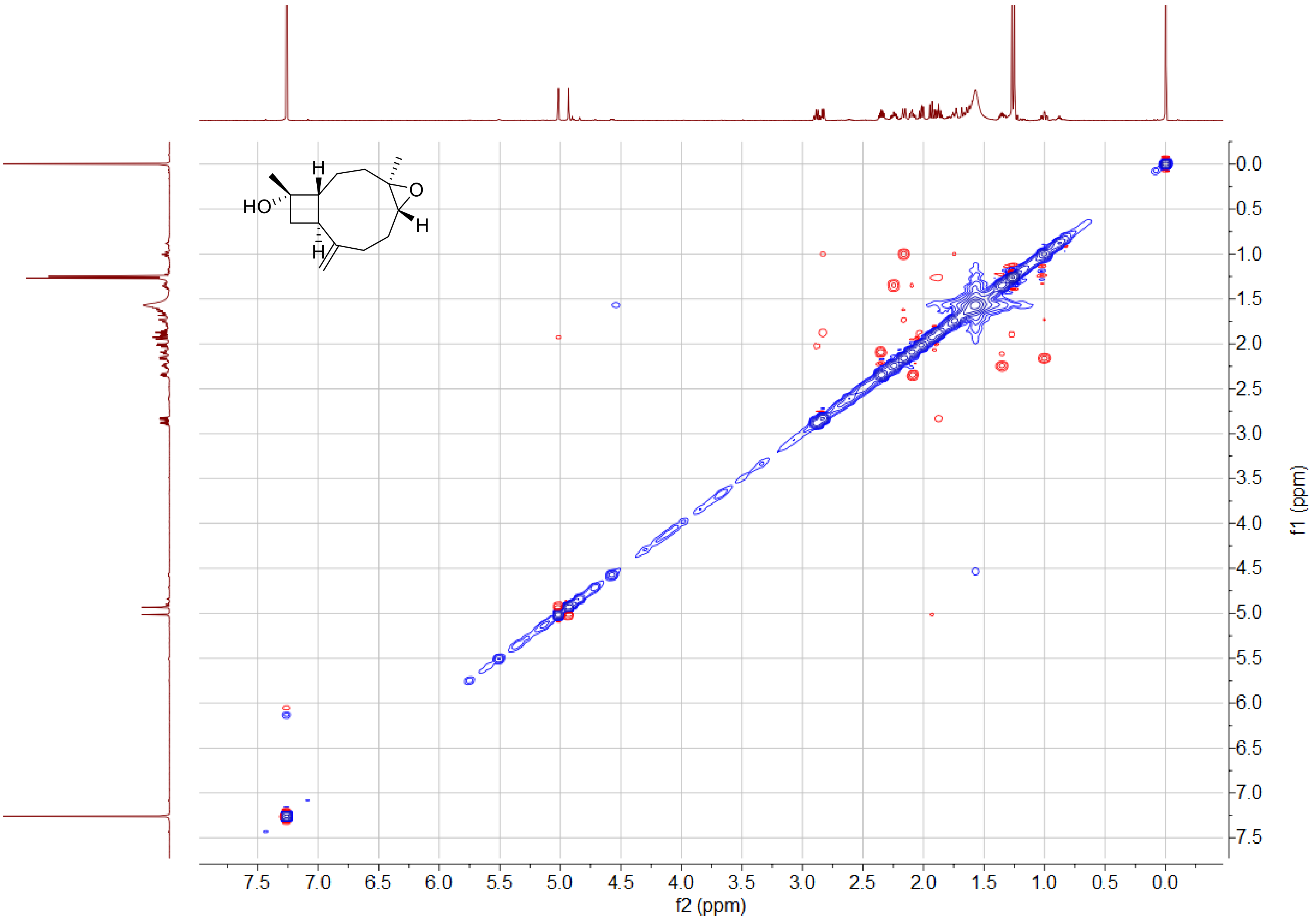

Figure S78. NOESY spectrum (500 MHz) of sinuhirtin G (**10**) in $CDCl_3$.

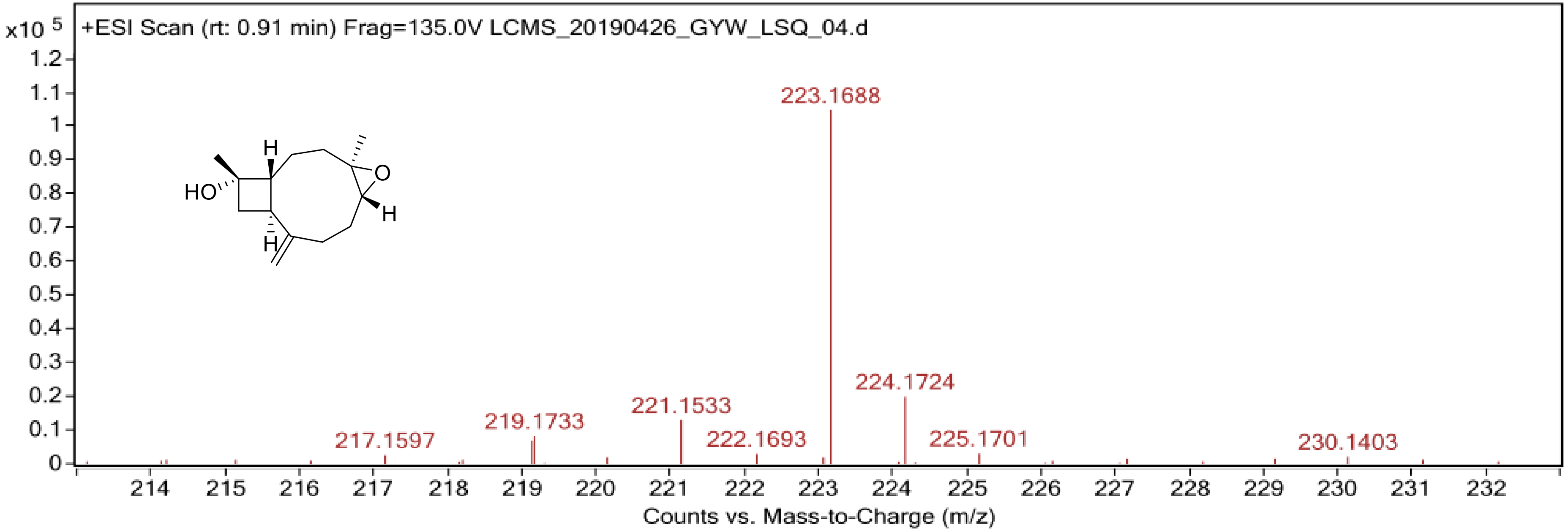

Formula Calculator Results

m/z	Calc m/z	Diff (mDa)	Diff (ppm)	Ion Formula	Ion
223.1688	223.1693	0.48	2.16	C14 H23 O2	(M+H)+

Figure S79. HRESIMS spectrum of sinuhirtin G (**10**) in MeOH.

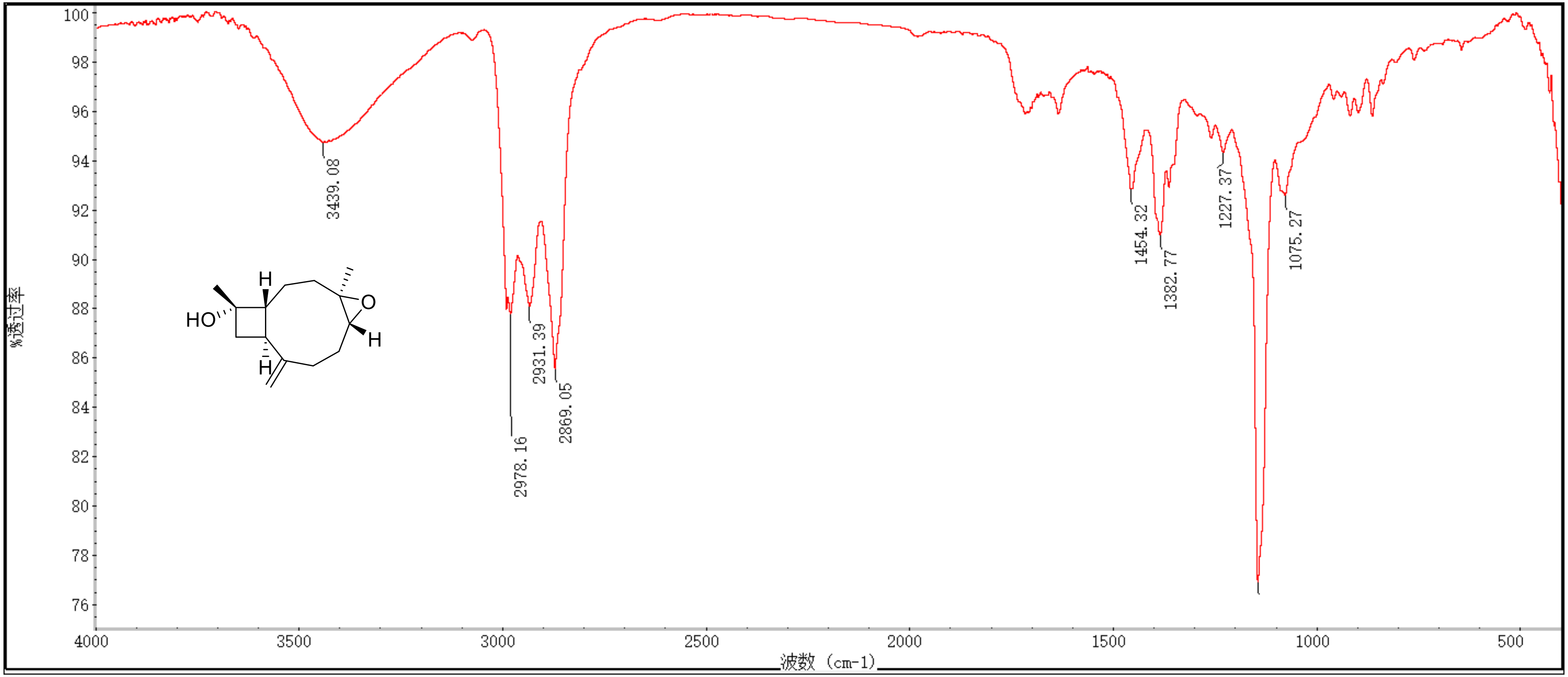

Figure S80. IR spectrum of sinuhirtin G (**10**).

2. Computational Section

2.1 QM-NMR calculation for compounds 1, 3, and 9

Theoretical calculation of all theoretical stereoisomers was carried out to determine the relative configuration of **1**, **3**, and **9**, based on the alignment of its 1D NMR chemical shifts (^{13}C NMR chemical shifts herein) and calculation-generated chemical shifts. Confab was used to search the conformational space of **1a**–**1b**, **3a**–**3b**, and **9a**–**9h**. Conformational searches were carried out using the torsional sampling (MCMM) method and OPLS_2005 force field in the Macromodel 9.9.223 software applying an energy window of 21 kJ/mol. Conformers above 1% population were re-optimized with Gaussian 09 at the B3LYP/6-311G(d,p) level with IEFPCM (Polarizable Continuum Model using the Integral Equation Formalism variant) solvent model for acetonitrile. The initial torsional sampling (MCMM) and OPLS_2005 force field conformational searches of **1a** and **1b**, afforded 18 and 11 conformers; **3a** and **3b**, afforded 102 and 113 conformers; **9a**–**9h**, afforded 87, 69, 90, 69, 76, 102, 77 and 65 conformers within the 21 kJ/mol energy window, respectively. The Boltzmann populations of the conformers were obtained based on the potential energy provided by the OPLS_2005 force field, leading to 6 and 4 conformers for **1a** and **1b**; 14 and 14 conformers for **3a** and **3b**; 13, 16, 18, 9, 8, 18, 11 and 14 conformers for **9a**–**9h**; above 1% population for further re-optimization, respectively. The obtained conformers were subjected to optimization and frequency calculations on B3LYP/6-311G(d,p) (CH_3CN) level of theory. GIAO DFT ^{13}C NMR calculations were calculated on mPW1PW91/6-31G* ($CHCl_3$) level of theory, and the calculated shielding tensors were Boltzmann averaged according to Gibbs free energy and then converted into chemical shifts following MSTD protocol. The experimental ^{13}C NMR data of **1**, **3**, and **9** were compared with the calculated NMR chemical shifts of **1a**–**1b**, **3a**–**3b** and **9a**–**9h** using the mean absolute error (MAE) values, maximum deviation (MD) values, correlation coefficient (R^2), and DP4+ probability analysis.

2.1.1 QM-NMR calculation for compound 1

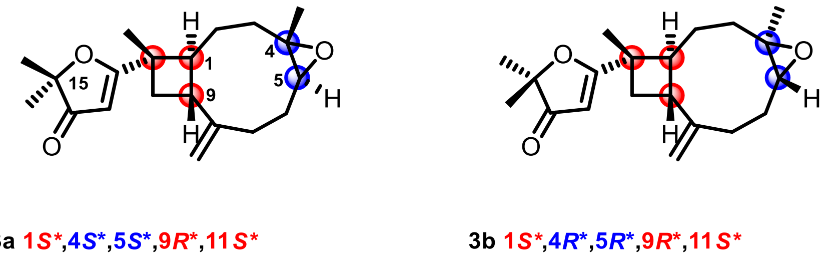

Figure S81. Structures of two stereoisomers **1a** and **1b**.

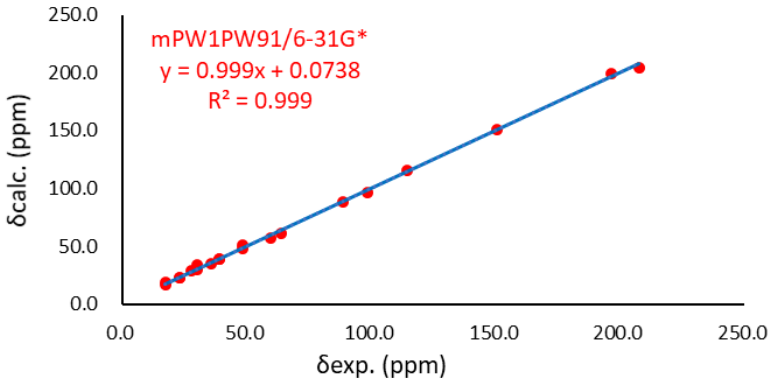

Figure S82. Regression analysis of experimental *versus* calculated ^{13}C NMR chemical shifts at mPW1PW91/6-31G* level using GIAO method of **1a**; linear fitting is shown as a line (blue).

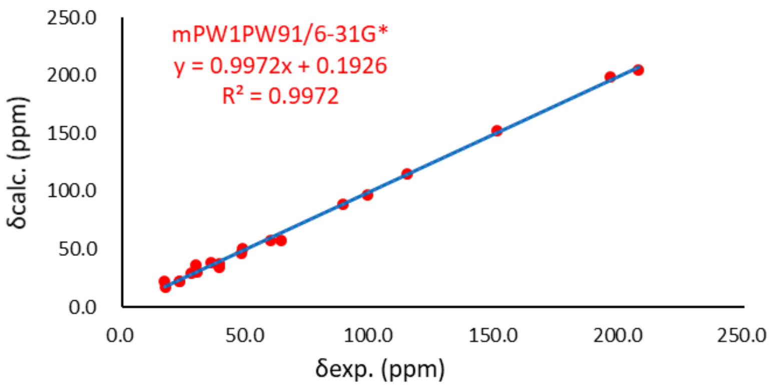

Figure S83. Regression analysis of experimental *versus* calculated ^{13}C NMR chemical shifts at mPW1PW91/6-31G* level using GIAO method of **1b**; linear fitting is shown as a line (blue).

Table S1. Experiment and calculated ^{13}C NMR chemical shifts of stereoisomers **1a** and **1b**.

No.	$\delta_{exp.}$	$\delta_{calc.}$	
		**1a**	**1b**
11	39.0	38.6	37.7
10	35.7	34.7	38.0
7	29.8	33.5	36.7
8	150.5	151.2	152.7
6	30.1	30.4	29.9
5	63.8	60.7	57.7
4	59.5	56.7	58.0
3	38.9	38.9	34.4
2	27.7	28.4	29.0
1	48.4	51.1	50.7
9	48.1	48.0	46.5
19	114.5	115.8	115.3
18	17.4	18.5	17.1
12	196.2	199.3	21.9
20	17.1	16.3	88.4
15	88.7	88.8	204.7
14	207.6	204.7	96.5
13	98.6	96.8	199.1
16	23.0	22.6	21.8
17	23.1	22.8	21.9

Table S2. Mean absolute error (MAE) values, maximum deviation (MD) values and correlation coefficient (R^2) analysis of four stereoisomers **1a** and **1b**.

Stereoisomers	MAE (ppm)	MD (ppm)	R^2
**1a**	1.4	3.1	0.999
**1b**	2.3	6.7	0.9972

Functional		Solvent?	Basis Set		Type of Data	
mPW1PW91		PCM	6-31G(d)		Shielding Tensors	
		DP4+	100.00%	0.00%	-	-	-
Nuclei	sp2?	xperimenta	Isomer 1	Isomer 2	Isomer 3	Isomer 4	Isomer 5
C		39.0	155.7890234	155.8947981			
C		35.7	159.4773185	155.6218254			
C		29.8	160.6518084	156.802516			
C	x	150.5	48.58205372	46.87059461			
C		30.1	163.5677837	163.2955573			
C		63.8	134.6771688	136.9097012			
C		59.5	138.5045627	136.6262688			
C		38.9	155.5153541	158.9625487			
C		27.7	165.4427611	164.1392787			
C		48.4	143.8090933	143.5606785			
C		48.1	146.8507098	147.5338904			
C	x	114.5	82.2693521	82.32887264			
C		17.4	174.9278456	175.3975325			
C	x	196.2	2.778756238	170.8593029			
C		17.1	176.9816878	107.8556727			
C		88.7	107.9445947	-2.423343618			
C	x	207.6	-2.405743843	100.1222371			
C		98.6	100.342037	2.905601513			
C		23.0	170.9690467	170.94724			
C		23.1	170.7992566	170.8665255			

Functional	Solvent?	Basis Set	Type of Data			
mPW1PW91	PCM	6-31G(d)	Shielding Tensors			
	Isomer 1	Isomer 2	Isomer 3	Isomer 4	Isomer 5	Isomer 6
sDP4+ (H data)	-	-	-	-	-	-
sDP4+ (C data)	100.00%	0.00%	-	-	-	-
sDP4+ (all data)	100.00%	0.00%	-	-	-	-
uDP4+ (H data)	-	-	-	-	-	-
uDP4+ (C data)	100.00%	0.00%	-	-	-	-
uDP4+ (all data)	100.00%	0.00%	-	-	-	-
DP4+ (H data)	-	-	-	-	-	-
DP4+ (C data)	100.00%	0.00%	-	-	-	-
DP4+ (all data)	100.00%	0.00%	-	-	-	-

Figure S84. DP4+ evaluation of theoretical and experimental data of **1**.

2.1.2 QM-NMR calculation for compound 3

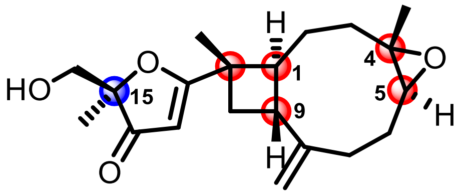

3a 1*S**,4*S**,5*S**,9*R**,11*S**,15*R**

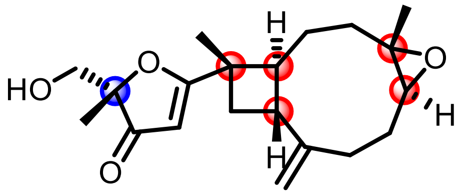

3b 1*S**,4*S**,5*S**,9*R**,11*S**,15*S**

Figure S85. Structures of two stereoisomers **3a** and **3b**.

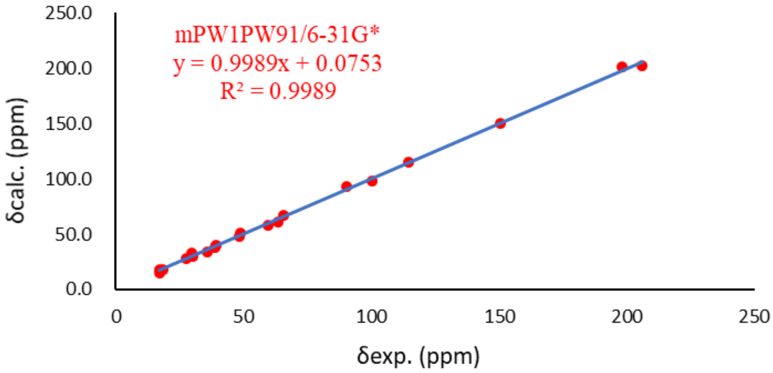

Figure S86. Regression analysis of experimental *versus* calculated ^{13}C NMR **c**hemical shifts at mPW1PW91/6-31G* level using GIAO method of **3a**; linear fitting is shown as a line (blue).

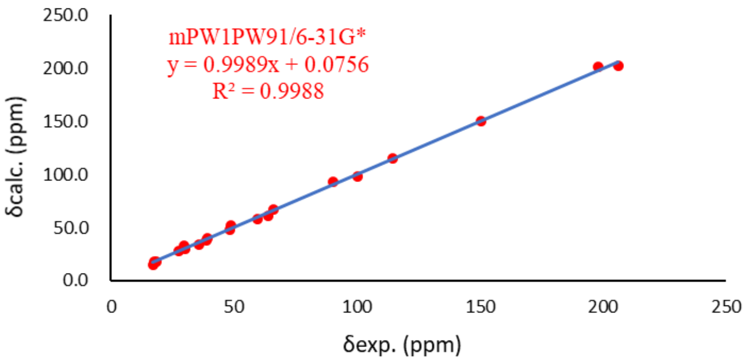

Figure S87. Regression analysis of experimental *versus* calculated ^{13}C NMR **c**hemical shifts at mPW1PW91/6-31G* level using GIAO method of **3b**; linear fitting is shown as a line (blue).

Functional		Solvent?		Basis Set		Type of Data	
mPW1PW91		PCM		6-31G(d)		Shielding Tensors	
		DP4+	76.94%	23.06%	–	–	–
Nuclei	sp2?	xperimenta	Isomer 1	Isomer 2	Isomer 3	Isomer 4	Isomer 5
C		39.2	154.0374053	154.0793742			
C		35.7	159.0487202	159.4976058			
C		29.8	159.965917	160.1030116			
C	x	150.4	48.55365689	48.52530175			
C		30.1	163.4483979	163.3940291			
C		63.7	133.3971039	133.3362534			
C		59.5	136.2276454	136.1328979			
C		38.8	155.2903294	155.2934902			
C		27.7	164.9832568	164.9218395			
C		48.7	142.8184764	142.2738077			
C		48.2	146.0586538	145.9884704			
C	x	114.6	82.35095239	82.3859694			
C		17.4	174.7779442	174.8903585			
C	x	198.1	0.793624287	0.864146715			
C		17.1	177.165943	177.0916575			
C		90.4	103.0056223	103.0455202			
C	x	206.1	-0.632364268	-0.553930485			
C	x	100.4	98.04954575	98.11299075			
C		65.9	128.0620078	127.9187716			
C		18.4	174.8540026	174.9171669			

Functional	Solvent?		Basis Set		Type of Data	
mPW1PW91	PCM		6-31G(d)		Shielding Tensors	
	Isomer 1	Isomer 2	Isomer 3	Isomer 4	Isomer 5	Isomer 6
sDP4+ (H data)	–	–	–	–	–	–
sDP4+ (C data)	67.28%	32.72%	–	–	–	–
sDP4+ (all data)	67.28%	32.72%	–	–	–	–
uDP4+ (H data)	–	–	–	–	–	–
uDP4+ (C data)	61.86%	38.14%	–	–	–	–
uDP4+ (all data)	61.86%	38.14%	–	–	–	–
DP4+ (H data)	–	–	–	–	–	–
DP4+ (C data)	76.94%	23.06%	–	–	–	–
DP4+ (all data)	76.94%	23.06%	–	–	–	–

Figure S88. DP4+ evaluation of theoretical and experimental data of **3**. It failed since the difference on DP4+ values between the two configurations were not distinct.

2.1.3 QM-NMR calculation for compound 9

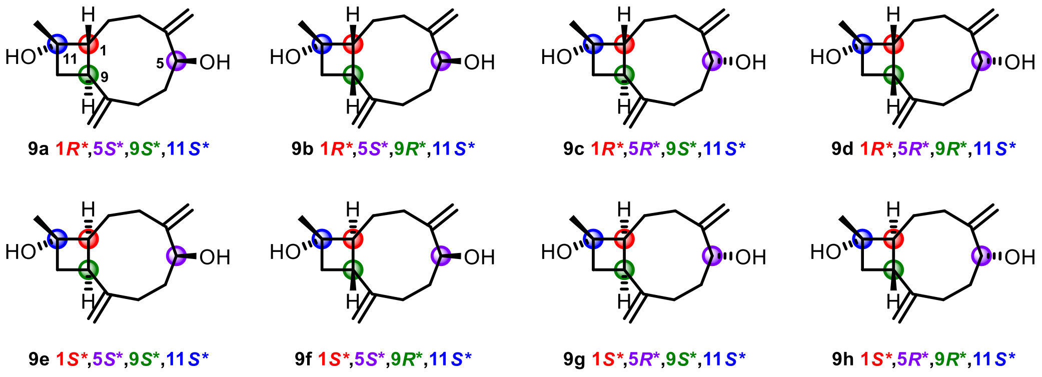

Figure S89. Structures of eight stereoisomers **9a–9h**.

Table S3. Cartesian coordinates of all conformers of isomers **9a–9h** used after optimization at the B3LYP/6-311G(d,p) level of theory as required for DP4+ analysis.

9a_1				9a_2				9a_3				9a_4			
C	2.711135	-0.1282	-0.01191	C	2.718332	-0.11447	-0.0138	C	2.728863	-0.27838	-0.02445	C	2.74066	-0.24474	-0.05788
C	2.353912	1.3813	0.075257	C	2.352645	1.385599	0.056358	C	2.51086	1.257333	0.077932	C	2.482341	1.278242	-0.04255
H	0.974859	-0.49172	1.206208	H	0.97577	-0.48538	1.213521	H	0.925338	-0.48904	1.125296	H	0.985808	-0.46133	1.194901
H	0.869848	1.008681	-1.44172	H	0.859788	0.996505	-1.44603	H	1.064847	1.031812	-1.50376	H	0.942716	0.936183	-1.51062
C	-1.53133	1.78168	-0.71947	C	-1.53456	1.778424	-0.72261	C	-1.34006	1.92241	-0.83684	C	-1.37227	1.96269	-0.80985
C	-0.29936	1.771471	0.171996	C	-0.30134	1.770904	0.167106	C	-0.09568	1.904804	0.047634	C	-0.1243	1.887806	0.06354
C	-1.94126	0.474549	-1.42539	C	-1.94355	0.469434	-1.42574	C	-2.57988	1.130664	-0.36922	C	-2.59992	1.122653	-0.39945
C	-2.61405	-0.61208	-0.56998	C	-2.61495	-0.6156	-0.56739	C	-2.54211	-0.39493	-0.52037	C	-2.48758	-0.39879	-0.54596
C	-1.7559	-1.24585	0.517889	C	-1.75535	-1.24493	0.5218	C	-1.74977	-1.16208	0.537737	C	-1.7566	-1.13206	0.578923
C	-0.67786	-2.22342	0.089743	C	-0.67681	-2.22329	0.09671	C	-0.72671	-2.16649	0.053888	C	-0.72673	-2.16866	0.185451
C	0.594383	-1.64728	-0.58244	C	0.59877	-1.64786	-0.5714	C	0.481166	-1.56905	-0.70031	C	0.511511	-1.63266	-0.56665
C	1.195134	-0.44177	0.135321	C	1.196703	-0.43888	0.142472	C	1.185601	-0.44761	0.063099	C	1.200589	-0.45573	0.12142
C	0.902471	1.026311	-0.34861	C	0.899009	1.023369	-0.3533	C	1.053041	1.043727	-0.4098	C	1.000594	1.005793	-0.42076
C	-0.2884	2.454507	1.319927	C	-0.2871	2.457958	1.31254	C	-0.03654	2.670142	1.141193	C	-0.0347	2.625957	1.173749
C	3.676888	-0.71346	1.004848	C	3.684542	-0.68958	1.008059	C	3.596289	-0.96673	1.016137	C	3.673523	-0.84721	0.979136
O	3.10324	-0.49269	-1.34448	O	3.183291	-0.37033	-1.34928	O	3.134067	-0.65869	-1.34817	O	3.173406	-0.5828	-1.38533
C	-2.00498	-1.02337	1.810096	C	-2.00412	-1.01968	1.81351	C	-2.02604	-1.01504	1.835204	C	-2.09332	-0.941	1.856052
O	-3.81358	-0.02352	-0.05711	O	-3.81458	-0.02688	-0.05538	O	-3.92884	-0.80384	-0.49541	O	-3.85202	-0.87001	-0.63894
H	2.912297	2.055824	-0.57818	H	2.909124	2.04617	-0.61125	H	3.156663	1.887651	-0.53806	H	3.074474	1.872098	-0.74152
H	2.416116	1.742062	1.103637	H	2.424058	1.757087	1.079705	H	2.563414	1.591712	1.1159	H	2.594233	1.68321	0.964341
H	-1.33245	2.507076	-1.5207	H	-1.33741	2.502655	-1.52528	H	-1.07229	1.591648	-1.846	H	-1.10434	1.707313	-1.84079
H	-2.38469	2.172344	-0.16056	H	-2.38776	2.168998	-0.1634	H	-1.66059	2.963626	-0.93759	H	-1.70644	3.00426	-0.83185
H	-2.66878	0.735716	-2.20036	H	-2.67144	0.728032	-2.20117	H	-2.82216	1.391128	0.664813	H	-2.89938	1.37526	0.6213
H	-1.09092	0.037454	-1.9507	H	-1.09308	0.031741	-1.95057	H	-3.42427	1.462217	-0.98225	H	-3.42657	1.422307	-1.05171
H	-2.88329	-1.4243	-1.26381	H	-2.88365	-1.43005	-1.25878	H	-2.13014	-0.63332	-1.51042	H	-1.98325	-0.61754	-1.49663
H	-0.36389	-2.77294	0.981966	H	-0.36519	-2.77231	0.989811	H	-0.36032	-2.73261	0.915675	H	-0.39241	-2.68488	1.090317
H	-1.11659	-2.96342	-0.59149	H	-1.11325	-2.96326	-0.58577	H	-1.22172	-2.88875	-0.60878	H	-1.20572	-2.92776	-0.44749
H	1.335958	-2.45303	-0.60815	H	1.333514	-2.46306	-0.58539	H	1.189288	-2.37937	-0.89961	H	1.212972	-2.47064	-0.66268
H	0.403733	-1.39709	-1.62912	H	0.412983	-1.40456	-1.62094	H	0.169314	-1.2025	-1.68313	H	0.244836	-1.35426	-1.59073
H	0.594981	2.527627	1.944328	H	0.597993	2.532271	1.934456	H	0.83693	2.701055	1.782422	H	0.842292	2.614002	1.811037
H	-1.17642	2.965576	1.677922	H	-1.17361	2.971275	1.671	H	-0.87277	3.296828	1.43627	H	-0.84998	3.26986	1.48925
H	3.708822	-1.80352	0.917297	H	3.742427	-1.7802	0.917519	H	3.527802	-2.05363	0.911766	H	3.653215	-1.94194	0.932012
H	4.690211	-0.33127	0.838005	H	4.688145	-0.28358	0.851407	H	4.646713	-0.68063	0.8921	H	4.701697	-0.51997	0.799194
H	3.382479	-0.45408	2.024907	H	3.3676	-0.45317	2.026828	H	3.289895	-0.69406	2.029158	H	3.384549	-0.54916	1.989971
H	3.971483	-0.10607	-1.50708	H	3.256061	-1.32545	-1.45972	H	4.041123	-0.35584	-1.47188	H	3.191808	-1.54428	-1.45674
H	-2.78239	-0.34022	2.129843	H	-2.7826	-0.33706	2.131653	H	-2.78272	-0.3216	2.185881	H	-2.85609	-0.229	2.151003
H	-1.4264	-1.51194	2.586694	H	-1.42444	-1.50517	2.591183	H	-1.51136	-1.59709	2.592506	H	-1.62146	-1.50455	2.654153
H	-4.27697	-0.69946	0.450037	H	-4.27801	-0.70221	0.452552	H	-3.94898	-1.76166	-0.39276	H	-3.83684	-1.83045	-0.5637

9a_5				9a_6				9a_7				9a_8			
C	2.714998	-0.229061	-0.081035	C	2.719584	-0.211216	-0.091542	C	2.701116	-0.092907	0.03313	C	2.707224	-0.07756	0.032786
C	2.425102	1.294165	-0.119445	C	2.412053	1.298656	-0.171862	C	2.316241	1.405711	0.176467	C	2.314692	1.412435	0.156358
H	1.019192	-0.432307	1.235751	H	1.030253	-0.416237	1.257424	H	0.915901	-0.541605	1.150245	H	0.914858	-0.534375	1.157669

H	0.840169	0.861235	-1.517123	H	0.793995	0.825795	-1.517738	H	0.909626	1.083119	-1.424665	H	0.8984	1.071186	-1.430967
C	-1.426322	1.976032	-0.80912	C	-1.436725	1.987451	-0.80061	C	-1.521759	1.782143	-0.796675	C	-1.526361	1.778418	-0.800606
C	-0.176298	1.87349	0.057878	C	-0.184271	1.871354	0.060027	C	-0.342173	1.761599	0.158704	C	-0.34518	1.761909	0.152774
C	-2.639389	1.086269	-0.451405	C	-2.642988	1.081739	-0.459481	C	-1.976675	0.451739	-1.432591	C	-1.979547	0.445505	-1.432669
C	-2.420134	-0.428452	-0.558033	C	-2.403107	-0.430155	-0.562501	C	-2.64794	-0.584504	-0.503705	C	-2.648364	-0.589087	-0.500375
C	-1.719709	-1.088774	0.625928	C	-1.719687	-1.083317	0.635377	C	-1.728703	-1.334072	0.455508	C	-1.726766	-1.334744	0.459496
C	-0.728243	-2.179957	0.301668	C	-0.72618	-2.179068	0.332735	C	-0.659914	-2.240957	-0.132729	C	-0.65764	-2.241958	-0.127504
C	0.527991	-1.694437	-0.45804	C	0.540048	-1.704297	-0.417886	C	0.628161	-1.599913	-0.715596	C	0.633497	-1.600397	-0.705056
C	1.195349	-0.464025	0.15596	C	1.198148	-0.460012	0.176597	C	1.184604	-0.433509	0.095155	C	1.185003	-0.429848	0.10249
C	0.936731	0.974674	-0.433943	C	0.918114	0.963149	-0.440194	C	0.892639	1.051491	-0.331205	C	0.888265	1.049512	-0.337102
C	-0.067786	2.60917	1.168033	C	-0.062363	2.601476	1.172442	C	-0.402287	2.401962	1.329536	C	-0.402016	2.407165	1.321014
C	3.699335	-0.763777	0.945846	C	3.719247	-0.70833	0.93913	C	3.628689	-0.712332	1.064909	C	3.634198	-0.686844	1.070822
O	3.03573	-0.726863	-1.389785	O	3.103735	-0.630549	-1.411255	O	3.160734	-0.385145	-1.295563	O	3.238886	-0.262613	-1.28924
C	-2.026483	-0.778348	1.887503	C	-2.044261	-0.768114	1.891249	C	-1.932283	-1.306133	1.774354	C	-1.928892	-1.304542	1.778459
O	-3.669905	-1.11478	-0.799957	O	-3.638938	-1.132169	-0.829573	O	-3.78129	-0.004512	0.147803	O	-3.781254	-0.009008	0.151571
H	2.974595	1.878598	-0.861427	H	2.943825	1.854386	-0.946738	H	2.892972	2.119564	-0.416373	H	2.890558	2.112132	-0.452289
H	2.555977	1.748379	0.864473	H	2.565264	1.784108	0.793077	H	2.324286	1.716062	1.223016	H	2.331985	1.735234	1.198397
H	-1.151848	1.774727	-1.850672	H	-1.163112	1.809696	-1.846744	H	-1.247525	2.438079	-1.633895	H	-1.25428	2.432724	-1.639793
H	-1.779359	3.011061	-0.780009	H	-1.79609	3.019352	-0.748502	H	-2.373795	2.270988	-0.313844	H	-2.378507	2.267317	-0.317984
H	-3.012894	1.351431	0.541981	H	-3.032751	1.342355	0.528729	H	-2.719757	0.702039	-2.195951	H	-2.723292	0.692288	-2.196464
H	-3.437131	1.328941	-1.160382	H	-3.43449	1.314646	-1.178589	H	-1.147004	-0.018025	-1.963866	H	-1.149342	-0.024395	-1.963309
H	-1.845971	-0.622661	-1.467998	H	-1.808723	-0.617311	-1.46098	H	-3.081793	-1.347927	-1.161058	H	-3.08216	-1.354906	-1.154999
H	-0.416507	-2.661848	1.233232	H	-0.425308	-2.651197	1.272584	H	-0.355818	-2.925786	0.66357	H	-0.355885	-2.927851	0.668515
H	-1.225266	-2.948389	-0.303707	H	-1.217215	-2.953258	-0.269954	H	-1.113751	-2.863319	-0.914078	H	-1.109043	-2.862818	-0.911243
H	1.245497	-2.521232	-0.479237	H	1.252407	-2.538742	-0.404939	H	1.382631	-2.392007	-0.767907	H	1.382184	-2.401379	-0.746411
H	0.285587	-1.491366	-1.505294	H	0.313186	-1.526503	-1.473294	H	0.472474	-1.283541	-1.74985	H	0.483842	-1.290821	-1.742564
H	0.810552	2.578915	1.803027	H	0.819259	2.559374	1.802417	H	0.443611	2.456915	2.005494	H	0.445812	2.464478	1.994435
H	-0.869922	3.26735	1.48756	H	-0.855711	3.266739	1.498945	H	-1.312598	2.895478	1.654612	H	-1.311177	2.902442	1.646571
H	3.687088	-1.857766	0.951452	H	3.737766	-1.803792	0.965755	H	3.688891	-1.79533	0.922577	H	3.71921	-1.769942	0.926982
H	4.718455	-0.438252	0.709015	H	4.725622	-0.358969	0.690443	H	4.640161	-0.301929	0.96888	H	4.635494	-0.254791	0.984966
H	3.456482	-0.407711	1.950198	H	3.46385	-0.35238	1.94026	H	3.278938	-0.512859	2.080942	H	3.263184	-0.509621	2.083291
H	3.907215	-0.386107	-1.622194	H	3.141685	-1.594035	-1.417371	H	4.027345	0.024781	-1.399347	H	3.339758	-1.21012	-1.43719
H	-2.729376	0.006013	2.144686	H	-2.751304	0.016386	2.135724	H	-2.725715	-0.72269	2.224746	H	-2.72284	-0.721184	2.227943
H	-1.576608	-1.310256	2.719453	H	-1.605651	-1.296582	2.731348	H	-1.308875	-1.88293	2.44922	H	-1.303829	-1.878788	2.453929
H	-4.153388	-1.117432	0.034374	H	-4.140818	-1.137976	-0.006132	H	-3.464039	0.696273	0.72803	H	-3.46466	0.696377	0.726547

**9a_9**				**9a_10**				**9a_11**				**9a_12**			
C	2.717628	-0.127108	-0.012	C	2.738531	-0.257597	-0.056784	C	-2.659083	-0.230146	-0.215385	C	-2.652846	-0.245236	-0.218299
C	2.360187	1.384723	0.037088	C	2.489385	1.275512	-0.067514	C	-2.354777	1.271886	-0.014678	C	-2.354598	1.269069	-0.039756
H	0.955698	-0.486527	1.189901	H	0.970383	-0.459737	1.179847	H	-0.795009	-0.371928	-1.270916	H	-0.795831	-0.382415	-1.263303
H	0.839653	1.019486	-1.460696	H	0.918958	0.949301	-1.523162	H	-1.042427	0.680185	1.590674	H	-1.060812	0.69263	1.58607
C	-1.538177	1.785929	-0.707526	C	-1.374113	1.972438	-0.795592	C	0.943844	1.869045	-1.012912	C	0.941827	1.86856	-1.014421
C	-0.295091	1.775432	0.168336	C	-0.117285	1.893126	0.064365	C	0.220624	1.81824	0.317969	C	0.216239	1.817974	0.315279
C	-1.956062	0.481997	-1.415328	C	-2.597078	1.127995	-0.379315	C	1.753708	0.620383	-1.439922	C	1.751083	0.619473	-1.440644
C	-2.619818	-0.609692	-0.559053	C	-2.487402	-0.392494	-0.540532	C	2.612616	-0.051734	-0.365346	C	2.613395	-0.048763	-0.366435
C	-1.749987	-1.252535	0.514081	C	-1.75394	-1.138487	0.574441	C	1.888695	-0.941133	0.63628	C	1.892723	-0.936843	0.638582
C	-0.676439	-2.226838	0.067115	C	-0.727456	-2.173311	0.166803	C	0.954365	-2.015176	0.101802	C	0.957816	-2.012493	0.108415
C	0.593718	-1.641801	-0.601966	C	0.50653	-1.629715	-0.585658	C	-0.549942	-1.767291	0.360568	C	-0.545849	-1.769491	0.373824
C	1.190497	-0.438309	0.12223	C	1.194518	-0.455563	0.108581	C	-1.112407	-0.480183	-0.231004	C	-1.113396	-0.48673	-0.223063
C	0.901113	1.029279	-0.366012	C	1.001318	1.008483	-0.431741	C	-0.941769	0.877761	0.521077	C	-0.948022	0.879864	0.516019
C	-0.269703	2.453816	1.318492	C	-0.015574	2.624321	1.177873	C	0.574423	2.651658	1.302333	C	0.569645	2.650724	1.300446
C	3.649997	-0.688464	1.042839	C	3.644615	-0.840583	1.009024	C	-3.470159	-0.667222	-1.423705	C	-3.463808	-0.697542	-1.421146
O	3.267334	-0.528694	-1.275697	O	3.239026	-0.748228	-1.309158	O	-3.268719	-0.699104	0.997822	O	-3.184762	-0.811635	0.988775
C	-1.98456	-1.040319	1.810731	C	-2.083907	-0.957003	1.854768	C	2.160515	-0.83489	1.938654	C	2.167782	-0.827044	1.940047
O	-3.811991	-0.022858	-0.028046	O	-3.852899	-0.860533	-0.633365	O	3.549247	-0.869497	-1.104525	O	3.550051	-0.866611	-1.105639
H	2.917472	2.038527	-0.637884	H	3.078596	1.859577	-0.778506	H	-3.014271	1.812051	0.667089	H	-3.015894	1.827864	0.626558
H	2.43338	1.774605	1.053728	H	2.608171	1.702237	0.929895	H	-2.302913	1.798717	-0.970293	H	-2.289511	1.782785	-1.002429
H	-1.351571	2.516104	-1.507349	H	-1.117305	1.725967	-1.831609	H	1.622268	2.726393	-0.988392	H	1.621183	2.725151	-0.988387
H	-2.384756	2.171868	-0.135388	H	-1.709368	3.013774	-0.805753	H	0.222047	2.074913	-1.813294	H	0.221636	2.075588	-1.815932
H	-2.692506	0.748127	-2.180004	H	-2.885935	1.372265	0.646443	H	1.108009	-0.131007	-1.896762	H	1.104633	-0.13354	-1.893731
H	-1.113126	0.046926	-1.954235	H	-3.430084	1.433645	-1.020564	H	2.444876	0.934013	-2.227924	H	2.439743	0.931637	-2.231438
H	-2.898319	-1.416473	-1.255491	H	-1.987037	-0.602872	-1.495176	H	3.164031	0.717569	0.190526	H	3.164753	0.722849	0.186372
H	-0.358046	-2.788049	0.950245	H	-0.389134	-2.697857	1.065413	H	1.124448	-2.143612	-0.970581	H	1.124683	-2.141382	-0.964583
H	-1.118912	-2.957242	-0.621722	H	-1.210061	-2.926326	-0.470638	H	1.217982	-2.970826	0.565329	H	1.225925	-2.967279	0.571377
H	1.340206	-2.442856	-0.634491	H	1.220873	-2.452301	-0.696337	H	-1.098822	-2.621392	-0.057488	H	-1.098398	-2.623462	-0.036217
H	0.395716	-1.389173	-1.647925	H	0.229366	-1.343843	-1.605901	H	-0.734339	-1.777731	1.439709	H	-0.728835	-1.774737	1.452238
H	0.621585	2.526631	1.931489	H	0.867765	2.608635	1.806132	H	0.073933	2.636271	2.265737	H	0.067669	2.635674	2.263081

H	-1.154108	2.961146	1.690178	H	-0.827877	3.265834	1.505425	H	1.373546	3.375436	1.178306	H	1.369914	3.373488	1.17788
H	3.690251	-1.779825	0.974998	H	3.604141	-1.933838	0.987889	H	-3.493453	-1.760181	-1.501197	H	-3.464281	-1.789165	-1.49274
H	4.662843	-0.301143	0.898704	H	4.680398	-0.534819	0.835016	H	-4.501155	-0.312361	-1.336483	H	-4.503668	-0.364002	-1.331929
H	3.311915	-0.415748	2.044508	H	3.343726	-0.502022	2.002395	H	-3.043266	-0.273523	-2.349461	H	-3.057337	-0.287373	-2.349106
H	2.747037	-0.133731	-1.983625	H	2.729352	-0.354769	-2.025842	H	-3.31951	-1.660881	0.950862	H	-4.083792	-0.47905	1.093236
H	-2.757947	-0.359434	2.144705	H	-2.842754	-0.244927	2.159567	H	2.825196	-0.065779	2.318365	H	2.832611	-0.056248	2.316159
H	-1.397997	-1.535861	2.576839	H	-1.610493	-1.529282	2.645716	H	1.717013	-1.50216	2.671605	H	1.726738	-1.492889	2.675778
H	-4.271044	-0.70161	0.479302	H	-3.839562	-1.821305	-0.562387	H	4.083667	-1.34255	-0.456169	H	4.084611	-1.339279	-0.457115

**9a_13**				**9b_1**				**9b_2**				**9b_3**			
C	2.720347	-0.225468	-0.088029	C	-2.720856	-0.363848	-0.175153	C	-2.726384	-0.357514	-0.17107	C	2.790953	-0.303361	-0.001647
C	2.420829	1.293785	-0.182295	C	-2.471142	1.049074	0.409618	C	-2.467427	1.044062	0.410792	C	2.49961	1.198966	-0.248427
H	1.01268	-0.422695	1.236386	H	-0.937917	-0.894383	-1.407706	H	-0.934939	-0.891385	-1.416553	H	1.078545	-1.135224	1.16887
H	0.782107	0.845837	-1.530037	H	-1.208062	1.472528	-1.29957	H	-1.211705	1.472862	-1.302539	H	1.331095	1.211449	1.580342
C	-1.435904	1.992154	-0.790246	C	1.265494	2.023459	-0.590702	C	1.266382	2.021173	-0.59834	C	-1.263226	1.582676	1.054596
C	-0.175751	1.875211	0.059074	C	0.094044	1.757541	0.336817	C	0.094672	1.758807	0.329858	C	-0.075773	1.786675	0.120229
C	-2.641251	1.089347	-0.437847	C	1.856463	0.810999	-1.333637	C	1.860093	0.806146	-1.334932	C	-2.590437	1.180847	0.394434
C	-2.410615	-0.423198	-0.555464	C	2.766499	-0.086577	-0.494617	C	2.767965	-0.088462	-0.490306	C	-2.543743	-0.088486	-0.464343
C	-1.718064	-1.090213	0.629334	C	2.104752	-0.857017	0.632107	C	2.102896	-0.857993	0.635066	C	-1.883752	-1.289918	0.213041
C	-0.72852	-2.184061	0.306606	C	0.980769	-1.819172	0.289544	C	0.98145	-1.82222	0.289949	C	-0.75345	-1.967606	-0.530813
C	0.532403	-1.70167	-0.447427	C	-0.462876	-1.37372	0.634255	C	-0.463098	-1.374006	0.626635	C	0.475466	-1.110992	-0.910417
C	1.192438	-0.463379	0.157601	C	-1.179647	-0.513273	-0.410789	C	-1.175721	-0.513149	-0.418198	C	1.265536	-0.535433	0.273699
C	0.922988	0.966795	-0.450057	C	-1.089508	1.066066	-0.288951	C	-1.08851	1.064657	-0.293145	C	1.164216	1.032299	0.512183
C	-0.045309	2.600729	1.173313	C	0.096768	2.20458	1.594863	C	0.097201	2.211307	1.586016	C	-0.132642	2.609128	-0.930097
C	3.688968	-0.712321	0.971141	C	-3.531285	-0.367886	-1.467517	C	-3.539519	-0.36505	-1.461139	C	3.666329	-0.572389	1.218921
O	3.187483	-0.775752	-1.328998	O	-3.24113	-1.334933	0.731711	O	-3.324667	-1.21872	0.795034	O	3.273642	-1.041804	-1.122247
C	-2.0307	-0.785645	1.890935	C	2.581775	-0.750765	1.874348	C	2.574618	-0.749046	1.879099	C	-2.34711	-1.76411	1.371266
O	-3.653141	-1.115989	-0.815283	O	3.35516	-1.012562	-1.433437	O	3.362133	-1.015167	-1.424889	O	-3.917676	-0.358993	-0.808648
H	2.955199	1.846853	-0.95863	H	-2.382548	0.976535	1.49487	H	-2.374315	0.968339	1.495509	H	2.343644	1.364391	-1.316363
H	2.573089	1.790153	0.777502	H	-3.175805	1.844959	0.155699	H	-3.175995	1.838509	0.16461	H	3.215451	1.93182	0.131965
H	-1.173363	1.814486	-1.839298	H	2.060185	2.536344	-0.039565	H	2.059883	2.537845	-0.049055	H	-1.429546	2.50792	1.61797
H	-1.792492	3.024758	-0.734734	H	0.916902	2.729559	-1.355589	H	0.917589	2.72294	-1.367122	H	-1.000425	0.82411	1.795435
H	-3.015805	1.34521	0.557434	H	2.471544	1.177587	-2.161098	H	2.477809	1.170366	-2.161507	H	-2.968513	1.991766	-0.235472
H	-3.441305	1.331259	-1.144367	H	1.065104	0.207777	-1.785928	H	1.070406	0.201175	-1.78767	H	-3.336281	1.023378	1.180614
H	-1.827062	-0.605875	-1.461958	H	3.558501	0.538288	-0.060705	H	3.557087	0.538431	-0.054067	H	-1.991916	0.133936	-1.384014
H	-0.420944	-2.667119	1.238759	H	1.04024	-2.080452	-0.77125	H	1.045885	-2.086348	-0.769805	H	-0.41653	-2.830865	0.052072
H	-1.225367	-2.950835	-0.300814	H	1.16724	-2.746717	0.839122	H	1.165479	-2.747901	0.843493	H	-1.163045	-2.36888	-1.468726
H	1.254632	-2.524708	-0.454914	H	-1.059776	-2.28226	0.747073	H	-1.059794	-2.284181	0.741458	H	1.142501	-1.756383	-1.485321
H	0.292929	-1.513585	-1.499329	H	-0.468629	-0.886645	1.613944	H	-0.473136	-0.887924	1.606838	H	0.177105	-0.30805	-1.591233
H	0.841405	2.558094	1.795871	H	0.962245	2.712692	2.007287	H	0.962556	2.72136	1.996316	H	-1.037967	3.155675	-1.174626
H	-0.837653	3.26254	1.508913	H	-0.752872	2.081804	2.255965	H	-0.752396	2.091575	2.247747	H	0.719702	2.773423	-1.578757
H	3.689232	-1.805745	1.013595	H	-3.513419	-1.36366	-1.918791	H	-3.548964	-1.368637	-1.90049	H	3.685914	-1.643628	1.437054
H	4.704863	-0.380449	0.737702	H	-4.573687	-0.097764	-1.265315	H	-4.571595	-0.066283	-1.257314	H	4.69297	-0.237209	1.034487
H	3.414327	-0.327782	1.955368	H	-3.137591	0.347822	-2.193549	H	-3.122023	0.31831	-2.204593	H	3.298809	-0.046073	2.103476
H	2.651744	-0.42462	-2.048453	H	-4.188329	-1.176781	0.818609	H	-3.522921	-2.061314	0.36962	H	4.20854	-0.832335	-1.230928
H	-2.733415	-0.001397	2.148654	H	2.166693	-1.324948	2.697249	H	2.156826	-1.32225	2.701302	H	-1.908871	-2.638061	1.842265
H	-1.58635	-1.323029	2.722301	H	3.394468	-0.072456	2.113968	H	3.385382	-0.069202	2.120894	H	-3.189469	-1.307287	1.88022
H	-4.144344	-1.129813	0.014456	H	3.935757	-1.596625	-0.932026	H	3.941446	-1.597469	-0.919953	H	-3.931305	-1.157271	-1.347922

**9b_4**				**9b_5**				**9b_6**				**9b_7**			
C	2.795392	-0.298836	-0.004057	C	-2.72678	-0.367418	-0.180362	C	2.748373	-0.234112	-0.035972	C	-2.712578	-0.363329	-0.174075
C	2.499521	1.192112	-0.252818	C	-2.47476	1.037244	0.41749	C	2.524267	1.088517	0.744714	C	-2.46951	1.076192	0.345243
H	1.070498	-1.131314	1.176605	H	-0.933019	-0.882879	-1.427295	H	1.369376	-1.383354	1.190609	H	-0.916746	-0.948694	-1.359514
H	1.338264	1.214874	1.581177	H	-1.22167	1.4821	-1.292352	H	0.727147	0.71548	1.971714	H	-1.172577	1.420932	-1.356698
C	-1.25987	1.579431	1.057948	C	1.257867	2.026656	-0.587416	C	-1.366834	1.760464	0.890513	C	1.286771	1.999979	-0.624197
C	-0.072696	1.788884	0.124466	C	0.087343	1.756768	0.340205	C	-0.004731	1.640106	0.210378	C	0.099947	1.768259	0.292622
C	-2.58744	1.179523	0.397187	C	1.854749	0.817329	-1.330658	C	-2.587572	1.302809	0.072674	C	1.868284	0.768935	-1.347333
C	-2.543971	-0.088105	-0.464593	C	2.764566	-0.07995	-0.490903	C	-2.479218	-0.080663	-0.579383	C	2.772183	-0.135063	-0.500718
C	-1.886781	-1.29315	0.209249	C	2.101223	-0.857962	0.629703	C	-1.912079	-1.184782	0.313269	C	2.107839	-0.841831	0.667868
C	-0.754226	-1.96791	-0.534179	C	0.98378	-1.824215	0.277745	C	-0.819345	-2.043867	-0.286542	C	0.975143	-1.816182	0.389818
C	0.4726	-1.105428	-0.905584	C	-0.46311	-1.381693	0.610807	C	0.461781	-1.320396	-0.754791	C	-0.465528	-1.341306	0.706858
C	1.259547	-0.53411	0.280009	C	-1.175706	-0.510921	-0.427369	C	1.305043	-0.689321	0.346662	C	-1.168736	-0.525464	-0.382159
C	1.166552	1.032787	0.51424	C	-1.095659	1.065171	-0.287128	C	1.010326	0.767484	0.919167	C	-1.075281	1.057748	-0.327687
C	-0.130107	2.614307	-0.923541	C	0.091789	2.200644	1.599406	C	0.253988	2.285985	-0.930446	C	0.084089	2.258429	1.534362
C	3.671337	-0.570893	1.214449	C	-3.534661	-0.362232	-1.46939	C	3.895354	-1.127499	0.413179	C	-3.50551	-0.429692	-1.475589
O	3.351075	-0.919398	-1.160319	O	-3.358042	-1.323639	0.666374	O	2.814106	-0.020619	-1.453636	O	-3.244666	-1.289214	0.772001
C	-2.353811	-1.77194	1.364062	C	2.571332	-0.75562	1.874811	C	-2.417009	-1.429026	1.523897	C	2.578042	-0.664332	1.903561

O	-3.918918	-0.354143	-0.808265
H	2.338978	1.352494	-1.320887
H	3.22087	1.924308	0.117492
H	-1.427009	2.501923	1.625582
H	-0.995859	0.817762	1.795231
H	-2.965309	1.99215	-0.230645
H	-3.33289	1.020999	1.18355
H	-1.992504	0.135121	-1.384283
H	-0.41758	-2.832495	0.046894
H	-1.161004	-2.366441	-1.474469
H	1.143603	-1.741498	-1.488987
H	0.172629	-0.299933	-1.582694
H	-1.035908	3.160527	-1.167043
H	0.722418	2.781716	-1.57124
H	3.710501	-1.645336	1.425026
H	4.689507	-0.215777	1.032696
H	3.28541	-0.072313	2.106953
H	3.57181	-1.832257	-0.939673
H	-1.917348	-2.647956	1.832869
H	-3.197463	-1.316722	1.872287
H	-3.935161	-1.151526	-1.348793

O	3.360955	-0.999759	-1.430559
H	-2.375859	0.969733	1.504077
H	-3.185875	1.831566	0.178134
H	2.05015	2.542606	-0.035758
H	0.906747	2.731554	-1.352226
H	2.472027	1.188012	-2.154635
H	1.066952	0.213169	-1.78772
H	3.551854	0.546246	-0.050482
H	1.050058	-2.083049	-0.783048
H	1.167947	-2.751874	0.827635
H	-1.056711	-2.297161	0.703203
H	-0.472374	-0.907906	1.598756
H	0.958015	2.707908	2.011223
H	-0.756208	2.078019	2.262674
H	-3.528548	-1.359575	-1.917825
H	-4.570746	-0.080993	-1.261809
H	-3.123046	0.342664	-2.194543
H	-2.953599	-1.273364	1.53894
H	2.154942	-1.335778	2.692843
H	3.379762	-0.074869	2.121682
H	3.944067	-1.58175	-0.929635

O	-3.81597	-0.394737	-1.02437
H	2.760454	1.991507	0.179412
H	3.05421	1.121718	1.69934
H	-1.529169	2.806221	1.174764
H	-1.345925	1.193312	1.822411
H	-2.805488	2.02058	-0.724037
H	-3.462724	1.297053	0.731206
H	-1.835018	0.007293	-1.459656
H	-0.550408	-2.829581	0.426894
H	-1.238802	-2.553625	-1.165554
H	1.084778	-2.064145	-1.263317
H	0.225107	-0.56946	-1.51134
H	-0.496817	2.906782	-1.409917
H	1.210085	2.201002	-1.432938
H	3.858101	-2.087944	-0.10924
H	4.859854	-0.656515	0.191812
H	3.852493	-1.314815	1.489209
H	3.652619	0.418214	-1.639679
H	-2.04255	-2.23953	2.141138
H	-3.229198	-0.839154	1.936187
H	-3.777278	-1.232393	-1.498871

O	3.42571	-1.100356	-1.353474
H	-2.402364	1.058082	1.434122
H	-3.167286	1.859445	0.038717
H	2.084187	2.506845	-0.071704
H	0.958599	2.700844	-1.402818
H	2.483927	1.117834	-2.18198
H	1.063832	0.172297	-1.790359
H	3.591059	0.471648	-0.104743
H	1.013352	-2.156809	-0.650988
H	1.158266	-2.708226	0.995694
H	-1.070534	-2.237967	0.862672
H	-0.467215	-0.806036	1.660818
H	0.945199	2.776278	1.943751
H	-0.777242	2.162813	2.184583
H	-3.484131	-1.44651	-1.87699
H	-4.549834	-0.147889	-1.30165
H	-3.099784	0.248654	-2.23043
H	-4.1911	-1.120357	0.846975
H	2.154985	-1.186513	2.756638
H	3.393469	0.021893	2.108375
H	2.752639	-1.516075	-1.904118

9b_8

C	2.762907	-0.226933	-0.033428
C	2.533682	1.118032	0.688484
H	1.360229	-1.361159	1.211164
H	0.768815	0.765134	1.964653
C	-1.365563	1.746925	0.897857
C	-0.009731	1.647642	0.202495
C	-2.591385	1.296374	0.084948
C	-2.487607	-0.08393	-0.57503
C	-1.915759	-1.190654	0.311424
C	-0.813096	-2.037185	-0.287528
C	0.468014	-1.304692	-0.74352
C	1.309088	-0.674579	0.360274
C	1.026363	0.795453	0.904524
C	0.225219	2.291739	-0.944443
C	3.901612	-1.107689	0.459414
O	2.915212	0.048381	-1.433307
C	-2.426472	-1.449565	1.516535
O	-3.827247	-0.394483	-1.012572
H	2.754299	1.987409	0.068489
H	3.085351	1.203066	1.62667
H	-1.530314	2.786466	1.203108
H	-1.331671	1.163482	1.81931
H	-2.815024	2.019138	-0.705596
H	-3.462005	1.285771	0.749346
H	-1.848277	0.006771	-1.458792
H	-0.542563	-2.825601	0.422005
H	-1.223135	-2.54371	-1.172705
H	1.087418	-2.05525	-1.250695
H	0.232625	-0.553487	-1.500953
H	-0.541784	2.896252	-1.418824
H	1.178041	2.225896	-1.455935
H	3.881677	-2.084411	-0.037075
H	4.864619	-0.635501	0.243948
H	3.830685	-1.277203	1.536812
H	2.934431	-0.792696	-1.904726
H	-2.048593	-2.261306	2.129967
H	-3.247383	-0.870834	1.927225
H	-3.794784	-1.235029	-1.482412

9b_9

C	2.772542	-0.273245	0.003016
C	2.45069	1.221214	-0.249423
H	1.068005	-1.13707	1.160381
H	1.269462	1.207594	1.568496
C	-1.318925	1.620772	1.047441
C	-0.130237	1.782877	0.103485
C	-2.633221	1.149053	0.397539
C	-2.502255	-0.11693	-0.45932
C	-1.823655	-1.293063	0.239321
C	-0.741332	-2.001212	-0.539252
C	0.488131	-1.144993	-0.9235
C	1.25029	-0.538678	0.263408
C	1.11149	1.029135	0.498997
C	-0.187439	2.586497	-0.961764
C	3.642877	-0.521765	1.231512
O	3.281348	-1.002089	-1.112589
C	-2.209435	-1.702905	1.449982
O	-3.78307	-0.505905	-0.99356
H	2.302101	1.379576	-1.319434
H	3.146195	1.97121	0.135479
H	-1.507131	2.578158	1.545836
H	-1.047888	0.917079	1.837225
H	-3.048322	1.933234	-0.24263
H	-3.370053	0.974056	1.190103
H	-1.92238	0.137428	-1.347077
H	-0.402897	-2.873379	0.029038
H	-1.180838	-2.381897	-1.471046
H	1.170098	-1.795411	-1.474576
H	0.193614	-0.358909	-1.625582
H	-1.08881	3.140141	-1.205568
H	0.658791	2.728691	-1.623267
H	3.682824	-1.591893	1.452282
H	4.663964	-0.165954	1.054943
H	3.257295	-0.001273	2.11185
H	4.211857	-0.770683	-1.2143
H	-1.760694	-2.570826	1.922031
H	-2.988247	-1.198765	2.013967
H	-4.310303	-0.837681	-0.257621

9b_10

C	2.777012	-0.267637	0.000654
C	2.449501	1.215012	-0.25446
H	1.060504	-1.133955	1.167473
H	1.275778	1.210797	1.568934
C	-1.316615	1.617279	1.051283
C	-0.128408	1.784422	0.107666
C	-2.631178	1.147114	0.400536
C	-2.502214	-0.117049	-0.459464
C	-1.825996	-1.29655	0.23594
C	-0.741771	-2.001774	-0.543076
C	0.486092	-1.140248	-0.919306
C	1.244655	-0.53786	0.269222
C	1.11283	1.029278	0.500651
C	-0.186581	2.590996	-0.955363
C	3.647712	-0.517671	1.227571
O	3.357623	-0.877016	-1.149423
C	-2.214197	-1.710727	1.444303
O	-3.783739	-0.502207	-0.994808
H	2.296152	1.367497	-1.32468
H	3.149855	1.965298	0.120137
H	-1.505619	2.572383	1.55372
H	-1.044394	0.910535	1.837966
H	-3.046197	1.933004	-0.237581
H	-3.367694	0.97084	1.193132
H	-1.922043	0.138664	-1.346632
H	-0.403624	-2.875471	0.023027
H	-1.178924	-2.379504	-1.477163
H	1.171489	-1.782647	-1.478384
H	0.190978	-0.351743	-1.618422
H	-1.088579	3.144289	-1.197663
H	0.659476	2.736372	-1.616443
H	3.708981	-1.590512	1.441001
H	4.659328	-0.14036	1.054011
H	3.243025	-0.025884	2.115557
H	3.598957	-1.78363	-0.924807
H	-1.766649	-2.580508	1.914045
H	-2.993724	-1.208234	2.008791
H	-4.313204	-0.830952	-0.259109

9b_11

C	-2.718834	-0.356201	-0.170642
C	-2.465614	1.071695	0.346071
H	-0.915177	-0.945354	-1.370138
H	-1.175075	1.421702	-1.359887
C	1.288271	1.998107	-0.629888
C	0.100763	1.768753	0.286631
C	1.873268	0.765273	-1.347259
C	2.774706	-0.135636	-0.494794
C	2.10576	-0.843572	0.670451
C	0.975921	-1.81958	0.386594
C	-0.465851	-1.343577	0.696631
C	-1.165291	-0.525761	-0.3906
C	-1.073932	1.056236	-0.332002
C	0.084229	2.263314	1.52671
C	-3.51526	-0.426692	-1.469037
O	-3.329648	-1.167931	0.829696
C	2.569227	-0.665616	1.908597
O	3.43551	-1.10026	-1.342892
H	-2.394012	1.049204	1.434622
H	-3.167128	1.854484	0.047975
H	2.084089	2.508615	-0.078438
H	0.960061	2.695214	-1.411872
H	2.491892	1.112675	-2.180358
H	1.070766	0.167006	-1.791491
H	3.58984	0.473425	-0.094706
H	1.019221	-2.159672	-0.654107
H	1.156816	-2.711451	0.993444
H	-1.070218	-2.242471	0.852244
H	-0.472371	-0.811468	1.652344
H	0.94506	2.78278	1.934641
H	-0.777271	2.170257	2.177072
H	-3.51991	-1.450467	-1.859366
H	-4.549569	-0.117851	-1.293619
H	-3.087351	0.219813	-2.239179
H	-3.532569	-2.027604	0.442466
H	2.142618	-1.188663	2.759375
H	3.38234	0.022015	2.117804
H	2.766877	-1.517112	-1.898022

9b_12

C	2.728443	-0.212678	-0.025532
C	2.473933	1.110655	0.744345
H	1.35239	-1.366637	1.195408
H	0.664704	0.715381	1.946087

9b_13

C	2.800357	-0.307582	-0.002008
C	2.505302	1.192117	-0.241489
H	1.080976	-1.141719	1.179975
H	1.338429	1.203102	1.587875

9c_1

C	2.651329	-0.34888	-0.097559
C	2.479181	1.195241	-0.129315
H	0.973475	-0.395883	1.248288
H	0.856865	0.881226	-1.513754

9c_2

C	2.659095	-0.338156	-0.101019
C	2.477466	1.196257	-0.151033
H	0.974977	-0.388787	1.256071
H	0.84346	0.868661	-1.516466

C	-1.408116	1.807756	0.864241	C	-1.25678	1.578444	1.060314	C	-1.367685	2.009435	-0.737041	C	-1.370757	2.007776	-0.738594
C	-0.050063	1.645961	0.180865	C	-0.06762	1.782848	0.128293	C	-0.096744	1.912956	0.090544	C	-0.097711	1.912963	0.08594
C	-2.631482	1.271497	0.088358	C	-2.584297	1.185895	0.395423	C	-2.015616	0.704492	-1.23689	C	-2.017664	0.701614	-1.236711
C	-2.436495	-0.100354	-0.569824	C	-2.540214	-0.08076	-0.467274	C	-2.726114	-0.152275	-0.183076	C	-2.727392	-0.154017	-0.181664
C	-1.859554	-1.185866	0.333752	C	-1.891929	-1.287198	0.212113	C	-1.854262	-0.871131	0.835748	C	-1.85453	-0.868328	0.839278
C	-0.809923	-2.074677	-0.291152	C	-0.760564	-1.971667	-0.523559	C	-0.947641	-2.001762	0.386501	C	-0.947647	-2.000249	0.393858
C	0.473081	-1.354347	-0.76185	C	0.476521	-1.123179	-0.896977	C	0.332486	-1.6346	-0.407176	C	0.336583	-1.634927	-0.395317
C	1.287943	-0.686629	0.339993	C	1.264845	-0.542741	0.283715	C	1.122621	-0.461296	0.165937	C	1.124335	-0.458391	0.173902
C	0.960379	0.770629	0.897535	C	1.170003	1.023967	0.5202	C	0.97001	0.985768	-0.430967	C	0.965812	0.982161	-0.435515
C	0.213019	2.269828	-0.971486	C	-0.122849	2.608905	-0.919331	C	0.064457	2.667742	1.180573	C	0.06888	2.67196	1.172166
C	3.883206	-1.086004	0.4428	C	3.670884	-0.585312	1.214876	C	3.608496	-0.960225	0.912028	C	3.620142	-0.936533	0.912543
O	2.808725	-0.006129	-1.443705	O	3.399222	-1.024621	-1.076876	O	2.907286	-0.868516	-1.411551	O	2.998044	-0.763101	-1.431205
C	-2.305249	-1.375331	1.577695	C	-2.366579	-1.761756	1.365504	C	-1.978484	-0.56393	2.129801	C	-1.978869	-0.558024	2.132514
O	-3.661691	-0.551066	-1.183	O	-3.913246	-0.34204	-0.82103	O	-3.499857	-1.109894	-0.93513	O	-3.498465	-1.11509	-0.931821
H	2.704698	2.013609	0.176793	H	2.340847	1.378907	-1.306675	H	3.06471	1.737761	-0.875281	H	3.058171	1.722286	-0.91115
H	2.989015	1.156809	1.706513	H	3.225125	1.921935	0.136491	H	2.655282	1.632884	0.855115	H	2.665018	1.644425	0.825897
H	-1.579179	2.870588	1.06621	H	-1.419777	2.501333	1.62841	H	-1.118167	2.591274	-1.634751	H	-1.123747	2.589531	-1.637026
H	-1.371326	1.315158	1.83712	H	-0.998053	0.814698	1.797219	H	-2.111366	2.598989	-0.191218	H	-2.114051	2.596666	-0.191562
H	-2.913777	1.968108	-0.706944	H	-2.956691	2.000663	-0.232836	H	-2.789456	0.977465	-1.961202	H	-2.791514	0.972596	-1.961697
H	-3.48498	1.227509	0.775243	H	-3.333042	1.029095	1.178922	H	-1.294257	0.095673	-1.784844	H	-1.295725	0.092472	-1.7838
H	-1.763591	0.031982	-1.416626	H	-1.980916	0.140923	-1.382693	H	-3.409809	0.504522	0.371321	H	-3.412587	0.502704	0.370844
H	-0.542192	-2.869578	0.41235	H	-0.430474	-2.834472	0.063519	H	-0.641906	-2.54851	1.283453	H	-0.644826	-2.546054	1.292119
H	-1.255426	-2.563678	-1.167792	H	-1.16544	-2.373312	-1.463193	H	-1.535075	-2.698373	-0.220427	H	-1.53316	-2.696892	-0.21461
H	1.111717	-2.103963	-1.241386	H	1.138946	-1.792318	-1.454854	H	0.970431	-2.52509	-0.414819	H	0.966647	-2.533479	-0.389645
H	0.241214	-0.624981	-1.540804	H	0.190333	-0.328856	-1.593704	H	0.095318	-1.439719	-1.455697	H	0.104489	-1.448033	-1.446766
H	-0.528864	2.899174	-1.454012	H	-1.028006	3.156235	-1.162492	H	0.984881	2.669343	1.753661	H	0.991557	2.674099	1.741712
H	1.163983	2.159086	-1.478831	H	0.729613	2.777917	-1.566539	H	-0.730398	3.312774	1.540996	H	-0.723254	3.320098	1.532955
H	3.866813	-2.049981	-0.074124	H	3.70676	-1.660812	1.409546	H	3.511967	-2.050003	0.917164	H	3.549634	-2.030198	0.917773
H	4.8432	-0.601981	0.230256	H	4.689499	-0.230038	1.037107	H	4.645754	-0.713818	0.659086	H	4.650372	-0.665615	0.663864
H	3.830013	-1.267595	1.519374	H	3.280674	-0.088615	2.105582	H	3.410058	-0.588348	1.920412	H	3.400108	-0.580982	1.9222
H	3.639657	0.450361	-1.621195	H	2.996492	-0.733539	-1.901816	H	3.801156	-0.604661	-1.658694	H	2.944467	-1.725505	-1.457198
H	-1.929304	-2.18461	2.195417	H	-1.936293	-2.638732	1.83809	H	-2.65606	0.214384	2.466365	H	-2.657607	0.220111	2.467059
H	-3.061691	-0.738648	2.025499	H	-3.210738	-1.30192	1.868625	H	-1.40042	-1.07193	2.894994	H	-1.399927	-1.063094	2.898952
H	-4.268079	-0.781187	-0.469363	H	-3.928554	-1.139072	-1.362124	H	-3.994618	-1.641894	-0.301364	H	-3.995611	-1.644112	-0.297416

**9c_3**				**9c_4**				**9c_5**				**9c_6**			
C	2.702225	-0.349183	0.181021	C	2.713969	-0.325558	0.171471	C	-2.696762	-0.284268	-0.14485	C	-2.703889	-0.266751	-0.13782
C	2.566799	1.182555	-0.022784	C	2.558791	1.192704	-0.05546	C	-2.4928	1.226467	0.128026	C	-2.486643	1.228079	0.165779
H	0.804707	-0.300968	1.190505	H	0.816122	-0.289573	1.210495	H	-0.850284	-0.273493	-1.255027	H	-0.851245	-0.262531	-1.265629
H	1.208017	0.743133	-1.646354	H	1.171601	0.725132	-1.64569	H	-1.045861	0.677909	1.641234	H	-1.012289	0.659366	1.644609
C	-1.288527	1.64021	-1.060837	C	-1.306176	1.643132	-1.056448	C	1.397547	1.675321	1.030069	C	1.410611	1.681522	1.024076
C	0.028758	1.939548	-0.353133	C	0.012467	1.935578	-0.3485	C	0.065819	1.923121	0.327755	C	0.074885	1.921222	0.326741
C	-2.456889	1.258098	-0.137819	C	-2.472496	1.251556	-0.134882	C	2.539243	1.221013	0.099635	C	2.547513	1.217397	0.092524
C	-2.257177	-0.020862	0.675444	C	-2.262193	-0.027801	0.674084	C	2.23353	-0.063129	-0.671092	C	2.233334	-0.068385	-0.671634
C	-2.058064	-1.290106	-0.156187	C	-2.045515	-1.292169	-0.160021	C	1.97558	-1.295184	0.19176	C	1.966821	-1.295239	0.195682
C	-0.849868	-2.153504	0.143094	C	-0.844004	-2.155603	0.164264	C	0.828894	-2.184575	-0.228506	C	0.825533	-2.186855	-0.234414
C	0.506094	-1.697961	-0.446656	C	0.520546	-1.705414	-0.410597	C	-0.558734	-1.756972	0.305281	C	-0.567864	-1.758703	0.284612
C	1.146024	-0.445853	0.160604	C	1.149509	-0.440332	0.178654	C	-1.146953	-0.442189	-0.214752	C	-1.14662	-0.436702	-0.225813
C	1.137879	0.948372	-0.572454	C	1.120442	0.943465	-0.573397	C	-1.039218	0.925154	0.574532	C	-1.025518	0.919091	0.580896
C	0.175394	3.048571	0.377163	C	0.161801	3.041294	0.386288	C	-0.096853	3.012442	-0.429308	C	-0.095097	3.007198	-0.4335
C	3.44252	-0.862826	1.404754	C	3.471605	-0.818372	1.392824	C	-3.517941	-0.706356	-1.351974	C	-3.531196	-0.660533	-1.350046
O	3.191901	-0.987901	-1.007564	O	3.280609	-0.871369	-1.029487	O	-3.151675	-0.966234	1.034173	O	-3.238879	-0.86257	1.054005
C	-2.96995	-1.660705	-1.057994	C	-2.934935	-1.660574	-1.084625	C	2.755432	-1.600239	1.231862	C	2.73527	-1.596595	1.245172
O	-3.430467	-0.125989	1.508465	O	-3.436455	-0.149692	1.50346	O	3.267096	-0.347884	-1.636427	O	3.264418	-0.365164	-1.635913
H	3.292388	1.654468	-0.689297	H	3.271759	1.649052	-0.744516	H	-3.157571	1.685323	0.863557	H	-3.141888	1.66138	0.923639
H	2.564401	1.705626	0.936063	H	2.575537	1.72928	0.894976	H	-2.534844	1.798838	-0.800621	H	-2.548598	1.82035	-0.74836
H	-1.136801	0.845104	-1.796403	H	-1.155509	0.854802	-1.799425	H	1.262068	0.932896	1.820833	H	1.279533	0.947554	1.823373
H	-1.591569	2.525813	-1.627975	H	-1.610371	2.534006	-1.614568	H	1.713957	2.599256	1.523314	H	1.72948	2.610812	1.50546
H	-2.644971	2.069943	0.571644	H	-2.665487	2.059875	0.577262	H	2.744825	2.002324	-0.638336	H	2.754505	1.994201	-0.649809
H	-3.365145	1.145778	-0.738376	H	-3.380233	1.135803	-0.735572	H	3.455209	1.093631	0.68751	H	3.464668	1.088891	0.678316
H	-1.38407	0.110347	1.325019	H	-1.392316	0.111271	1.326345	H	1.353802	0.119264	-1.290761	H	1.354696	0.117192	-1.291869
H	-0.73865	-2.245756	1.231707	H	-0.747028	-2.238176	1.254739	H	0.792248	-2.2315	-1.323722	H	0.798951	-2.234282	-1.32967
H	-1.048023	-3.160773	-0.234937	H	-1.03692	-3.165923	-0.20801	H	1.016725	-3.20267	0.125287	H	1.010075	-3.204517	0.122023
H	1.204941	-2.528909	-0.303226	H	1.209448	-2.540815	-0.230208	H	-1.259899	-2.555089	0.036729	H	-1.259345	-2.559711	-0.009015
H	0.407532	-1.578468	-1.530111	H	0.441452	-1.612774	-1.498385	H	-0.531156	-1.740007	1.398879	H	-0.554911	-1.756777	1.378999
H	1.104007	3.294352	0.878617	H	1.09102	3.284399	0.887874	H	-1.026493	3.238277	-0.937345	H	-1.027436	3.228414	-0.938632
H	-0.638863	3.757559	0.492663	H	-0.651507	3.750687	0.505755	H	0.709769	3.727217	-0.561725	H	0.70848	3.72417	-0.572218

H	3.325588	-1.946798	1.495202	H	3.389747	-1.90719	1.488417	H	-3.445961	-1.787385	-1.503699	H	-3.492331	-1.74366	-1.512022
H	4.513188	-0.643775	1.326238	H	4.532405	-0.565608	1.307172	H	-4.574264	-0.455481	-1.204567	H	-4.57763	-0.378501	-1.201712
H	3.068638	-0.394347	2.318627	H	3.078634	-0.36964	2.308388	H	-3.174897	-0.203414	-2.259605	H	-3.162594	-0.17176	-2.255192
H	4.118825	-0.741556	-1.106757	H	3.25476	-1.832842	-0.961543	H	-4.063443	-0.693753	1.189493	H	-3.258446	-1.818245	0.927359
H	-3.850988	-1.061811	-1.261987	H	-3.811111	-1.061845	-1.308895	H	3.56442	-0.957731	1.563268	H	3.54157	-0.953558	1.581919
H	-2.871415	-2.586648	-1.615432	H	-2.821771	-2.584626	-1.642439	H	2.60882	-2.516399	1.795093	H	2.58205	-2.51025	1.810709
H	-3.38451	-0.969332	1.97206	H	-3.376348	-0.988238	1.974108	H	4.038753	-0.648702	-1.142394	H	4.035548	-0.665142	-1.140542

**9c_7**				**9c_8**				**9c_9**				**9c_10**			
C	2.645146	-0.350566	-0.105537	C	2.707043	-0.350406	0.181533	C	2.653079	-0.339163	-0.109705	C	2.658502	-0.348433	-0.10188
C	2.470669	1.190975	-0.193647	C	2.575457	1.175199	-0.067495	C	2.468173	1.192037	-0.212596	C	2.48204	1.194987	-0.1597
H	0.998735	-0.342446	1.279154	H	0.791374	-0.293634	1.182951	H	0.998503	-0.338124	1.283484	H	0.959031	-0.399496	1.233844
H	0.821077	0.821825	-1.529641	H	1.170066	0.741239	-1.66635	H	0.808912	0.812911	-1.531966	H	0.827167	0.894837	-1.524535
C	-1.389848	1.987319	-0.748681	C	-1.294913	1.650566	-1.049669	C	-1.393353	1.986462	-0.748689	C	-1.372293	2.017016	-0.719142
C	-0.100634	1.915555	0.054356	C	0.031841	1.942227	-0.356583	C	-0.102265	1.915075	0.051283	C	-0.092883	1.914845	0.094312
C	-2.024248	0.672967	-1.245342	C	-2.454374	1.261804	-0.117932	C	-2.028533	0.671636	-1.243218	C	-2.028736	0.716662	-1.220216
C	-2.737409	-0.202043	-0.201908	C	-2.252255	-0.024998	0.682362	C	-2.739009	-0.202546	-0.197549	C	-2.727727	-0.147767	-0.164844
C	-1.871012	-0.831351	0.880827	C	-2.055212	-1.287149	-0.16056	C	-1.86931	-0.831245	0.882702	C	-1.845084	-0.882784	0.832767
C	-0.938688	-1.975433	0.531076	C	-0.851216	-2.157543	0.1357	C	-0.938336	-1.9758	0.531061	C	-0.947936	-2.009819	0.35595
C	0.325007	-1.652789	-0.304893	C	0.506089	-1.700188	-0.448616	C	0.329414	-1.652143	-0.299721	C	0.333047	-1.631078	-0.432135
C	1.123169	-0.452622	0.19725	C	1.140552	-0.445075	0.156931	C	1.124709	-0.450061	0.201838	C	1.11924	-0.461627	0.153072
C	0.9556	0.969371	-0.454442	C	1.133255	0.943525	-0.587697	C	0.951367	0.966589	-0.458562	C	0.967233	0.986985	-0.441173
C	0.085469	2.710964	1.110962	C	0.193273	3.049101	0.373368	C	0.088441	2.712687	1.105361	C	0.081909	2.6621	1.187145
C	3.626217	-0.922703	0.903966	C	3.420774	-0.823119	1.432424	C	3.63556	-0.900076	0.90451	C	3.592246	-0.937467	0.936519
O	2.869304	-0.92158	-1.403837	O	3.329892	-1.040513	-0.910579	O	2.963941	-0.812942	-1.430161	O	3.067563	-0.907123	-1.359519
C	-2.01079	-0.425538	2.144931	C	-2.964393	-1.646166	-1.069645	C	-2.005741	-0.426258	2.147374	C	-1.950854	-0.59268	2.132359
O	-3.512946	-1.21608	-0.874658	O	-3.423795	-0.137506	1.516851	O	-3.516453	-1.217119	-0.867116	O	-3.517357	-1.093101	-0.915766
H	3.03908	1.704646	-0.972508	H	3.295827	1.619602	-0.758086	H	3.032385	1.689938	-1.003396	H	3.061916	1.722134	-0.920936
H	2.667113	1.666599	0.769064	H	2.595694	1.727176	0.874339	H	2.674728	1.676282	0.743016	H	2.670335	1.65147	0.813492
H	-1.167161	2.575692	-1.649141	H	-1.153969	0.862372	-1.794829	H	-1.172741	2.574322	-1.649965	H	-1.131933	2.604311	-1.615817
H	-2.134908	2.562184	-0.189462	H	-1.603074	2.541998	-1.604604	H	-2.137289	2.561461	-0.188145	H	-2.108673	2.604372	-0.161444
H	-2.791683	0.936223	-1.980237	H	-2.634415	2.067748	0.600266	H	-2.797453	0.934145	-1.976796	H	-2.811403	0.997516	-1.931852
H	-1.284509	0.075353	-1.785366	H	-3.368233	1.157323	-0.711342	H	-1.289838	0.07367	-1.784529	H	-1.317035	0.110532	-1.784098
H	-3.484117	0.420005	0.299318	H	-1.37797	0.100034	1.331445	H	-3.483866	0.419943	0.305838	H	-3.399856	0.506565	0.406351
H	-0.611992	-2.428008	1.471889	H	-0.741118	-2.256392	1.223688	H	-0.614227	-2.431728	1.470915	H	-0.641974	-2.577472	1.239549
H	-1.511601	-2.748205	0.005627	H	-1.051155	-3.161826	-0.249101	H	-1.510525	-2.74589	0.001257	H	-1.541265	-2.68932	-0.264164
H	0.963321	-2.542607	-0.276696	H	1.20913	-2.527413	-0.301593	H	0.96049	-2.549158	-0.260234	H	0.975708	-2.51816	-0.449973
H	0.072934	-1.512444	-1.358987	H	0.406268	-1.585524	-1.533583	H	0.081976	-1.516791	-1.35594	H	0.091073	-1.427959	-1.479165
H	1.019463	2.734584	1.660965	H	1.129051	3.291333	0.862868	H	1.024294	2.735981	1.652294	H	1.008744	2.659662	1.749708
H	-0.70117	3.368993	1.465847	H	-0.617153	3.760251	0.501215	H	-0.695801	3.373021	1.461206	H	-0.708444	3.304956	1.560787
H	3.529363	-2.011289	0.954248	H	3.304493	-1.904301	1.553584	H	3.565253	-1.992788	0.950373	H	3.502014	-2.027817	0.955295
H	4.657048	-0.688021	0.616183	H	4.489718	-0.601181	1.364658	H	4.660238	-0.638748	0.624693	H	4.630283	-0.68599	0.700332
H	3.452539	-0.511261	1.901467	H	3.017946	-0.329614	2.319063	H	3.436338	-0.508505	1.905065	H	3.358553	-0.553721	1.931568
H	3.756418	-0.66756	-1.683643	H	2.956953	-0.719408	-1.738965	H	2.921398	-1.776125	-1.416607	H	2.553301	-0.50089	-2.065638
H	-2.705732	0.362913	2.416277	H	-3.842068	-1.04199	-1.272448	H	-2.700293	0.361744	2.420893	H	-2.62058	0.183645	2.488508
H	-1.428845	-0.860338	2.951295	H	-2.866925	-2.567561	-1.634743	H	-1.421488	-0.861184	2.951965	H	-1.36517	-1.113559	2.882909
H	-2.958123	-1.620187	-1.551195	H	-3.374738	-0.982748	1.976667	H	-2.966076	-1.617052	-1.549735	H	-4.005671	-1.629456	-0.28064

**9c_11**				**9c_12**				**9c_13**				**9c_14**			
C	-2.638708	-0.260361	-0.3222	C	-2.645997	-0.242839	-0.322205	C	-2.617466	-0.235345	-0.317879	C	-2.623766	-0.217751	-0.317844
C	-2.363346	1.268233	-0.284252	C	-2.363579	1.275123	-0.261001	C	-2.309598	1.286276	-0.294066	C	-2.310628	1.293698	-0.26707
H	-0.637398	-0.508427	-1.037948	H	-0.636794	-0.503025	-1.049638	H	-0.630874	-0.541117	-1.050973	H	-0.629425	-0.533799	-1.063043
H	-1.325805	0.918669	1.57826	H	-1.304676	0.904562	1.584738	H	-1.256521	0.930551	1.558331	H	-1.233573	0.915051	1.566514
C	0.930649	1.835267	-0.933545	C	0.93704	1.835597	-0.932239	C	0.99102	1.821609	-0.973548	C	0.996549	1.822031	-0.972073
C	0.134218	1.871206	0.360105	C	0.141165	1.870326	0.361545	C	0.194356	1.868411	0.319446	C	0.201166	1.867821	0.321505
C	2.283228	1.092495	-0.867987	C	2.287651	1.089114	-0.866829	C	2.321891	1.033115	-0.918543	C	2.324962	1.0291	-0.918164
C	2.24748	-0.438892	-0.817585	C	2.248192	-0.442099	-0.816832	C	2.197392	-0.491026	-0.806978	C	2.196258	-0.494599	-0.806633
C	1.862625	-1.094296	0.513835	C	1.856853	-1.097134	0.512929	C	1.8106	-1.060615	0.558384	C	1.805077	-1.063176	0.557917
C	0.778979	-2.158464	0.486564	C	0.774947	-2.16281	0.479014	C	0.778971	-2.170301	0.561242	C	0.775056	-2.174253	0.556346
C	-0.652735	-1.664049	0.792204	C	-0.659101	-1.668028	0.774917	C	-0.67046	-1.680543	0.793941	C	-0.676861	-1.684159	0.775582
C	-1.11947	-0.482425	-0.05654	C	-1.118081	-0.47949	-0.067706	C	-1.101147	-0.490858	-0.065228	C	-1.099075	-0.487062	-0.076801
C	-1.05913	0.96814	0.520069	C	-1.050263	0.964449	0.523967	C	-1.001642	0.967669	0.496784	C	-0.992623	0.96401	0.502093
C	0.463593	2.727653	1.332682	C	0.468777	2.728474	1.333151	C	0.517845	2.743981	1.276813	C	0.523826	2.744713	1.277866
C	-3.254617	-0.865571	-1.572358	C	-3.265156	-0.832294	-1.578066	C	-3.258288	-0.836068	-1.557707	C	-3.267163	-0.799259	-1.565315
O	-3.353323	-0.685916	0.846929	O	-3.436622	-0.568671	0.831066	O	-3.331206	-0.636279	0.860954	O	-3.410701	-0.521902	0.844245
C	2.537325	-0.81416	1.630494	C	2.524573	-0.817145	1.633699	C	2.408949	-0.652352	1.679271	C	2.398553	-0.654776	1.681226
O	3.581638	-0.847427	-1.196717	O	3.582853	-0.853332	-1.190966	O	3.415921	-1.131371	-1.248615	O	3.41385	-1.138394	-1.24557

H	-3.114484	1.894748	0.202388	H	-3.113333	1.887122	0.243827	H	-3.041909	1.931666	0.196653	H	-3.041918	1.922257	0.244513
H	-2.151065	1.664044	-1.280866	H	-2.165209	1.683694	-1.254577	H	-2.100912	1.670912	-1.295849	H	-2.117301	1.694341	-1.264906
H	1.152443	2.864796	-1.228448	H	1.16193	2.865124	-1.224698	H	1.240506	2.84631	-1.262588	H	1.249605	2.846549	-1.258569
H	0.327926	1.413747	-1.744526	H	0.333571	1.417294	-1.744334	H	0.376766	1.419096	-1.786247	H	0.380929	1.423348	-1.785644
H	2.840588	1.339717	-1.777289	H	2.846132	1.335185	-1.775734	H	2.858362	1.220652	-1.853963	H	2.861774	1.215233	-1.853656
H	2.869455	1.472139	-0.026413	H	2.87447	1.466989	-0.024851	H	2.948017	1.427781	-0.112194	H	2.952488	1.42174	-0.11187
H	1.550496	-0.790373	-1.589464	H	1.553207	-0.791742	-1.591309	H	1.462438	-0.818591	-1.545865	H	1.461987	-0.820449	-1.54688
H	0.778983	-2.637784	-0.499532	H	0.780781	-2.641475	-0.50716	H	0.829182	-2.702613	-0.394913	H	0.832666	-2.708466	-0.398109
H	1.027	-2.939125	1.211793	H	1.018395	-2.9435	1.20558	H	1.021333	-2.899361	1.339988	H	1.011575	-2.901236	1.338644
H	-1.336238	-2.507492	0.645689	H	-1.334351	-2.518165	0.613485	H	-1.346465	-2.522406	0.608327	H	-1.343746	-2.531958	0.57265
H	-0.721839	-1.397169	1.8514	H	-0.738431	-1.412045	1.836219	H	-0.793274	-1.4187	1.84922	H	-0.812433	-1.433808	1.832411
H	-0.077791	2.754172	2.273021	H	-0.071651	2.753955	2.274067	H	-0.021481	2.782273	2.217968	H	-0.013923	2.782046	2.219965
H	1.286077	3.428231	1.223134	H	1.288852	3.431619	1.222159	H	1.334704	3.448765	1.153288	H	1.338421	3.451758	1.152424
H	-3.24636	-1.957825	-1.510487	H	-3.287268	-1.926567	-1.521992	H	-3.272256	-1.927819	-1.48858	H	-3.311413	-1.89264	-1.505465
H	-4.29511	-0.541186	-1.684932	H	-4.293645	-0.478903	-1.695239	H	-4.292808	-0.490597	-1.662165	H	-4.289204	-0.424909	-1.673484
H	-2.70838	-0.563021	-2.469319	H	-2.696533	-0.552422	-2.468307	H	-2.714911	-0.551032	-2.462119	H	-2.701847	-0.534091	-2.462168
H	-4.258506	-0.363394	0.767052	H	-3.486097	-1.529664	0.894075	H	-4.228738	-0.291771	0.787176	H	-3.478045	-1.481401	0.913457
H	3.324191	-0.068148	1.653792	H	3.310605	-0.070494	1.662115	H	3.123867	0.163128	1.694206	H	3.1131	0.160916	1.698845
H	2.327591	-1.328639	2.562956	H	2.309676	-1.33221	2.564644	H	2.196087	-1.122354	2.63424	H	2.181875	-1.124721	2.635346
H	3.661167	-1.7896	-1.010864	H	3.658407	-1.796778	-1.009936	H	4.056622	-1.037293	-0.534246	H	4.055232	-1.041425	-0.53218

**9c_15**				**9c_16**				**9c_17**				**9c_18**			
C	-2.701502	-0.285606	-0.145628	C	2.719538	-0.214054	-0.166061	C	2.724124	-0.208705	-0.161817	C	-2.691265	-0.000355	0.127924
C	-2.500815	1.214788	0.183349	C	2.329675	1.256437	-0.464666	C	2.329505	1.243329	-0.505668	C	-2.187296	-1.447647	-0.129207
H	-0.833219	-0.267474	-1.24331	H	1.210952	-0.225666	1.380946	H	1.162895	-0.248408	1.352441	H	-0.933993	0.288395	1.298934
H	-1.004461	0.670562	1.663754	H	0.507564	0.605896	-1.462255	H	0.489629	0.61303	-1.489786	H	-0.907853	-0.583132	-1.629357
C	1.40313	1.685735	1.019749	C	-1.468475	2.085644	-0.536877	C	-1.47038	2.092669	-0.526817	C	1.140169	-1.719705	0.96028
C	0.06117	1.924369	0.33378	C	-0.181123	1.780194	0.206565	C	-0.169013	1.786436	0.191131	C	0.451333	-1.637102	-0.3889
C	2.535582	1.225919	0.080449	C	-2.291324	0.900053	-1.071249	C	-2.304074	0.909477	-1.049234	C	1.689954	-0.426064	1.608116
C	2.227259	-0.06486	-0.678295	C	-2.819148	-0.097595	-0.035416	C	-2.81989	-0.089466	-0.008551	C	2.537993	0.522084	0.745556
C	1.974819	-1.291223	0.194398	C	-1.786884	-1.002256	0.617274	C	-1.786576	-1.012094	0.617412	C	1.781091	1.319972	-0.311894
C	0.831037	-2.188151	-0.218539	C	-0.888184	-1.807616	-0.306719	C	-0.909899	-1.81272	-0.331117	C	0.667803	2.225738	0.197059
C	-0.557079	-1.75871	0.312063	C	0.611607	-1.82396	0.047399	C	0.601899	-1.819883	-0.02209	C	-0.764376	1.817329	-0.216911
C	-1.140763	-0.442097	-0.207496	C	1.254707	-0.4563	0.313838	C	1.238062	-0.460887	0.283401	C	-1.19934	0.431842	0.24879
C	-1.034273	0.91789	0.595144	C	0.823327	0.851416	-0.446583	C	0.820197	0.850797	-0.476539	C	-0.818176	-0.841637	-0.572044
C	-0.117585	3.009201	-0.42561	C	0.062422	2.367074	1.382267	C	0.099851	2.37787	1.359226	C	0.92464	-2.34845	-1.418019
C	-3.490142	-0.657982	-1.385619	C	3.857912	-0.485349	0.804085	C	3.821967	-0.44136	0.863613	C	-3.619893	0.256575	1.303093
O	-3.30145	-1.016256	0.93344	O	2.914598	-0.946756	-1.385558	O	3.046353	-0.858309	-1.400131	O	-3.225825	0.581105	-1.070556
C	2.756584	-1.585994	1.235973	C	-1.754744	-1.126324	1.945403	C	-1.7391	-1.157811	1.942972	C	2.134602	1.305637	-1.596971
O	3.256471	-0.355607	-1.646278	O	-3.769759	-0.91223	-0.757697	O	-3.792753	-0.890127	-0.717438	O	3.627048	-0.237923	0.223989
H	-3.159172	1.636139	0.946659	H	2.71566	1.674149	-1.397487	H	2.719952	1.613797	-1.455233	H	-2.733175	-2.038592	-0.868549
H	-2.568455	1.824448	-0.719209	H	2.583799	1.923845	0.360412	H	2.59368	1.939621	0.290976	H	-2.104326	-2.015635	0.801074
H	1.280332	0.950568	1.819342	H	-1.214768	2.704331	-1.408462	H	-1.232922	2.713241	-1.401637	H	1.964947	-2.429017	0.863745
H	1.723084	2.615091	1.500207	H	-2.103558	2.707177	0.102334	H	-2.093095	2.713038	0.125549	H	0.445128	-2.158732	1.688601
H	2.732406	2.002112	-0.665267	H	-3.172549	1.307642	-1.576226	H	-3.192197	1.32057	-1.539044	H	0.885635	0.157051	2.055554
H	3.457445	1.10554	0.660528	H	-1.731892	0.362127	-1.84054	H	-1.758629	0.372456	-1.829224	H	2.324671	-0.738878	2.443241
H	1.344693	0.11187	-1.29532	H	-3.342248	0.459006	0.75398	H	-3.321812	0.466413	0.794857	H	2.931751	1.277923	1.446059
H	0.793068	-2.242755	-1.313214	H	-1.2437	-2.843854	-0.333403	H	-1.25831	-2.851334	-0.347068	H	0.716517	2.293241	1.28902
H	1.021605	-3.203091	0.142618	H	-1.004853	-1.443136	-1.328721	H	-1.059304	-1.449248	-1.348929	H	0.846853	3.240001	-0.173623
H	-1.261881	-2.554118	0.044718	H	0.775081	-2.45946	0.924429	H	0.802952	-2.493075	0.818347	H	-1.454007	2.563529	0.195835
H	-0.523956	-1.745343	1.406969	H	1.137266	-2.304658	-0.781037	H	1.096981	-2.260773	-0.893103	H	-0.860654	1.878848	-1.304726
H	-1.054944	3.229675	-0.92152	H	0.986285	2.213045	1.93019	H	1.034534	2.224517	1.888648	H	0.444798	-2.326625	-2.39183
H	0.684538	3.725899	-0.57352	H	-0.666972	3.022402	1.846513	H	-0.618291	3.037159	1.835266	H	1.807991	-2.97151	-1.322286
H	-3.420943	-1.733854	-1.572518	H	3.928615	-1.555456	1.019499	H	3.918573	-1.508435	1.094257	H	-3.766339	1.331246	1.445951
H	-4.545599	-0.402518	-1.254169	H	4.813896	-0.161537	0.377415	H	4.783171	-0.088452	0.478237	H	-4.600825	-0.197507	1.124166
H	-3.109721	-0.129138	-2.261724	H	3.711313	0.050157	1.745559	H	3.607229	0.084872	1.796922	H	-3.216189	-0.166262	2.226543
H	-2.887998	-0.749795	1.761806	H	3.724346	-0.617336	-1.792502	H	3.236832	-1.784609	-1.211439	H	-4.064514	0.14286	-1.255958
H	3.563738	-0.938323	1.561667	H	-2.42093	-0.552283	2.581394	H	-2.389819	-0.585827	2.596624	H	2.913989	0.651092	-1.967351
H	2.613703	-2.498616	1.805848	H	-1.058123	-1.790981	2.446205	H	-1.044992	-1.839719	2.423901	H	1.632732	1.932568	-2.327047
H	4.031046	-0.651644	-1.153958	H	-4.10974	-1.567718	-0.137774	H	-4.125902	-1.547257	-0.095542	H	4.233663	0.37439	-0.206696

**9d_1**				**9d_2**				**9d_3**				**9d_4**			
C	2.792681	0.185485	-0.145526	C	2.796677	0.17997	-0.140422	C	2.798443	0.185558	-0.148145	C	2.805151	0.173189	-0.113721
C	2.395959	-1.083655	0.649265	C	2.393101	-1.079221	0.648079	C	2.396014	-1.068926	0.662884	C	2.378937	-1.054039	0.730943
H	1.123239	0.634887	-1.550378	H	1.117525	0.633954	-1.555683	H	1.118828	0.620308	-1.566418	H	1.178503	0.559504	-1.58962
H	1.157922	-1.690213	-1.02311	H	1.161577	-1.690841	-1.02779	H	1.172276	-1.700295	-1.00946	H	1.207695	-1.739896	-0.956665
C	-1.38875	-1.872329	-0.348463	C	-1.389694	-1.870397	-0.358004	C	-1.382948	-1.874405	-0.348582	C	-1.355176	-1.899355	-0.384975

C	-0.22093	-1.565103	0.571809	C	-0.221347	-1.569003	0.563661	C	-0.218085	-1.563855	0.574361	C	-0.232994	-1.549415	0.573032
C	-1.761872	-0.802432	-1.391297	C	-1.763423	-0.795401	-1.395306	C	-1.759635	-0.806005	-1.391514	C	-1.781627	-0.815766	-1.395065
C	-2.629633	0.35823	-0.883705	C	-2.629579	0.36411	-0.882154	C	-2.627818	0.354257	-0.883522	C	-2.677279	0.316331	-0.851179
C	-2.007538	1.222473	0.202194	C	-2.006802	1.224123	0.206717	C	-2.003675	1.22322	0.197501	C	-2.018539	1.266695	0.138918
C	-0.768537	2.018723	-0.174572	C	-0.768023	2.022374	-0.166514	C	-0.769148	2.022859	-0.186477	C	-0.759553	1.989817	-0.317032
C	0.595545	1.499552	0.348552	C	0.59547	1.494478	0.348752	C	0.598291	1.503214	0.326091	C	0.598395	1.505156	0.254763
C	1.282655	0.417278	-0.489876	C	1.277997	0.416072	-0.495282	C	1.279193	0.412132	-0.504338	C	1.307118	0.388244	-0.516458
C	1.043894	-1.103744	-0.10467	C	1.043281	-1.103825	-0.110172	C	1.048347	-1.102643	-0.099781	C	1.056312	-1.112643	-0.071361
C	-0.300535	-1.799973	1.883943	C	-0.301067	-1.811718	1.87443	C	-0.302749	-1.796107	1.886673	C	-0.371642	-1.721974	1.889833
C	3.652487	-0.094725	-1.374383	C	3.659472	-0.094646	-1.367581	C	3.653738	-0.111118	-1.371188	C	3.701812	-0.169976	-1.299755
O	3.358888	1.250611	0.616558	O	3.42735	1.140933	0.702949	O	3.480072	1.218478	0.557453	O	3.349245	1.275578	0.609879
C	-2.57203	1.352854	1.403706	C	-2.570667	1.349631	1.409081	C	-2.562937	1.355314	1.401164	C	-2.579043	1.546532	1.316522
O	-3.88137	-0.215416	-0.498274	O	-3.881389	-0.21009	-0.497751	O	-3.876368	-0.220919	-0.490709	O	-3.920965	-0.209311	-0.381917
H	2.278033	-0.824883	1.702747	H	2.270872	-0.818249	1.700553	H	2.262929	-0.808843	1.716267	H	2.221727	-0.742965	1.765061
H	3.034918	-1.96609	0.562919	H	3.036179	-1.958891	0.568162	H	3.040383	-1.948944	0.597794	H	3.019416	-1.939381	0.713194
H	-2.27256	-2.121616	0.243212	H	-2.273162	-2.1226	0.232974	H	-2.266432	-2.127368	0.241958	H	-2.224373	-2.249746	0.180561
H	-1.122707	-2.7804	-0.906233	H	-1.124389	-2.775655	-0.920694	H	-1.112623	-2.781198	-0.906333	H	-1.021709	-2.764253	-0.973112
H	-2.339904	-1.283011	-2.186216	H	-2.343347	-1.272113	-2.191206	H	-2.339214	-1.288587	-2.184081	H	-2.359086	-1.302683	-2.186389
H	-0.867366	-0.399193	-1.869877	H	-0.869288	-0.391145	-1.873545	H	-0.866922	-0.402459	-1.873013	H	-0.902985	-0.385607	-1.880785
H	-2.792658	1.022989	-1.747445	H	-2.792837	1.032176	-1.743306	H	-2.795867	1.016044	-1.748532	H	-2.958914	0.927256	-1.718179
H	-0.713092	2.13246	-1.263484	H	-0.715021	2.145155	-1.254547	H	-0.719091	2.136755	-1.275427	H	-0.706328	1.994952	-1.411622
H	-0.898288	3.028599	0.22549	H	-0.895241	3.028797	0.242964	H	-0.896884	3.032051	0.215609	H	-0.871823	3.036488	-0.020422
H	1.275216	2.355036	0.371088	H	1.276577	2.35021	0.380092	H	1.277603	2.361979	0.327853	H	1.274079	2.363838	0.245276
H	0.482639	1.174229	1.38697	H	0.484982	1.163686	1.385766	H	0.489497	1.191908	1.370718	H	0.470586	1.233954	1.306842
H	-1.224814	-2.145607	2.33545	H	-1.225488	-2.159665	2.323902	H	-1.228952	-2.140976	2.33471	H	-1.312518	-2.055816	2.315139
H	0.540684	-1.658952	2.552092	H	0.540105	-1.675436	2.543645	H	0.535033	-1.655357	2.559233	H	0.436525	-1.538633	2.587906
H	3.741519	0.808362	-1.984402	H	3.771511	0.814917	-1.967984	H	3.756362	0.789609	-1.982761	H	3.810725	0.701143	-1.951456
H	4.657678	-0.409822	-1.073261	H	4.653381	-0.431703	-1.060244	H	4.650781	-0.436144	-1.061585	H	4.69694	-0.469697	-0.953147
H	3.226847	-0.889309	-1.992774	H	3.217041	-0.862108	-2.007748	H	3.209552	-0.895754	-1.987319	H	3.293357	-0.994444	-1.889824
H	4.281179	1.029087	0.790174	H	3.714191	1.883112	0.157774	H	3.044898	1.349036	1.406661	H	4.262849	1.059944	0.830532
H	-2.153929	2.018788	2.151769	H	-2.152262	2.012704	2.159512	H	-2.143739	2.024686	2.145529	H	-2.130105	2.26527	1.994617
H	-3.446817	0.782159	1.690571	H	-3.44525	0.777745	1.694156	H	-3.434912	0.782968	1.69314	H	-3.499073	1.076575	1.640855
H	-4.468254	0.505135	-0.243439	H	-4.467713	0.509917	-0.240055	H	-4.465324	0.499138	-0.239251	H	-3.742945	-0.742718	0.400751

**9d_5**				**9d_6**				**9d_7**				**9d_8**			
C	2.809526	0.166278	-0.106646	C	2.784647	0.135868	-0.093286	C	2.796297	0.126422	-0.098437	C	2.811108	0.173368	-0.114746
C	2.375139	-1.053089	0.72708	C	2.459468	-1.381089	-0.15092	C	2.45482	-1.378591	-0.049961	C	2.378102	-1.038512	0.74438
H	1.177102	0.556286	-1.597594	H	1.433981	0.694909	-1.710074	H	1.393875	0.669454	-1.703452	H	1.176004	0.543823	-1.605398
H	1.208682	-1.741042	-0.96365	H	0.715699	-1.49769	-1.485967	H	0.757727	-1.545395	-1.435196	H	1.22159	-1.748692	-0.941951
C	-1.357256	-1.897623	-0.395059	C	-1.43276	-1.89888	-0.149458	C	-1.442042	-1.892369	-0.163952	C	-1.348485	-1.902226	-0.385916
C	-0.234497	-1.552207	0.563912	C	-0.109716	-1.479234	0.468835	C	-0.131998	-1.48647	0.488306	C	-0.230341	-1.548004	0.575239
C	-1.786073	-0.809066	-1.39888	C	-2.020668	-0.96887	-1.228479	C	-1.995502	-0.94769	-1.248463	C	-1.780055	-0.819848	-1.395195
C	-2.679605	0.321177	-0.847609	C	-2.795752	0.25708	-0.723453	C	-2.778807	0.274264	-0.746599	C	-2.675925	0.311329	-0.849771
C	-2.017226	1.269321	0.142216	C	-1.994218	1.22239	0.132758	C	-1.993522	1.222668	0.140966	C	-2.015136	1.267405	0.133632
C	-0.759422	1.993007	-0.316052	C	-0.811945	1.910647	-0.535335	C	-0.802058	1.925275	-0.491869	C	-0.761217	1.993391	-0.331265
C	0.598871	1.5019	0.249064	C	0.58412	1.570374	0.049993	C	0.591331	1.548779	0.078234	C	0.60084	1.509091	0.230066
C	1.303838	0.386324	-0.523857	C	1.356538	0.452372	-0.645404	C	1.34834	0.432175	-0.635792	C	1.304304	0.383046	-0.530734
C	1.054778	-1.113467	-0.078969	C	0.972568	-1.083302	-0.509085	C	0.978685	-1.103089	-0.461311	C	1.060569	-1.11086	-0.066227
C	-0.373666	-1.72949	1.880106	C	0.080223	-1.518655	1.790091	C	0.021317	-1.523457	1.811986	C	-0.374462	-1.718041	1.891814
C	3.712727	-0.16741	-1.28933	C	3.96301	0.627623	-0.921694	C	3.947078	0.551222	-0.999351	C	3.703412	-0.186212	-1.294
O	3.413608	1.165616	0.71083	O	2.892296	0.61545	1.254345	O	3.023592	0.571495	1.245644	O	3.473681	1.239131	0.558596
C	-2.573718	1.547039	1.322217	C	-2.353121	1.515522	1.383168	C	-2.369113	1.486609	1.375525	C	-2.570489	1.550878	1.312715
O	-3.921073	-0.205998	-0.374228	O	-3.963993	-0.242846	-0.068862	O	-3.963573	-0.229447	-0.126114	O	-3.915595	-0.21556	-0.372019
H	2.214559	-0.742498	1.760807	H	2.623271	-1.905757	0.791587	H	2.594967	-1.810762	0.940901	H	2.205422	-0.726522	1.777792
H	3.018657	-1.935945	0.713358	H	2.985694	-1.913155	-0.946666	H	3.001398	-1.980495	-0.778607	H	3.023193	-1.920349	0.747756
H	-2.225538	-2.251761	0.169544	H	-2.177115	-2.062113	0.633738	H	-2.206515	-2.055792	0.599465	H	-2.216492	-2.258485	0.177673
H	-1.023897	-2.759012	-0.988354	H	-1.267137	-2.87326	-0.629067	H	-1.271656	-2.864114	-0.646999	H	-1.009294	-2.764227	-0.97493
H	-2.366094	-1.292318	-2.190602	H	-2.727863	-1.542478	-1.834784	H	-2.687809	-1.510418	-1.881398	H	-2.359249	-1.308879	-2.183944
H	-0.908554	-0.37716	-1.884943	H	-1.238542	-0.634608	-1.91427	H	-1.193108	-0.607071	-1.907292	H	-0.903808	-0.388839	-1.884335
H	-2.965291	0.934375	-1.711685	H	-3.101603	0.819268	-1.620975	H	-3.06145	0.850112	-1.64297	H	-2.963709	0.918706	-1.717232
H	-0.710338	2.002131	-1.410764	H	-0.820291	1.715548	-1.612891	H	-0.808645	1.776581	-1.57666	H	-0.713729	1.997653	-1.425908
H	-0.868802	3.038556	-0.014447	H	-0.961927	2.989462	-0.430217	H	-0.940324	2.999987	-0.340086	H	-0.871899	3.039761	-0.033541
H	1.275038	2.361891	0.243945	H	1.209452	2.466996	-0.024472	H	1.223345	2.443073	0.008457	H	1.275547	2.370702	0.198138
H	0.474495	1.22951	1.301252	H	0.488099	1.356478	1.114942	H	0.497136	1.323256	1.141991	H	0.477122	1.25382	1.288094
H	-1.314947	-2.064174	2.303865	H	-0.723742	-1.794542	2.464766	H	-0.803158	-1.789173	2.465409	H	-1.316858	-2.05239	2.313275
H	0.43423	-1.549691	2.579411	H	1.029871	-1.253911	2.240645	H	0.961436	-1.273404	2.290082	H	0.429613	-1.533755	2.594382
H	3.847921	0.713373	-1.9267	H	4.003379	1.720771	-0.915657	H	4.013526	1.643532	-1.055762	H	3.828262	0.682955	-1.945682

H	4.694505	-0.491349	-0.932907	H	4.906662	0.249138	-0.512791	H	4.894776	0.170632	-0.607156	H	4.689025	-0.497641	-0.937533
H	3.28955	-0.962817	-1.907939	H	3.886577	0.28841	-1.958102	H	3.813252	0.170601	-2.015222	H	3.275589	-0.999204	-1.884182
H	3.722116	1.879975	0.140908	H	3.703075	0.248061	1.625805	H	3.130404	1.529808	1.230776	H	3.015252	1.411192	1.387877
H	-2.122388	2.264352	2.000241	H	-1.802796	2.241752	1.972575	H	-1.82958	2.201484	1.987691	H	-2.120873	2.274447	1.985203
H	-3.492659	1.076489	1.648733	H	-3.184691	1.020992	1.869903	H	-3.207938	0.981034	1.837217	H	-3.487128	1.079016	1.643644
H	-3.739126	-0.742291	0.405558	H	-4.50084	0.513814	0.191724	H	-4.500763	0.526192	0.136881	H	-3.731615	-0.750486	0.408229

**9d_9**				**9e_1**				**9e_2**				**9e_3**			
C	2.605657	-0.446327	-0.058646	C	-2.802819	0.144997	-0.176335	C	-2.794673	0.155902	-0.183867	C	-2.815533	0.132064	-0.142583
C	2.51656	0.655363	1.03283	C	-2.382144	-1.08779	0.647616	C	-2.382621	-1.081898	0.651559	C	-2.365983	-1.060978	0.724341
H	0.997504	-1.56244	0.849922	H	-1.137345	0.615351	-1.566184	H	-1.143082	0.609495	-1.56907	H	-1.191862	0.543344	-1.602751
H	0.503486	0.445208	1.95111	H	-1.14893	-1.689888	-1.01638	H	-1.148932	-1.691427	-1.010423	H	-1.196506	-1.73668	-0.955056
C	-1.24178	1.95742	0.638621	C	1.407352	-1.863007	-0.343231	C	1.405942	-1.866073	-0.33587	C	1.374448	-1.890391	-0.381373
C	0.242331	1.789088	0.334016	C	0.238302	-1.557963	0.576142	C	0.23722	-1.557588	0.582829	C	0.24986	-1.543463	0.574895
C	-2.221511	1.306313	-0.374912	C	1.782015	-0.790328	-1.382142	C	1.780355	-0.796827	-1.378493	C	1.802915	-0.803377	-1.386441
C	-2.873745	-0.001585	0.083915	C	2.63821	0.374105	-0.86414	C	2.637609	0.368807	-0.864964	C	2.685117	0.333541	-0.830937
C	-1.945252	-1.167041	0.380022	C	1.994431	1.234937	0.211381	C	1.994263	1.23467	0.206774	C	2.005821	1.280021	0.148888
C	-1.106475	-1.741181	-0.743829	C	0.759768	2.025309	-0.190092	C	0.760429	2.024375	-0.198713	C	0.751557	1.996089	-0.329568
C	0.213567	-1.001011	-1.070805	C	-0.613137	1.514832	0.32012	C	-0.612919	1.517767	0.313245	C	-0.615261	1.518822	0.229066
C	1.088366	-0.729316	0.14648	C	-1.285996	0.411017	-0.50126	C	-1.283812	0.410002	-0.503403	C	-1.309856	0.382918	-0.526474
C	0.960568	0.619142	0.974781	C	-1.027529	-1.10404	-0.10009	C	-1.027697	-1.103678	-0.095322	C	-1.039374	-1.112693	-0.070121
C	0.847383	2.678588	-0.4592	C	0.318051	-1.796103	1.88804	C	0.317198	-1.791418	1.895462	C	0.386753	-1.720984	1.891607
C	3.570485	-1.5995	0.176564	C	-3.650846	1.212513	0.49552	C	-3.644781	1.223025	0.485575	C	-3.644695	1.229803	0.50363
O	2.804997	0.10265	-1.368932	O	-3.474246	-0.347041	-1.349622	O	-3.39368	-0.236162	-1.432013	O	-3.521908	-0.415912	-1.269529
C	-1.947377	-1.709495	1.601311	C	2.537063	1.369464	1.422239	C	2.536589	1.373839	1.41724	C	2.546313	1.565368	1.334355
O	-3.796604	-0.354657	-0.965936	O	3.886629	-0.1927	-0.459346	O	3.885444	-0.197406	-0.457515	O	3.924518	-0.184276	-0.342773
H	3.000808	1.600962	0.783106	H	-3.022052	-1.96772	0.556336	H	-3.019385	-1.966407	0.570034	H	-3.007833	-1.943815	0.696145
H	2.892037	0.314916	2.000566	H	-2.276089	-0.826324	1.701958	H	-2.273652	-0.81753	1.705537	H	-2.223068	-0.752016	1.761359
H	-1.453661	3.029074	0.660432	H	1.141239	-2.769173	-0.904016	H	1.139438	-2.774019	-0.893592	H	1.041804	-2.752995	-0.973238
H	-1.458482	1.582302	1.644387	H	2.290481	-2.114054	0.24874	H	2.289241	-2.115328	0.256601	H	2.242734	-2.242696	0.184504
H	-1.730193	1.153034	-1.338075	H	2.368616	-1.266374	-2.173513	H	2.36592	-1.275751	-2.16889	H	2.390876	-1.285382	-2.173044
H	-3.048832	1.996644	-0.56282	H	0.888308	-0.391488	-1.866131	H	0.886486	-0.398845	-1.86288	H	0.925565	-0.378711	-1.879439
H	-3.438283	0.212669	1.00124	H	2.810671	1.039229	-1.725757	H	2.810823	1.030229	-1.729259	H	2.975893	0.945943	-1.693859
H	-0.857597	-2.774578	-0.483631	H	0.884082	3.040721	0.197384	H	0.885518	3.041366	0.184288	H	0.859238	3.04639	-0.044454
H	-1.71842	-1.781553	-1.650402	H	0.716408	2.123931	-1.280845	H	0.717465	2.117974	-1.289877	H	0.709823	1.987965	-1.424398
H	0.772183	-1.628099	-1.773741	H	-1.281901	2.38131	0.303387	H	-1.281798	2.383851	0.290234	H	-1.281039	2.386258	0.181649
H	0.019311	-0.065702	-1.598199	H	-0.519879	1.220014	1.370281	H	-0.520987	1.229259	1.365313	H	-0.505379	1.276562	1.290962
H	0.299939	3.518861	-0.875453	H	-0.52378	-1.661003	2.556758	H	-0.524246	-1.65302	2.563983	H	-0.422781	-1.543805	2.589779
H	1.88672	2.584979	-0.744898	H	1.243054	-2.140378	2.339226	H	1.242037	-2.135232	2.347381	H	1.327556	-2.054769	2.317304
H	3.429608	-2.375509	-0.581679	H	-3.790243	2.077264	-0.162347	H	-3.764714	2.090951	-0.16882	H	-3.806266	2.061825	-0.190402
H	4.60799	-1.251408	0.116746	H	-4.637025	0.797185	0.721176	H	-4.640836	0.818479	0.696582	H	-4.622466	0.824867	0.779069
H	3.422646	-2.045982	1.163252	H	-3.205028	1.564062	1.428246	H	-3.214882	1.554797	1.433024	H	-3.171637	1.626328	1.404371
H	3.710993	0.43087	-1.408873	H	-3.555403	0.389621	-1.967064	H	-4.25489	-0.622495	-1.232536	H	-3.622405	0.289759	-1.919466
H	-1.331283	-2.568795	1.846018	H	3.410036	0.803656	1.723895	H	3.409038	0.808619	1.72153	H	3.463965	1.101119	1.673193
H	-2.566358	-1.30993	2.398637	H	2.102483	2.033136	2.162851	H	2.102382	2.041073	2.15487	H	2.082879	2.282194	2.004678
H	-4.259931	-1.152222	-0.685588	H	4.46691	0.531189	-0.198865	H	4.466334	0.527085	-0.2001	H	3.737535	-0.720613	0.435794

**9e_4**				**9e_5**				**9e_6**				**9e_7**			
C	-2.807774	0.142539	-0.150837	C	-2.808629	0.153133	-0.185213	C	-2.82177	0.141203	-0.15375	C	2.947657	-0.217942	-0.067172
C	-2.365495	-1.054597	0.727852	C	-2.395083	-1.099723	0.628218	C	-2.382871	-1.079052	0.695817	C	2.476004	1.00638	0.744887
H	-1.198416	0.538215	-1.605763	H	-1.14621	0.605636	-1.576541	H	-1.19933	0.538172	-1.611319	H	1.499126	-0.508049	-1.729093
H	-1.196493	-1.737181	-0.949999	H	-1.116524	-1.705715	-1.015285	H	-1.159372	-1.749966	-0.962529	H	1.519103	1.771313	-1.041904
C	1.373454	-1.893279	-0.37565	C	1.408815	-1.866378	-0.31028	C	1.378529	-1.894276	-0.343602	C	-1.164679	1.733199	-0.758896
C	0.249373	-1.54321	0.580102	C	0.232006	-1.5496	0.594971	C	0.241604	-1.535522	0.593463	C	-0.09209	1.573207	0.315894
C	1.802216	-0.808826	-1.383372	C	1.786051	-0.811128	-1.366258	C	1.808104	-0.826234	-1.368484	C	-2.593869	1.29472	-0.396032
C	2.68475	0.329243	-0.830649	C	2.643579	0.35978	-0.865602	C	2.689786	0.320577	-0.832873	C	-2.932295	-0.191538	-0.603993
C	2.004981	1.279422	0.145289	C	1.998879	1.236076	0.196513	C	2.010866	1.278396	0.135847	C	-2.000847	-1.178305	0.090149
C	0.752106	1.995101	-0.337447	C	0.764129	2.019512	-0.218167	C	0.756503	1.988385	-0.351109	C	-0.769024	-1.619163	-0.689267
C	-0.615619	1.522142	0.221719	C	-0.608992	1.516412	0.299043	C	-0.610538	1.517556	0.212733	C	0.614709	-1.440931	-0.001706
C	-1.307934	0.382437	-0.52913	C	-1.288379	0.407247	-0.509733	C	-1.31196	0.380212	-0.534233	C	1.493746	-0.364691	-0.644103
C	-1.039056	-1.111607	-0.066225	C	-1.028596	-1.107036	-0.100492	C	-1.040603	-1.116327	-0.075449	C	1.251515	1.133675	-0.192746
C	0.386391	-1.718115	1.897129	C	0.30136	-1.762622	1.911495	C	0.363017	-1.687664	1.914577	C	-0.307218	1.895638	1.59347
C	-3.638945	1.239418	0.493901	C	-3.633551	1.214382	0.515996	C	-3.624324	1.230428	0.530569	C	3.595928	-1.376732	0.672894
O	-3.44446	-0.309346	-1.359284	O	-3.545641	-0.178675	-1.373287	O	-3.595706	-0.236565	-1.303863	O	3.83515	0.274478	-1.085619
C	2.543888	1.568033	1.330683	C	2.539975	1.387709	1.406037	C	2.550553	1.576642	1.318499	C	-2.310051	-1.69234	1.281946
O	3.923364	-0.187763	-0.339587	O	3.890784	-0.202467	-0.451324	O	3.932328	-0.187118	-0.342407	O	-4.289501	-0.313271	-0.162978
H	-3.003465	-1.941738	0.710293	H	-3.030107	-1.982331	0.52076	H	-3.020636	-1.964514	0.641857	H	3.173401	1.84505	0.789364

H	-2.218987	-0.742384	1.764055	H	-2.299782	-0.85715	1.68842	H	-2.254825	-0.797023	1.742796	H	2.196451	0.714963	1.758921
H	1.040055	-2.757025	-0.965439	H	1.150536	-2.78414	-0.855733	H	1.058809	-2.773039	-0.918692	H	-0.843679	1.221089	-1.671156
H	2.241703	-2.244847	0.19075	H	2.288634	-2.102969	0.292334	H	2.243418	-2.22681	0.238959	H	-1.193542	2.79534	-1.028963
H	2.389912	-1.292985	-2.168836	H	2.372328	-1.300668	-2.149513	H	2.397143	-1.322395	-2.145349	H	-2.818805	1.539208	0.645206
H	0.92491	-0.385103	-1.877287	H	0.893765	-0.417527	-1.857473	H	0.9316	-0.410242	-1.87075	H	-3.305585	1.857055	-1.007857
H	2.976728	0.938643	-1.695275	H	2.817188	1.011738	-1.736936	H	2.975635	0.921209	-1.705619	H	-2.883554	-0.402921	-1.683241
H	0.860804	3.046379	-0.056395	H	0.887333	3.040693	0.154142	H	0.863631	3.041759	-0.077408	H	-0.902336	-2.68064	-0.922929
H	0.711392	1.982631	-1.432216	H	0.721589	2.101561	-1.310275	H	0.71506	1.968372	-1.445753	H	-0.754844	-1.116224	-1.659776
H	-1.28131	2.389104	0.167429	H	-1.276691	2.383277	0.274809	H	-1.274675	2.385769	0.161356	H	1.154073	-2.390457	-0.063891
H	-0.507808	1.286473	1.28535	H	-0.514479	1.230967	1.351635	H	-0.499276	1.28034	1.275574	H	0.472672	-1.239471	1.063726
H	-0.422657	-1.538015	2.595097	H	-0.546171	-1.619313	2.571054	H	-0.45539	-1.502775	2.600082	H	0.47731	1.834617	2.338124
H	1.326819	-2.052525	2.323206	H	1.224661	-2.0931	2.376113	H	1.300973	-2.00694	2.357097	H	-1.27352	2.239528	1.945096
H	-3.782066	2.074787	-0.197179	H	-3.758443	2.094241	-0.121543	H	-3.770168	2.084156	-0.137262	H	3.790529	-2.214602	-0.005417
H	-4.626612	0.844534	0.756097	H	-4.625694	0.810154	0.736031	H	-4.608597	0.835987	0.798328	H	4.552169	-1.049701	1.090762
H	-3.18012	1.616928	1.41012	H	-3.174512	1.525739	1.455234	H	-3.135988	1.578852	1.441578	H	2.969378	-1.741492	1.489591
H	-4.295739	-0.69163	-1.114423	H	-3.087092	-0.891178	-1.832052	H	-3.148191	-0.961958	-1.753219	H	3.969718	-0.438137	-1.721735
H	3.460591	1.104058	1.672447	H	3.413082	0.826832	1.716435	H	3.468267	1.116723	1.663004	H	-3.210887	-1.394067	1.804862
H	2.080095	2.28748	1.997948	H	2.104302	2.061452	2.136852	H	2.086521	2.300637	1.980671	H	-1.672411	-2.42154	1.771013
H	3.735406	-0.721121	0.440786	H	4.471753	0.524355	-0.200752	H	3.751033	-0.712787	0.444734	H	-4.536877	-1.241576	-0.243868

**9e_8**				**9f_1**				**9f_2**				**9f_3**			
C	2.94041	-0.228122	-0.077203	C	-2.664787	-0.343875	-0.045944	C	-2.656974	-0.347489	-0.043725	C	2.715121	-0.321349	-0.221609
C	2.474689	1.000131	0.747356	C	-2.47872	1.186398	-0.124992	C	-2.479404	1.195016	-0.116725	C	2.555915	1.191083	0.027176
H	1.505058	-0.50591	-1.73019	H	-0.975941	-0.373681	1.264622	H	-0.978264	-0.369557	1.265137	H	0.819724	-0.28442	-1.207014
H	1.518705	1.77061	-1.038419	H	-0.870794	0.842591	-1.530924	H	-0.875792	0.852907	-1.527568	H	1.202965	0.713181	1.655499
C	-1.164678	1.735476	-0.75509	C	1.354124	1.985054	-0.807446	C	1.355078	1.985173	-0.806931	C	-1.288386	1.613039	1.104479
C	-0.092103	1.573849	0.319557	C	0.095092	1.903463	0.039333	C	0.09792	1.90618	0.042865	C	0.018874	1.916721	0.380915
C	-2.593809	1.296388	-0.392743	C	2.006134	0.670805	-1.276691	C	2.002124	0.669429	-1.278933	C	-2.469151	1.251581	0.189621
C	-2.931917	-0.189611	-0.602962	C	2.731191	-0.150091	-0.204801	C	2.727794	-0.154242	-0.209582	C	-2.271984	-0.006978	-0.654629
C	-1.999558	-1.177044	0.089043	C	1.871265	-0.837234	0.845127	C	1.868941	-0.838436	0.843185	C	-2.05981	-1.293174	0.146878
C	-0.769237	-1.617757	-0.692917	C	0.965011	-1.985805	0.443352	C	0.958181	-1.98476	0.444899	C	-0.858225	-2.149698	-0.194031
C	0.615467	-1.444272	-0.006829	C	-0.324038	-1.655246	-0.353307	C	-0.330301	-1.652463	-0.351335	C	0.508771	-1.720061	0.392536
C	1.491688	-0.364868	-0.646224	C	-1.122393	-0.470393	0.184402	C	-1.124985	-0.464653	0.184697	C	1.152107	-0.452538	-0.177802
C	1.250638	1.132238	-0.189908	C	-0.976963	0.961703	-0.447411	C	-0.978704	0.969276	-0.443411	C	1.134767	0.926847	0.581705
C	-0.306709	1.896813	1.597066	C	-0.056618	2.684362	1.111794	C	-0.049056	2.68571	1.116896	C	0.153916	3.027639	-0.348254
C	3.593328	-1.384487	0.662387	C	-3.193876	-0.971472	-1.331305	C	-3.188927	-0.961999	-1.334047	C	3.471138	-1.050126	0.884545
O	3.761239	0.173282	-1.187901	O	-3.484992	-0.719612	1.062145	O	-3.377466	-0.854382	1.08074	O	3.315738	-0.587201	-1.489931
C	-2.307025	-1.691781	1.280972	C	2.006299	-0.487459	2.127128	C	2.009135	-0.488404	2.124588	C	-2.954672	-1.684637	1.056754
O	-4.288786	-0.312497	-0.161149	O	3.499363	-1.12912	-0.934548	O	3.490198	-1.135745	-0.941985	O	-3.450957	-0.099136	-1.480584
H	3.16678	1.843828	0.802133	H	-2.628845	1.620801	0.86437	H	-2.627148	1.624979	0.874922	H	2.532942	1.711975	-0.93161
H	2.191374	0.705576	1.760139	H	-3.073955	1.735043	-0.858133	H	-3.073032	1.747852	-0.848535	H	3.277792	1.675947	0.687738
H	-0.843678	1.224619	-1.668021	H	2.099881	2.595337	-0.288101	H	2.103838	2.59307	-0.289104	H	-1.130753	0.804081	1.823336
H	-1.193583	2.797986	-1.02367	H	1.08921	2.539019	-1.718252	H	1.089267	2.540509	-1.716656	H	-1.578535	2.490136	1.69122
H	-2.818552	1.539167	0.64894	H	1.284445	0.041089	-1.800111	H	1.277166	0.042101	-1.800671	H	-3.369462	1.12315	0.79877
H	-3.305681	1.859601	-1.00358	H	2.771252	0.928073	-2.015829	H	2.7659	0.92499	-2.020053	H	-2.667696	2.078778	-0.498796
H	-2.883833	-0.399241	-1.68256	H	3.418315	0.525799	0.321443	H	3.418935	0.519215	0.314523	H	-1.403527	0.142966	-1.306555
H	-0.904791	-2.67831	-0.929381	H	1.55022	-2.702352	-0.142071	H	1.540516	-2.704615	-0.139329	H	-1.054631	-3.168298	0.153085
H	-0.755068	-1.112251	-1.662055	H	0.667429	-2.500119	1.361761	H	0.659706	-2.495838	1.364812	H	-0.758778	-2.206372	-1.285956
H	1.155089	-2.393032	-0.076195	H	-0.947639	-2.556515	-0.318864	H	-0.958803	-2.54949	-0.31269	H	1.190805	-2.556846	0.201022
H	0.475743	-1.249269	1.060147	H	-0.09238	-1.500569	-1.410853	H	-0.099474	-1.500303	-1.409474	H	0.424828	-1.645404	1.482323
H	0.477628	1.834086	2.34178	H	-0.970009	2.695834	1.695734	H	-0.961502	2.699144	1.70226	H	1.07471	3.275698	-0.862583
H	-1.272392	2.242833	1.948395	H	0.739772	3.341154	1.446336	H	0.750107	3.339392	1.450934	H	-0.66342	3.734801	-0.451536
H	3.774825	-2.222468	-0.016246	H	-3.169142	-2.064516	-1.274404	H	-3.132478	-2.052627	-1.296573	H	3.453713	-2.133543	0.729677
H	4.557995	-1.063738	1.070835	H	-4.230567	-0.661889	-1.4879	H	-4.237731	-0.678618	-1.474124	H	4.514493	-0.723907	0.88448
H	2.979651	-1.735005	1.495042	H	-2.609377	-0.669969	-2.204015	H	-2.635072	-0.615835	-2.210101	H	3.04719	-0.847275	1.871114
H	4.599841	0.488922	-0.830379	H	-3.56379	-1.681017	1.063814	H	-4.320348	-0.790532	0.887241	H	3.3964	-1.543325	-1.589639
H	-3.207107	-1.393772	1.805347	H	1.435603	-0.970717	2.913551	H	1.439427	-0.969355	2.913139	H	-2.845669	-2.623132	1.590825
H	-1.668855	-2.421461	1.768619	H	2.68558	0.302432	2.431814	H	2.691759	0.299657	2.426559	H	-3.831971	-1.091411	1.29117
H	-4.535882	-1.240766	-0.243306	H	4.005446	-1.637224	-0.290195	H	3.996229	-1.646055	-0.299333	H	-3.399655	-0.926184	-1.972148

**9f_4**				**9f_5**				**9f_6**				**9f_7**			
C	2.708766	-0.322297	-0.220742	C	2.705749	-0.26256	-0.187572	C	-2.664227	-0.358588	-0.048901	C	2.699709	-0.26246	-0.186529
C	2.554541	1.202094	0.023947	C	2.486535	1.226635	0.132212	C	-2.487666	1.182992	-0.12001	C	2.484431	1.238993	0.12806
H	0.825949	-0.278826	-1.208754	H	0.854767	-0.255535	-1.265575	H	-0.968767	-0.364784	1.270913	H	0.863331	-0.247604	-1.270243
H	1.203148	0.723068	1.653015	H	1.046379	0.648068	1.651873	H	-0.88343	0.837441	-1.527901	H	1.045586	0.657653	1.647393
C	-1.291052	1.613902	1.103764	C	-1.392589	1.654784	1.071062	C	1.342501	1.982813	-0.816058	C	-1.395536	1.655854	1.070821
C	0.013567	1.918676	0.375766	C	-0.067728	1.904996	0.357357	C	0.086698	1.903615	0.035863	C	-0.074311	1.907056	0.350589

C	-2.473203	1.247753	0.19261	C	-2.544213	1.219195	0.144091	C	1.991978	0.667343	-1.28531	C	-2.549555	1.213721	0.149995
C	-2.273091	-0.010402	-0.651365	C	-2.244549	-0.049918	-0.65313	C	2.726547	-0.148639	-0.216088	C	-2.246593	-0.053917	-0.648189
C	-2.052297	-1.295326	0.149724	C	-1.979641	-1.297423	0.184615	C	1.87643	-0.827219	0.847418	C	-1.969925	-1.300124	0.187447
C	-0.850233	-2.148124	-0.198855	C	-0.840771	-2.181112	-0.266372	C	0.96766	-1.980287	0.46441	C	-0.831416	-2.17893	-0.274041
C	0.518213	-1.719542	0.384485	C	0.555621	-1.772115	0.26129	C	-0.323248	-1.660435	-0.332614	C	0.566639	-1.770481	0.248432
C	1.156977	-0.446812	-0.178971	C	1.147995	-0.447731	-0.228645	C	-1.124736	-0.472004	0.190618	C	1.153197	-0.44057	-0.232349
C	1.134182	0.93421	0.578766	C	1.040502	0.903955	0.585825	C	-0.986312	0.959575	-0.444762	C	1.039066	0.911953	0.581004
C	0.14329	3.026532	-0.35889	C	0.088192	2.997801	-0.395656	C	-0.06114	2.688265	1.10609	C	0.075059	2.995959	-0.409255
C	3.469039	-1.039051	0.890334	C	3.434935	-1.027855	0.912197	C	-3.174326	-0.972236	-1.344541	C	3.428478	-1.015132	0.922303
O	3.208981	-0.715657	-1.499183	O	3.373346	-0.43778	-1.438434	O	-3.503738	-0.877799	0.979648	O	3.275928	-0.569591	-1.45706
C	-2.939913	-1.688972	1.065719	C	-2.748129	-1.620984	1.227522	C	2.022859	-0.465915	2.125009	C	-2.727996	-1.626786	1.237003
O	-3.453901	-0.108504	-1.473994	O	-3.285148	-0.317717	-1.61556	O	3.485059	-1.133509	-0.947654	O	-3.290421	-0.328027	-1.605374
H	2.530622	1.720459	-0.936161	H	2.509315	1.797546	-0.797047	H	-2.632163	1.625534	0.868413	H	2.506305	1.806236	-0.803429
H	3.272424	1.690815	0.686545	H	3.152752	1.693322	0.860981	H	-3.085082	1.734288	-0.849627	H	3.145542	1.710718	0.858706
H	-1.581546	2.491683	1.689344	H	-1.699046	2.573388	1.580317	H	2.090404	2.594368	-0.301384	H	-1.70249	2.575291	1.578311
H	-1.129694	0.806896	1.823942	H	-1.253719	0.900805	1.85018	H	1.073531	2.534835	-1.726839	H	-1.251275	0.904827	1.851752
H	-3.371383	1.1165	0.804278	H	-3.454704	1.081333	0.738007	H	1.266712	0.034898	-1.800357	H	-3.456373	1.071349	0.748441
H	-2.67654	2.073757	-0.495833	H	-2.755958	2.01419	-0.577272	H	2.750873	0.922255	-2.031616	H	-2.769056	2.007103	-0.57081
H	-1.407278	0.143345	-1.305982	H	-1.369044	0.143984	-1.275554	H	3.42018	0.529265	0.298861	H	-1.375367	0.145226	-1.27508
H	-1.043366	-3.168497	0.144859	H	-1.0286	-3.20648	0.065563	H	1.550288	-2.704981	-0.113491	H	-1.01507	-3.206455	0.05352
H	-0.754469	-2.200398	-1.291283	H	-0.813707	-2.202708	-1.362456	H	0.673256	-2.482262	1.390671	H	-0.81024	-2.194938	-1.370313
H	1.202766	-2.550687	0.180594	H	1.241099	-2.572026	-0.044199	H	-0.949049	-2.559119	-0.28394	H	1.25507	-2.561204	-0.071979
H	0.439315	-1.653211	1.475236	H	0.538231	-1.790993	1.356546	H	-0.094803	-1.51767	-1.392411	H	0.556655	-1.799674	1.343593
H	1.062265	3.274666	-0.876388	H	1.012076	3.224705	-0.913429	H	-0.972586	2.703596	1.693031	H	0.996603	3.222632	-0.931272
H	-0.676574	3.730525	-0.463813	H	-0.71939	3.7135	-0.515829	H	0.736815	3.345716	1.435459	H	-0.735423	3.708132	-0.53087
H	3.423475	-2.122733	0.759806	H	3.450857	-2.102717	0.705967	H	-3.118727	-2.062709	-1.302275	H	3.422074	-2.091568	0.736133
H	4.521815	-0.737302	0.869534	H	4.468699	-0.677131	0.969688	H	-4.21966	-0.6884	-1.492204	H	4.471185	-0.682847	0.967301
H	3.075467	-0.791776	1.879274	H	2.967864	-0.882207	1.88936	H	-2.601111	-0.629299	-2.207954	H	2.982142	-0.8298	1.902393
H	4.171281	-0.65109	-1.479038	H	3.497553	-1.383177	-1.583878	H	-3.209261	-0.505881	1.818423	H	4.233301	-0.4755	-1.384531
H	-2.824587	-2.626995	1.599315	H	-2.597257	-2.547977	1.771613	H	1.45978	-0.942529	2.92096	H	-2.568875	-2.553345	1.779521
H	-3.817292	-1.098221	1.306076	H	-3.552402	-0.98363	1.579711	H	2.704651	0.32678	2.416524	H	-3.531638	-0.992652	1.596381
H	-3.399161	-0.934541	-1.966903	H	-4.052809	-0.628299	-1.121383	H	3.999484	-1.636552	-0.305957	H	-4.053523	-0.643458	-1.107206

**9f_8**				**9f_9**				**9f_10**				**9f_11**			
C	2.719889	-0.328481	-0.217735	C	2.631457	-0.248078	-0.372329	C	-2.658944	-0.344926	-0.053596	C	2.636784	-0.243405	-0.374289
C	2.56088	1.194839	0.020456	C	2.352018	1.278852	-0.296159	C	-2.47207	1.181416	-0.186635	C	2.352163	1.271344	-0.292872
H	0.819062	-0.274234	-1.215805	H	0.642466	-0.489271	-1.050713	H	-0.997948	-0.320112	1.291128	H	0.637446	-0.492397	-1.047399
H	1.210894	0.709745	1.647524	H	1.344145	0.899927	1.587831	H	-0.838093	0.787734	-1.547168	H	1.345775	0.89219	1.591862
C	-1.280495	1.607933	1.111605	C	-0.934448	1.848958	-0.887732	C	1.375087	1.963084	-0.819816	C	-0.930496	1.853138	-0.881653
C	0.020989	1.912926	0.377994	C	-0.131654	1.855875	0.402102	C	0.098048	1.906132	0.003115	C	-0.129114	1.853797	0.409114
C	-2.46943	1.250978	0.205864	C	-2.289452	1.110971	-0.829931	C	2.013976	0.638857	-1.283568	C	-2.285441	1.114589	-0.829471
C	-2.278589	-0.002935	-0.646544	C	-2.256105	-0.420756	-0.809232	C	2.742164	-0.198906	-0.220191	C	-2.252617	-0.417295	-0.813176
C	-2.054555	-1.292911	0.145362	C	-1.862697	-1.101233	0.506952	C	1.8878	-0.798483	0.888111	C	-1.867889	-1.101689	0.503579
C	-0.857365	-2.146389	-0.217929	C	-0.781436	-2.166167	0.450154	C	0.957907	-1.956571	0.580997	C	-0.78754	-2.167753	0.451324
C	0.515404	-1.727896	0.362448	C	0.655942	-1.685277	0.753842	C	-0.315076	-1.669073	-0.255237	C	0.648909	-1.686394	0.759474
C	1.15848	-0.452545	-0.188238	C	1.124323	-0.48579	-0.068756	C	-1.121807	-0.460401	0.212828	C	1.120313	-0.490066	-0.066146
C	1.14036	0.925812	0.574756	C	1.068432	0.955546	0.530679	C	-0.96409	0.946639	-0.471008	C	1.068873	0.950179	0.535111
C	0.148111	3.022097	-0.35518	C	-0.457145	2.687026	1.397501	C	-0.077166	2.72517	1.043057	C	-0.455346	2.679955	1.408372
C	3.456763	-1.040291	0.907676	C	3.613521	-0.740783	0.684905	C	-3.160001	-1.022261	-1.325012	C	3.615108	-0.746231	0.681652
O	3.360989	-0.743641	-1.420862	O	2.977469	-0.77221	-1.654362	O	-3.501333	-0.680249	1.050472	O	3.069727	-0.633233	-1.6782
C	-2.93556	-1.689764	1.06632	C	-2.530038	-0.84401	1.633446	C	2.035297	-0.354749	2.138373	C	-2.542211	-0.846819	1.626452
O	-3.465673	-0.093454	-1.460908	O	-3.593218	-0.82033	-1.186855	O	3.518654	-1.228451	-0.867689	O	-3.587622	-0.815335	-1.200014
H	2.531796	1.72167	-0.936783	H	2.108207	1.662401	-1.289438	H	-2.64168	1.652572	0.782552	H	2.107831	1.655992	-1.285681
H	3.280705	1.687401	0.677787	H	3.100605	1.921168	0.173333	H	-3.052745	1.701508	-0.951501	H	3.104381	1.911547	0.172936
H	-1.564294	2.483062	1.704357	H	-0.337323	1.440126	-1.70905	H	2.122681	2.560158	-0.288205	H	-0.332169	1.448559	-1.704213
H	-1.117464	0.795963	1.825702	H	-1.152038	2.885172	-1.161561	H	1.135986	2.52201	-1.734698	H	-1.148099	2.890657	-1.150498
H	-3.36401	1.118735	0.822551	H	-2.870737	1.475834	0.021585	H	1.274009	0.019943	-1.798866	H	-2.868979	1.477018	0.021543
H	-2.674963	2.081537	-0.476408	H	-2.850144	1.377342	-1.731734	H	2.772781	0.88543	-2.033044	H	-2.843711	1.383694	-1.731969
H	-1.417303	0.152605	-1.306854	H	-1.564015	-0.757887	-1.591785	H	3.489003	0.443094	0.254879	H	-1.555998	-0.752476	-1.592426
H	-1.051769	-3.169383	0.117156	H	-1.025736	-2.96163	1.160318	H	1.53013	-2.743478	0.076278	H	-1.034974	-2.962443	1.161279
H	-0.767885	-2.188995	-1.31131	H	-0.789153	-2.624209	-0.545779	H	0.639364	-2.379965	1.537861	H	-0.792195	-2.626542	-0.544346
H	1.197319	-2.558038	0.144407	H	1.326101	-2.533496	0.572982	H	-0.939609	-2.567626	-0.185371	H	1.318933	-2.537124	0.586772
H	0.443118	-1.673801	1.454174	H	0.736653	-1.451242	1.821046	H	-0.068364	-1.567948	-1.31626	H	0.724746	-1.448828	1.826216
H	1.065032	3.272774	-0.875139	H	0.09078	2.69413	2.334365	H	-1.003819	2.757999	1.60471	H	0.091863	2.682307	2.345672
H	-0.672257	3.726113	-0.455659	H	-1.283666	3.385868	1.310508	H	0.711958	3.393412	1.372075	H	-1.281959	3.379085	1.324407
H	3.420505	-2.123573	0.771178	H	3.645501	-1.832757	0.704328	H	-3.132592	-2.112314	-1.226547	H	3.666418	-1.839756	0.680913
H	4.505138	-0.730675	0.902933	H	4.619747	-0.373573	0.456252	H	-4.194349	-0.723108	-1.513989	H	4.614225	-0.35776	0.467601

H	3.032457	-0.799902	1.884331	H	3.35128	-0.383732	1.683744	H	-2.559051	-0.75167	-2.196687	H	3.32943	-0.42727	1.68699
H	2.938488	-0.286248	-2.156366	H	3.919557	-0.621846	-1.797197	H	-3.582836	-1.640843	1.083944	H	3.240766	-1.582533	-1.665809
H	-2.818116	-2.631167	1.593471	H	-2.314369	-1.377459	2.553837	H	1.460335	-0.767076	2.961339	H	-2.332995	-1.382955	2.546761
H	-3.809383	-1.09829	1.317611	H	-3.31665	-0.098706	1.6771	H	2.729353	0.443693	2.381157	H	-3.328437	-0.100923	1.666991
H	-3.418883	-0.918779	-1.955826	H	-3.671532	-1.766654	-1.022872	H	2.960175	-1.659389	-1.524347	H	-3.668108	-1.761593	-1.036726

**9f_12**				**9f_13**				**9f_14**				**9f_15**			
C	-2.651269	-0.348816	-0.051291	C	2.709211	-0.272901	-0.185486	C	2.61386	-0.218625	-0.369888	C	2.607824	-0.223877	-0.368301
C	-2.472888	1.189393	-0.184787	C	2.493462	1.230762	0.111346	C	2.296749	1.2894	-0.30146	C	2.296501	1.296439	-0.304682
H	-1.00179	-0.312965	1.293722	H	0.851287	-0.248316	-1.271079	H	0.629003	-0.524963	-1.060889	H	0.632289	-0.522316	-1.06319
H	-0.840016	0.793208	-1.544994	H	1.066777	0.653793	1.642362	H	1.273035	0.904669	1.572599	H	1.271588	0.912271	1.568868
C	1.376338	1.962671	-0.82001	C	-1.379675	1.648535	1.081425	C	-0.992295	1.839808	-0.92404	C	-0.995765	1.836469	-0.929587
C	0.100345	1.908747	0.004825	C	-0.062455	1.903747	0.355186	C	-0.191268	1.852618	0.366853	C	-0.19354	1.854972	0.360421
C	2.009163	0.636639	-1.286987	C	-2.541174	1.218779	0.164368	C	-2.324542	1.053588	-0.883987	C	-2.327647	1.049974	-0.884382
C	2.73855	-0.204241	-0.226891	C	-2.249612	-0.044841	-0.644694	C	-2.199751	-0.471966	-0.802418	C	-2.201269	-0.475165	-0.798837
C	1.887292	-0.797356	0.887275	C	-1.975799	-1.298351	0.181259	C	-1.81359	-1.067151	0.551991	C	-1.808526	-1.066225	0.555431
C	0.951912	-1.953219	0.588118	C	-0.839516	-2.17722	-0.285635	C	-0.787742	-2.181435	0.530728	C	-0.781739	-2.179613	0.532072
C	-0.32096	-1.66661	-0.247849	C	0.559497	-1.77519	0.239392	C	0.667186	-1.704144	0.760593	C	0.673854	-1.702307	0.756629
C	-1.12472	-0.454281	0.215341	C	1.153298	-0.447087	-0.235489	C	1.100368	-0.498409	-0.074926	C	1.103519	-0.494091	-0.07702
C	-0.965231	0.952752	-0.468853	C	1.050956	0.906933	0.576083	C	1.008919	0.950082	0.512063	C	1.00852	0.955521	0.508018
C	-0.071654	2.727372	1.045535	C	0.082778	2.996388	-0.400079	C	-0.511718	2.700029	1.349948	C	-0.513561	2.706752	1.339938
C	-3.152442	-1.019654	-1.325953	C	3.418904	-1.013294	0.939299	C	3.594377	-0.692445	0.697656	C	3.592208	-0.688575	0.699642
O	-3.396377	-0.80856	1.07713	O	3.416113	-0.613299	-1.375622	O	3.066911	-0.607217	-1.66737	O	2.975988	-0.748275	-1.644132
C	2.042841	-0.350731	2.135542	C	-2.735416	-1.630483	1.227944	C	-2.407788	-0.677853	1.681654	C	-2.397992	-0.674295	1.68668
O	3.505097	-1.238941	-0.877921	O	-3.29993	-0.305802	-1.598277	O	-3.416838	-1.10535	-1.257393	O	-3.419879	-1.110744	-1.2466
H	-2.642727	1.659932	0.784637	H	2.503336	1.798528	-0.822102	H	2.057604	1.663584	-1.299546	H	2.057627	1.669898	-1.303032
H	-3.0502	1.710582	-0.951931	H	3.162296	1.712089	0.828311	H	3.029747	1.947549	0.170079	H	3.026308	1.956171	0.170323
H	2.126996	2.556308	-0.288856	H	-1.680458	2.563641	1.600298	H	-0.381328	1.455057	-1.747578	H	-0.385698	1.4483	-1.752192
H	1.137536	2.523881	-1.733582	H	-1.232153	0.889448	1.853884	H	-1.239018	2.872092	-1.187232	H	-1.242993	2.867545	-1.197007
H	1.265083	0.020415	-1.799598	H	-3.444916	1.0764	0.767399	H	-2.950011	1.432814	-0.069779	H	-2.95148	1.431014	-0.069757
H	2.765823	0.880822	-2.03939	H	-2.761319	2.018427	-0.549267	H	-2.860975	1.260277	-1.815382	H	-2.866374	1.253644	-1.815113
H	3.492055	0.43393	0.242816	H	-1.380665	0.153965	-1.274908	H	-1.463044	-0.78419	-1.545974	H	-1.467521	-0.7889	-1.544809
H	1.520534	-2.744972	0.086914	H	-1.025729	-3.206208	0.035773	H	-1.028408	-2.922874	1.298133	H	-1.01938	-2.920333	1.301099
H	0.633356	-2.370086	1.547838	H	-0.818417	-2.187057	-1.382034	H	-0.84568	-2.696641	-0.434153	H	-0.842707	-2.695974	-0.431966
H	-0.949927	-2.560893	-0.171884	H	1.246523	-2.56633	-0.083996	H	1.328755	-2.551868	0.545592	H	1.335559	-2.547206	0.534013
H	-0.075494	-1.570453	-1.309693	H	0.549126	-1.808062	1.334231	H	0.80013	-1.473402	1.823204	H	0.811841	-1.47404	1.819178
H	-0.997985	2.762143	1.607617	H	1.001681	3.227562	-0.924854	H	0.032488	2.71476	2.288923	H	0.031168	2.725825	2.27854
H	0.719497	3.393292	1.37441	H	-0.728788	3.708501	-0.514301	H	-1.332123	3.404395	1.250529	H	-1.333933	3.410717	1.237625
H	-3.091527	-2.107428	-1.241102	H	3.413733	-2.091013	0.760822	H	3.670102	-1.784488	0.705722	H	3.647447	-1.779416	0.727265
H	-4.199222	-0.748844	-1.500979	H	4.458879	-0.679766	0.986042	H	4.586151	-0.283256	0.488145	H	4.591892	-0.301503	0.475076
H	-2.581142	-0.707931	-2.203779	H	2.953207	-0.820164	1.907554	H	3.294111	-0.372328	1.698322	H	3.315582	-0.329997	1.694027
H	-4.334982	-0.745025	0.863578	H	3.008764	-0.139379	-2.109173	H	3.256256	-1.552923	-1.648138	H	3.914507	-0.573124	-1.782264
H	1.470278	-0.758321	2.962528	H	-2.579382	-2.561321	1.763953	H	-2.194985	-1.166803	2.627085	H	-2.180473	-1.160361	2.632526
H	2.741286	0.445612	2.372581	H	-3.537528	-0.996607	1.591196	H	-3.119034	0.14026	1.713666	H	-3.10982	0.143284	1.719462
H	2.939152	-1.669203	-1.528614	H	-4.062468	-0.619976	-1.098438	H	-4.059949	-1.022162	-0.543822	H	-4.058627	-1.029205	-0.528947

**9f_16**				**9f_17**				**9f_18**				**9g_1**			
C	-2.658011	-0.359403	-0.055654	C	-2.693153	-0.020386	-0.17266	C	-2.687256	-0.013358	-0.175389	C	2.732037	-0.330434	-0.221168
C	-2.481073	1.178704	-0.17939	C	-2.186026	-1.44711	0.128494	C	-2.186398	-1.455193	0.120067	C	2.462826	1.046077	0.418729
H	-0.987331	-0.313864	1.293776	H	-0.924471	0.262191	-1.297108	H	-0.924426	0.260807	-1.294359	H	0.948449	-0.859177	-1.438361
H	-0.852669	0.7863	-1.545266	H	-0.927738	-0.561448	1.649575	H	-0.932499	-0.575729	1.648307	H	1.20086	1.492407	-1.271507
C	1.363411	1.9614	-0.829051	C	1.133083	-1.740129	-0.908956	C	1.135363	-1.739391	-0.911749	C	-1.281638	2.027248	-0.54695
C	0.089954	1.90649	-0.000482	C	0.444431	-1.620744	0.437196	C	0.445219	-1.624387	0.434	C	-0.10655	1.748263	0.371922
C	2.001528	0.635922	-1.290353	C	1.690461	-0.464606	-1.585329	C	1.690624	-0.461409	-1.585385	C	-1.875199	0.825591	-1.304576
C	2.737177	-0.195417	-0.227019	C	2.54316	0.496578	-0.742076	C	2.54099	0.499962	-0.739988	C	-2.772431	-0.089082	-0.470469
C	1.890141	-0.791466	0.888823	C	1.789377	1.330436	0.289635	C	1.785676	1.330159	0.293614	C	-2.092371	-0.879236	0.631277
C	0.96073	-1.9527	0.592073	C	0.677135	2.222106	-0.245617	C	0.671313	2.220406	-0.239638	C	-0.973163	-1.832173	0.250535
C	-0.314645	-1.672531	-0.242502	C	-0.754651	1.825354	0.182861	C	-0.75972	1.820897	0.18789	C	0.475864	-1.399133	0.59153
C	-1.123344	-0.462144	0.215665	C	-1.196576	0.432394	-0.25303	C	-1.198619	0.427701	-0.250223	C	1.182088	-0.502999	-0.430185
C	-0.973852	0.945798	-0.468959	C	-0.830564	-0.827033	0.593001	C	-0.832581	-0.835674	0.590602	C	1.076477	1.072999	-0.268518
C	-0.080397	2.727861	1.038391	C	0.922877	-2.295391	1.488092	C	0.924722	-2.300038	1.483867	C	-0.105948	2.177884	1.636431
C	-3.14333	-1.021282	-1.337106	C	-3.515052	0.599053	0.952528	C	-3.512844	0.592528	0.95453	C	3.525257	-1.357758	0.570033
O	-3.515413	-0.842047	0.975646	O	-3.416827	0.037832	-1.403409	O	-3.337096	0.194346	-1.430171	O	3.37255	-0.084552	-1.485486
C	2.043786	-0.341811	2.136263	C	2.146873	1.360342	1.573385	C	2.143937	1.358742	1.577199	C	-2.549351	-0.800112	1.882858
O	3.512154	-1.226598	-0.873513	O	3.620966	-0.260649	-0.193714	O	3.62058	-0.256087	-0.193473	O	-3.372829	-0.998707	-1.417579
H	-2.64391	1.656304	0.789654	H	-2.077377	-2.005732	-0.803956	H	-2.075449	-2.009006	-0.814924	H	3.16902	1.840185	0.167806
H	-3.065188	1.702682	-0.939205	H	-2.739864	-2.047404	0.853588	H	-2.738777	-2.059132	0.843745	H	2.389526	0.956683	1.50402

H	2.112569	2.561406	-0.303049
H	1.119509	2.516528	-1.744955
H	1.259593	0.013535	-1.798529
H	2.755681	0.880138	-2.045242
H	3.485318	0.450371	0.240802
H	1.532363	-2.742169	0.090775
H	0.645713	-2.370498	1.552587
H	-0.941861	-2.567855	-0.161703
H	-0.071784	-1.579592	-1.305014
H	-1.00474	2.76411	1.603686
H	0.710754	3.396052	1.362482
H	-3.084355	-2.109336	-1.254695
H	-4.186988	-0.747509	-1.512993
H	-2.556472	-0.707212	-2.202373
H	-3.235612	-0.440792	1.805884
H	1.474762	-0.751963	2.964457
H	2.737571	0.45909	2.371586
H	2.950689	-1.663099	-1.523922

H	0.436302	-2.192659	-1.627064
H	1.954136	-2.450977	-0.794331
H	2.323539	-0.799275	-2.413099
H	0.88863	0.112528	-2.044359
H	2.949012	1.229895	-1.459433
H	0.857032	3.246906	0.094073
H	0.722499	2.255713	-1.339115
H	-1.441897	2.566291	-0.244712
H	-0.838884	1.917428	1.27107
H	0.443374	-2.24713	2.461078
H	1.811688	-2.913119	1.41146
H	-3.706594	1.659458	0.757999
H	-4.477403	0.085786	1.027092
H	-3.009647	0.523662	1.918269
H	-3.716489	0.945714	-1.531894
H	1.649809	2.014524	2.282568
H	2.926319	0.717959	1.964278
H	4.236659	0.35704	0.215854

H	0.440372	-2.192246	-1.631366
H	1.958046	-2.448438	-0.797688
H	2.325099	-0.793478	-2.41314
H	0.888308	0.114932	-2.044699
H	2.945143	1.235749	-1.455785
H	0.849236	3.244822	0.102163
H	0.716661	2.256487	-1.333071
H	-1.449106	2.558637	-0.240402
H	-0.844924	1.910931	1.276232
H	0.444326	-2.255189	2.456577
H	1.815046	-2.91545	1.406434
H	-3.677489	1.659064	0.78248
H	-4.489607	0.099536	1.007171
H	-3.029494	0.468772	1.926584
H	-4.256862	-0.083065	-1.342983
H	1.645848	2.010704	2.287708
H	2.925083	0.717486	1.966556
H	4.235027	0.362074	0.217226

H	-0.935455	2.743862	-1.303046
H	-2.074568	2.532225	0.014107
H	-2.500383	1.203537	-2.119258
H	-1.085655	0.233932	-1.775122
H	-3.558775	0.525931	-0.012933
H	-1.155042	-2.775063	0.774817
H	-1.042035	-2.062585	-0.816645
H	1.060108	-2.322187	0.664173
H	0.492908	-0.948065	1.589068
H	0.746409	2.050188	2.293213
H	-0.971255	2.677994	2.059034
H	3.553901	-2.320626	0.048349
H	4.553892	-1.005107	0.685931
H	3.104737	-1.523789	1.564
H	3.344986	-0.906965	-1.988845
H	-3.359644	-0.129009	2.149274
H	-2.119647	-1.389807	2.687057
H	-3.947482	-1.591293	-0.919305

9g_2

C	2.72273	-0.340471	-0.227249
C	2.461491	1.038604	0.42873
H	0.95431	-0.85247	-1.443472
H	1.201934	1.49733	-1.260768
C	-1.279667	2.031382	-0.537321
C	-0.106399	1.747262	0.382342
C	-1.872697	0.833193	-1.300862
C	-2.770971	-0.084863	-0.471579
C	-2.09184	-0.881807	0.625881
C	-0.973607	-1.834057	0.240313
C	0.475535	-1.404443	0.583863
C	1.179912	-0.502017	-0.432783
C	1.076219	1.073041	-0.259996
C	-0.107359	2.17117	1.64876
C	3.521504	-1.370013	0.554884
O	3.276833	-0.190192	-1.546461
C	-2.548769	-0.808889	1.877848
O	-3.372715	-0.988629	-1.423372
H	3.165117	1.840477	0.191337
H	2.38366	0.941539	1.513718
H	-0.931534	2.750756	-1.28992
H	-2.073196	2.534724	0.02438
H	-2.497007	1.214859	-2.114481
H	-1.082764	0.243486	-1.7732
H	-3.55646	0.528689	-0.010579
H	-1.156545	-2.779246	0.76004
H	-1.042462	-2.059083	-0.827979
H	1.059848	-2.32781	0.649723
H	0.492794	-0.959919	1.584403
H	0.743744	2.039217	2.306347
H	-0.97266	2.670789	2.072017
H	3.533646	-2.330145	0.031492
H	4.557203	-1.028097	0.658581
H	3.120942	-1.523598	1.559155
H	4.175885	0.145466	-1.447735
H	-3.358263	-0.138219	2.147843
H	-2.119826	-1.403407	2.678905
H	-3.947798	-1.583325	-0.928124

9g_3

C	-2.805428	-0.280794	0.045154
C	-2.492928	1.202697	-0.23631
H	-1.091089	-1.124006	1.186052
H	-1.307251	1.215522	1.569558
C	1.284741	1.599404	1.018186
C	0.089832	1.782065	0.088896
C	2.605035	1.180046	0.355258
C	2.542629	-0.103294	-0.480885
C	1.880449	-1.287458	0.223832
C	0.7498	-1.981478	-0.503531
C	-0.487212	-1.138531	-0.894076
C	-1.269168	-0.535216	0.281529
C	-1.146197	1.035716	0.502265
C	0.140094	2.586457	-0.976331
C	-3.571939	-1.072376	-1.001735
O	-3.50337	-0.327859	1.301705
C	2.343085	-1.737145	1.392009
O	3.911242	-0.390225	-0.83273
H	-3.206404	1.928741	0.158634
H	-2.361123	1.36952	-1.307414
H	1.027448	0.859738	1.779708
H	1.456857	2.538086	1.556981
H	2.981691	1.977679	-0.292197
H	3.356837	1.032578	1.13765
H	1.983857	0.106997	-1.399314
H	1.156268	-2.399552	-1.435279
H	0.416709	-2.833888	0.097024
H	-1.140545	-1.815971	-1.450699
H	-0.200126	-0.35412	-1.601356
H	-0.715775	2.742335	-1.622376
H	1.043872	3.129064	-1.235207
H	-3.609025	-2.136197	-0.742577
H	-4.598473	-0.698846	-1.049827
H	-3.127583	-0.980184	-1.99485
H	-3.50987	-1.246652	1.595512
H	3.186085	-1.2705	1.890805
H	1.90364	-2.59985	1.882102
H	3.914271	-1.196382	-1.360293

9g_4

C	-2.796288	-0.293723	0.048599
C	-2.491839	1.197082	-0.245744
H	-1.095786	-1.122052	1.188253
H	-1.309486	1.218652	1.562665
C	1.283395	1.60371	1.013617
C	0.089064	1.784321	0.083049
C	2.603939	1.181958	0.35263
C	2.541746	-0.103761	-0.479842
C	1.879451	-1.286027	0.227907
C	0.750243	-1.983324	-0.498659
C	-0.486937	-1.142505	-0.892295
C	-1.26666	-0.53563	0.282264
C	-1.146251	1.036432	0.496123
C	0.139517	2.588069	-0.982613
C	-3.567753	-1.087952	-0.992169
O	-3.410734	-0.451671	1.339827
C	2.340694	-1.731422	1.398265
O	3.910533	-0.391608	-0.830483
H	-3.202745	1.932942	0.13844
H	-2.355278	1.357994	-1.317834
H	1.025347	0.866168	1.776929
H	1.455728	2.543735	1.549979
H	2.981217	1.977624	-0.296888
H	3.355195	1.036543	1.135931
H	1.98331	0.103928	-1.399087
H	1.157931	-2.403477	-1.428933
H	0.417273	-2.834239	0.103975
H	-1.140821	-1.821835	-1.445802
H	-0.200338	-0.360675	-1.602633
H	-0.715707	2.741967	-1.629984
H	1.042834	3.13199	-1.240454
H	-3.592865	-2.149605	-0.730435
H	-4.600491	-0.725333	-1.036819
H	-3.13838	-0.982685	-1.99068
H	-4.296696	-0.073476	1.288441
H	3.182518	-1.262363	1.896783
H	1.900986	-2.592628	1.890722
H	3.913934	-1.20033	-1.354098

9g_5

C	2.714024	-0.340827	-0.227727
C	2.457196	1.063704	0.3736
H	0.933265	-0.904906	-1.40031
H	1.169549	1.450375	-1.313259
C	-1.302141	2.008276	-0.569505
C	-0.11351	1.759373	0.340654
C	-1.885986	0.791526	-1.313916
C	-2.777736	-0.132914	-0.476853
C	-2.093073	-0.867129	0.662757
C	-0.968048	-1.834773	0.336207
C	0.479637	-1.38012	0.652319
C	1.169098	-0.515091	-0.406349
C	1.06158	1.065605	-0.29467
C	-0.09544	2.23	1.59026
C	3.524587	-1.335263	0.587134
O	3.250355	-0.245044	-1.559088
C	-2.538725	-0.720574	1.911331
O	-3.443665	-1.078334	-1.341969
H	3.154097	1.857217	0.092488
H	2.396332	1.011709	1.462649
H	-0.974356	2.722846	-1.33571
H	-2.098501	2.505544	-0.00667
H	-2.510984	1.156351	-2.134723
H	-1.083289	0.208029	-1.777304
H	-3.590369	0.463263	-0.053031
H	-1.148341	-2.748883	0.909062
H	-1.01771	-2.134466	-0.716245
H	1.070499	-2.296103	0.752767
H	0.498343	-0.894908	1.633444
H	0.767404	2.12633	2.237324
H	-0.955988	2.740954	2.009609
H	3.532959	-2.316159	0.10375
H	4.560594	-0.987668	0.664956
H	3.135785	-1.447935	1.601415
H	4.147372	0.102805	-1.487518
H	-3.349744	-0.039692	2.148666
H	-2.099278	-1.263324	2.743009
H	-2.779948	-1.475107	-1.917368

9g_6

C	2.723581	-0.330123	-0.222122
C	2.457809	1.069892	0.365979
H	0.928295	-0.910184	-1.396149
H	1.169733	1.446283	-1.321896
C	-1.303462	2.004406	-0.579187
C	-0.114109	1.759669	0.331057
C	-1.888387	0.784282	-1.317248

9g_7

C	-2.785034	-0.249854	0.050179
C	-2.44231	1.225237	-0.23696
H	-1.079773	-1.125031	1.17831
H	-1.243157	1.211066	1.557075
C	1.34269	1.639061	1.00777
C	0.145524	1.778847	0.070938
C	2.648934	1.146278	0.356911

9g_8

C	-2.775662	-0.262335	0.053558
C	-2.440325	1.219886	-0.247019
H	-1.08409	-1.122988	1.180108
H	-1.244093	1.214339	1.549314
C	1.342419	1.644519	1.002233
C	0.145839	1.781181	0.064039
C	2.648621	1.147622	0.354187

9g_9

C	-2.811344	-0.290248	0.0475
C	-2.504237	1.209225	-0.199587
H	-1.104851	-1.13362	1.189035
H	-1.269787	1.217771	1.587382
C	1.290836	1.607748	1.004394
C	0.0878	1.778209	0.083217
C	2.607868	1.186818	0.33625

C	-2.779184	-0.136069	-0.474838	C	2.499035	-0.133144	-0.475473	C	2.497596	-0.134403	-0.473911	C	2.545144	-0.105581	-0.486061
C	-2.093479	-0.865203	0.667367	C	1.818281	-1.29006	0.251454	C	1.815771	-1.288364	0.256456	C	1.883757	-1.282294	0.231848
C	-0.967549	-1.832849	0.344243	C	0.737221	-2.016692	-0.510592	C	0.737526	-2.019289	-0.505453	C	0.752654	-1.983735	-0.487391
C	0.480038	-1.375215	0.658146	C	-0.500511	-1.174596	-0.906036	C	-0.500374	-1.179512	-0.904553	C	-0.485598	-1.145663	-0.884079
C	1.171533	-0.515783	-0.404465	C	-1.253127	-0.53872	0.271367	C	-1.250168	-0.539263	0.271603	C	-1.273401	-0.539767	0.286069
C	1.061891	1.065313	-0.301847	C	-1.091599	1.032051	0.488375	C	-1.090512	1.032738	0.481351	C	-1.144009	1.03175	0.513856
C	-0.095651	2.234902	1.578953	C	0.194304	2.565251	-1.008149	C	0.194665	2.566495	-1.015805	C	0.127873	2.571224	-0.990568
C	3.529785	-1.322954	0.599251	C	-3.578462	-1.024199	-0.989621	C	-3.574663	-1.03957	-0.979317	C	-3.556373	-1.048357	-1.033543
O	3.346999	-0.133072	-1.50311	O	-3.472804	-0.280394	1.312884	O	-3.381491	-0.404777	1.350731	O	-3.564598	-0.508185	1.251193
C	-2.539228	-0.71464	1.915434	C	2.202319	-1.671185	1.471903	C	2.196012	-1.662789	1.480145	C	2.34737	-1.719536	1.40433
O	-3.446128	-1.085712	-1.33477	O	3.770079	-0.544603	-1.016156	O	3.768323	-0.549162	-1.013027	O	3.913626	-0.39546	-0.835438
H	3.157291	1.855788	0.07395	H	-3.13497	1.968601	0.163065	H	-3.12976	1.973438	0.142197	H	-3.210974	1.928074	0.221629
H	2.400421	1.023427	1.454825	H	-2.318554	1.38591	-1.309891	H	-2.312214	1.374023	-1.32107	H	-2.387246	1.40717	-1.26744
H	-0.976272	2.71525	-1.349085	H	1.077058	0.958545	1.819375	H	1.075668	0.967807	1.816628	H	1.043004	0.874391	1.775053
H	-2.099193	2.504484	-0.017966	H	1.538335	2.60937	1.477375	H	1.538861	2.616698	1.467591	H	1.463338	2.552155	1.532967
H	-2.514333	1.14544	-2.138966	H	3.064335	1.915464	-0.301051	H	3.065486	1.913995	-0.306147	H	2.977718	1.979327	-0.321296
H	-1.086275	0.19862	-1.778915	H	3.391055	0.980118	1.146403	H	3.389869	0.983291	1.144871	H	3.364952	1.049804	1.11543
H	-3.591363	0.462124	-0.053002	H	1.911895	0.110395	-1.361596	H	1.910883	0.106918	-1.360947	H	1.985806	0.094267	-1.406504
H	-1.146711	-2.745033	0.920558	H	1.174877	-2.41333	-1.436376	H	1.177226	-2.417713	-1.429467	H	1.157988	-2.409564	-1.416101
H	-1.017215	-2.136658	-0.707085	H	0.402987	-2.878631	0.075258	H	0.403365	-2.879869	0.082327	H	0.420647	-2.830982	0.120928
H	1.070831	-2.290704	0.764725	H	-1.169603	-1.857387	-1.436699	H	-1.17026	-1.864257	-1.431528	H	-1.137001	-1.827127	-1.43774
H	0.498243	-0.884273	1.636356	H	-0.217959	-0.408737	-1.635451	H	-0.218212	-0.416795	-1.637415	H	-0.200097	-0.363495	-1.594429
H	0.767737	2.134616	2.225833	H	-0.655954	2.698177	-1.666373	H	-0.654816	2.696796	-1.675559	H	-0.733025	2.721394	-1.630985
H	-0.956454	2.74635	1.997121	H	1.093235	3.116546	-1.266226	H	1.092939	3.119371	-1.272912	H	1.030063	3.109737	-1.26291
H	3.557187	-2.304789	0.114107	H	-3.640932	-2.086275	-0.728277	H	-3.624017	-2.099837	-0.715452	H	-3.585432	-2.118478	-0.808968
H	4.557997	-0.961948	0.69041	H	-4.595535	-0.624909	-1.031819	H	-4.598487	-0.6517	-1.016947	H	-4.585635	-0.682086	-1.081557
H	3.120754	-1.452775	1.603369	H	-3.138156	-0.945059	-1.98569	H	-3.150069	-0.946674	-1.981115	H	-3.098338	-0.909138	-2.013697
H	3.324156	-0.977271	-1.969196	H	-3.497303	-1.198607	1.6075	H	-4.259676	-0.007986	1.306617	H	-3.149559	-0.017651	1.969007
H	-3.350892	-0.033717	2.150408	H	2.980881	-1.153566	2.023736	H	2.971716	-1.140959	2.032039	H	3.190648	-1.247314	1.897356
H	-2.099187	-1.253984	2.749015	H	1.75248	-2.526984	1.964486	H	1.745282	-2.516403	1.975663	H	1.908527	-2.577094	1.903865
H	-2.783249	-1.484541	-1.909695	H	4.302956	-0.865357	-0.279354	H	4.299267	-0.871474	-0.275504	H	3.916983	-1.207139	-1.35447

**9g_10**				**9h_1**				**9h_2**				**9h_3**			
C	2.583387	-0.496987	-0.387018	C	-2.721097	-0.121841	0.035757	C	-2.714014	-0.127667	0.036331	C	-2.744631	-0.263391	0.011991
C	2.54996	1.020829	-0.0976	C	-2.351589	1.376053	0.079484	C	-2.352129	1.382895	0.085794	C	-2.497938	1.259258	0.060355
H	0.509832	-1.116605	-0.932568	H	-0.976154	-0.474601	1.218965	H	-0.977154	-0.471204	1.217083	H	-0.955415	-0.476604	1.158882
H	0.931371	1.153812	-1.513893	H	-0.884481	0.98316	-1.459726	H	-0.886934	0.99539	-1.456449	H	-1.043526	0.979737	-1.515993
C	-1.278682	2.122095	-0.455149	C	1.517631	1.76299	-0.777145	C	1.51835	1.765426	-0.772841	C	1.337122	1.91265	-0.881136
C	0.056358	1.936237	0.24817	C	0.297481	1.763044	0.129854	C	0.298913	1.765515	0.135131	C	0.105383	1.883478	0.019709
C	-2.00283	0.896824	-1.051094	C	1.931049	0.44243	-1.455196	C	1.928033	0.445808	-1.45502	C	2.586773	1.126451	-0.432583
C	-2.746442	-0.052527	-0.08265	C	2.616823	-0.617827	-0.577536	C	2.612865	-0.618335	-0.581326	C	2.531503	-0.401311	-0.542572
C	-1.90403	-1.18831	0.482351	C	1.770238	-1.225565	0.533662	C	1.766983	-1.226944	0.529957	C	1.765279	-1.130354	0.560638
C	-0.911137	-0.804869	1.56346	C	0.693965	-2.219143	0.140688	C	0.687999	-2.217549	0.136709	C	0.742658	-2.162077	0.138288
C	0.583009	-1.008836	1.218366	C	-0.586813	-1.670339	-0.540401	C	-0.592019	-1.665856	-0.542757	C	-0.486628	-1.616534	-0.623039
C	1.029414	-0.536249	-0.16638	C	-1.196247	-0.451189	0.147225	C	-1.19793	-0.445506	0.145513	C	-1.19533	-0.465622	0.091203
C	1.037213	1.022819	-0.433384	C	-0.910473	1.00769	-0.364949	C	-0.911742	1.015412	-0.361547	C	-1.043911	1.011104	-0.421008
C	0.345662	2.630998	1.352323	C	0.297838	2.463566	1.266858	C	0.301688	2.46197	1.274572	C	0.050634	2.652538	1.110568
C	3.503653	-1.384376	0.435722	C	-3.394821	-0.558049	-1.261253	C	-3.392038	-0.553791	-1.261746	C	-3.406646	-0.744832	-1.274924
O	2.886713	-0.642016	-1.785651	O	-3.517315	-0.502804	1.159273	O	-3.42388	-0.649739	1.160595	O	-3.483802	-0.715151	1.148009
C	-2.059153	-2.447081	0.067789	C	2.027476	-0.967906	1.817519	C	2.027267	-0.972925	1.813946	C	2.065765	-0.933941	1.846011
O	-3.873386	-0.5332	-0.825216	O	3.817098	-0.009467	-0.090521	O	3.815569	-0.01412	-0.09507	O	3.914076	-0.824752	-0.550632
H	3.205047	1.640172	-0.713285	H	-2.389354	1.727579	1.111499	H	-2.389271	1.730343	1.119197	H	-2.537902	1.596711	1.097162
H	2.749405	1.22077	0.956316	H	-2.915673	2.059184	-0.559229	H	-2.91429	2.068622	-0.552383	H	-3.131756	1.899862	-0.556745
H	-1.085539	2.793937	-1.302516	H	2.373971	2.173418	-0.23741	H	2.376067	2.172547	-0.232781	H	1.65025	2.955888	-0.983958
H	-1.965015	2.661381	0.205473	H	1.303675	2.467662	-1.592822	H	1.305126	2.472838	-1.586351	H	1.058066	1.581593	-1.887205
H	-2.785554	1.287941	-1.706553	H	2.651495	0.689615	-2.241253	H	2.647816	0.693564	-2.241508	H	2.862389	1.411863	0.586373
H	-1.331689	0.316284	-1.689458	H	1.080437	-0.013203	-1.964426	H	1.075668	-0.006637	-1.964158	H	3.413294	1.437438	-1.079796
H	-3.107212	0.545927	0.768703	H	2.884837	-1.44691	-1.251714	H	2.877584	-1.446322	-1.258141	H	2.086151	-0.661471	-1.512492
H	-1.06891	0.237709	1.839645	H	1.13145	-2.977418	-0.520932	H	1.123396	-2.976015	-0.526057	H	1.232747	-2.910861	-0.498247
H	-1.126243	-1.390637	2.463559	H	0.386566	-2.742849	1.050373	H	0.38018	-2.741559	1.046066	H	0.394808	-2.690936	1.030769
H	0.823863	-2.074556	1.296006	H	-1.315134	-2.489766	-0.533999	H	-1.324043	-2.481214	-0.533591	H	-1.17931	-2.455176	-0.75628
H	1.165505	-0.508317	1.999261	H	-0.400685	-1.453506	-1.596419	H	-0.406937	-1.448912	-1.598992	H	-0.199388	-1.306462	-1.633202
H	1.316685	2.580059	1.830931	H	-0.577888	2.542367	1.901072	H	-0.573805	2.540959	1.909071	H	-0.814434	2.674659	1.763351
H	-0.388123	3.283907	1.814366	H	1.188598	2.982341	1.606133	H	1.193988	2.977151	1.615291	H	0.883737	3.288863	1.393171
H	3.350793	-2.440853	0.19011	H	-3.498323	-1.647222	-1.302915	H	-3.464907	-1.642567	-1.320816	H	-3.424549	-1.83848	-1.322954
H	4.543919	-1.132413	0.211573	H	-4.393869	-0.118022	-1.319522	H	-4.405702	-0.140906	-1.304573	H	-4.438477	-0.38548	-1.310409
H	3.343575	-1.259834	1.508448	H	-2.828457	-0.243832	-2.141494	H	-2.852455	-0.194628	-2.14159	H	-2.884786	-0.381544	-2.163815

H	2.688502	-1.553182	-2.032423
H	-2.76998	-2.706767	-0.70742
H	-1.469732	-3.255883	0.487413
H	-4.355419	-1.144529	-0.256186

H	-3.716103	-1.44345	1.080505
H	1.455579	-1.437098	2.610763
H	2.804643	-0.273986	2.113599
H	4.287837	-0.668778	0.431534

H	-4.359081	-0.441376	1.048227
H	1.455925	-1.442818	2.607174
H	2.806421	-0.281267	2.110119
H	4.285545	-0.675815	0.424655

H	-3.59474	-1.670396	1.07232
H	1.568882	-1.490419	2.633727
H	2.82366	-0.223276	2.156867
H	3.927907	-1.780788	-0.431549

9h_4

C	-2.736956	-0.270027	0.015268
C	-2.500717	1.264909	0.075056
H	-0.951968	-0.475027	1.151254
H	-1.056981	0.998808	-1.512999
C	1.335371	1.910505	-0.881214
C	0.104989	1.886983	0.021982
C	2.585143	1.125589	-0.430681
C	2.533023	-0.402276	-0.542572
C	1.761297	-1.134061	0.554941
C	0.736185	-2.160144	0.124879
C	-0.489438	-1.606666	-0.636091
C	-1.196817	-0.459084	0.084714
C	-1.049902	1.021495	-0.417725
C	0.055986	2.655097	1.113685
C	-3.409051	-0.737095	-1.271485
O	-3.371909	-0.861443	1.149868
C	2.059302	-0.944398	1.841938
O	3.916401	-0.822867	-0.542577
H	-2.534876	1.593869	1.114805
H	-3.137033	1.909876	-0.53548
H	1.648999	2.953054	-0.989578
H	1.054539	1.575169	-1.885347
H	2.858024	1.409924	0.589319
H	3.412821	1.438424	-1.075536
H	2.093996	-0.662183	-1.515468
H	1.225278	-2.906894	-0.514745
H	0.384159	-2.692536	1.013578
H	-1.184775	-2.441195	-0.776208
H	-0.198519	-1.289271	-1.642951
H	-0.808428	2.681187	1.767166
H	0.892856	3.286675	1.395867
H	-3.399718	-1.827767	-1.339058
H	-4.452044	-0.403065	-1.288613
H	-2.918293	-0.330893	-2.159428
H	-4.323749	-0.730056	1.063948
H	1.558609	-1.502552	2.626039
H	2.819091	-0.237783	2.157488
H	3.931585	-1.778243	-0.418542

9h_5

C	-2.722774	-0.134282	0.031041
C	-2.359551	1.37433	0.089442
H	-0.966372	-0.472714	1.220255
H	-0.897474	0.987319	-1.454524
C	1.507327	1.763956	-0.778651
C	0.289979	1.76401	0.132203
C	1.918774	0.443683	-1.458321
C	2.61132	-0.614876	-0.583937
C	1.772482	-1.222291	0.533409
C	0.695741	-2.218618	0.148809
C	-0.587042	-1.674536	-0.531763
C	-1.198544	-0.45309	0.148685
C	-0.919349	1.008902	-0.359907
C	0.29425	2.464774	1.26903
C	-3.380256	-0.553988	-1.275516
O	-3.558212	-0.64877	1.065314
C	2.037203	-0.961672	1.815182
O	3.813655	-0.004334	-0.10489
H	-2.389923	1.727876	1.122768
H	-2.925633	2.063216	-0.541514
H	2.36522	2.174621	-0.241627
H	1.290383	2.468871	-1.593296
H	2.634476	0.691523	-2.248471
H	1.066147	-0.013558	-1.962689
H	2.8762	-1.444335	-1.258817
H	1.131813	-2.979915	-0.510218
H	0.39138	-2.738085	1.061968
H	-1.317229	-2.491734	-0.516453
H	-0.404385	-1.462884	-1.589291
H	-0.57941	2.545971	1.905814
H	1.18665	2.982562	1.605381
H	-3.460902	-1.642304	-1.332639
H	-4.387847	-0.13297	-1.326623
H	-2.817459	-0.203039	-2.142644
H	-3.173484	-0.393254	1.911095
H	1.471371	-1.430576	2.612964
H	2.815309	-0.266124	2.104846
H	4.290256	-0.663439	0.412084

9h_6

C	-2.72896	-0.221471	-0.033556
C	-2.425937	1.288283	-0.096719
H	-1.021427	-0.423785	1.244879
H	-0.864892	0.84199	-1.5273
C	1.411562	1.949846	-0.867133
C	0.175313	1.860465	0.021073
C	2.637498	1.081673	-0.501493
C	2.430238	-0.43668	-0.567532
C	1.730812	-1.068954	0.631971
C	0.743837	-2.171448	0.334835
C	-0.520714	-1.710072	-0.427984
C	-1.197348	-0.474562	0.166004
C	-0.947821	0.957356	-0.441625
C	0.084855	2.611267	1.122197
C	-3.328808	-0.785942	-1.317268
O	-3.556696	-0.547376	1.084614
C	2.033988	-0.725736	1.885716
O	3.685537	-1.11956	-0.788205
H	-2.5277	1.71998	0.899969
H	-2.986395	1.892113	-0.813969
H	1.754598	2.988564	-0.871991
H	1.125315	1.717294	-1.898919
H	3.019685	1.374918	0.480677
H	3.425051	1.313632	-1.225318
H	1.859953	-0.659191	-1.473626
H	1.241569	-2.950382	-0.256255
H	0.437631	-2.634623	1.277492
H	-1.223898	-2.550644	-0.422587
H	-0.282487	-1.530183	-1.481515
H	-0.783328	2.590937	1.771218
H	0.892894	3.272322	1.419902
H	-3.391971	-1.878102	-1.276451
H	-4.339381	-0.390923	-1.4505
H	-2.736479	-0.518989	-2.19606
H	-3.706913	-1.500231	1.078027
H	1.583877	-1.237509	2.729998
H	2.733913	0.067105	2.124183
H	4.168567	-1.092959	0.045968

9h_7

C	-2.721865	-0.225914	-0.0312
C	-2.426666	1.296543	-0.085867
H	-1.021913	-0.420788	1.2417
H	-0.871999	0.856216	-1.525267
C	1.412723	1.949388	-0.866443
C	0.177961	1.862639	0.024263
C	2.63771	1.079248	-0.502449
C	2.428119	-0.438723	-0.569593
C	1.726719	-1.070534	0.629002
C	0.737086	-2.170249	0.330486
C	-0.526204	-1.705592	-0.432021
C	-1.199457	-0.468923	0.162888
C	-0.950271	0.965832	-0.438608
C	0.092838	2.610948	1.127429
C	-3.328111	-0.774521	-1.318693
O	-3.457373	-0.692791	1.101083
C	2.030433	-0.72913	1.883133
O	3.682607	-1.123311	-0.78972
H	-2.524242	1.72187	0.913957
H	-2.986989	1.905474	-0.799412
H	1.757594	2.987508	-0.872577
H	1.124145	1.716757	-1.897546
H	3.021032	1.371111	0.479693
H	3.425091	1.31058	-1.226664
H	1.858055	-0.659765	-1.476195
H	1.233095	-2.949764	-0.26126
H	0.429394	-2.633558	1.272582
H	-1.233085	-2.542293	-0.424992
H	-0.288041	-1.525401	-1.485571
H	-0.774424	2.592809	1.777699
H	0.904258	3.267793	1.425316
H	-3.363169	-1.866429	-1.298209
H	-4.35191	-0.401972	-1.432819
H	-2.764145	-0.461811	-2.200962
H	-4.395518	-0.54641	0.930768
H	1.578803	-1.240467	2.726869
H	2.73222	0.061837	2.122404
H	4.163793	-1.100547	0.045622

9h_8

C	-2.707629	-0.081159	0.083309
C	-2.305396	1.405888	0.181767
H	-0.913897	-0.527163	1.161271
H	-0.921391	1.058689	-1.444322
C	1.51118	1.758861	-0.861285
C	0.347789	1.7486	0.113152
C	1.973408	0.415869	-1.464398
C	2.651524	-0.589634	-0.50699
C	1.73488	-1.318127	0.470493
C	0.671234	-2.244625	-0.095115
C	-0.627271	-1.62531	-0.681589
C	-1.185333	-0.442975	0.104963
C	-0.896011	1.033242	-0.349309
C	0.426257	2.399161	1.276844
C	-3.45222	-0.439093	-1.198654
O	-3.456951	-0.501108	1.224733
C	1.934411	-1.253234	1.788422
O	3.778198	0.017536	0.130903
H	-2.285441	1.70563	1.230604
H	-2.88518	2.130614	-0.393887
H	2.364163	2.269819	-0.403998

9h_9

C	-2.745657	-0.278542	0.01233
C	-2.508858	1.254492	0.085793
H	-0.937589	-0.477875	1.149973
H	-1.073294	0.9956	-1.509608
C	1.321748	1.90645	-0.887983
C	0.094547	1.884491	0.019786
C	2.576896	1.131072	-0.436119
C	2.535062	-0.397534	-0.543826
C	1.766633	-1.131273	0.554861
C	0.7441	-2.160616	0.126648
C	-0.483892	-1.611685	-0.634209
C	-1.196654	-0.46665	0.084391
C	-1.060071	1.016157	-0.414716
C	0.048885	2.655999	1.109226
C	-3.402666	-0.733304	-1.28255
O	-3.505252	-0.876927	1.059682
C	2.065975	-0.939933	1.841341
O	3.921089	-0.808538	-0.540465
H	-2.533907	1.586847	1.126505
H	-3.151703	1.903632	-0.513064
H	1.629626	2.949721	-1.005281

9h_10

C	-2.701051	-0.087583	0.083226
C	-2.305194	1.41142	0.195771
H	-0.916512	-0.525724	1.159683
H	-0.926553	1.074171	-1.436827
C	1.510881	1.761206	-0.856753
C	0.349812	1.750438	0.120498
C	1.968728	0.419042	-1.465042
C	2.647204	-0.59067	-0.51236
C	1.731581	-1.318883	0.466298
C	0.664721	-2.242529	-0.098034
C	-0.632395	-1.620564	-0.683959
C	-1.187624	-0.437715	0.103496
C	-0.897671	1.041295	-0.342068
C	0.432392	2.396742	1.286203
C	-3.448494	-0.426515	-1.202201
O	-3.368153	-0.656728	1.21044
C	1.934658	-1.256407	1.783817
O	3.777559	0.011953	0.123359
H	-2.282627	1.701652	1.24722
H	-2.883365	2.141916	-0.374805
H	2.366047	2.269152	-0.400192

9h_11

C	2.654384	-0.226854	-0.27117
C	2.352746	1.268751	-0.036807
H	0.785478	-0.355088	-1.260143
H	1.083319	0.662951	1.612148
C	-0.932519	1.891932	-0.95217
C	-0.207445	1.801966	0.375528
C	-1.748239	0.659164	-1.412148
C	-2.614983	-0.032283	-0.356728
C	-1.901246	-0.952278	0.624401
C	-0.967439	-2.01487	0.066995
C	0.536408	-1.781198	0.342803
C	1.110749	-0.488322	-0.225936
C	0.961365	0.86163	0.543351
C	-0.562796	2.60143	1.386966
C	3.471571	-0.877689	0.839529
O	3.275531	-0.450897	-1.538388
C	-2.184672	-0.882992	1.926915
O	-3.555922	-0.82475	-1.117738
H	2.257207	1.783155	-0.995673
H	3.024029	1.831596	0.615305
H	-0.211321	2.11722	-1.747601

H	1.217351	2.388388	-1.71196	H	1.038979	1.562544	-1.888613	H	1.216152	2.393809	-1.704841	H	-1.607499	2.750867	-0.902423
H	2.714097	0.651099	-2.234862	H	2.848772	1.419742	0.582931	H	2.707854	0.654902	-2.236813	H	-2.434273	0.995693	-2.195169
H	1.146557	-0.073308	-1.982903	H	3.402057	1.44739	-1.082429	H	1.13943	-0.066843	-1.982683	H	-1.104592	-0.084471	-1.883857
H	3.092961	-1.367421	-1.141961	H	2.099005	-0.663169	-1.516487	H	3.084509	-1.36809	-1.150616	H	-3.161764	0.727435	0.216626
H	1.124995	-2.878195	-0.867243	H	1.234786	-2.906781	-0.512373	H	1.116277	-2.877784	-0.87006	H	-1.238333	-2.981746	0.502215
H	0.376608	-2.916297	0.71558	H	0.394247	-2.693145	1.016172	H	0.368713	-2.912804	0.713302	H	-1.129661	-2.113088	-1.009821
H	-1.370793	-2.430372	-0.702559	H	-1.177232	-2.448443	-0.772307	H	-1.379094	-2.422028	-0.702565	H	1.084368	-2.630157	-0.085015
H	-0.480376	-1.339136	-1.72699	H	-0.19511	-1.29442	-1.641542	H	-0.48571	-1.334182	-1.729361	H	0.706644	-1.816388	1.424389
H	-0.408083	2.457201	1.966648	H	-0.813339	2.685675	1.765477	H	-0.40082	2.454853	1.97734	H	-0.060025	2.558545	2.34829
H	1.341068	2.896813	1.582073	H	0.886709	3.288408	1.386542	H	1.349137	2.890857	1.591432	H	-1.366613	3.323741	1.287898
H	-3.587067	-1.522184	-1.285068	H	-3.396707	-1.823745	-1.354008	H	-3.558735	-1.508014	-1.312501	H	3.522816	-1.962835	0.702973
H	-4.440615	0.027834	-1.18871	H	-4.442158	-0.395339	-1.299539	H	-4.449199	0.01829	-1.178165	H	4.490732	-0.482562	0.822485
H	-2.919373	-0.0981	-2.08976	H	-2.895016	-0.326454	-2.159236	H	-2.937038	-0.038557	-2.086529	H	3.044985	-0.685986	1.826938
H	-3.684922	-1.431085	1.10806	H	-3.116628	-0.606916	1.899116	H	-4.303912	-0.429604	1.152283	H	3.445635	-1.396515	-1.624621
H	1.31143	-1.814036	2.476939	H	1.568308	-1.499109	2.626658	H	1.312282	-1.816858	2.473169	H	-1.752033	-1.574047	2.644191
H	2.723933	-0.654036	2.224766	H	2.824581	-0.231237	2.155039	H	2.726534	-0.659475	2.219021	H	-2.849564	-0.122271	2.322901
H	3.453071	0.729029	0.693524	H	3.942618	-1.763949	-0.417502	H	3.456091	0.722895	0.688766	H	-4.093712	-1.311195	-0.482199

**9h_12**				**9h_13**				**9h_14**			
C	-2.73003	-0.234412	-0.037394	C	2.646958	-0.23293	-0.272627	C	-2.710452	-0.09494	0.078356
C	-2.434332	1.286891	-0.084558	C	2.352584	1.276421	-0.045751	C	-2.31441	1.402088	0.197325
H	-1.012015	-0.422065	1.246781	H	0.785258	-0.353584	-1.258206	H	-0.905152	-0.526835	1.163286
H	-0.880093	0.845592	-1.522594	H	1.087937	0.676307	1.608796	H	-0.936806	1.065271	-1.434833
C	1.400227	1.949193	-0.872061	C	-0.936088	1.890405	-0.956529	C	1.498544	1.761746	-0.859736
C	0.168153	1.860323	0.022183	C	-0.209002	1.805639	0.370435	C	0.338903	1.74982	0.11922
C	2.629548	1.084595	-0.50949	C	-1.749668	0.654803	-1.412733	C	1.95881	0.420347	-1.467848
C	2.425257	-0.434336	-0.571459	C	-2.613574	-0.036073	-0.354595	C	2.64615	-0.585468	-0.517397
C	1.732457	-1.065657	0.632471	C	-1.896489	-0.951721	0.628164	C	1.739234	-1.314151	0.469095
C	0.745869	-2.170317	0.341942	C	-0.960957	-2.013739	0.072442	C	0.673188	-2.244419	-0.085582
C	-0.521138	-1.713831	-0.419321	C	0.542499	-1.777243	0.346385	C	-0.626736	-1.629834	-0.672802
C	-1.199831	-0.476849	0.167738	C	1.113045	-0.483647	-0.22432	C	-1.188154	-0.44552	0.107287
C	-0.957387	0.958054	-0.43651	C	0.963056	0.869905	0.539425	C	-0.906211	1.034611	-0.340384
C	0.083864	2.611191	1.123705	C	-0.56504	2.607325	1.379959	C	0.421293	2.400047	1.282788
C	-3.313861	-0.778261	-1.333152	C	3.468504	-0.871081	0.84235	C	-3.437548	-0.430347	-1.215548
O	-3.588364	-0.705115	0.99911	O	3.176659	-0.595784	-1.548365	O	-3.507391	-0.648181	1.122486
C	2.041238	-0.719957	1.884209	C	-2.178302	-0.879288	1.930858	C	1.949685	-1.245958	1.785205
O	3.680873	-1.115043	-0.796276	O	-3.553549	-0.832706	-1.112439	O	3.779093	0.021545	0.109382
H	-2.527602	1.721013	0.913803	H	2.254463	1.785374	-1.007231	H	-2.286622	1.700718	1.24801
H	-2.997789	1.897772	-0.793698	H	3.02218	1.843495	0.604968	H	-2.896247	2.133628	-0.367818
H	1.741012	2.988593	-0.881747	H	-0.216413	2.115637	-1.75336	H	2.352987	2.2726	-0.405148
H	1.109664	1.71295	-1.901753	H	-1.613062	2.747832	-0.907942	H	1.200755	2.39281	-1.707879
H	3.015274	1.381001	0.470341	H	-1.105428	-0.088594	-1.884137	H	2.69349	0.658033	-2.24327
H	3.41343	1.316693	-1.237227	H	-2.437451	0.98829	-2.19552	H	1.129204	-0.069382	-1.981306
H	1.851489	-0.659859	-1.474588	H	-3.161497	0.723908	0.217339	H	3.081296	-1.363052	-1.156885
H	1.242891	-2.950796	-0.247645	H	-1.22979	-2.980075	0.510063	H	1.124139	-2.884048	-0.854331
H	0.442884	-2.630677	1.287035	H	-1.123924	-2.114873	-1.003993	H	0.381027	-2.909842	0.73117
H	-1.226185	-2.552259	-0.404918	H	1.093411	-2.622773	-0.083062	H	-1.371241	-2.433744	-0.685623
H	-0.286947	-1.538868	-1.474447	H	0.714578	-1.810728	1.427754	H	-0.482868	-1.348371	-1.719735
H	-0.781146	2.593408	1.77706	H	-0.060958	2.568537	2.340775	H	-0.41071	2.459499	1.975299
H	0.894357	3.271014	1.417352	H	-1.370537	3.327552	1.279637	H	1.337219	2.897423	1.584992
H	-3.354448	-1.869915	-1.310897	H	3.491646	-1.957991	0.731124	H	-3.551985	-1.511676	-1.32298
H	-4.33231	-0.400567	-1.456405	H	4.498756	-0.500873	0.803508	H	-4.433952	0.019027	-1.19798
H	-2.727041	-0.470939	-2.200902	H	3.070297	-0.632433	1.831401	H	-2.905134	-0.050333	-2.089542
H	-3.251942	-0.369563	1.83742	H	4.127698	-0.434879	-1.536276	H	-3.082713	-0.439589	1.961977
H	1.596448	-1.231385	2.731538	H	-1.743156	-1.567335	2.649503	H	1.334117	-1.806972	2.480224
H	2.741188	0.074252	2.117998	H	-2.844391	-0.118952	2.325561	H	2.741769	-0.644364	2.213525
H	4.16862	-1.083897	0.034989	H	-4.089649	-1.318578	-0.475044	H	3.460032	0.734157	0.674022

Table S4. SCF energies (Hartree) computed at the PCM/ mPW1PW91/6-31G* level of theory using coordinate files incorporated in Table S1.

9a_1_DP4+	SCF Energy (PCM/mPW1PW91/6-31G*) =-696.95182809	9a_2_DP4+	SCF Energy (PCM/mPW1PW91/6-31G*) =-696.95197954
9a_3_DP4+	SCF Energy (PCM/mPW1PW91/6-31G*) =-696.94708373	9a_4_DP4+	SCF Energy (PCM/mPW1PW91/6-31G*) =-696.94698528
9a_5_DP4+	SCF Energy (PCM/mPW1PW91/6-31G*) =-696.94850907	9a_6_DP4+	SCF Energy (PCM/mPW1PW91/6-31G*) =-696.94882964

9a_7_DP4+	SCF Energy (PCM/mPW1PW91/6-31G*) =-696.94991133	9a_8_DP4+	SCF Energy (PCM/mPW1PW91/6-31G*) =-696.95013221
9a_9_DP4+	SCF Energy (PCM/mPW1PW91/6-31G*) =-696.95032041	9a_10_DP4+	SCF Energy (PCM/mPW1PW91/6-31G*) =-696.94550900
9a_11_DP4+	SCF Energy (PCM/mPW1PW91/6-31G*) =-696.95123483	9a_12_DP4+	SCF Energy (PCM/mPW1PW91/6-31G*) =-696.95088670
9a_13_DP4+	SCF Energy (PCM/mPW1PW91/6-31G*) =-696.94720707	9b_1_DP4+	SCF Energy (PCM/mPW1PW91/6-31G*) =-696.95141682
9b_2_DP4+	SCF Energy (PCM/mPW1PW91/6-31G*) =-696.95254338	9b_3_DP4+	SCF Energy (PCM/mPW1PW91/6-31G*) =-696.94870302
9b_4_DP4+	SCF Energy (PCM/mPW1PW91/6-31G*) =-696.94993075	9b_5_DP4+	SCF Energy (PCM/mPW1PW91/6-31G*) =-696.95278074
9b_6_DP4+	SCF Energy (PCM/mPW1PW91/6-31G*) =-696.94448975	9b_7_DP4+	SCF Energy (PCM/mPW1PW91/6-31G*) =-696.94856288
9b_8_DP4+	SCF Energy (PCM/mPW1PW91/6-31G*) =-696.94509643	9b_9_DP4+	SCF Energy (PCM/mPW1PW91/6-31G*) =-696.94975268
9b_10_DP4+	SCF Energy (PCM/mPW1PW91/6-31G*) =-696.95095833	9b_11_DP4+	SCF Energy (PCM/mPW1PW91/6-31G*) =-696.94958843
9b_12_DP4+	SCF Energy (PCM/mPW1PW91/6-31G*) =-696.94626708	9b_13_DP4+	SCF Energy (PCM/mPW1PW91/6-31G*) =-696.94975157
9c_1_DP4+	SCF Energy (PCM/mPW1PW91/6-31G*) =-696.95196893	9c_2_DP4+	SCF Energy (PCM/mPW1PW91/6-31G*) =-696.95229624
9c_3_DP4+	SCF Energy (PCM/mPW1PW91/6-31G*) =-696.94982954	9c_4_DP4+	SCF Energy (PCM/mPW1PW91/6-31G*) =-696.95006491
9c_5_DP4+	SCF Energy (PCM/mPW1PW91/6-31G*) =-696.95119700	9c_6_DP4+	SCF Energy (PCM/mPW1PW91/6-31G*) =-696.95167364
9c_7_DP4+	SCF Energy (PCM/mPW1PW91/6-31G*) =-696.95010625	9c_8_DP4+	SCF Energy (PCM/mPW1PW91/6-31G*) =-696.94871308
9c_9_DP4+	SCF Energy (PCM/mPW1PW91/6-31G*) =-696.95026755	9c_10_DP4+	SCF Energy (PCM/mPW1PW91/6-31G*) =-696.95061292
9c_11_DP4+	SCF Energy (PCM/mPW1PW91/6-31G*) =-696.94675456	9c_12_DP4+	SCF Energy (PCM/mPW1PW91/6-31G*) =-696.94692474
9c_13_DP4+	SCF Energy (PCM/mPW1PW91/6-31G*) =-696.94784561	9c_14_DP4+	SCF Energy (PCM/mPW1PW91/6-31G*) =-696.94814039
9c_15_DP4+	SCF Energy (PCM/mPW1PW91/6-31G*) =-696.95009544	9c_16_DP4+	SCF Energy (PCM/mPW1PW91/6-31G*) =-696.94919931
9c_17_DP4+	SCF Energy (PCM/mPW1PW91/6-31G*) =-696.94947936	9c_18_DP4+	SCF Energy (PCM/mPW1PW91/6-31G*) =-696.94807322
9d_1_DP4+	SCF Energy (PCM/mPW1PW91/6-31G*) =-696.95047740	9d_2_DP4+	SCF Energy (PCM/mPW1PW91/6-31G*) =-696.95152322
9d_3_DP4+	SCF Energy (PCM/mPW1PW91/6-31G*) =-696.95185250	9d_4_DP4+	SCF Energy (PCM/mPW1PW91/6-31G*) =-696.94922517
9d_5_DP4+	SCF Energy (PCM/mPW1PW91/6-31G*) =-696.95027600	9d_6_DP4+	SCF Energy (PCM/mPW1PW91/6-31G*) =-696.94662414
9d_7_DP4+	SCF Energy (PCM/mPW1PW91/6-31G*) =-696.94729939	9d_8_DP4+	SCF Energy (PCM/mPW1PW91/6-31G*) =-696.95056892
9d_9_DP4+	SCF Energy (PCM/mPW1PW91/6-31G*) =-696.94756311	9e_1_DP4+	SCF Energy (PCM/mPW1PW91/6-31G*) =-696.94879354
9e_2_DP4+	SCF Energy (PCM/mPW1PW91/6-31G*) =-696.94893285	9e_3_DP4+	SCF Energy (PCM/mPW1PW91/6-31G*) =-696.94763358
9e_4_DP4+	SCF Energy (PCM/mPW1PW91/6-31G*) =-696.94771759	9e_5_DP4+	SCF Energy (PCM/mPW1PW91/6-31G*) =-696.94702445
9e_6_DP4+	SCF Energy (PCM/mPW1PW91/6-31G*) =-696.94567459	9e_7_DP4+	SCF Energy (PCM/mPW1PW91/6-31G*) =-696.94433143
9e_8_DP4+	SCF Energy (PCM/mPW1PW91/6-31G*) =-696.94442420	9f_1_DP4+	SCF Energy (PCM/mPW1PW91/6-31G*) =-696.95262428
9f_2_DP4+	SCF Energy (PCM/mPW1PW91/6-31G*) =-696.95281593	9f_3_DP4+	SCF Energy (PCM/mPW1PW91/6-31G*) =-696.95025682
9f_4_DP4+	SCF Energy (PCM/mPW1PW91/6-31G*) =-696.95047752	9f_5_DP4+	SCF Energy (PCM/mPW1PW91/6-31G*) =-696.95166389
9f_6_DP4+	SCF Energy (PCM/mPW1PW91/6-31G*) =-696.95333646	9f_7_DP4+	SCF Energy (PCM/mPW1PW91/6-31G*) =-696.95179307
9f_8_DP4+	SCF Energy (PCM/mPW1PW91/6-31G*) =-696.95096659	9f_9_DP4+	SCF Energy (PCM/mPW1PW91/6-31G*) =-696.94790742
9f_10_DP4+	SCF Energy (PCM/mPW1PW91/6-31G*) =-696.95047527	9f_11_DP4+	SCF Energy (PCM/mPW1PW91/6-31G*) =-696.94760336
9f_12_DP4+	SCF Energy (PCM/mPW1PW91/6-31G*) =-696.95066588	9f_13_DP4+	SCF Energy (PCM/mPW1PW91/6-31G*) =-696.95239316
9f_14_DP4+	SCF Energy (PCM/mPW1PW91/6-31G*) =-696.94872894	9f_15_DP4+	SCF Energy (PCM/mPW1PW91/6-31G*) =-696.94894527
9f_16_DP4+	SCF Energy (PCM/mPW1PW91/6-31G*) =-696.95122761	9f_17_DP4+	SCF Energy (PCM/mPW1PW91/6-31G*) =-696.94906605
9f_18_DP4+	SCF Energy (PCM/mPW1PW91/6-31G*) =-696.94940655	9g_1_DP4+	SCF Energy (PCM/mPW1PW91/6-31G*) =-696.94985663
9g_2_DP4+	SCF Energy (PCM/mPW1PW91/6-31G*) =-696.94983799	9g_3_DP4+	SCF Energy (PCM/mPW1PW91/6-31G*) =-696.94649303
9g_4_DP4+	SCF Energy (PCM/mPW1PW91/6-31G*) =-696.94651521	9g_5_DP4+	SCF Energy (PCM/mPW1PW91/6-31G*) =-696.94714284
9g_6_DP4+	SCF Energy (PCM/mPW1PW91/6-31G*) =-696.94694392	9g_7_DP4+	SCF Energy (PCM/mPW1PW91/6-31G*) =-696.94771970
9g_8_DP4+	SCF Energy (PCM/mPW1PW91/6-31G*) =-696.94776702	9g_9_DP4+	SCF Energy (PCM/mPW1PW91/6-31G*) =-696.94464267
9g_10_DP4+	SCF Energy (PCM/mPW1PW91/6-31G*) =-696.94768813	9h_1_DP4+	SCF Energy (PCM/mPW1PW91/6-31G*) =-696.95227693
9h_2_DP4+	SCF Energy (PCM/mPW1PW91/6-31G*) =-696.95249891	9h_3_DP4+	SCF Energy (PCM/mPW1PW91/6-31G*) =-696.94741880
9h_4_DP4+	SCF Energy (PCM/mPW1PW91/6-31G*) =-696.94764091	9h_5_DP4+	SCF Energy (PCM/mPW1PW91/6-31G*) =-696.95308323
9h_6_DP4+	SCF Energy (PCM/mPW1PW91/6-31G*) =-696.94912048	9h_7_DP4+	SCF Energy (PCM/mPW1PW91/6-31G*) =-696.94927003
9h_8_DP4+	SCF Energy (PCM/mPW1PW91/6-31G*) =-696.95049933	9h_9_DP4+	SCF Energy (PCM/mPW1PW91/6-31G*) =-696.94816833
9h_10_DP4+	SCF Energy (PCM/mPW1PW91/6-31G*) =-696.95066550	9h_11_DP4+	SCF Energy (PCM/mPW1PW91/6-31G*) =-696.95183489
9h_12_DP4+	SCF Energy (PCM/mPW1PW91/6-31G*) =-696.94973388	9h_13_DP4+	SCF Energy (PCM/mPW1PW91/6-31G*) =-696.95207403
9h_14_DP4+	SCF Energy (PCM/mPW1PW91/6-31G*) =-696.95121199		

Table S5. Imaginary frequencies and absolute energy values of all conformers of isomers **9a**–**9h** used after optimization at the B3LYP/6-311G(d,p) level of theory as required for DP4+.

9a_1_DP4+

Imaginary Freq = 0

Zero-point correction= 0.335127 (Hartree/Particle)

Thermal correction to Energy= 0.351283

Thermal correction to Enthalpy= 0.352227

Thermal correction to Gibbs Free Energy= 0.293321

Sum of electronic and zero-point Energies= -696.971926

Sum of electronic and thermal Energies= -696.955771

Sum of electronic and thermal Enthalpies= -696.954827

Sum of electronic and thermal Free Energies= -697.013732

9a_2_DP4+

Imaginary Freq = 0

Zero-point correction= 0.335120 (Hartree/Particle)

Thermal correction to Energy= 0.351292

Thermal correction to Enthalpy= 0.352236

Thermal correction to Gibbs Free Energy= 0.293216

Sum of electronic and zero-point Energies= -696.972282

Sum of electronic and thermal Energies= -696.956110

Sum of electronic and thermal Enthalpies= -696.955166

Sum of electronic and thermal Free Energies= -697.014186

9a_3_DP4+

Imaginary Freq = 0

Zero-point correction= 0.334382 (Hartree/Particle)

Thermal correction to Energy= 0.350983

Thermal correction to Enthalpy= 0.351927

Thermal correction to Gibbs Free Energy= 0.291244

Sum of electronic and zero-point Energies= -696.968462

Sum of electronic and thermal Energies= -696.951861

Sum of electronic and thermal Enthalpies= -696.950917

Sum of electronic and thermal Free Energies= -697.011600

9a_4_DP4+

Imaginary Freq = 0

Zero-point correction= 0.334475 (Hartree/Particle)

Thermal correction to Energy= 0.351008

Thermal correction to Enthalpy= 0.351952

Thermal correction to Gibbs Free Energy= 0.291547

Sum of electronic and zero-point Energies= -696.968621

Sum of electronic and thermal Energies= -696.952089

Sum of electronic and thermal Enthalpies= -696.951145

Sum of electronic and thermal Free Energies= -697.011549

9a_5_DP4+

Imaginary Freq = 0

Zero-point correction= 0.335016 (Hartree/Particle)

Thermal correction to Energy= 0.351415

Thermal correction to Enthalpy= 0.352360

Thermal correction to Gibbs Free Energy= 0.292287

Sum of electronic and zero-point Energies= -696.968928

Sum of electronic and thermal Energies= -696.952528

Sum of electronic and thermal Enthalpies= -696.951584

Sum of electronic and thermal Free Energies= -697.011657

9a_6_DP4+

Imaginary Freq = 0

Zero-point correction= 0.335125 (Hartree/Particle)

Thermal correction to Energy= 0.351451

Thermal correction to Enthalpy= 0.352395

Thermal correction to Gibbs Free Energy= 0.292680

Sum of electronic and zero-point Energies= -696.969253

Sum of electronic and thermal Energies= -696.952928

Sum of electronic and thermal Enthalpies= -696.951984

Sum of electronic and thermal Free Energies= -697.011698

9a_7_DP4+

Imaginary Freq = 0

Zero-point correction= 0.334912 (Hartree/Particle)

Thermal correction to Energy= 0.351252

Thermal correction to Enthalpy= 0.352197

Thermal correction to Gibbs Free Energy= 0.292276

Sum of electronic and zero-point Energies= -696.970197

Sum of electronic and thermal Energies= -696.953856

Sum of electronic and thermal Enthalpies= -696.952912

Sum of electronic and thermal Free Energies= -697.012833

9a_8_DP4+

Imaginary Freq = 0

Zero-point correction= 0.335091 (Hartree/Particle)

Thermal correction to Energy= 0.351349

Thermal correction to Enthalpy= 0.352293

Thermal correction to Gibbs Free Energy= 0.292616

Sum of electronic and zero-point Energies= -696.970394

Sum of electronic and thermal Energies= -696.954136

Sum of electronic and thermal Enthalpies= -696.953192

Sum of electronic and thermal Free Energies= -697.012869

9a_9_DP4+

Imaginary Freq = 0

Zero-point correction= 0.335026 (Hartree/Particle)

Thermal correction to Energy= 0.351251

Thermal correction to Enthalpy= 0.352195

9a_10_DP4+

Imaginary Freq = 0

Zero-point correction= 0.334377 (Hartree/Particle)

Thermal correction to Energy= 0.350971

Thermal correction to Enthalpy= 0.351916

Thermal correction to Gibbs Free Energy= 0.293044

Sum of electronic and zero-point Energies= -696.970797

Sum of electronic and thermal Energies= -696.954572

Sum of electronic and thermal Enthalpies= -696.953627

Sum of electronic and thermal Free Energies= -697.012778

9a_11_DP4+

Imaginary Freq = 0

Zero-point correction= 0.334951 (Hartree/Particle)

Thermal correction to Energy= 0.351248

Thermal correction to Enthalpy= 0.352192

Thermal correction to Gibbs Free Energy= 0.292959

Sum of electronic and zero-point Energies= -696.971171

Sum of electronic and thermal Energies= -696.954874

Sum of electronic and thermal Enthalpies= -696.953929

Sum of electronic and thermal Free Energies= -697.013163

9a_13_DP4+

Imaginary Freq = 0

Zero-point correction= 0.334969 (Hartree/Particle)

Thermal correction to Energy= 0.351395

Thermal correction to Enthalpy= 0.352339

Thermal correction to Gibbs Free Energy= 0.292318

Sum of electronic and zero-point Energies= -696.967965

Sum of electronic and thermal Energies= -696.951539

Sum of electronic and thermal Enthalpies= -696.950595

Sum of electronic and thermal Free Energies= -697.010616

9b_2_DP4+

Imaginary Freq = 0

Zero-point correction= 0.335189 (Hartree/Particle)

Thermal correction to Energy= 0.351356

Thermal correction to Enthalpy= 0.352300

Thermal correction to Gibbs Free Energy= 0.293308

Sum of electronic and zero-point Energies= -696.972438

Sum of electronic and thermal Energies= -696.956272

Sum of electronic and thermal Enthalpies= -696.955328

Sum of electronic and thermal Free Energies= -697.014319

9b_4_DP4+

Imaginary Freq = 0

Zero-point correction= 0.334951 (Hartree/Particle)

Thermal correction to Energy= 0.351220

Thermal correction to Enthalpy= 0.352165

Thermal correction to Gibbs Free Energy= 0.292962

Sum of electronic and zero-point Energies= -696.969662

Sum of electronic and thermal Energies= -696.953392

Sum of electronic and thermal Enthalpies= -696.952448

Sum of electronic and thermal Free Energies= -697.011650

9b_6_DP4+

Imaginary Freq = 0

Zero-point correction= 0.334677 (Hartree/Particle)

Thermal correction to Energy= 0.351075

Thermal correction to Gibbs Free Energy= 0.291276

Sum of electronic and zero-point Energies= -696.967317

Sum of electronic and thermal Energies= -696.950722

Sum of electronic and thermal Enthalpies= -696.949778

Sum of electronic and thermal Free Energies= -697.010418

9a_12_DP4+

Imaginary Freq = 0

Zero-point correction= 0.334847 (Hartree/Particle)

Thermal correction to Energy= 0.351188

Thermal correction to Enthalpy= 0.352132

Thermal correction to Gibbs Free Energy= 0.292799

Sum of electronic and zero-point Energies= -696.970913

Sum of electronic and thermal Energies= -696.954571

Sum of electronic and thermal Enthalpies= -696.953627

Sum of electronic and thermal Free Energies= -697.012960

9b_1_DP4+

Imaginary Freq = 0

Zero-point correction= 0.334910 (Hartree/Particle)

Thermal correction to Energy= 0.351210

Thermal correction to Enthalpy= 0.352154

Thermal correction to Gibbs Free Energy= 0.292811

Sum of electronic and zero-point Energies= -696.971650

Sum of electronic and thermal Energies= -696.955351

Sum of electronic and thermal Enthalpies= -696.954406

Sum of electronic and thermal Free Energies= -697.013750

9b_3_DP4+

Imaginary Freq = 0

Zero-point correction= 0.334713 (Hartree/Particle)

Thermal correction to Energy= 0.351127

Thermal correction to Enthalpy= 0.352071

Thermal correction to Gibbs Free Energy= 0.292495

Sum of electronic and zero-point Energies= -696.968747

Sum of electronic and thermal Energies= -696.952334

Sum of electronic and thermal Enthalpies= -696.951390

Sum of electronic and thermal Free Energies= -697.010966

9b_5_DP4+

Imaginary Freq = 0

Zero-point correction= 0.335296 (Hartree/Particle)

Thermal correction to Energy= 0.351463

Thermal correction to Enthalpy= 0.352408

Thermal correction to Gibbs Free Energy= 0.293393

Sum of electronic and zero-point Energies= -696.972447

Sum of electronic and thermal Energies= -696.956280

Sum of electronic and thermal Enthalpies= -696.955336

Sum of electronic and thermal Free Energies= -697.014350

9b_7_DP4+

Imaginary Freq = 0

Zero-point correction= 0.334737 (Hartree/Particle)

Thermal correction to Energy= 0.351181

Thermal correction to Enthalpy= 0.352020
Thermal correction to Gibbs Free Energy= 0.292394
Sum of electronic and zero-point Energies= -696.965382
Sum of electronic and thermal Energies= -696.948983
Sum of electronic and thermal Enthalpies= -696.948039
Sum of electronic and thermal Free Energies= -697.007665

9b_8_DP4+
Imaginary Freq = 0
Zero-point correction= 0.334796 (Hartree/Particle)
Thermal correction to Energy= 0.351153
Thermal correction to Enthalpy= 0.352097
Thermal correction to Gibbs Free Energy= 0.292298
Sum of electronic and zero-point Energies= -696.965974
Sum of electronic and thermal Energies= -696.949618
Sum of electronic and thermal Enthalpies= -696.948674
Sum of electronic and thermal Free Energies= -697.008473

9b_10_DP4+
Imaginary Freq = 0
Zero-point correction= 0.335216 (Hartree/Particle)
Thermal correction to Energy= 0.351393
Thermal correction to Enthalpy= 0.352337
Thermal correction to Gibbs Free Energy= 0.293453
Sum of electronic and zero-point Energies= -696.970174
Sum of electronic and thermal Energies= -696.953997
Sum of electronic and thermal Enthalpies= -696.953053
Sum of electronic and thermal Free Energies= -697.011937

9b_12_DP4+
Imaginary Freq = 0
Zero-point correction= 0.335059 (Hartree/Particle)
Thermal correction to Energy= 0.351328
Thermal correction to Enthalpy= 0.352272
Thermal correction to Gibbs Free Energy= 0.293102
Sum of electronic and zero-point Energies= -696.966285
Sum of electronic and thermal Energies= -696.950015
Sum of electronic and thermal Enthalpies= -696.949071
Sum of electronic and thermal Free Energies= -697.008242

9c_1_DP4+
Imaginary Freq = 0
Zero-point correction= 0.334956 (Hartree/Particle)
Thermal correction to Energy= 0.351268
Thermal correction to Enthalpy= 0.352213
Thermal correction to Gibbs Free Energy= 0.292683
Sum of electronic and zero-point Energies= -696.972454
Sum of electronic and thermal Energies= -696.956141
Sum of electronic and thermal Enthalpies= -696.955197
Sum of electronic and thermal Free Energies= -697.014727

9c_3_DP4+
Imaginary Freq = 0
Zero-point correction= 0.334567 (Hartree/Particle)

Thermal correction to Enthalpy= 0.352126
Thermal correction to Gibbs Free Energy= 0.292423
Sum of electronic and zero-point Energies= -696.969599
Sum of electronic and thermal Energies= -696.953154
Sum of electronic and thermal Enthalpies= -696.952210
Sum of electronic and thermal Free Energies= -697.011913

9b_9_DP4+
Imaginary Freq = 0
Zero-point correction= 0.334980 (Hartree/Particle)
Thermal correction to Energy= 0.351295
Thermal correction to Enthalpy= 0.352240
Thermal correction to Gibbs Free Energy= 0.293024
Sum of electronic and zero-point Energies= -696.969288
Sum of electronic and thermal Energies= -696.952972
Sum of electronic and thermal Enthalpies= -696.952028
Sum of electronic and thermal Free Energies= -697.011243

9b_11_DP4+
Imaginary Freq = 0
Zero-point correction= 0.334879 (Hartree/Particle)
Thermal correction to Energy= 0.351241
Thermal correction to Enthalpy= 0.352185
Thermal correction to Gibbs Free Energy= 0.292600
Sum of electronic and zero-point Energies= -696.970496
Sum of electronic and thermal Energies= -696.954134
Sum of electronic and thermal Enthalpies= -696.953190
Sum of electronic and thermal Free Energies= -697.012775

9b_13_DP4+
Imaginary Freq = 0
Zero-point correction= 0.335011 (Hartree/Particle)
Thermal correction to Energy= 0.351313
Thermal correction to Enthalpy= 0.352257
Thermal correction to Gibbs Free Energy= 0.292932
Sum of electronic and zero-point Energies= -696.969429
Sum of electronic and thermal Energies= -696.953128
Sum of electronic and thermal Enthalpies= -696.952183
Sum of electronic and thermal Free Energies= -697.011508

9c_2_DP4+
Imaginary Freq = 0
Zero-point correction= 0.335032 (Hartree/Particle)
Thermal correction to Energy= 0.351305
Thermal correction to Enthalpy= 0.352249
Thermal correction to Gibbs Free Energy= 0.292814
Sum of electronic and zero-point Energies= -696.972779
Sum of electronic and thermal Energies= -696.956506
Sum of electronic and thermal Enthalpies= -696.955562
Sum of electronic and thermal Free Energies= -697.014997

9c_4_DP4+
Imaginary Freq = 0
Zero-point correction= 0.334680 (Hartree/Particle)

Thermal correction to Energy= 0.351086

Thermal correction to Enthalpy= 0.352030

Thermal correction to Gibbs Free Energy= 0.291808

Sum of electronic and zero-point Energies= -696.970850

Sum of electronic and thermal Energies= -696.954331

Sum of electronic and thermal Enthalpies= -696.953387

Sum of electronic and thermal Free Energies= -697.013609

9c_5_DP4+

Imaginary Freq = 0

Zero-point correction= 0.335168 (Hartree/Particle)

Thermal correction to Energy= 0.351486

Thermal correction to Enthalpy= 0.352430

Thermal correction to Gibbs Free Energy= 0.292946

Sum of electronic and zero-point Energies= -696.971217

Sum of electronic and thermal Energies= -696.954899

Sum of electronic and thermal Enthalpies= -696.953955

Sum of electronic and thermal Free Energies= -697.013440

9c_7_DP4+

Imaginary Freq = 0

Zero-point correction= 0.334964 (Hartree/Particle)

Thermal correction to Energy= 0.351312

Thermal correction to Enthalpy= 0.352256

Thermal correction to Gibbs Free Energy= 0.292808

Sum of electronic and zero-point Energies= -696.970834

Sum of electronic and thermal Energies= -696.954486

Sum of electronic and thermal Enthalpies= -696.953542

Sum of electronic and thermal Free Energies= -697.012991

9c_9_DP4+

Imaginary Freq = 0

Zero-point correction= 0.335010 (Hartree/Particle)

Thermal correction to Energy= 0.351331

Thermal correction to Enthalpy= 0.352275

Thermal correction to Gibbs Free Energy= 0.292931

Sum of electronic and zero-point Energies= -696.971132

Sum of electronic and thermal Energies= -696.954810

Sum of electronic and thermal Enthalpies= -696.953866

Sum of electronic and thermal Free Energies= -697.013211

9c_11_DP4+

Imaginary Freq = 0

Zero-point correction= 0.334467 (Hartree/Particle)

Thermal correction to Energy= 0.350991

Thermal correction to Enthalpy= 0.351935

Thermal correction to Gibbs Free Energy= 0.292005

Sum of electronic and zero-point Energies= -696.968025

Sum of electronic and thermal Energies= -696.951501

Sum of electronic and thermal Enthalpies= -696.950557

Sum of electronic and thermal Free Energies= -697.010487

9c_13_DP4+

Imaginary Freq = 0

Thermal correction to Energy= 0.351150

Thermal correction to Enthalpy= 0.352094

Thermal correction to Gibbs Free Energy= 0.291987

Sum of electronic and zero-point Energies= -696.971166

Sum of electronic and thermal Energies= -696.954697

Sum of electronic and thermal Enthalpies= -696.953753

Sum of electronic and thermal Free Energies= -697.013859

9c_6_DP4+

Imaginary Freq = 0

Zero-point correction= 0.335310 (Hartree/Particle)

Thermal correction to Energy= 0.351568

Thermal correction to Enthalpy= 0.352513

Thermal correction to Gibbs Free Energy= 0.293131

Sum of electronic and zero-point Energies= -696.971640

Sum of electronic and thermal Energies= -696.955381

Sum of electronic and thermal Enthalpies= -696.954437

Sum of electronic and thermal Free Energies= -697.013818

9c_8_DP4+

Imaginary Freq = 0

Zero-point correction= 0.334622 (Hartree/Particle)

Thermal correction to Energy= 0.351149

Thermal correction to Enthalpy= 0.352094

Thermal correction to Gibbs Free Energy= 0.291813

Sum of electronic and zero-point Energies= -696.969915

Sum of electronic and thermal Energies= -696.953387

Sum of electronic and thermal Enthalpies= -696.952443

Sum of electronic and thermal Free Energies= -697.012723

9c_10_DP4+

Imaginary Freq = 0

Zero-point correction= 0.334910 (Hartree/Particle)

Thermal correction to Energy= 0.351239

Thermal correction to Enthalpy= 0.352183

Thermal correction to Gibbs Free Energy= 0.292665

Sum of electronic and zero-point Energies= -696.971332

Sum of electronic and thermal Energies= -696.955002

Sum of electronic and thermal Enthalpies= -696.954058

Sum of electronic and thermal Free Energies= -697.013577

9c_12_DP4+

Imaginary Freq = 0

Zero-point correction= 0.334536 (Hartree/Particle)

Thermal correction to Energy= 0.351042

Thermal correction to Enthalpy= 0.351986

Thermal correction to Gibbs Free Energy= 0.292062

Sum of electronic and zero-point Energies= -696.968264

Sum of electronic and thermal Energies= -696.951758

Sum of electronic and thermal Enthalpies= -696.950814

Sum of electronic and thermal Free Energies= -697.010738

9c_14_DP4+

Imaginary Freq = 0

Zero-point correction= 0.334675 (Hartree/Particle)
Thermal correction to Energy= 0.351150
Thermal correction to Enthalpy= 0.352094
Thermal correction to Gibbs Free Energy= 0.292390
Sum of electronic and zero-point Energies= -696.968417
Sum of electronic and thermal Energies= -696.951943
Sum of electronic and thermal Enthalpies= -696.950999
Sum of electronic and thermal Free Energies= -697.010703

Zero-point correction= 0.334760 (Hartree/Particle)
Thermal correction to Energy= 0.351202
Thermal correction to Enthalpy= 0.352147
Thermal correction to Gibbs Free Energy= 0.292553
Sum of electronic and zero-point Energies= -696.968686
Sum of electronic and thermal Energies= -696.952243
Sum of electronic and thermal Enthalpies= -696.951299
Sum of electronic and thermal Free Energies= -697.010893

9c_15_DP4+
Imaginary Freq = 0
Zero-point correction= 0.335116 (Hartree/Particle)
Thermal correction to Energy= 0.351479
Thermal correction to Enthalpy= 0.352424
Thermal correction to Gibbs Free Energy= 0.292720
Sum of electronic and zero-point Energies= -696.970424
Sum of electronic and thermal Energies= -696.954060
Sum of electronic and thermal Enthalpies= -696.953116
Sum of electronic and thermal Free Energies= -697.012820

9c_16_DP4+
Imaginary Freq = 0
Zero-point correction= 0.334786 (Hartree/Particle)
Thermal correction to Energy= 0.351208
Thermal correction to Enthalpy= 0.352153
Thermal correction to Gibbs Free Energy= 0.292089
Sum of electronic and zero-point Energies= -696.969668
Sum of electronic and thermal Energies= -696.953245
Sum of electronic and thermal Enthalpies= -696.952301
Sum of electronic and thermal Free Energies= -697.012364

9c_17_DP4+
Imaginary Freq = 0
Zero-point correction= 0.334851 (Hartree/Particle)
Thermal correction to Energy= 0.351245
Thermal correction to Enthalpy= 0.352189
Thermal correction to Gibbs Free Energy= 0.292119
Sum of electronic and zero-point Energies= -696.969952
Sum of electronic and thermal Energies= -696.953558
Sum of electronic and thermal Enthalpies= -696.952614
Sum of electronic and thermal Free Energies= -697.012684

9c_18_DP4+
Imaginary Freq = 0
Zero-point correction= 0.334891 (Hartree/Particle)
Thermal correction to Energy= 0.351181
Thermal correction to Enthalpy= 0.352125
Thermal correction to Gibbs Free Energy= 0.292933
Sum of electronic and zero-point Energies= -696.968647
Sum of electronic and thermal Energies= -696.952356
Sum of electronic and thermal Enthalpies= -696.951412
Sum of electronic and thermal Free Energies= -697.010604

9d_1_DP4+
Imaginary Freq = 0
Zero-point correction= 0.335210 (Hartree/Particle)
Thermal correction to Energy= 0.351329
Thermal correction to Enthalpy= 0.352274
Thermal correction to Gibbs Free Energy= 0.293529
Sum of electronic and zero-point Energies= -696.970530
Sum of electronic and thermal Energies= -696.954412
Sum of electronic and thermal Enthalpies= -696.953467
Sum of electronic and thermal Free Energies= -697.012212

9d_2_DP4+
Imaginary Freq = 0
Zero-point correction= 0.335436 (Hartree/Particle)
Thermal correction to Energy= 0.351438
Thermal correction to Enthalpy= 0.352383
Thermal correction to Gibbs Free Energy= 0.293908
Sum of electronic and zero-point Energies= -696.971332
Sum of electronic and thermal Energies= -696.955330
Sum of electronic and thermal Enthalpies= -696.954386
Sum of electronic and thermal Free Energies= -697.012860

9d_3_DP4+
Imaginary Freq = 0
Zero-point correction= 0.335492 (Hartree/Particle)
Thermal correction to Energy= 0.351506
Thermal correction to Enthalpy= 0.352451
Thermal correction to Gibbs Free Energy= 0.293891
Sum of electronic and zero-point Energies= -696.971392
Sum of electronic and thermal Energies= -696.955377
Sum of electronic and thermal Enthalpies= -696.954433
Sum of electronic and thermal Free Energies= -697.012993

9d_4_DP4+
Imaginary Freq = 0
Zero-point correction= 0.335216 (Hartree/Particle)
Thermal correction to Energy= 0.351322
Thermal correction to Enthalpy= 0.352266
Thermal correction to Gibbs Free Energy= 0.293571
Sum of electronic and zero-point Energies= -696.969268
Sum of electronic and thermal Energies= -696.953161
Sum of electronic and thermal Enthalpies= -696.952217
Sum of electronic and thermal Free Energies= -697.010913

9d_5_DP4+

9d_6_DP4+

Imaginary Freq = 0

Zero-point correction= 0.335269 (Hartree/Particle)

Thermal correction to Energy= 0.351344

Thermal correction to Enthalpy= 0.352288

Thermal correction to Gibbs Free Energy= 0.293558

Sum of electronic and zero-point Energies= -696.970245

Sum of electronic and thermal Energies= -696.954170

Sum of electronic and thermal Enthalpies= -696.953226

Sum of electronic and thermal Free Energies= -697.011956

9d_7_DP4+

Imaginary Freq = 0

Zero-point correction= 0.335539 (Hartree/Particle)

Thermal correction to Energy= 0.351544

Thermal correction to Enthalpy= 0.352488

Thermal correction to Gibbs Free Energy= 0.294019

Sum of electronic and zero-point Energies= -696.970092

Sum of electronic and thermal Energies= -696.954087

Sum of electronic and thermal Enthalpies= -696.953143

Sum of electronic and thermal Free Energies= -697.011612

9d_9_DP4+

Imaginary Freq = 0

Zero-point correction= 0.335038 (Hartree/Particle)

Thermal correction to Energy= 0.351304

Thermal correction to Enthalpy= 0.352248

Thermal correction to Gibbs Free Energy= 0.292929

Sum of electronic and zero-point Energies= -696.968143

Sum of electronic and thermal Energies= -696.951877

Sum of electronic and thermal Enthalpies= -696.950933

Sum of electronic and thermal Free Energies= -697.010252

9e_2_DP4+

Imaginary Freq = 0

Zero-point correction= 0.335251 (Hartree/Particle)

Thermal correction to Energy= 0.351340

Thermal correction to Enthalpy= 0.352284

Thermal correction to Gibbs Free Energy= 0.293540

Sum of electronic and zero-point Energies= -696.968949

Sum of electronic and thermal Energies= -696.952860

Sum of electronic and thermal Enthalpies= -696.951916

Sum of electronic and thermal Free Energies= -697.010659

9e_4_DP4+

Imaginary Freq = 0

Zero-point correction= 0.335177 (Hartree/Particle)

Thermal correction to Energy= 0.351290

Thermal correction to Enthalpy= 0.352234

Thermal correction to Gibbs Free Energy= 0.293422

Sum of electronic and zero-point Energies= -696.967791

Sum of electronic and thermal Energies= -696.951678

Sum of electronic and thermal Enthalpies= -696.950734

Sum of electronic and thermal Free Energies= -697.009546

Imaginary Freq = 0

Zero-point correction= 0.334581 (Hartree/Particle)

Thermal correction to Energy= 0.350984

Thermal correction to Enthalpy= 0.351928

Thermal correction to Gibbs Free Energy= 0.291741

Sum of electronic and zero-point Energies= -696.967907

Sum of electronic and thermal Energies= -696.951504

Sum of electronic and thermal Enthalpies= -696.950560

Sum of electronic and thermal Free Energies= -697.010747

9d_8_DP4+

Imaginary Freq = 0

Zero-point correction= 0.335539 (Hartree/Particle)

Thermal correction to Energy= 0.351544

Thermal correction to Enthalpy= 0.352488

Thermal correction to Gibbs Free Energy= 0.294019

Sum of electronic and zero-point Energies= -696.970092

Sum of electronic and thermal Energies= -696.954087

Sum of electronic and thermal Enthalpies= -696.953143

Sum of electronic and thermal Free Energies= -697.011612

9e_1_DP4+

Imaginary Freq = 0

Zero-point correction= 0.335284 (Hartree/Particle)

Thermal correction to Energy= 0.351370

Thermal correction to Enthalpy= 0.352314

Thermal correction to Gibbs Free Energy= 0.293543

Sum of electronic and zero-point Energies= -696.968861

Sum of electronic and thermal Energies= -696.952775

Sum of electronic and thermal Enthalpies= -696.951831

Sum of electronic and thermal Free Energies= -697.010602

9e_3_DP4+

Imaginary Freq = 0

Zero-point correction= 0.335254 (Hartree/Particle)

Thermal correction to Energy= 0.351340

Thermal correction to Enthalpy= 0.352284

Thermal correction to Gibbs Free Energy= 0.293540

Sum of electronic and zero-point Energies= -696.967675

Sum of electronic and thermal Energies= -696.951589

Sum of electronic and thermal Enthalpies= -696.950645

Sum of electronic and thermal Free Energies= -697.009389

9e_5_DP4+

Imaginary Freq = 0

Zero-point correction= 0.335119 (Hartree/Particle)

Thermal correction to Energy= 0.351292

Thermal correction to Enthalpy= 0.352236

Thermal correction to Gibbs Free Energy= 0.293287

Sum of electronic and zero-point Energies= -696.967419

Sum of electronic and thermal Energies= -696.951246

Sum of electronic and thermal Enthalpies= -696.950302

Sum of electronic and thermal Free Energies= -697.009251

9e_6_DP4+

Imaginary Freq = 0

Zero-point correction= 0.335157 (Hartree/Particle)

Thermal correction to Energy= 0.351310

Thermal correction to Enthalpy= 0.352254

Thermal correction to Gibbs Free Energy= 0.293402

Sum of electronic and zero-point Energies= -696.966105

Sum of electronic and thermal Energies= -696.949951

Sum of electronic and thermal Enthalpies= -696.949007

Sum of electronic and thermal Free Energies= -697.007859

9e_7_DP4+

Imaginary Freq = 0

Zero-point correction= 0.334818 (Hartree/Particle)

Thermal correction to Energy= 0.351099

Thermal correction to Enthalpy= 0.352043

Thermal correction to Gibbs Free Energy= 0.292790

Sum of electronic and zero-point Energies= -696.964852

Sum of electronic and thermal Energies= -696.948572

Sum of electronic and thermal Enthalpies= -696.947627

Sum of electronic and thermal Free Energies= -697.006881

9e_8_DP4+

Imaginary Freq = 0

Zero-point correction= 0.334799 (Hartree/Particle)

Thermal correction to Energy= 0.351079

Thermal correction to Enthalpy= 0.352023

Thermal correction to Gibbs Free Energy= 0.292808

Sum of electronic and zero-point Energies= -696.964892

Sum of electronic and thermal Energies= -696.948612

Sum of electronic and thermal Enthalpies= -696.947668

Sum of electronic and thermal Free Energies= -697.006883

9f_1_DP4+

Imaginary Freq = 0

Zero-point correction= 0.335089 (Hartree/Particle)

Thermal correction to Energy= 0.351341

Thermal correction to Enthalpy= 0.352285

Thermal correction to Gibbs Free Energy= 0.292947

Sum of electronic and zero-point Energies= -696.972726

Sum of electronic and thermal Energies= -696.956474

Sum of electronic and thermal Enthalpies= -696.955529

Sum of electronic and thermal Free Energies= -697.014868

9f_2_DP4+

Imaginary Freq = 0

Zero-point correction= 0.334985 (Hartree/Particle)

Thermal correction to Energy= 0.351274

Thermal correction to Enthalpy= 0.352218

Thermal correction to Gibbs Free Energy= 0.292806

Sum of electronic and zero-point Energies= -696.972842

Sum of electronic and thermal Energies= -696.956554

Sum of electronic and thermal Enthalpies= -696.955610

Sum of electronic and thermal Free Energies= -697.015021

9f_3_DP4+

Imaginary Freq = 0

Zero-point correction= 0.334870 (Hartree/Particle)

Thermal correction to Energy= 0.351304

Thermal correction to Enthalpy= 0.352249

Thermal correction to Gibbs Free Energy= 0.292312

Sum of electronic and zero-point Energies= -696.971007

Sum of electronic and thermal Energies= -696.954573

Sum of electronic and thermal Enthalpies= -696.953628

Sum of electronic and thermal Free Energies= -697.013565

9f_4_DP4+

Imaginary Freq = 0

Zero-point correction= 0.334748 (Hartree/Particle)

Thermal correction to Energy= 0.351228

Thermal correction to Enthalpy= 0.352172

Thermal correction to Gibbs Free Energy= 0.292111

Sum of electronic and zero-point Energies= -696.971085

Sum of electronic and thermal Energies= -696.954605

Sum of electronic and thermal Enthalpies= -696.953661

Sum of electronic and thermal Free Energies= -697.013722

9f_5_DP4+

Imaginary Freq = 0

Zero-point correction= 0.335416 (Hartree/Particle)

Thermal correction to Energy= 0.351656

Thermal correction to Enthalpy= 0.352600

Thermal correction to Gibbs Free Energy= 0.293329

Sum of electronic and zero-point Energies= -696.971481

Sum of electronic and thermal Energies= -696.955240

Sum of electronic and thermal Enthalpies= -696.954296

Sum of electronic and thermal Free Energies= -697.013567

9f_6_DP4+

Imaginary Freq = 0

Zero-point correction= 0.335135 (Hartree/Particle)

Thermal correction to Energy= 0.351372

Thermal correction to Enthalpy= 0.352317

Thermal correction to Gibbs Free Energy= 0.293020

Sum of electronic and zero-point Energies= -696.973159

Sum of electronic and thermal Energies= -696.956922

Sum of electronic and thermal Enthalpies= -696.955978

9f_7_DP4+

Imaginary Freq = 0

Zero-point correction= 0.335351 (Hartree/Particle)

Thermal correction to Energy= 0.351607

Thermal correction to Enthalpy= 0.352551

Thermal correction to Gibbs Free Energy= 0.293228

Sum of electronic and zero-point Energies= -696.971512

Sum of electronic and thermal Energies= -696.955257

Sum of electronic and thermal Enthalpies= -696.954313

Sum of electronic and thermal Free Energies= -697.015274

9f_8_DP4+

Imaginary Freq = 0

Zero-point correction= 0.334985 (Hartree/Particle)

Thermal correction to Energy= 0.351375

Thermal correction to Enthalpy= 0.352320

Thermal correction to Gibbs Free Energy= 0.292525

Sum of electronic and zero-point Energies= -696.971310

Sum of electronic and thermal Energies= -696.954919

Sum of electronic and thermal Enthalpies= -696.953975

Sum of electronic and thermal Free Energies= -697.013770

9f_10_DP4+

Imaginary Freq = 0

Zero-point correction= 0.334975 (Hartree/Particle)

Thermal correction to Energy= 0.351319

Thermal correction to Enthalpy= 0.352263

Thermal correction to Gibbs Free Energy= 0.292818

Sum of electronic and zero-point Energies= -696.971147

Sum of electronic and thermal Energies= -696.954802

Sum of electronic and thermal Enthalpies= -696.953858

Sum of electronic and thermal Free Energies= -697.013304

9f_12_DP4+

Imaginary Freq = 0

Zero-point correction= 0.334794 (Hartree/Particle)

Thermal correction to Energy= 0.351213

Thermal correction to Enthalpy= 0.352157

Thermal correction to Gibbs Free Energy= 0.292437

Sum of electronic and zero-point Energies= -696.971344

Sum of electronic and thermal Energies= -696.954925

Sum of electronic and thermal Enthalpies= -696.953981

Sum of electronic and thermal Free Energies= -697.013701

9f_14_DP4+

Imaginary Freq = 0

Zero-point correction= 0.334995 (Hartree/Particle)

Thermal correction to Energy= 0.351371

Thermal correction to Enthalpy= 0.352316

Thermal correction to Gibbs Free Energy= 0.292891

Sum of electronic and zero-point Energies= -696.968828

Sum of electronic and thermal Energies= -696.952452

Sum of electronic and thermal Enthalpies= -696.951508

Sum of electronic and thermal Free Energies= -697.010933

9f_16_DP4+

Imaginary Freq = 0

Sum of electronic and thermal Free Energies= -697.013636

9f_9_DP4+

Imaginary Freq = 0

Zero-point correction= 0.334671 (Hartree/Particle)

Thermal correction to Energy= 0.351125

Thermal correction to Enthalpy= 0.352069

Thermal correction to Gibbs Free Energy= 0.292357

Sum of electronic and zero-point Energies= -696.968586

Sum of electronic and thermal Energies= -696.952132

Sum of electronic and thermal Enthalpies= -696.951188

Sum of electronic and thermal Free Energies= -697.010900

9f_11_DP4+

Imaginary Freq = 0

Zero-point correction= 0.334713 (Hartree/Particle)

Thermal correction to Energy= 0.351166

Thermal correction to Enthalpy= 0.352110

Thermal correction to Gibbs Free Energy= 0.292400

Sum of electronic and zero-point Energies= -696.968476

Sum of electronic and thermal Energies= -696.952023

Sum of electronic and thermal Enthalpies= -696.951079

Sum of electronic and thermal Free Energies= -697.010789

9f_13_DP4+

Imaginary Freq = 0

Zero-point correction= 0.335523 (Hartree/Particle)

Thermal correction to Energy= 0.351719

Thermal correction to Enthalpy= 0.352663

Thermal correction to Gibbs Free Energy= 0.293505

Sum of electronic and zero-point Energies= -696.971798

Sum of electronic and thermal Energies= -696.955602

Sum of electronic and thermal Enthalpies= -696.954658

Sum of electronic and thermal Free Energies= -697.013816

9f_15_DP4+

Imaginary Freq = 0

Zero-point correction= 0.334958 (Hartree/Particle)

Thermal correction to Energy= 0.351330

Thermal correction to Enthalpy= 0.352274

Thermal correction to Gibbs Free Energy= 0.292905

Sum of electronic and zero-point Energies= -696.968915

Sum of electronic and thermal Energies= -696.952543

Sum of electronic and thermal Enthalpies= -696.951598

Sum of electronic and thermal Free Energies= -697.010968

9f_17_DP4+

Imaginary Freq = 0

Zero-point correction= 0.334983 (Hartree/Particle)

Thermal correction to Energy= 0.351329

Thermal correction to Enthalpy= 0.352273

Thermal correction to Gibbs Free Energy= 0.292750

Sum of electronic and zero-point Energies= -696.971629

Sum of electronic and thermal Energies= -696.955283

Sum of electronic and thermal Enthalpies= -696.954339

Sum of electronic and thermal Free Energies= -697.013862

9f_18_DP4+

Imaginary Freq = 0

Zero-point correction= 0.335086 (Hartree/Particle)

Thermal correction to Energy= 0.351317

Thermal correction to Enthalpy= 0.352261

Thermal correction to Gibbs Free Energy= 0.293257

Sum of electronic and zero-point Energies= -696.969389

Sum of electronic and thermal Energies= -696.953158

Sum of electronic and thermal Enthalpies= -696.952214

Sum of electronic and thermal Free Energies= -697.011218

9g_2_DP4+

Imaginary Freq = 0

Zero-point correction= 0.335177 (Hartree/Particle)

Thermal correction to Energy= 0.351346

Thermal correction to Enthalpy= 0.352290

Thermal correction to Gibbs Free Energy= 0.293262

Sum of electronic and zero-point Energies= -696.969866

Sum of electronic and thermal Energies= -696.953697

Sum of electronic and thermal Enthalpies= -696.952753

Sum of electronic and thermal Free Energies= -697.011781

9g_4_DP4+

Imaginary Freq = 0

Zero-point correction= 0.334657 (Hartree/Particle)

Thermal correction to Energy= 0.351044

Thermal correction to Enthalpy= 0.351988

Thermal correction to Gibbs Free Energy= 0.292432

Sum of electronic and zero-point Energies= -696.966693

Sum of electronic and thermal Energies= -696.950306

Sum of electronic and thermal Enthalpies= -696.949362

Sum of electronic and thermal Free Energies= -697.008917

9g_6_DP4+

Imaginary Freq = 0

Zero-point correction= 0.334978 (Hartree/Particle)

Thermal correction to Energy= 0.351293

Zero-point correction= 0.335130 (Hartree/Particle)

Thermal correction to Energy= 0.351351

Thermal correction to Enthalpy= 0.352295

Thermal correction to Gibbs Free Energy= 0.293328

Sum of electronic and zero-point Energies= -696.969256

Sum of electronic and thermal Energies= -696.953035

Sum of electronic and thermal Enthalpies= -696.952091

Sum of electronic and thermal Free Energies= -697.011058

9g_1_DP4+

Imaginary Freq = 0

Zero-point correction= 0.335219 (Hartree/Particle)

Thermal correction to Energy= 0.351379

Thermal correction to Enthalpy= 0.352323

Thermal correction to Gibbs Free Energy= 0.293338

Sum of electronic and zero-point Energies= -696.969811

Sum of electronic and thermal Energies= -696.953652

Sum of electronic and thermal Enthalpies= -696.952707

Sum of electronic and thermal Free Energies= -697.011692

9g_3_DP4+

Imaginary Freq = 0

Zero-point correction= 0.334746 (Hartree/Particle)

Thermal correction to Energy= 0.351109

Thermal correction to Enthalpy= 0.352053

Thermal correction to Gibbs Free Energy= 0.292555

Sum of electronic and zero-point Energies= -696.966635

Sum of electronic and thermal Energies= -696.950272

Sum of electronic and thermal Enthalpies= -696.949328

Sum of electronic and thermal Free Energies= -697.008826

9g_5_DP4+

Imaginary Freq = 0

Zero-point correction= 0.334978 (Hartree/Particle)

Thermal correction to Energy= 0.351285

Thermal correction to Enthalpy= 0.352229

Thermal correction to Gibbs Free Energy= 0.292953

Sum of electronic and zero-point Energies= -696.967848

Sum of electronic and thermal Energies= -696.951541

Sum of electronic and thermal Enthalpies= -696.950597

Sum of electronic and thermal Free Energies= -697.009874

9g_7_DP4+

Imaginary Freq = 0

Zero-point correction= 0.335006 (Hartree/Particle)

Thermal correction to Energy= 0.351314

Thermal correction to Enthalpy= 0.352237

Thermal correction to Gibbs Free Energy= 0.292944

Sum of electronic and zero-point Energies= -696.967794

Sum of electronic and thermal Energies= -696.951479

Sum of electronic and thermal Enthalpies= -696.950534

Sum of electronic and thermal Free Energies= -697.009828

9g_8_DP4+

Imaginary Freq = 0

Zero-point correction= 0.334959 (Hartree/Particle)

Thermal correction to Energy= 0.351276

Thermal correction to Enthalpy= 0.352220

Thermal correction to Gibbs Free Energy= 0.292869

Sum of electronic and zero-point Energies= -696.967294

Sum of electronic and thermal Energies= -696.950978

Sum of electronic and thermal Enthalpies= -696.950034

Sum of electronic and thermal Free Energies= -697.009385

9g_10_DP4+

Imaginary Freq = 0

Zero-point correction= 0.335202 (Hartree/Particle)

Thermal correction to Energy= 0.351333

Thermal correction to Enthalpy= 0.352277

Thermal correction to Gibbs Free Energy= 0.293478

Sum of electronic and zero-point Energies= -696.966877

Sum of electronic and thermal Energies= -696.950746

Sum of electronic and thermal Enthalpies= -696.949802

Sum of electronic and thermal Free Energies= -697.008601

9h_2_DP4+

Imaginary Freq = 0

Zero-point correction= 0.335304 (Hartree/Particle)

Thermal correction to Energy= 0.351378

Thermal correction to Enthalpy= 0.352322

Thermal correction to Gibbs Free Energy= 0.293689

Sum of electronic and zero-point Energies= -696.972136

Sum of electronic and thermal Energies= -696.956062

Sum of electronic and thermal Enthalpies= -696.955118

Sum of electronic and thermal Free Energies= -697.013750

9h_4_DP4+

Imaginary Freq = 0

Zero-point correction= 0.334511 (Hartree/Particle)

Thermal correction to Energy= 0.351063

Thermal correction to Enthalpy= 0.352007

Thermal correction to Gibbs Free Energy= 0.291471

Thermal correction to Enthalpy= 0.352258

Thermal correction to Gibbs Free Energy= 0.292929

Sum of electronic and zero-point Energies= -696.967266

Sum of electronic and thermal Energies= -696.950958

Sum of electronic and thermal Enthalpies= -696.950014

Sum of electronic and thermal Free Energies= -697.009343

9g_9_DP4+

Imaginary Freq = 0

Zero-point correction= 0.334468 (Hartree/Particle)

Thermal correction to Energy= 0.350993

Thermal correction to Enthalpy= 0.351937

Thermal correction to Gibbs Free Energy= 0.291955

Sum of electronic and zero-point Energies= -696.965258

Sum of electronic and thermal Energies= -696.948733

Sum of electronic and thermal Enthalpies= -696.947789

Sum of electronic and thermal Free Energies= -697.007771

9h_1_DP4+

Imaginary Freq = 0

Zero-point correction= 0.335339 (Hartree/Particle)

Thermal correction to Energy= 0.351421

Thermal correction to Enthalpy= 0.352365

Thermal correction to Gibbs Free Energy= 0.293715

Sum of electronic and zero-point Energies= -696.972080

Sum of electronic and thermal Energies= -696.955999

Sum of electronic and thermal Enthalpies= -696.955055

Sum of electronic and thermal Free Energies= -697.013705

9h_3_DP4+

Imaginary Freq = 0

Zero-point correction= 0.334552 (Hartree/Particle)

Thermal correction to Energy= 0.351102

Thermal correction to Enthalpy= 0.352046

Thermal correction to Gibbs Free Energy= 0.290915

Sum of electronic and zero-point Energies= -696.968606

Sum of electronic and thermal Energies= -696.952056

Sum of electronic and thermal Enthalpies= -696.951111

Sum of electronic and thermal Free Energies= -697.012243

9h_5_DP4+

Imaginary Freq = 0

Zero-point correction= 0.335458 (Hartree/Particle)

Thermal correction to Energy= 0.351500

Thermal correction to Enthalpy= 0.352444

Thermal correction to Gibbs Free Energy= 0.293868

Sum of electronic and zero-point Energies= -696.968660

Sum of electronic and thermal Energies= -696.952109

Sum of electronic and thermal Enthalpies= -696.951164

Sum of electronic and thermal Free Energies= -697.011700

9h_6_DP4+

Imaginary Freq = 0

Zero-point correction= 0.335313 (Hartree/Particle)

Thermal correction to Energy= 0.351589

Thermal correction to Enthalpy= 0.352534

Thermal correction to Gibbs Free Energy= 0.293071

Sum of electronic and zero-point Energies= -696.969048

Sum of electronic and thermal Energies= -696.952772

Sum of electronic and thermal Enthalpies= -696.951828

Sum of electronic and thermal Free Energies= -697.011290

9h_8_DP4+

Imaginary Freq = 0

Zero-point correction= 0.335320 (Hartree/Particle)

Thermal correction to Energy= 0.351499

Thermal correction to Enthalpy= 0.352443

Thermal correction to Gibbs Free Energy= 0.293171

Sum of electronic and zero-point Energies= -696.970199

Sum of electronic and thermal Energies= -696.954021

Sum of electronic and thermal Enthalpies= -696.953077

Sum of electronic and thermal Free Energies= -697.012349

9h_10_DP4+

Imaginary Freq = 0

Zero-point correction= 0.335235 (Hartree/Particle)

Thermal correction to Energy= 0.351430

Thermal correction to Enthalpy= 0.352374

Thermal correction to Gibbs Free Energy= 0.293038

Sum of electronic and zero-point Energies= -696.970280

Sum of electronic and thermal Energies= -696.954085

Sum of electronic and thermal Enthalpies= -696.953141

Sum of electronic and thermal Free Energies= -697.012477

9h_12_DP4+

Imaginary Freq = 0

Zero-point correction= 0.335317 (Hartree/Particle)

Thermal correction to Energy= 0.351605

Thermal correction to Enthalpy= 0.352549

Thermal correction to Gibbs Free Energy= 0.293071

Sum of electronic and zero-point Energies= -696.969482

Sum of electronic and thermal Energies= -696.953194

Sum of electronic and zero-point Energies= -696.972479

Sum of electronic and thermal Energies= -696.956436

Sum of electronic and thermal Enthalpies= -696.955492

Sum of electronic and thermal Free Energies= -697.014069

9h_7_DP4+

Imaginary Freq = 0

Zero-point correction= 0.335193 (Hartree/Particle)

Thermal correction to Energy= 0.351504

Thermal correction to Enthalpy= 0.352448

Thermal correction to Gibbs Free Energy= 0.292888

Sum of electronic and zero-point Energies= -696.969158

Sum of electronic and thermal Energies= -696.952846

Sum of electronic and thermal Enthalpies= -696.951902

Sum of electronic and thermal Free Energies= -697.011463

9h_9_DP4+

Imaginary Freq = 0

Zero-point correction= 0.334745 (Hartree/Particle)

Thermal correction to Energy= 0.351219

Thermal correction to Enthalpy= 0.352163

Thermal correction to Gibbs Free Energy= 0.291927

Sum of electronic and zero-point Energies= -696.968895

Sum of electronic and thermal Energies= -696.952421

Sum of electronic and thermal Enthalpies= -696.951477

Sum of electronic and thermal Free Energies= -697.011713

9h_11_DP4+

Imaginary Freq = 0

Zero-point correction= 0.335036 (Hartree/Particle)

Thermal correction to Energy= 0.351305

Thermal correction to Enthalpy= 0.352249

Thermal correction to Gibbs Free Energy= 0.293123

Sum of electronic and zero-point Energies= -696.971536

Sum of electronic and thermal Energies= -696.955267

Sum of electronic and thermal Enthalpies= -696.954323

Sum of electronic and thermal Free Energies= -697.013450

9h_13_DP4+

Imaginary Freq = 0

Zero-point correction= 0.334886 (Hartree/Particle)

Thermal correction to Energy= 0.351202

Thermal correction to Enthalpy= 0.352147

Thermal correction to Gibbs Free Energy= 0.292891

Sum of electronic and zero-point Energies= -696.971746

Sum of electronic and thermal Energies= -696.955430

Sum of electronic and thermal Enthalpies= -696.952250

Sum of electronic and thermal Free Energies= -697.011728

Sum of electronic and thermal Enthalpies= -696.954486

Sum of electronic and thermal Free Energies= -697.013741

9h_14_DP4+

Imaginary Freq = 0

Zero-point correction= 0.335342 (Hartree/Particle)

Thermal correction to Energy= 0.351526

Thermal correction to Enthalpy= 0.352470

Thermal correction to Gibbs Free Energy= 0.292948

Sum of electronic and zero-point Energies= -696.970665

Sum of electronic and thermal Energies= -696.954481

Sum of electronic and thermal Enthalpies= -696.953537

Table S6. Experiment and calculated ^{13}C NMR chemical shifts of stereoisomers **9a–9h**.

		$\delta_{calc.}$							
No.	$\delta_{exp.}$	**9a**	**9b**	**9c**	**9d**	**9e**	**9f**	**9g**	**9h**
11	70.7	73.7	70.7	73.1	71.2	76.1	70.6	75.4	70.8
10	40.3	36.1	37.5	35.4	37.9	35.9	37.2	35.4	38.0
7	33.2	33.5	35.6	32.5	34.1	33.7	33.1	35.6	34.4
8	151.1	151.0	146.8	150.3	147.7	147.4	149.7	146.3	150.3
6	32.3	32.6	31.9	33.7	28.5	28.0	34.4	30.8	33.4
5	75.7	72.5	75.0	75.1	75.2	74.7	75.6	75.8	73.2
4	150.9	151.5	152.7	151.6	154.1	153.4	151.7	151.9	151.1
3	32.5	35.2	34.4	33.9	34.8	34.8	35.2	33.0	36.3
2	29.9	27.1	25.7	29.1	28.8	30.5	30.6	28.3	28.3
1	58.1	52.2	54.0	51.4	57.2	58.1	54.1	55.5	55.1
9	40.0	45.3	38.8	44.9	37.5	40.2	41.8	40.6	41.8
13	110.0	110.2	113.4	109.6	113.9	113.0	110.5	112.4	111.0
12	21.6	25.2	28.4	24.9	28.8	24.3	20.7	24.2	21.2
14	114.6	114.5	116.1	115.4	111.2	110.8	115.8	115.9	115.1

Table S7. Mean absolute error (MAE) values, maximum deviation (MD) values and correlation coefficient (R^2) analysis of four stereoisomers **9a–9h**.

Stereoisomers	MAE (ppm)	MD (ppm)	R^2
**9a**	2.3	5.8	0.9952
**9b**	2.5	6.8	0.9950
**9c**	2.1	6.3	0.9956
**9d**	2.6	6.7	0.9950
**9e**	2.5	5.4	0.9954
**9f**	1.4	3.9	0.9982
**9g**	2.2	4.8	0.9961
**9h**	1.5	3.7	0.9982

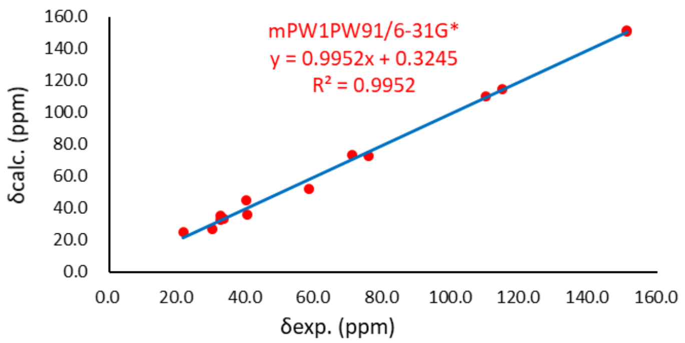

Figure S90. Regression analysis of experimental *versus* calculated ^{13}C NMR **c**hemical shifts at mPW1PW91/6-31G* level using GIAO method of **9a**; linear fitting is shown as a line (blue).

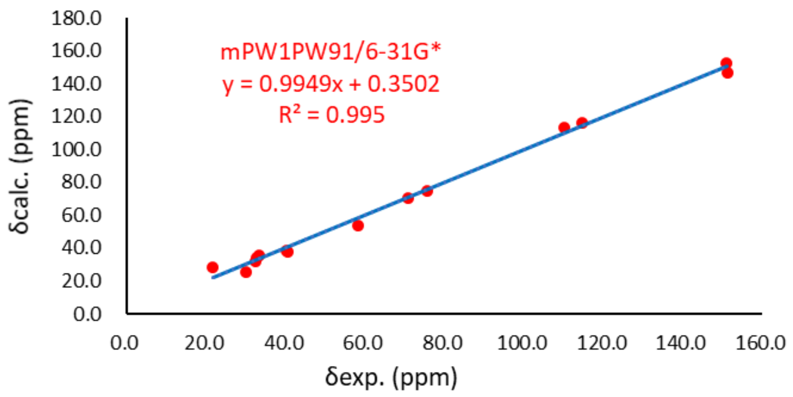

Figure S91. Regression analysis of experimental *versus* calculated ^{13}C NMR **c**hemical shifts at mPW1PW91/6-31G* level using GIAO method of **9b**; linear fitting is shown as a line (blue).

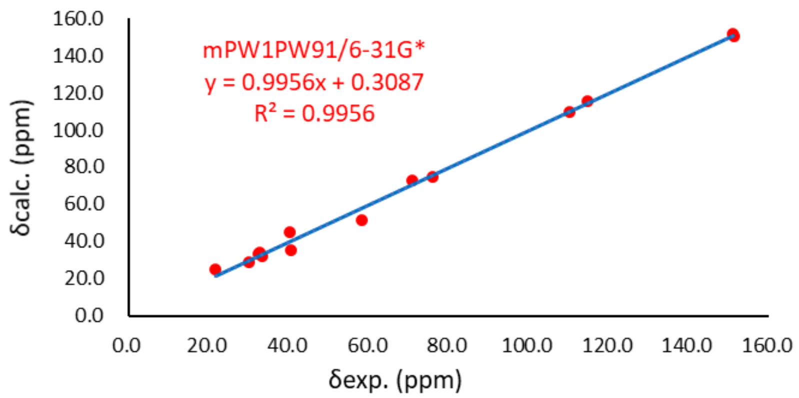

Figure S92. Regression analysis of experimental *versus* calculated ^{13}C NMR **c**hemical shifts at mPW1PW91/6-31G* level using GIAO method of **9c**; linear fitting is shown as a line (blue).

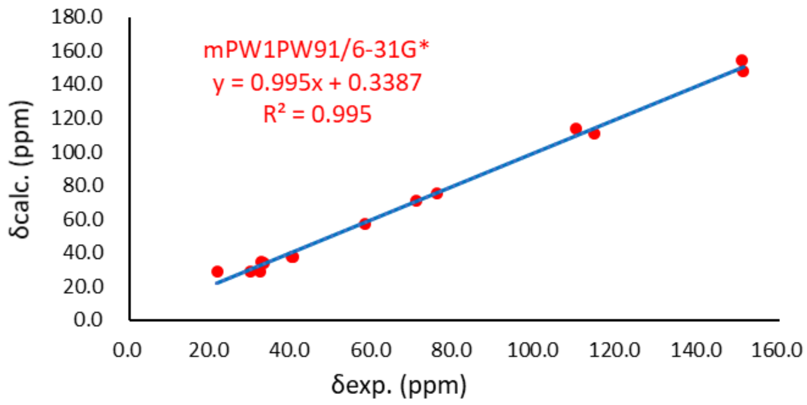

Figure S93. Regression analysis of experimental *versus* calculated ^{13}C NMR **c**hemical shifts at mPW1PW91/6-31G* level using GIAO method of **9d**; linear fitting is shown as a line (blue).

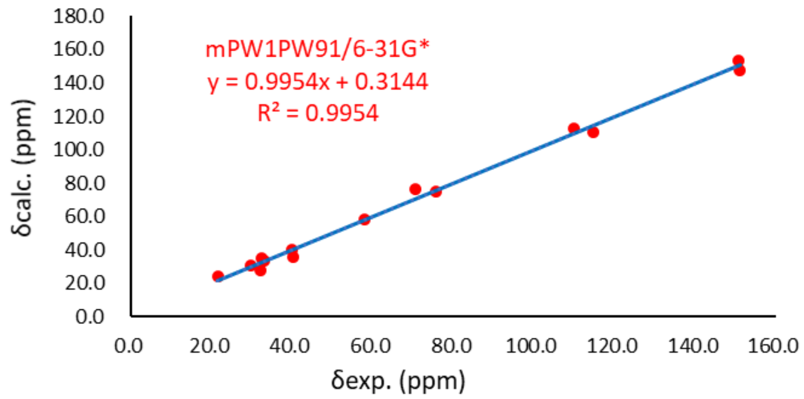

Figure S94. Regression analysis of experimental *versus* calculated ^{13}C NMR **c**hemical shifts at mPW1PW91/6-31G* level using GIAO method of **9e**; linear fitting is shown as a line (blue).

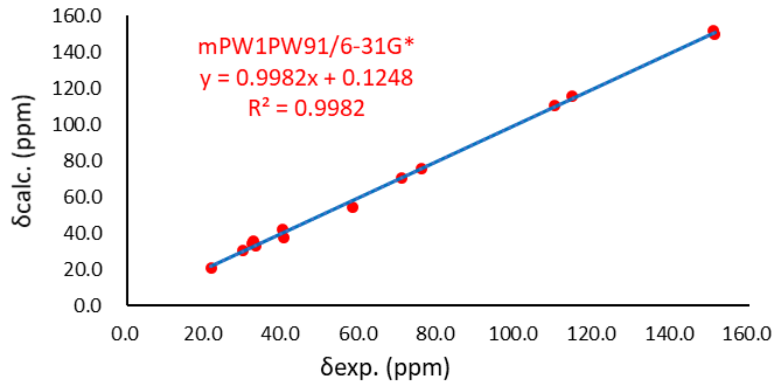

Figure S95. Regression analysis of experimental *versus* calculated ^{13}C NMR **c**hemical shifts at mPW1PW91/6-31G* level using GIAO method of **9f**; linear fitting is shown as a line (blue).

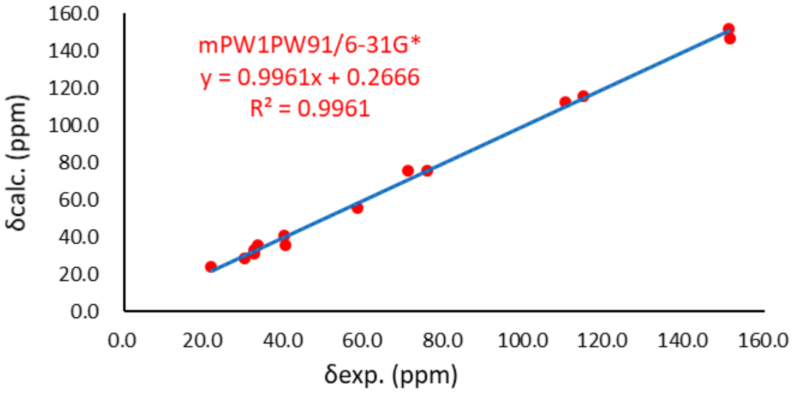

Figure S96. Regression analysis of experimental *versus* calculated ^{13}C NMR chemical shifts at mPW1PW91/6-31G* level using GIAO method of **9g**; linear fitting is shown as a line (blue).

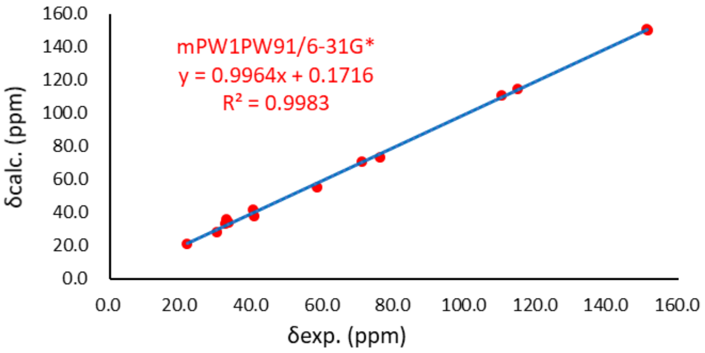

Figure S97. Regression analysis of experimental *versus* calculated ^{13}C NMR chemical shifts at mPW1PW91/6-31G* level using GIAO method of **9h**; linear fitting is shown as a line (blue).

Functional		Solvent?		Basis Set		Type of Data				
mPW1PW91		PCM		6-31G(d)		Shielding Tensors				
		DP4+	0.00%	0.00%	0.00%	0.00%	0.00%	96.73%	0.00%	3.27%
Nuclei	sp2?	xperimenta	Isomer 1	Isomer 2	Isomer 3	Isomer 4	Isomer 5	Isomer 6	Isomer 7	Isomer 8
C		70.7	121.200592	125.110271	121.379852	124.968309	119.781659	124.28159	119.986568	124.5199
C		40.3	157.085254	157.335803	157.390907	157.28339	158.788205	156.412934	158.946993	156.1082
C		33.2	159.506851	159.138195	160.199449	160.95431	160.966021	160.378385	158.743921	159.5432
C	x	151.1	47.5183783	51.3902424	47.62528	50.9202805	50.5968151	48.2982481	51.1340108	48.06823
C		32.3	160.41153	162.778468	158.990029	166.390521	166.446662	159.093125	163.421214	160.5086
C		75.7	122.352314	121.005477	119.484283	121.154009	121.098285	119.542622	119.65854	122.2425
C	x	150.9	47.0051036	45.7257709	46.3390253	44.717655	44.754766	46.3526849	45.6943868	47.23202
C		32.5	157.89411	160.361928	158.805139	160.296954	159.89847	158.276862	161.288701	157.7112
C		29.9	165.664903	168.766077	163.40156	166.092747	163.978271	162.747949	165.833471	165.396
C		58.1	141.670979	141.328155	142.062011	138.61209	137.193347	140.197184	139.379465	139.6405
C		40.0	148.255046	156.051097	148.299233	157.606266	154.610271	151.969081	153.858407	152.3824
C	x	110.0	86.3898373	83.8193636	86.4961071	83.6926657	84.0026716	85.9558828	84.0739748	85.83477
C		21.6	167.424342	166.135538	167.413848	166.082984	170.039516	172.243826	169.786635	172.2612
C	x	114.6	82.26337	81.1346699	80.9419757	86.2697602	86.0759777	80.8429074	80.6997886	80.90848

Functional	Solvent?		Basis Set		Type of Data			
mPW1PW91	PCM		6-31G(d)		Shielding Tensors			
	Isomer 1	Isomer 2	Isomer 3	Isomer 4	Isomer 5	Isomer 6	Isomer 7	Isomer 8
sDP4+ (H data)	–	–	–	–	–	–	–	–
sDP4+ (C data)	0.00%	0.00%	0.04%	0.00%	0.00%	83.56%	0.00%	16.40%
sDP4+ (all data)	0.00%	0.00%	0.04%	0.00%	0.00%	83.56%	0.00%	16.40%
uDP4+ (H data)	–	–	–	–	–	–	–	–
uDP4+ (C data)	0.02%	0.00%	0.22%	0.00%	0.02%	85.07%	0.04%	14.63%
uDP4+ (all data)	0.02%	0.00%	0.22%	0.00%	0.02%	85.07%	0.04%	14.63%
DP4+ (H data)	–	–	–	–	–	–	–	–
DP4+ (C data)	0.00%	0.00%	0.00%	0.00%	0.00%	96.73%	0.00%	3.27%
DP4+ (all data)	0.00%	0.00%	0.00%	0.00%	0.00%	96.73%	0.00%	3.27%

Figure S98. DP4+ evaluation of theoretical and experimental data of **9**.

2.2 ECD calculation for compound 1, 3, 6, and 8

Conformational search was carried out by using the torsional sampling (MCMM) method and OPLS_2005 force field with the conformational search in an energy window of 21 kJ/mol. Conformers above 1% Boltzmann populations were re-optimized at the MPW1PW91/6-31G(d) level with the IEFPCM solvent model for MeCN. Frequency analysis was also carried out to confirm that the re-optimized geometries were at the energy minima. ECD spectra were obtained by TDDFT calculations displayed with the identical functional basis set and solvent model as the energy optimization. Finally, the Boltzmann-averaged ECD spectra of the compounds were obtained with SpecDis 1.62.

The initial torsional sampling (MCMM) and OPLS_2005 force field conformational searches of (1*S*,4*S*,5*S*,9*R*,11*S*)-**1**, (1*S*,4*S*,5*S*,9*R*,11*S*,15*S*)-**3**, (1*S*,4*S*,5*S*,9*R*,11*S*,15*R*)-**3**, (1*S*,4*S*,9*R*,11*S*)-**6**, and (1*S*,4*S*,5*S*,9*R*,11*S*)-**8** afforded 18, 124, 118, 89, and 98 conformers within the 21 kJ/mol energy window, respectively. The Boltzmann populations of the conformers were obtained based on the potential energy provided by the OPLS_2005 force field, leading to 6, 14, 13, 6 and 10 conformers for (1*S*,4*S*,5*S*,9*R*,11*S*)-**1**, (1*S*,4*S*,5*S*,9*R*,11*S*,15*S*)-**3**, (1*S*,4*S*,5*S*,9*R*,11*S*,15*R*)-**3**, (1*S*,4*S*,9*R*,11*S*)-**6**, and (1*S*,4*S*,5*S*,9*R*,11*S*)-**8** above 1% population for further re-optimization. The resulting geometries were re-optimized at the MPW1PW91/6-31G(d) level with IEFPCM solvent model for CH_3CN, and frequency analysis was performed as well to confirm that the re-optimized geometries were at the energy minima. Finally, the Boltzmann-averaged ECD spectra of (1*S*,4*S*,5*S*,9*R*,11*S*)-**1**, (1*S*,4*S*,5*S*,9*R*,11*S*,15*S*)-**3**, (1*S*,4*S*,5*S*,9*R*,11*S*,15*R*)-**3**, (1*S*,4*S*,9*R*,11*S*)-**6**, (1*S*,4*S*,5*S*,9*R*,11*S*)-**8** and their enantiomers were matched to the experimental curves of sinuhirfuranone A (**1**), sinuhirfuranone C (**3**), sinuhirtin E (**6**) and sinuhirtone B (**8**), respectively.

2.2.1 ECD calculation for (1*S*,4*S*,5*S*,9*R*,11*S*)-1

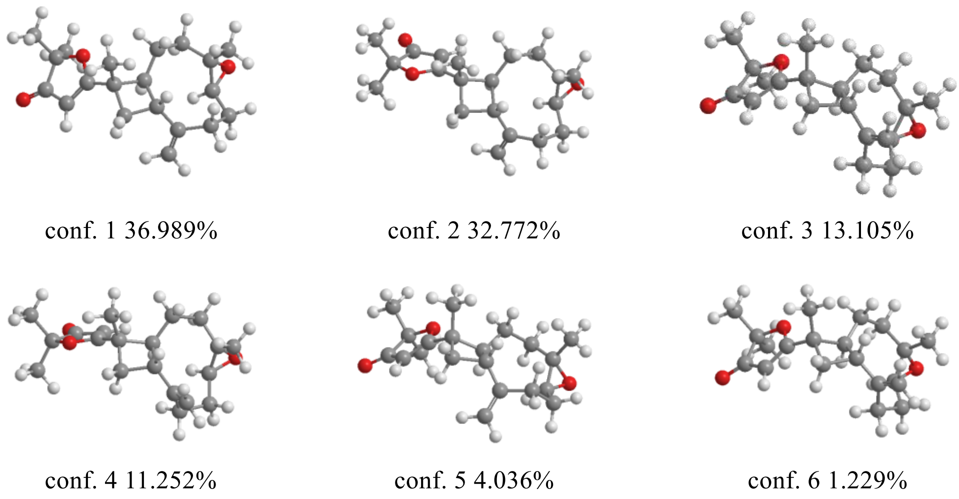

Figure S99. Re-optimized conformers above 1% population (OPLS_2005) of (1*S*,4*S*,5*S*,9*R*,11*S*)-**1** calculated at the MPW1PW91/6-31G(d) level with IEFPCM solvent model for acetonitrile.

Table S8. Cartesian coordinates for the re-optimized conformers of (1*S*,4*S*,5*S*,9*R*,11*S*)-**1** at the MPW1PW91/6-31G(d) level with IEFPCM solvent model for acetonitrile.

MPW1PW91/6-31G(d) Energy / Hartree =-1004.37956193 a.u. MPW1PW91/6-31G(d) Energy / Hartree =-1004.37904371 a.u.

Population = 36.989% Population = 32.772%

**1** Conf. 1		Standard Orientation (Ångstroms)			**1** Conf. 2		Standard Orientation (Ångstroms)		
I	atom	X	Y	Z	I	atom	X	Y	Z
1	C	0.826632	0.700812	1.110007	1	C	0.821397	0.164539	1.006731

2	C	0.132799	2.064506	0.93018	2	C	0.351046	1.641277	0.962972
3	H	-0.206714	0.018701	-0.680827	3	H	-0.37575	-0.20672	-0.76693
4	H	-1.698505	1.207135	1.702274	4	H	-1.57051	1.03147	1.750426
5	C	-3.697754	1.614237	0.012932	5	C	-3.52318	1.88696	0.175993
6	C	-2.230128	1.937141	-0.224515	6	C	-2.03046	1.971199	-0.1049
7	C	-4.280898	0.610338	-0.996583	7	C	-4.30154	1.04806	-0.85365
8	C	-3.335947	-0.547839	-1.158251	8	C	-3.56522	-0.23439	-1.12391
9	C	-3.129896	-1.585957	-0.142771	9	C	-3.49279	-1.3539	-0.17939
10	C	-1.7393	-2.167324	-0.031469	10	C	-2.2149	-2.16065	-0.17132
11	C	-0.799862	-1.362317	0.874292	11	C	-1.12123	-1.57984	0.732173
12	C	-0.406594	0.034518	0.397727	12	C	-0.52032	-0.23661	0.319685
13	C	-1.21634	1.343237	0.727308	13	C	-1.10088	1.157005	0.768057
14	C	-1.886865	2.73959	-1.237286	14	C	-1.58693	2.763256	-1.08636
15	C	0.985829	0.321806	2.586188	15	C	0.978625	-0.33288	2.447927
16	C	2.118944	0.50014	0.40009	16	C	2.066673	-0.12346	0.241871
17	O	-3.82268	-1.874917	-1.375839	17	O	-4.27213	-1.44864	-1.39084
18	C	-3.995548	-1.699732	1.087463	18	C	-4.31602	-1.40251	1.083682
19	H	-2.467122	-0.314313	-1.774594	19	H	-2.69476	-0.10616	-1.7683
20	O	2.537891	-0.775976	0.342124	20	O	3.166033	0.49923	0.699653
21	C	3.795668	-0.816798	-0.381817	21	C	4.292198	0.119882	-0.13377
22	C	4.063271	0.652491	-0.73771	22	C	3.679691	-0.83196	-1.17053
23	C	2.949144	1.391731	-0.204742	23	C	2.280141	-0.91464	-0.84387
24	C	4.865918	-1.362504	0.547515	24	C	4.846782	1.370377	-0.7937
25	O	5.052143	1.03032	-1.353749	25	O	4.326408	-1.36991	-2.06058
26	C	3.602264	-1.658817	-1.631347	26	C	5.309987	-0.59961	0.734856
27	H	0.467296	2.566537	0.020787	27	H	0.740316	2.158176	0.083779
28	H	0.198462	2.766232	1.76571	28	H	0.558606	2.247246	1.8486
29	H	-4.286492	2.536624	-0.026546	29	H	-3.9483	2.895706	0.2012
30	H	-3.820638	1.207091	1.021597	30	H	-3.68132	1.456727	1.170179
31	H	-4.426786	1.098154	-1.966703	31	H	-4.40779	1.608858	-1.78889
32	H	-5.263047	0.266851	-0.659956	32	H	-5.31124	0.845655	-0.48544
33	H	-1.311288	-2.232254	-1.038465	33	H	-1.84392	-2.22693	-1.20045
34	H	-1.802853	-3.193482	0.352122	34	H	-2.43305	-3.18572	0.153726
35	H	-1.212806	-1.307172	1.888091	35	H	-1.4812	-1.52312	1.765959
36	H	0.128583	-1.938641	0.962367	36	H	-0.30067	-2.30824	0.74775
37	H	-0.858816	3.002215	-1.463491	37	H	-0.53672	2.858565	-1.34133
38	H	-2.641625	3.166408	-1.892699	38	H	-2.27701	3.351779	-1.6853
39	H	1.308939	-0.714696	2.706815	39	H	1.122731	-1.41604	2.489881
40	H	1.732655	0.969509	3.055542	40	H	1.844654	0.145108	2.912612
41	H	0.043857	0.453535	3.125037	41	H	0.096058	-0.08241	3.042206
42	H	-4.973639	-1.239527	0.939272	42	H	-5.21058	-0.78273	1.006985
43	H	-4.152679	-2.756729	1.327459	43	H	-4.63396	-2.43317	1.273945
44	H	-3.521955	-1.231216	1.954797	44	H	-3.73916	-1.06954	1.951018
45	H	2.804967	2.459698	-0.26882	45	H	1.539423	-1.50128	-1.36615
46	H	5.834084	-1.316095	0.043135	46	H	5.63142	1.083086	-1.49792
47	H	4.923089	-0.768899	1.46356	47	H	4.063607	1.898578	-1.34364
48	H	4.653104	-2.401512	0.811365	48	H	5.270974	2.044883	-0.04574

49	H	4.514426	-1.62237	-2.231947	49	H	6.115754	-0.97854	0.101367
50	H	3.393897	-2.698423	-1.36619	50	H	5.732856	0.08169	1.477452
51	H	2.77475	-1.27434	-2.23325	51	H	4.848619	-1.445	1.251893

MPW1PW91/6-31G(d) Energy / Hartree =-1004.38048828 a.u. MPW1PW91/6-31G(d) Energy / Hartree =-1004.38008758 a.u.

Population = 13.105% Population = 11.252%

**1** Conf. 3		Standard Orientation (Ångstroms)			**1** Conf. 4		Standard Orientation (Ångstroms)		
I	atom	X	Y	Z	I	atom	X	Y	Z
1	C	0.74223	1.180421	0.831235	1	C	0.769632	0.775291	0.87834
2	C	-0.05367	2.2871	0.106103	2	C	0.228408	1.948612	0.014681
3	H	-0.35813	-0.22987	-0.36507	3	H	-0.51731	-0.5026	-0.26679
4	H	-1.68634	1.843194	1.444925	4	H	-1.51039	1.931273	1.286436
5	C	-2.53235	1.032134	-1.85731	5	C	-2.35859	0.989854	-1.98022
6	C	-2.53704	1.672308	-0.4833	6	C	-2.2965	1.743755	-0.66668
7	C	-3.33508	-0.28281	-1.91951	7	C	-3.39582	-0.15056	-1.97593
8	C	-2.6407	-1.33917	-1.10973	8	C	-2.94771	-1.23638	-1.04134
9	C	-2.85662	-1.60413	0.32086	9	C	-3.26349	-1.32131	0.392666
10	C	-1.63852	-2.0206	1.115175	10	C	-2.17843	-1.88333	1.283405
11	C	-0.84229	-0.82185	1.657349	11	C	-1.18909	-0.81071	1.768079
12	C	-0.49817	0.240484	0.613637	12	C	-0.60372	0.069716	0.662266
13	C	-1.36074	1.527966	0.447573	13	C	-1.20821	1.47071	0.339747
14	C	-3.58032	2.419902	-0.10649	14	C	-3.19415	2.702719	-0.41363
15	C	1.03036	1.520396	2.296439	15	C	1.054102	1.207227	2.320351
16	C	1.987072	0.699617	0.172807	16	C	1.963629	0.082799	0.318532
17	O	-3.30542	-2.53225	-0.68987	17	O	-3.8423	-2.24045	-0.55896
18	C	-3.982	-0.99388	1.116531	18	C	-4.2835	-0.44372	1.072191
19	H	-1.63394	-1.54731	-1.47951	19	H	-1.98502	-1.6615	-1.33505
20	O	2.472063	-0.45579	0.6589	20	O	3.066067	0.845723	0.226871
21	C	3.668946	-0.80269	-0.08648	21	C	4.137408	0.04325	-0.33465
22	C	3.82337	0.347135	-1.09206	22	C	3.490277	-1.33031	-0.5596
23	C	2.714035	1.230838	-0.84653	23	C	2.126443	-1.19486	-0.11899
24	C	4.840115	-0.84887	0.879444	24	C	4.568754	0.670566	-1.64911
25	O	4.73738	0.417141	-1.90425	25	O	4.088399	-2.29266	-1.02427
26	C	3.425695	-2.12348	-0.79645	26	C	5.26016	-0.03992	0.685121
27	H	0.137658	2.283199	-0.97087	27	H	0.470647	1.81223	-1.04355
28	H	0.044212	3.307846	0.484511	28	H	0.503141	2.961827	0.31842
29	H	-1.50584	0.842393	-2.19443	29	H	-1.37505	0.579877	-2.24091
30	H	-2.96648	1.730781	-2.57893	30	H	-2.62157	1.687112	-2.78142
31	H	-3.40604	-0.61321	-2.96268	31	H	-4.37659	0.240556	-1.69287
32	H	-4.35468	-0.11304	-1.56342	32	H	-3.48843	-0.55909	-2.98928
33	H	-0.99399	-2.61897	0.461282	33	H	-1.63605	-2.64893	0.716998
34	H	-1.93333	-2.6677	1.950287	34	H	-2.62254	-2.3853	2.151706
35	H	-1.38282	-0.35572	2.489397	35	H	-1.66376	-0.17719	2.526166
36	H	0.089079	-1.21213	2.082263	36	H	-0.37064	-1.32844	2.28231
37	H	-3.63365	2.874638	0.878972	37	H	-3.20146	3.24804	0.526217
38	H	-4.4203	2.603404	-0.77167	38	H	-3.9559	2.976174	-1.13932

39	H	1.426558	0.656774	2.835579	39	H	1.247004	0.344455	2.964231
40	H	1.76688	2.327401	2.356662	40	H	1.931142	1.858507	2.351774
41	H	0.123	1.855303	2.805401	41	H	0.207492	1.759229	2.736498
42	H	-4.80984	-0.68652	0.476809	42	H	-5.02013	-0.05898	0.366091
43	H	-4.35845	-1.73648	1.828717	43	H	-4.81299	-1.03366	1.828528
44	H	-3.64647	-0.12222	1.683025	44	H	-3.81573	0.406359	1.574054
45	H	2.502614	2.149997	-1.37178	45	H	1.375112	-1.97005	-0.13139
46	H	5.760799	-1.01631	0.315185	46	H	5.30861	0.024033	-2.12727
47	H	4.932537	0.095757	1.421624	47	H	3.715999	0.780434	-2.32402
48	H	4.713659	-1.66025	1.600684	48	H	5.014504	1.653678	-1.47823
49	H	4.282869	-2.34866	-1.43567	49	H	6.032468	-0.71607	0.310473
50	H	3.30081	-2.93266	-0.07257	50	H	5.702079	0.945587	0.852295
51	H	2.531441	-2.06741	-1.42266	51	H	4.890457	-0.42887	1.637391

MPW1PW91/6-31G(d) Energy / Hartree =-1004.37956191 a.u. MPW1PW91/6-31G(d) Energy / Hartree =-1004.38048837 a.u.

Population = 4.036% Population = 1.229%

**1** Conf. 5		Standard Orientation (Ångstroms)			**1** Conf. 6		Standard Orientation (Ångstroms)		
I	atom	X	Y	Z	I	atom	X	Y	Z
1	C	0.826632	0.70027	1.110176	1	C	-0.74224	1.180558	-0.8311
2	C	0.132723	2.064024	0.931006	2	C	0.053692	2.287158	-0.10585
3	H	-0.2067	0.018695	-0.68093	3	H	0.358163	-0.22977	0.365063
4	H	-1.69864	1.206264	1.702489	4	H	1.686235	1.843426	-1.44484
5	C	-3.69772	1.61446	0.01319	5	C	2.532496	1.032103	1.85728
6	C	-2.23001	1.937159	-0.22405	6	C	2.537164	1.67224	0.483247
7	C	-4.28085	0.610776	-0.99654	7	C	3.335114	-0.28291	1.919475
8	C	-3.33601	-0.54746	-1.15842	8	C	2.640634	-1.33921	1.10971
9	C	-3.13007	-1.58578	-0.14309	9	C	2.856534	-1.60417	-0.32089
10	C	-1.73959	-2.16744	-0.03198	10	C	1.6384	-2.02052	-1.11523
11	C	-0.79995	-1.3627	0.873836	11	C	0.842286	-0.82168	-1.65739
12	C	-0.4066	0.03422	0.397619	12	C	0.498172	0.240615	-0.61363
13	C	-1.21638	1.342802	0.727637	13	C	1.360738	1.528078	-0.44749
14	C	-1.88658	2.739787	-1.23663	14	C	3.580579	2.419561	0.106272
15	C	0.985886	0.320588	2.586162	15	C	-1.03042	1.520665	-2.29626
16	C	2.118884	0.499941	0.400086	16	C	-1.98706	0.699688	-0.17268
17	O	-3.82298	-1.87444	-1.37619	17	O	3.305249	-2.53234	0.689832
18	C	-3.99573	-1.69965	1.087122	18	C	3.981961	-0.99403	-1.11656
19	H	-2.46716	-0.31399	-1.77475	19	H	1.633857	-1.54726	1.47949
20	O	2.538149	-0.77606	0.342137	20	O	-2.47231	-0.45546	-0.65919
21	C	3.795957	-0.81655	-0.38184	21	C	-3.66905	-0.80255	0.086253
22	C	4.062987	0.652788	-0.73804	22	C	-3.82325	0.346901	1.092276
23	C	2.948732	1.391742	-0.20492	23	C	-2.71382	1.230561	0.846977
24	C	4.866428	-1.36146	0.547721	24	C	-4.84039	-0.84851	-0.87946
25	O	5.051613	1.030847	-1.35432	25	O	-4.73713	0.4167	1.904626
26	C	3.602944	-1.65909	-1.63104	26	C	-3.42567	-2.12354	0.795861
27	H	0.467225	2.566509	0.02187	27	H	-0.1376	2.283095	0.971128
28	H	0.198274	2.765309	1.766909	28	H	-0.04422	3.307957	-0.48411

29	H	-4.28632	2.536931	-0.02628	29	H	1.505998	0.84247	2.194463
30	H	-3.8208	1.207211	1.021788	30	H	2.966743	1.73073	2.578856
31	H	-5.26304	0.267257	-0.66008	31	H	3.406094	-0.61332	2.962636
32	H	-4.42663	1.098811	-1.96656	32	H	4.354714	-0.11322	1.563344
33	H	-1.31169	-2.23235	-1.03901	33	H	0.993795	-2.61885	-0.46137
34	H	-1.80326	-3.19361	0.351566	34	H	1.933171	-2.66762	-1.95036
35	H	-1.2128	-1.30778	1.887681	35	H	1.382923	-0.35553	-2.48936
36	H	0.128439	-1.93915	0.961652	36	H	-0.08906	-1.21187	-2.08243
37	H	-0.85849	3.002284	-1.46276	37	H	3.633881	2.874235	-0.87922
38	H	-2.64127	3.166889	-1.89194	38	H	4.420708	2.602861	0.771318
39	H	1.309087	-0.71594	2.706344	39	H	-1.42664	0.657084	-2.83545
40	H	1.732673	0.968139	3.055792	40	H	-1.76694	2.327674	-2.3564
41	H	0.043927	0.45202	3.125104	41	H	-0.12307	1.855594	-2.80523
42	H	-4.9738	-1.23939	0.938926	42	H	4.809775	-0.68659	-0.47684
43	H	-4.15293	-2.75667	1.326975	43	H	4.358442	-1.73672	-1.82864
44	H	-3.52221	-1.23127	1.954562	44	H	3.64648	-0.12242	-1.68317
45	H	2.804322	2.459679	-0.26896	45	H	-2.50224	2.149477	1.372592
46	H	5.834586	-1.31489	0.043347	46	H	-5.76096	-1.01611	-0.31506
47	H	4.923352	-0.76747	1.463529	47	H	-4.93294	0.096253	-1.42138
48	H	4.654005	-2.40045	0.811983	48	H	-4.71406	-1.6597	-1.60093
49	H	4.515106	-1.62245	-2.23163	49	H	-4.28275	-2.34889	1.435158
50	H	3.395049	-2.69869	-1.36551	50	H	-3.30088	-2.93254	0.071766
51	H	2.775276	-1.27523	-2.23312	51	H	-2.53132	-2.06762	1.421946

2.2.2 ECD calculation for (1*S*,4*S*,5*S*,9*R*,11*S*,15*S*)-3

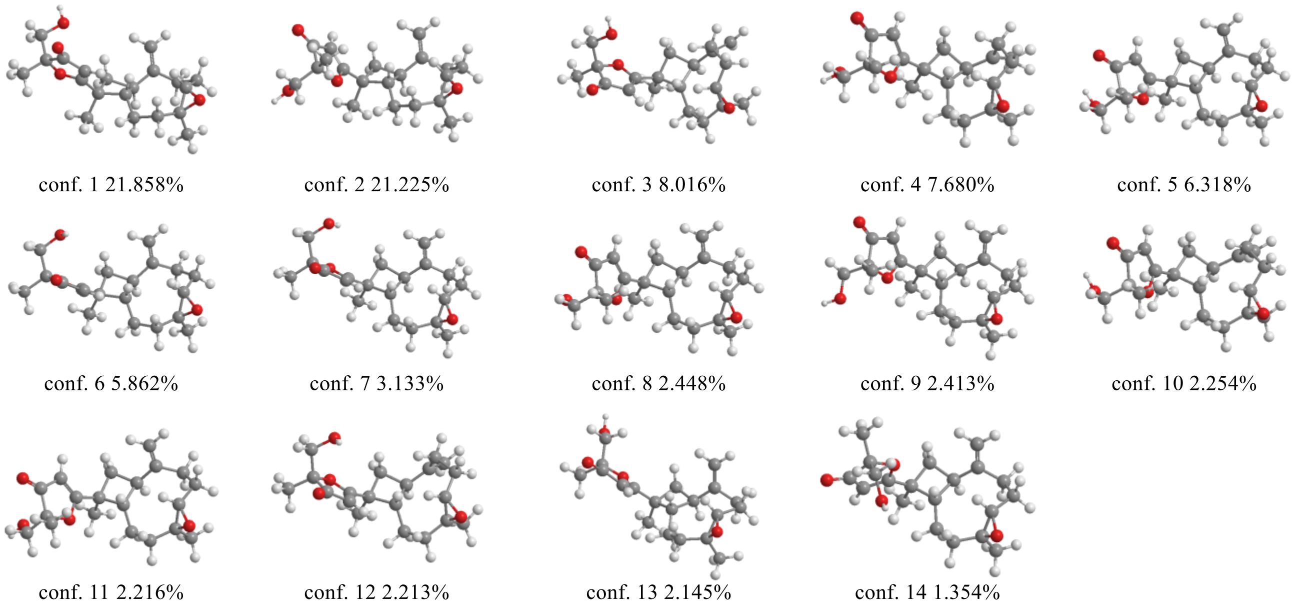

Figure S100. Re-optimized conformers above 1% population (OPLS_2005) of (1*S*,4*S*,5*S*,9*R*,11*S*,15*S*)-**3** calculated at the MPW1PW91/6-31G(d) level with IEFPCM solvent model for acetonitrile.

Table S9. Cartesian coordinates for the re-optimized conformers of (1*S*,4*S*,5*S*,9*R*,11*S*,15*S*)-**3** at the MPW1PW91/6-31G(d) level with IEFPCM solvent model for acetonitrile.

MPW1PW91/6-31G(d) Energy / Hartree =-1080.08961929 a.u. MPW1PW91/6-31G(d) Energy / Hartree =-1080.08982902 a.u.

Population = 21.858%

Population = 21.225%

**3** Conf. 1		Standard Orientation (Ångstroms)			**3** Conf. 2		Standard Orientation (Ångstroms)		
I	atom	X	Y	Z	I	atom	X	Y	Z
1	C	0.628738	-0.23131	1.119018	1	C	0.642149	0.688028	1.041437
2	C	0.244204	1.259611	1.371403	2	C	-0.07872	2.056987	0.966369
3	H	-0.54419	-0.19019	-0.71689	3	H	-0.48314	0.046469	-0.7167
4	H	-1.75786	0.656126	1.952242	4	H	-1.88551	1.153565	1.762259
5	C	-3.57999	1.9252	0.492052	5	C	-3.9514	1.590276	0.143963
6	C	-2.06492	1.968015	0.302383	6	C	-2.49213	1.963847	-0.11006
7	C	-4.35584	1.337686	-0.71204	7	C	-4.54536	0.607917	-0.89349
8	C	-3.69365	0.069991	-1.18701	8	C	-3.59571	-0.5407	-1.11865
9	C	-3.74962	-1.21289	-0.47108	9	C	-3.3605	-1.62108	-0.14933
10	C	-2.52159	-2.09785	-0.55395	10	C	-1.95602	-2.18792	-0.08369
11	C	-1.43469	-1.7586	0.485285	11	C	-1.00055	-1.38681	0.822817
12	C	-0.72378	-0.40533	0.340299	12	C	-0.6304	0.031005	0.367998
13	C	-1.23217	0.928925	1.032269	13	C	-1.43732	1.332113	0.779975
14	C	-1.52116	2.921853	-0.45924	14	C	-2.19565	2.847133	-1.06806
15	C	0.732888	-1.02224	2.435779	15	C	0.904484	0.245323	2.493083
16	C	1.875708	-0.41093	0.317266	16	C	1.897658	0.540476	0.249637
17	O	-4.46765	-1.02228	-1.72366	17	O	-4.08054	-1.87184	-1.38999
18	C	-4.65696	-1.44635	0.717012	18	C	-4.20768	-1.80248	1.091269
19	H	-2.7844	0.246403	-1.75854	19	H	-2.74413	-0.27965	-1.74412
20	O	2.998865	0.044019	0.912284	20	O	2.325412	-0.73107	0.096424
21	C	4.121827	-0.15677	-0.00123	21	C	3.573079	-0.71182	-0.66449
22	C	3.47423	-0.82656	-1.2332	22	C	3.809168	0.789863	-0.93922
23	C	2.073323	-0.94434	-0.91829	23	C	2.6932	1.4742	-0.33823
24	C	4.7132	1.206535	-0.3349	24	C	4.668921	-1.31268	0.205808
25	O	4.103376	-1.16499	-2.22585	25	O	4.77204	1.218299	-1.55985
26	C	5.139522	-1.074	0.664949	26	C	3.375965	-1.49529	-1.95613
27	O	3.719522	1.997876	-0.97564	27	O	4.800431	-0.53264	1.387674
28	H	0.709611	1.919539	0.639867	28	H	0.21235	2.618443	0.079669
29	H	0.44341	1.646523	2.372354	29	H	0.013654	2.70485	1.839929
30	H	-3.94861	2.937176	0.681144	30	H	-4.56025	2.498587	0.150485
31	H	-3.81421	1.335401	1.382189	31	H	-4.04124	1.145569	1.138445
32	H	-5.39378	1.149713	-0.42854	32	H	-4.71448	1.128049	-1.84157
33	H	-4.3728	2.063533	-1.53119	33	H	-5.51657	0.245898	-0.54884
34	H	-2.10455	-2.01317	-1.56239	34	H	-1.55171	-2.22528	-1.10027
35	H	-2.81906	-3.14445	-0.41996	35	H	-1.99781	-3.22135	0.279799
36	H	-1.84363	-1.85636	1.495824	36	H	-1.39275	-1.35902	1.844367
37	H	-0.66793	-2.53691	0.403073	37	H	-0.06699	-1.95579	0.877178
38	H	-0.45489	3.001061	-0.63242	38	H	-1.18193	3.152494	-1.29699
39	H	-2.14137	3.666545	-0.94841	39	H	-2.9776	3.300266	-1.66965
40	H	0.833925	-2.09536	2.257758	40	H	1.252528	-0.78723	2.543104
41	H	1.60578	-0.68591	2.998478	41	H	1.672604	0.883923	2.937484
42	H	-0.15164	-0.85969	3.054678	42	H	0.000139	0.337614	3.097499
43	H	-5.48888	-0.74293	0.738334	43	H	-5.18298	-1.32706	0.991436

44	H	-5.07058	-2.45816	0.660841	44	H	-4.36832	-2.87113	1.264929
45	H	-4.11073	-1.36421	1.659622	45	H	-3.71269	-1.39402	1.975595
46	H	1.318964	-1.36638	-1.56221	46	H	2.535155	2.540569	-0.33835
47	H	5.062153	1.677167	0.591832	47	H	4.405881	-2.35109	0.440115
48	H	5.575843	1.039248	-0.99136	48	H	5.594124	-1.30806	-0.38351
49	H	5.927053	-1.31895	-0.05022	49	H	4.26529	-1.39132	-2.58069
50	H	5.58648	-0.59218	1.53699	50	H	3.208574	-2.55396	-1.74776
51	H	4.661027	-2.00191	0.983188	51	H	2.518825	-1.10652	-2.50876
52	H	4.096175	2.867423	-1.1441	52	H	5.481009	-0.93725	1.934791

MPW1PW91/6-31G(d) Energy / Hartree =-1080.08994987 a.u. MPW1PW91/6-31G(d) Energy / Hartree =-1080.09015224 a.u.

Population = 8.016% Population = 7.680%

**3** Conf. 3		Standard Orientation (Ångstroms)			**3** Conf. 4		Standard Orientation (Ångstroms)		
I	atom	X	Y	Z	I	atom	X	Y	Z
1	C	0.585007	0.338373	1.262857	1	C	0.574835	1.157425	0.716853
2	C	0.129547	1.761516	0.801337	2	C	-0.23803	2.285355	0.024685
3	H	-0.66691	-0.45996	-0.29213	3	H	-0.61127	-0.25553	-0.4034
4	H	-1.71181	1.419724	1.878615	4	H	-1.83336	1.871864	1.424106
5	C	-2.34219	1.588499	-1.56981	5	C	-2.84663	1.031396	-1.82869
6	C	-2.34993	1.900393	-0.08016	6	C	-2.76444	1.708586	-0.46801
7	C	-3.39969	0.535503	-1.99144	7	C	-3.67884	-0.27745	-1.82451
8	C	-3.043	-0.80977	-1.41904	8	C	-2.96567	-1.33727	-1.02849
9	C	-3.47804	-1.33857	-0.11342	9	C	-3.1233	-1.58576	0.416216
10	C	-2.47006	-2.18928	0.637	10	C	-1.87296	-2.00627	1.166045
11	C	-1.49689	-1.35207	1.498942	11	C	-1.02501	-0.8065	1.648648
12	C	-0.80065	-0.19904	0.758923	12	C	-0.70039	0.234781	0.568338
13	C	-1.34618	1.268385	0.859443	13	C	-1.55038	1.544156	0.420183
14	C	-3.22521	2.787935	0.403939	14	C	-3.76023	2.506148	-0.06745
15	C	0.803233	0.261361	2.783837	15	C	0.953148	1.500844	2.169558
16	C	1.789473	-0.18031	0.549319	16	C	1.784483	0.658187	0.001631
17	O	-3.98928	-1.89471	-1.35862	17	O	-3.63028	-2.53049	-0.56974
18	C	-4.55245	-0.69136	0.731231	18	C	-4.21523	-0.95819	1.252796
19	H	-2.06655	-1.15474	-1.75976	19	H	-1.98022	-1.56396	-1.43571
20	O	2.908999	0.55216	0.727586	20	O	2.236045	-0.54344	0.418936
21	C	3.984044	-0.04411	-0.06323	21	C	3.437759	-0.87603	-0.34435
22	C	3.315073	-1.27105	-0.72099	22	C	3.619261	0.335503	-1.28547
23	C	1.949712	-1.26076	-0.26143	23	C	2.519676	1.219738	-0.99529
24	C	4.437726	0.97896	-1.09667	24	C	4.596508	-1.01763	0.633388
25	O	3.905266	-2.04523	-1.46125	25	O	4.53538	0.432137	-2.08993
26	C	5.115799	-0.45201	0.87105	26	C	3.186605	-2.15996	-1.12479
27	O	3.341568	1.295822	-1.94597	27	O	4.771482	0.215111	1.320743
28	H	0.453145	1.972354	-0.22021	28	H	-0.09056	2.288272	-1.05722
29	H	0.39879	2.600974	1.444652	29	H	-0.11044	3.299515	0.407585
30	H	-1.35133	1.248279	-1.88843	30	H	-1.84482	0.815859	-2.2149
31	H	-2.54277	2.508673	-2.12398	31	H	-3.30726	1.722157	-2.53911
32	H	-4.39205	0.858033	-1.67045	32	H	-4.67685	-0.08208	-1.42729

33	H	-3.41816	0.467473	-3.08488	33	H	-3.80183	-0.62443	-2.85653
34	H	-1.90115	-2.76878	-0.09675	34	H	-1.27123	-2.63131	0.499115
35	H	-2.98969	-2.90928	1.279177	35	H	-2.14195	-2.62643	2.028609
36	H	-2.02124	-0.95854	2.375771	36	H	-1.52025	-0.31508	2.492306
37	H	-0.7373	-2.03779	1.888156	37	H	-0.08708	-1.20981	2.041376
38	H	-3.27888	3.017388	1.463353	38	H	-3.75134	2.985501	0.906209
39	H	-3.91858	3.315366	-0.24404	39	H	-4.61916	2.705762	-0.70086
40	H	0.938188	-0.77146	3.114229	40	H	1.368769	0.634108	2.686018
41	H	1.69418	0.827759	3.062156	41	H	1.704442	2.294805	2.18095
42	H	-0.0483	0.682188	3.321949	42	H	0.08295	1.850997	2.727777
43	H	-5.21029	-0.05918	0.136521	43	H	-5.06295	-0.64815	0.643544
44	H	-5.15971	-1.47788	1.191042	44	H	-4.56963	-1.69482	1.981202
45	H	-4.12532	-0.08517	1.532107	45	H	-3.8516	-0.08819	1.802409
46	H	1.191263	-1.97712	-0.53335	46	H	2.330443	2.168872	-1.47039
47	H	4.809118	1.868761	-0.57459	47	H	4.375667	-1.83559	1.329532
48	H	5.264299	0.530195	-1.66095	48	H	5.486889	-1.28194	0.049951
49	H	5.873769	-0.99554	0.303774	49	H	4.035672	-2.3546	-1.78283
50	H	5.57569	0.424898	1.331418	50	H	3.057506	-3.00777	-0.44895
51	H	4.739455	-1.10595	1.65961	51	H	2.289792	-2.06069	-1.73886
52	H	3.637704	1.959371	-2.5771	52	H	5.511642	0.113675	1.927514

MPW1PW91/6-31G(d) Energy / Hartree =-1080.09084922 a.u. MPW1PW91/6-31G(d) Energy / Hartree =-1080.09060698 a.u.

Population = 6.318% Population = 5.862%

**3** Conf. 5		Standard Orientation (Ångstroms)			**3** Conf. 6		Standard Orientation (Ångstroms)		
I	atom	X	Y	Z	I	atom	X	Y	Z
1	C	0.640738	0.684776	1.038026	1	C	0.627449	-0.22472	1.118749
2	C	-0.07603	2.055517	0.956558	2	C	0.243701	1.267932	1.364241
3	H	-0.48702	0.0383	-0.71741	3	H	-0.54697	-0.18553	-0.71613
4	H	-1.88633	1.161993	1.755698	4	H	-1.75435	0.662415	1.955198
5	C	-3.94965	1.600106	0.135357	5	C	-3.5868	1.9186	0.494444
6	C	-2.4887	1.965185	-0.12127	6	C	-2.07202	1.970088	0.30452
7	C	-4.54821	0.613869	-0.89578	7	C	-4.36029	1.331228	-0.71112
8	C	-3.60284	-0.53934	-1.11532	8	C	-3.6945	0.06581	-1.18686
9	C	-3.37074	-1.61548	-0.1405	9	C	-3.74819	-1.21783	-0.47217
10	C	-1.96832	-2.18732	-0.07312	10	C	-2.51741	-2.09903	-0.55341
11	C	-1.00906	-1.3858	0.828991	11	C	-1.43306	-1.75556	0.487035
12	C	-0.63439	0.028526	0.36733	12	C	-0.72522	-0.40076	0.341276
13	C	-1.43687	1.334278	0.77293	13	C	-1.23349	0.934207	1.032152
14	C	-2.18802	2.840195	-1.08553	14	C	-1.53368	2.929574	-0.45383
15	C	0.902281	0.247867	2.491412	15	C	0.730337	-1.00966	2.439206
16	C	1.893702	0.52917	0.244261	16	C	1.874453	-0.4082	0.318471
17	O	-4.0926	-1.87017	-1.37921	17	O	-4.46467	-1.02806	-1.7257
18	C	-4.21759	-1.78735	1.101705	18	C	-4.65663	-1.45465	0.714396
19	H	-2.7508	-0.28474	-1.74282	19	H	-2.78486	0.245191	-1.75688
20	O	2.328598	-0.74085	0.110348	20	O	3.001963	0.019916	0.922125
21	C	3.571964	-0.72711	-0.65647	21	C	4.122732	-0.17673	0.006362

22	C	3.798074	0.768384	-0.95539	22	C	3.46869	-0.80934	-1.23803
23	C	2.681353	1.457742	-0.36512	23	C	2.067704	-0.9211	-0.92796
24	C	4.6801	-1.30114	0.233915	24	C	4.725373	1.198413	-0.30347
25	O	4.773546	1.192397	-1.56265	25	O	4.093302	-1.10586	-2.24889
26	C	3.382521	-1.54252	-1.92875	26	C	5.136065	-1.11104	0.654107
27	O	4.866351	-0.57705	1.441365	27	O	3.802335	2.101286	-0.89605
28	H	0.216364	2.612724	0.067687	28	H	0.706183	1.923204	0.626539
29	H	0.018333	2.706596	1.827481	29	H	0.447108	1.660696	2.361998
30	H	-4.55424	2.511275	0.13616	30	H	-3.96042	2.928066	0.687094
31	H	-4.04128	1.162126	1.132633	31	H	-3.81774	1.324679	1.382707
32	H	-4.71645	1.129062	-1.84671	32	H	-5.39796	1.140382	-0.42856
33	H	-5.52033	0.257365	-0.54801	33	H	-4.37846	2.058193	-1.52925
34	H	-1.56486	-2.23113	-1.08977	34	H	-2.09941	-2.01351	-1.56138
35	H	-2.01381	-3.21882	0.29533	35	H	-2.8116	-3.14652	-0.41925
36	H	-1.40013	-1.35198	1.850766	36	H	-1.84309	-1.8532	1.497112
37	H	-0.07757	-1.95806	0.88499	37	H	-0.66394	-2.53179	0.406878
38	H	-1.17289	3.138973	-1.31692	38	H	-0.46782	3.01646	-0.62574
39	H	-2.96783	3.292759	-1.69031	39	H	-2.15816	3.672278	-0.94058
40	H	1.240378	-0.78762	2.547203	40	H	0.831997	-2.0835	2.266072
41	H	1.67734	0.881715	2.9306	41	H	1.60213	-0.67046	3.001685
42	H	0.000048	0.352501	3.09689	42	H	-0.1553	-0.84482	3.055744
43	H	-5.1918	-1.3102	0.999525	43	H	-5.49076	-0.75383	0.734868
44	H	-4.38076	-2.85453	1.281868	44	H	-5.06702	-2.46771	0.657163
45	H	-3.72116	-1.37484	1.983341	45	H	-4.11199	-1.37121	1.657811
46	H	2.519688	2.5234	-0.37982	46	H	1.309125	-1.31869	-1.58232
47	H	4.405275	-2.31858	0.518988	47	H	5.050002	1.654604	0.633724
48	H	5.599711	-1.34298	-0.3599	48	H	5.604596	1.044989	-0.93856
49	H	4.268623	-1.43898	-2.55816	49	H	5.917167	-1.35201	-0.06959
50	H	3.234746	-2.59891	-1.69434	50	H	5.59326	-0.64236	1.528272
51	H	2.517245	-1.18309	-2.48869	51	H	4.654311	-2.03976	0.965235
52	H	5.380504	0.213647	1.245411	52	H	3.719172	1.881964	-1.83043

MPW1PW91/6-31G(d) Energy / Hartree =-1080.09096903 a.u. MPW1PW91/6-31G(d) Energy / Hartree =-1080.09155350 a.u.

Population = 3.133% Population = 2.448%

**3** Conf. 7		Standard Orientation (Ångstroms)			**3** Conf. 8		Standard Orientation (Ångstroms)		
I	atom	X	Y	Z	I	atom	X	Y	Z
1	C	0.633234	-0.09878	1.084586	1	C	0.646187	0.688082	1.060036
2	C	0.230387	1.404173	1.219944	2	C	-0.0774	2.05564	0.984815
3	H	-0.56791	-0.20017	-0.7292	3	H	-0.47021	0.052403	-0.70754
4	H	-1.74069	0.808402	1.902167	4	H	-1.88644	1.14622	1.769289
5	C	-3.63321	1.911279	0.392669	5	C	-3.94544	1.586468	0.143307
6	C	-2.12349	1.990271	0.173351	6	C	-2.48558	1.963001	-0.1026
7	C	-4.41008	1.240565	-0.76536	7	C	-4.53342	0.607786	-0.90105
8	C	-3.72343	-0.03785	-1.17232	8	C	-3.58096	-0.53833	-1.12698
9	C	-3.74333	-1.27539	-0.37878	9	C	-3.34811	-1.62244	-0.16122
10	C	-2.49499	-2.13418	-0.41803	10	C	-1.94296	-2.18731	-0.0922

11	C	-1.40874	-1.70233	0.586596	11	C	-0.99257	-1.38927	0.822447
12	C	-0.72737	-0.34768	0.342682	12	C	-0.62344	0.0313	0.376154
13	C	-1.24738	1.023413	0.949435	13	C	-1.4339	1.329392	0.789906
14	C	-1.62378	2.916201	-0.65042	14	C	-2.18587	2.850402	-1.05573
15	C	0.75717	-0.77678	2.461104	15	C	0.895769	0.241319	2.511886
16	C	1.872427	-0.33562	0.284716	16	C	1.899871	0.542763	0.265042
17	O	-4.47585	-1.1787	-1.63354	17	O	-4.06223	-1.86917	-1.40594
18	C	-4.63358	-1.4529	0.831856	18	C	-4.20035	-1.81054	1.074891
19	H	-2.82363	0.125167	-1.76254	19	H	-2.72723	-0.27327	-1.74778
20	O	3.022892	0.047697	0.888727	20	O	2.369914	-0.72472	0.170117
21	C	4.139245	-0.2429	-0.01049	21	C	3.579635	-0.7103	-0.65383
22	C	3.454315	-0.82143	-1.26402	22	C	3.770054	0.781397	-0.9923
23	C	2.045856	-0.85927	-0.95777	23	C	2.652485	1.465339	-0.39059
24	C	4.86265	1.074844	-0.30202	24	C	4.727942	-1.25696	0.198599
25	O	4.06246	-1.16498	-2.26761	25	O	4.706172	1.2087	-1.65273
26	C	5.054064	-1.2674	0.64818	26	C	3.340152	-1.54617	-1.90413
27	O	4.001848	2.075307	-0.8241	27	O	4.918146	-0.52962	1.402186
28	H	0.657696	2.002573	0.415835	28	H	0.215301	2.62106	0.101438
29	H	0.452308	1.881495	2.175878	29	H	0.01053	2.700755	1.860802
30	H	-4.02971	2.919776	0.539125	30	H	-4.55543	2.493987	0.150952
31	H	-3.83388	1.358515	1.313993	31	H	-4.03927	1.137414	1.13547
32	H	-5.44003	1.046544	-0.45792	32	H	-4.69917	1.131759	-1.8476
33	H	-4.45281	1.917063	-1.62472	33	H	-5.50557	0.242904	-0.56216
34	H	-2.08886	-2.10429	-1.43396	34	H	-1.53422	-2.21905	-1.10719
35	H	-2.76515	-3.17691	-0.21432	35	H	-1.98454	-3.22252	0.266132
36	H	-1.80755	-1.73962	1.605204	36	H	-1.38881	-1.36742	1.842552
37	H	-0.6252	-2.46747	0.550236	37	H	-0.05836	-1.95733	0.877399
38	H	-0.56378	3.02211	-0.84626	38	H	-1.17162	3.157934	-1.27921
39	H	-2.27643	3.611271	-1.16958	39	H	-2.96571	3.304957	-1.65896
40	H	0.872206	-1.85936	2.370026	40	H	1.224297	-0.79754	2.565894
41	H	1.628539	-0.38454	2.987873	41	H	1.66942	0.869695	2.961907
42	H	-0.12568	-0.5775	3.071192	42	H	-0.01004	0.34716	3.111655
43	H	-5.49221	-0.7827	0.80539	43	H	-5.17595	-1.33624	0.972694
44	H	-5.00728	-2.48128	0.858056	44	H	-4.36002	-2.88018	1.243241
45	H	-4.08589	-1.27538	1.760538	45	H	-3.71005	-1.40505	1.963191
46	H	1.272845	-1.22655	-1.61274	46	H	2.465091	2.525553	-0.44098
47	H	5.36887	1.41674	0.608156	47	H	4.553749	-2.32134	0.395403
48	H	5.617827	0.875348	-1.06492	48	H	5.64805	-1.15683	-0.38055
49	H	5.846095	-1.54381	-0.05095	49	H	4.207006	-1.46426	-2.56322
50	H	5.506034	-0.85747	1.553629	50	H	3.188965	-2.59604	-1.64509
51	H	4.495954	-2.16816	0.91015	51	H	2.462042	-1.18687	-2.4439
52	H	3.437756	2.376104	-0.10358	52	H	4.169559	-0.7188	1.978271

MPW1PW91/6-31G(d) Energy / Hartree =-1080.08906484 a.u. MPW1PW91/6-31G(d) Energy / Hartree =-1080.09113041 a.u.

Population = 2.413% Population = 2.254%

**3**<br>Conf. 9	Standard Orientation<br>(Ångstroms)	**3**<br>Conf. 10	Standard Orientation<br>(Ångstroms)

I	atom	X	Y	Z	I	atom	X	Y	Z
1	C	-0.58398	-0.79257	1.142607	1	C	0.572932	1.154095	0.721289
2	C	0.210664	-2.12033	1.076363	2	C	-0.23668	2.283553	0.027734
3	H	0.448122	-0.13737	-0.66801	3	H	-0.61421	-0.25654	-0.4014
4	H	1.986584	-1.09554	1.791873	4	H	-1.83529	1.872818	1.424244
5	C	4.024029	-1.46104	0.119201	5	C	-2.84376	1.034579	-1.83072
6	C	2.580877	-1.92119	-0.07819	6	C	-2.76312	1.711297	-0.46971
7	C	4.529617	-0.47195	-0.95863	7	C	-3.67819	-0.27286	-1.82849
8	C	3.508254	0.613361	-1.1821	8	C	-2.96845	-1.33403	-1.03125
9	C	3.24173	1.705957	-0.23499	9	C	-3.12951	-1.58261	0.413094
10	C	1.809801	2.193752	-0.13918	10	C	-1.88145	-2.00544	1.165428
11	C	0.928891	1.36525	0.816876	11	C	-1.03233	-0.80725	1.649892
12	C	0.628086	-0.08321	0.410462	12	C	-0.70402	0.233607	0.570352
13	C	1.519667	-1.3259	0.829452	13	C	-1.55103	1.544708	0.420828
14	C	2.305363	-2.84489	-1.0038	14	C	-3.75831	2.51038	-0.07076
15	C	-0.82144	-0.32625	2.590618	15	C	0.949549	1.496433	2.174533
16	C	-1.86613	-0.73154	0.382079	16	C	1.781302	0.651551	0.006674
17	O	3.906219	1.961526	-1.50555	17	O	-3.63604	-2.52619	-0.57411
18	C	4.116617	1.970533	0.970829	18	C	-4.22212	-0.95335	1.247538
19	H	2.654081	0.285731	-1.77167	19	H	-1.98261	-1.56242	-1.43657
20	O	-2.37604	0.508782	0.22708	20	O	2.234323	-0.54751	0.425716
21	C	-3.61615	0.408116	-0.54522	21	C	3.432329	-0.88312	-0.34082
22	C	-3.76607	-1.10708	-0.79666	22	C	3.612966	0.325198	-1.28148
23	C	-2.61324	-1.71753	-0.18557	23	C	2.514522	1.209918	-0.9953
24	C	-4.76964	0.90946	0.315978	24	C	4.598723	-1.01765	0.64457
25	O	-4.70663	-1.60254	-1.40269	25	O	4.545576	0.430458	-2.06829
26	C	-3.44262	1.18314	-1.84544	26	C	3.185061	-2.17295	-1.11209
27	O	-4.63859	2.312181	0.507609	27	O	4.840755	0.161961	1.397419
28	H	-0.07415	-2.71968	0.212788	28	H	-0.08761	2.286472	-1.05393
29	H	0.181952	-2.75032	1.967324	29	H	-0.10777	3.297348	0.411082
30	H	4.682727	-2.33386	0.125639	30	H	-1.84155	0.817496	-2.21506
31	H	4.12051	-0.98898	1.100506	31	H	-3.30183	1.726302	-2.54184
32	H	5.48746	-0.04628	-0.65126	32	H	-4.67663	-0.0758	-1.43322
33	H	4.701795	-1.0049	-1.89901	33	H	-3.79975	-0.6194	-2.86082
34	H	1.374077	2.17983	-1.14319	34	H	-1.27946	-2.63152	0.49972
35	H	1.802865	3.23755	0.195783	35	H	-2.15325	-2.6251	2.027442
36	H	1.351322	1.387779	1.826426	36	H	-1.52822	-0.31503	2.492682
37	H	-0.03374	1.881788	0.884299	37	H	-0.09591	-1.21237	2.044343
38	H	1.303935	-3.21184	-1.1928	38	H	-3.75048	2.989431	0.903046
39	H	3.092927	-3.26974	-1.61855	39	H	-4.61572	2.711547	-0.70573
40	H	-1.22257	0.687459	2.627385	40	H	1.35681	0.627443	2.693756
41	H	-1.53581	-0.99454	3.079165	41	H	1.706677	2.284834	2.187472
42	H	0.107051	-0.35266	3.164063	42	H	0.080114	1.853791	2.729265
43	H	5.114851	1.551794	0.847113	43	H	-5.06786	-0.6414	0.636521
44	H	4.217374	3.051021	1.113428	44	H	-4.57954	-1.68965	1.974793
45	H	3.678049	1.555541	1.881509	45	H	-3.85812	-0.0843	1.798412
46	H	-2.39148	-2.77231	-0.1807	46	H	2.326428	2.159092	-1.4707

47	H	-5.69828	0.654872	-0.20926	47	H	4.356833	-1.80313	1.363221
48	H	-4.7573	0.37471	1.273703	48	H	5.483986	-1.32596	0.07772
49	H	-4.32136	1.024447	-2.47429	49	H	4.031558	-2.36435	-1.77456
50	H	-3.34029	2.24814	-1.64036	50	H	3.069833	-3.01759	-0.42943
51	H	-2.56126	0.830912	-2.38471	51	H	2.282785	-2.08748	-1.72032
52	H	-5.39508	2.610153	1.022972	52	H	5.333415	0.779339	0.845972

MPW1PW91/6-31G(d) Energy / Hartree =-1080.08982887 a.u. MPW1PW91/6-31G(d) Energy / Hartree =-1080.09089415 a.u.

Population = 2.216% Population = 2.213%

**3** Conf. 11		Standard Orientation (Ångstroms)			**3** Conf. 12		Standard Orientation (Ångstroms)		
I	atom	X	Y	Z	I	atom	X	Y	Z
1	C	0.642182	0.687682	1.0415	1	C	-0.58413	0.406992	-1.25837
2	C	-0.07868	2.05665	0.966961	2	C	-0.12594	1.803847	-0.72665
3	H	-0.48307	0.046592	-0.71683	3	H	0.659704	-0.46657	0.26152
4	H	-1.88565	1.153012	1.762304	4	H	1.715905	1.509021	-1.81726
5	C	-3.95129	1.590637	0.144168	5	C	2.335823	1.516992	1.637787
6	C	-2.49195	1.963793	-0.10993	6	C	2.351911	1.894761	0.163582
7	C	-4.54557	0.608323	-0.89319	7	C	3.387635	0.442429	2.016681
8	C	-3.59602	-0.54032	-1.11861	8	C	3.027233	-0.87451	1.384357
9	C	-3.36077	-1.62088	-0.1495	9	C	3.46643	-1.3482	0.058991
10	C	-1.95632	-2.18784	-0.08415	10	C	2.457662	-2.15984	-0.73245
11	C	-1.00059	-1.38705	0.822349	11	C	1.49391	-1.281	-1.56324
12	C	-0.63035	0.030855	0.36786	12	C	0.798573	-0.1589	-0.77626
13	C	-1.43728	1.33183	0.780153	13	C	1.348565	1.310312	-0.80678
14	C	-2.19523	2.846844	-1.06807	14	C	3.233508	2.798172	-0.27749
15	C	0.904581	0.244507	2.493026	15	C	-0.79908	0.40635	-2.78188
16	C	1.89772	0.540334	0.249682	16	C	-1.79236	-0.14557	-0.57803
17	O	-4.08099	-1.87137	-1.39009	17	O	3.967787	-1.96111	1.281511
18	C	-4.20782	-1.80247	1.091163	18	C	4.549356	-0.6732	-0.75239
19	H	-2.74451	-0.27925	-1.74415	19	H	2.046934	-1.22888	1.703723
20	O	2.325509	-0.73113	0.096295	20	O	-2.90079	0.614107	-0.68728
21	C	3.573234	-0.71173	-0.66455	21	C	-3.97775	-0.02621	0.063979
22	C	3.809192	0.790007	-0.93916	22	C	-3.32476	-1.31018	0.613411
23	C	2.693205	1.474203	-0.33807	23	C	-1.9645	-1.28727	0.14375
24	C	4.669057	-1.31252	0.205815	24	C	-4.39295	0.9247	1.193593
25	O	4.772002	1.218533	-1.55983	25	O	-3.91714	-2.11736	1.318659
26	C	3.376323	-1.49514	-1.95625	26	C	-5.133	-0.32863	-0.8805
27	O	4.800457	-0.53245	1.387681	27	O	-3.33529	1.237794	2.0885
28	H	0.212425	2.618655	0.080625	28	H	-0.44974	1.965304	0.303776
29	H	0.013574	2.704004	1.840908	29	H	-0.39228	2.674871	-1.32774
30	H	-4.55991	2.499104	0.150655	30	H	1.342223	1.166191	1.936068
31	H	-4.04122	1.146019	1.138681	31	H	2.53645	2.410704	2.233615
32	H	-5.51669	0.246326	-0.54827	32	H	4.382153	0.77418	1.712753
33	H	-4.71494	1.128464	-1.84121	33	H	3.402204	0.32657	3.106138
34	H	-1.55221	-2.22507	-1.10082	34	H	1.881362	-2.76621	-0.02681
35	H	-1.99818	-3.22133	0.279179	35	H	2.976736	-2.85506	-1.40178

36	H	-1.39262	-1.35944	1.843971	36	H	2.025274	-0.85208	-2.41899
37	H	-0.0671	-1.95615	0.876404	37	H	0.733673	-1.94528	-1.98678
38	H	-1.18143	3.151874	-1.29707	38	H	3.292156	3.07389	-1.3255
39	H	-2.97706	3.300098	-1.66973	39	H	3.926999	3.293053	0.395612
40	H	1.252643	-0.78805	2.542688	40	H	-0.9445	-0.6072	-3.1632
41	H	1.672698	0.883	2.937588	41	H	-1.68319	0.995585	-3.03376
42	H	0.000261	0.336606	3.097507	42	H	0.058403	0.844163	-3.29628
43	H	-5.18283	-1.32634	0.991882	43	H	5.207085	-0.06819	-0.12988
44	H	-4.36912	-2.87114	1.264184	44	H	5.155545	-1.44432	-1.23895
45	H	-3.71235	-1.3949	1.975636	45	H	4.130294	-0.03454	-1.53192
46	H	2.534965	2.540542	-0.33813	46	H	-1.21434	-2.03306	0.351855
47	H	4.406069	-2.35093	0.440108	47	H	-4.71693	1.868737	0.751339
48	H	5.59431	-1.30784	-0.38343	48	H	-5.24435	0.474325	1.715341
49	H	4.26573	-1.39101	-2.58067	49	H	-5.88992	-0.90919	-0.34942
50	H	3.209024	-2.55385	-1.74798	50	H	-5.58611	0.594791	-1.24752
51	H	2.519223	-1.10642	-2.50898	51	H	-4.78531	-0.91267	-1.73449
52	H	5.480366	-0.93753	1.93529	52	H	-3.22	0.498062	2.694798

MPW1PW91/6-31G(d) Energy / Hartree =-1080.08838676 a.u. MPW1PW91/6-31G(d) Energy / Hartree =-1080.09071889 a.u.

Population = 2.145% Population = 1.354%

**3** Conf. 13		Standard Orientation (Ångstroms)			**3** Conf. 14		Standard Orientation (Ångstroms)		
I	atom	X	Y	Z	I	atom	X	Y	Z
1	C	0.586746	0.006091	1.032725	1	C	0.688846	0.857927	1.342974
2	C	0.208301	1.518843	1.006328	2	C	0.114158	2.189764	0.77036
3	H	-0.64095	-0.29604	-0.74172	3	H	-0.22729	-0.15514	-0.3536
4	H	-1.77079	1.043034	1.758376	4	H	-1.85395	1.643777	1.506118
5	C	-3.64398	2.03124	0.158299	5	C	-3.64612	1.670908	-0.47649
6	C	-2.13825	2.018459	-0.09961	6	C	-2.14609	1.941879	-0.5784
7	C	-4.47309	1.229963	-0.87529	7	C	-4.13858	0.481141	-1.33493
8	C	-3.82589	-0.10624	-1.13357	8	C	-3.24513	-0.71198	-1.11667
9	C	-3.84352	-1.23262	-0.18857	9	C	-3.25134	-1.53502	0.101144
10	C	-2.61673	-2.12267	-0.15943	10	C	-1.91989	-2.10142	0.552015
11	C	-1.49303	-1.60653	0.759631	11	C	-1.08241	-1.11415	1.389426
12	C	-0.78816	-0.3073	0.34229	12	C	-0.5367	0.119073	0.657732
13	C	-1.2778	1.134825	0.786105	13	C	-1.27116	1.521258	0.587505
14	C	-1.62722	2.793359	-1.06094	14	C	-1.66286	2.572791	-1.65299
15	C	0.726895	-0.51851	2.474114	15	C	0.67005	0.838519	2.874096
16	C	1.811025	-0.34733	0.253681	16	C	2.011148	0.425194	0.80286
17	O	-4.61606	-1.27734	-1.42263	17	O	-3.76505	-2.05696	-1.1577
18	C	-4.69836	-1.23514	1.059975	18	C	-4.30722	-1.39331	1.175714
19	H	-2.94294	-0.04025	-1.76678	19	H	-2.28369	-0.62892	-1.62012
20	O	2.949717	0.227473	0.694261	20	O	2.153849	0.532302	-0.53645
21	C	4.0554	-0.2203	-0.1551	21	C	3.459393	-0.00192	-0.91194
22	C	3.372882	-1.14944	-1.18098	22	C	4.083046	-0.42162	0.436445
23	C	1.976291	-1.15778	-0.82731	23	C	3.097791	-0.09743	1.435737
24	C	4.650079	0.990534	-0.86516	24	C	3.238435	-1.19588	-1.83227

25	O	3.973785	-1.723	-2.07937	25	O	5.197297	-0.91516	0.54043
26	C	5.056542	-0.97343	0.711456	26	C	4.267163	1.098235	-1.5887
27	O	5.323307	1.810086	0.081914	27	O	2.481312	-2.18064	-1.1384
28	H	0.642311	2.02599	0.144958	28	H	0.583607	2.438272	-0.17937
29	H	0.439944	2.091724	1.905882	29	H	0.162625	3.055021	1.434379
30	H	-4.00038	3.064958	0.162759	30	H	-4.19466	2.567307	-0.77867
31	H	-3.84205	1.628287	1.155037	31	H	-3.90698	1.480684	0.567963
32	H	-5.49572	1.103019	-0.51252	32	H	-5.17349	0.242786	-1.07938
33	H	-4.53287	1.788103	-1.81502	33	H	-4.12425	0.75661	-2.39426
34	H	-2.23957	-2.22129	-1.18199	34	H	-1.35166	-2.40129	-0.33403
35	H	-2.90549	-3.12738	0.170893	35	H	-2.0903	-3.00851	1.143592
36	H	-1.86564	-1.51375	1.784592	36	H	-1.64256	-0.8076	2.278225
37	H	-0.72886	-2.39102	0.795178	37	H	-0.21721	-1.67396	1.762027
38	H	-0.56964	2.824638	-1.29355	38	H	-0.61133	2.797529	-1.78222
39	H	-2.26798	3.430475	-1.66265	39	H	-2.32146	2.88976	-2.45577
40	H	0.81494	-1.60697	2.502268	40	H	0.874149	-0.15538	3.278002
41	H	1.619643	-0.09136	2.934927	41	H	1.422738	1.526635	3.268005
42	H	-0.13593	-0.22951	3.077206	42	H	-0.30126	1.160266	3.253554
43	H	-5.53374	-0.54057	0.978688	43	H	-5.22067	-0.94078	0.791057
44	H	-5.10561	-2.23893	1.216327	44	H	-4.55878	-2.38401	1.566922
45	H	-4.11511	-0.97293	1.945748	45	H	-3.94699	-0.78864	2.01147
46	H	1.205694	-1.71491	-1.33506	46	H	3.203927	-0.26322	2.495134
47	H	5.334888	0.60919	-1.63254	47	H	2.718881	-0.85638	-2.73609
48	H	3.843848	1.541277	-1.36536	48	H	4.227027	-1.5772	-2.1157
49	H	5.830893	-1.40156	0.071218	49	H	5.282363	0.740938	-1.77149
50	H	5.521474	-0.29623	1.426977	50	H	3.813476	1.385419	-2.53945
51	H	4.561077	-1.78487	1.247915	51	H	4.321427	1.978296	-0.94539
52	H	5.723803	2.542823	-0.39707	52	H	2.341755	-2.92241	-1.73561

2.2.3 ECD calculation for (1*S*,4*S*,5*S*,9*R*,11*S*,15*R*)-3

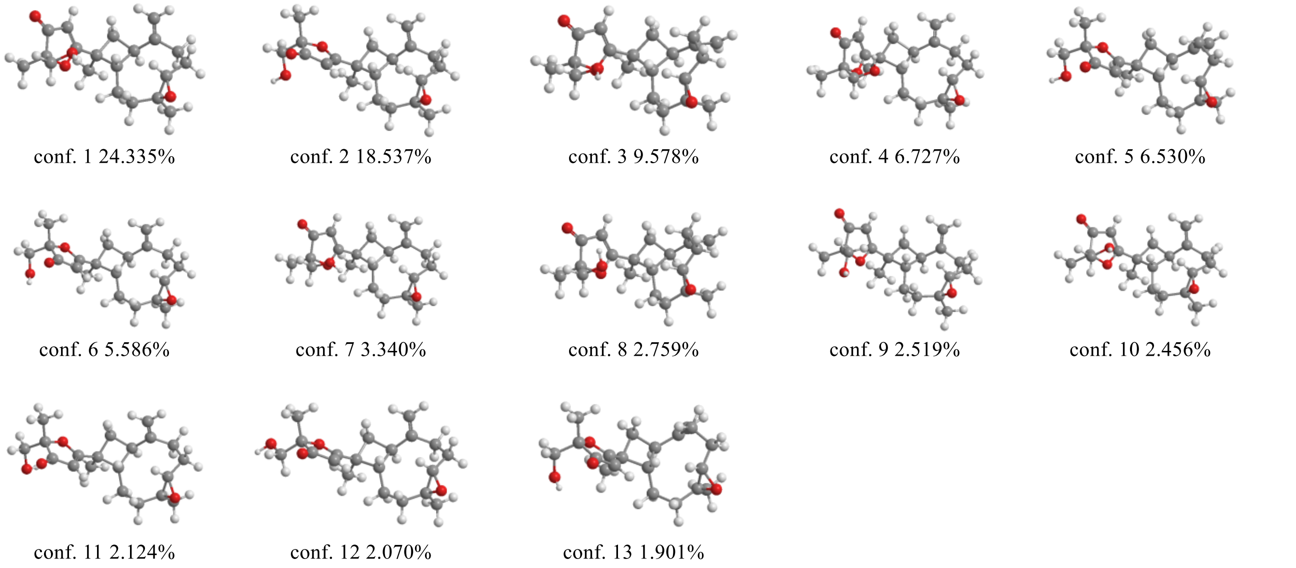

Figure S101. Re-optimized conformers above 1% population (OPLS_2005) of (1*S*,4*S*,5*S*,9*R*,11*S*,15*R*)-**3** calculated at the MPW1PW91/6-31G(d) level with IEFPCM solvent model for acetonitrile.

Table S10. Cartesian coordinates for the re-optimized conformers of (1*S*,4*S*,5*S*,9*R*,11*S*,15*R*)-**3** at the MPW1PW91/6-31G(d) level with IEFPCM solvent model for acetonitrile.

MPW1PW91/6-31G(d) Energy / Hartree =-1080.09014065 a.u. MPW1PW91/6-31G(d) Energy / Hartree =-1080.08921629 a.u.

Population = 24.335% Population = 18.537%

**3** Conf. 1		Standard Orientation (Ångstroms)			**3** Conf. 2		Standard Orientation (Ångstroms)		
I	atom	X	Y	Z	I	atom	X	Y	Z
1	C	0.671789	1.008757	1.150341	1	C	0.634496	0.131849	0.967986
2	C	-0.0436	2.309536	0.707704	2	C	0.108077	1.587775	1.149566
3	H	-0.28728	-0.04605	-0.50369	3	H	-0.6199	-0.06846	-0.80124
4	H	-1.92077	1.567778	1.513374	4	H	-1.7876	0.82144	1.876193
5	C	-3.81509	1.56966	-0.35934	5	C	-3.80343	1.793751	0.443434
6	C	-2.33939	1.918232	-0.54493	6	C	-2.31265	1.980986	0.167704
7	C	-4.29385	0.361169	-1.19982	7	C	-4.57042	1.060013	-0.68279
8	C	-3.31953	-0.78036	-1.06333	8	C	-3.80328	-0.16219	-1.11846
9	C	-3.18579	-1.61227	0.141681	9	C	-3.68632	-1.39318	-0.32284
10	C	-1.79415	-2.1093	0.479605	10	C	-2.37736	-2.15176	-0.42048
11	C	-0.94603	-1.09415	1.271317	11	C	-1.2853	-1.63791	0.537989
12	C	-0.53591	0.187983	0.535986	12	C	-0.72718	-0.23155	0.275322
13	C	-1.37759	1.530724	0.56408	13	C	-1.33966	1.085744	0.913386
14	C	-1.95034	2.571442	-1.64379	14	C	-1.9143	2.930612	-0.68398
15	C	0.806373	0.90721	2.679877	15	C	0.88262	-0.55903	2.322487
16	C	1.978074	0.699834	0.499885	16	C	1.859312	0.004415	0.123966
17	O	-3.76597	-2.15189	-1.08024	17	O	-4.4827	-1.36197	-1.54175
18	C	-4.16229	-1.53788	1.295149	18	C	-4.50204	-1.63527	0.928654
19	H	-2.40657	-0.63902	-1.63886	19	H	-2.94808	0.069267	-1.75088
20	O	2.419511	-0.56215	0.691899	20	O	2.943303	0.651199	0.601776
21	C	3.671245	-0.73266	-0.04312	21	C	4.065301	0.405665	-0.30189
22	C	3.908849	0.652525	-0.68374	22	C	3.457139	-0.49105	-1.40291
23	C	2.795526	1.467341	-0.27016	23	C	2.072954	-0.6627	-1.04196
24	C	3.465455	-1.81907	-1.09067	24	C	5.156822	-0.31347	0.479472
25	O	4.880194	0.916115	-1.37873	25	O	4.094965	-0.91419	-2.35673
26	C	4.77617	-1.09363	0.941599	26	C	4.545717	1.738066	-0.86247
27	O	2.434226	-1.41305	-1.98267	27	O	4.640839	-1.54794	0.962071
28	H	0.326217	2.671031	-0.25069	28	H	0.460014	2.242901	0.353058
29	H	-0.03554	3.135191	1.421659	29	H	0.313552	2.06061	2.111406
30	H	-4.42595	2.438137	-0.62082	30	H	-4.26725	2.771537	0.600138
31	H	-4.00562	1.35916	0.696292	31	H	-3.92909	1.234527	1.373963
32	H	-5.29286	0.057915	-0.87841	32	H	-5.5694	0.787151	-0.3351
33	H	-4.36928	0.648271	-2.25341	33	H	-4.70053	1.728412	-1.53976
34	H	-1.28157	-2.36275	-0.45377	34	H	-2.01933	-2.09008	-1.453
35	H	-1.86949	-3.03432	1.063043	35	H	-2.5566	-3.21259	-0.20968
36	H	-1.44679	-0.84161	2.211421	36	H	-1.63608	-1.71035	1.572299
37	H	-0.02169	-1.60769	1.553245	37	H	-0.44607	-2.33834	0.463634
38	H	-0.91963	2.843941	-1.83473	38	H	-0.87318	3.110709	-0.923
39	H	-2.66866	2.856279	-2.40627	39	H	-2.63602	3.568583	-1.18473

40	H	1.145059	-0.0816	2.992173	40	H	1.076463	-1.62705	2.20095
41	H	1.53146	1.644653	3.035238	41	H	1.750981	-0.11049	2.808778
42	H	-0.14792	1.115288	3.16765	42	H	0.02234	-0.43824	2.983847
43	H	-5.12166	-1.12221	0.988738	43	H	-5.40912	-1.0317	0.944635
44	H	-4.33818	-2.54558	1.684579	44	H	-4.7955	-2.68889	0.97001
45	H	-3.7679	-0.93072	2.113491	45	H	-3.92551	-1.41624	1.830728
46	H	2.638149	2.498836	-0.54042	46	H	1.346015	-1.23636	-1.59386
47	H	4.419086	-1.95237	-1.61596	47	H	6.000156	-0.47061	-0.20423
48	H	3.208692	-2.75648	-0.58342	48	H	5.486791	0.331017	1.302978
49	H	5.733598	-1.11367	0.41742	49	H	5.308174	1.557061	-1.62247
50	H	4.834273	-0.34909	1.73746	50	H	3.716152	2.274406	-1.32651
51	H	4.594201	-2.07278	1.389392	51	H	4.969686	2.361953	-0.07301
52	H	2.293331	-2.12329	-2.61685	52	H	5.33484	-1.98152	1.468712

MPW1PW91/6-31G(d) Energy / Hartree =-1080.09093991 a.u. MPW1PW91/6-31G(d) Energy / Hartree =-1080.09110362 a.u.

Population = 9.578% Population = 6.727%

**3** Conf. 3		Standard Orientation (Ångstroms)			**3** Conf. 4		Standard Orientation (Ångstroms)		
I	atom	X	Y	Z	I	atom	X	Y	Z
1	C	0.584795	1.518271	0.672497	1	C	0.669139	1.000806	1.157567
2	C	-0.25284	2.432912	-0.26251	2	C	-0.04961	2.303733	0.727609
3	H	-0.42077	-0.15392	-0.23886	3	H	-0.2871	-0.03805	-0.50923
4	H	-1.9147	2.156348	1.095702	4	H	-1.92336	1.547936	1.527729
5	C	-2.61285	0.671906	-2.00874	5	C	-3.82113	1.568373	-0.34038
6	C	-2.69406	1.569693	-0.78224	6	C	-2.34649	1.921344	-0.5254
7	C	-3.32977	-0.69285	-1.8353	7	C	-4.29987	0.368219	-1.1927
8	C	-2.59454	-1.53557	-0.82773	8	C	-3.3228	-0.77273	-1.07155
9	C	-2.84592	-1.56234	0.624834	9	C	-3.18406	-1.61784	0.123706
10	C	-1.62787	-1.7267	1.514604	10	C	-1.79052	-2.11594	0.452048
11	C	-0.94199	-0.38144	1.846814	11	C	-0.94243	-1.10872	1.253874
12	C	-0.61521	0.486203	0.623453	12	C	-0.53659	0.183007	0.533181
13	C	-1.53816	1.675078	0.189174	13	C	-1.38164	1.52315	0.57722
14	C	-3.78413	2.317965	-0.58281	14	C	-1.96081	2.587823	-1.61746
15	C	0.837944	2.15	2.050905	15	C	0.801877	0.883632	2.686056
16	C	1.847437	0.949674	0.119264	16	C	1.976038	0.700551	0.505055
17	O	-3.18693	-2.69961	-0.21895	17	O	-3.76649	-2.14484	-1.10254
18	C	-4.05333	-0.92176	1.272011	18	C	-4.1576	-1.55866	1.280544
19	H	-1.56365	-1.72685	-1.12623	19	H	-2.41164	-0.62327	-1.64795
20	O	2.397851	-0.02953	0.869249	20	O	2.43808	-0.55006	0.713388
21	C	3.57345	-0.54362	0.170559	21	C	3.684344	-0.71482	-0.02995
22	C	3.638231	0.313548	-1.11282	22	C	3.893545	0.658755	-0.69787
23	C	2.522158	1.220698	-1.03023	23	C	2.776771	1.467174	-0.28574
24	C	3.338808	-2.01743	-0.13446	24	C	3.475602	-1.82659	-1.06483
25	O	4.504229	0.184083	-1.96677	25	O	4.840799	0.912678	-1.43158
26	C	4.799792	-0.33167	1.049269	26	C	4.805264	-1.05111	0.944966
27	O	2.180834	-2.14415	-0.95217	27	O	2.413938	-1.56699	-1.97226
28	H	-0.03318	2.253693	-1.31651	28	H	0.317963	2.675641	-0.22761

29	H	-0.21948	3.506082	-0.06651	29	H	-0.04273	3.122415	1.449505
30	H	-1.56912	0.49297	-2.28755	30	H	-4.43396	2.438653	-0.59103
31	H	-3.07219	1.188519	-2.85501	31	H	-4.00902	1.346168	0.713311
32	H	-4.36855	-0.53191	-1.54097	32	H	-5.2973	0.059483	-0.87156
33	H	-3.34164	-1.21296	-2.79959	33	H	-4.37902	0.666663	-2.24285
34	H	-0.91406	-2.37659	0.999062	34	H	-1.28012	-2.35751	-0.4856
35	H	-1.9069	-2.23048	2.446896	35	H	-1.86246	-3.04792	1.024711
36	H	-1.55551	0.189931	2.550785	36	H	-1.44165	-0.86809	2.197893
37	H	-0.01161	-0.61224	2.373571	37	H	-0.01651	-1.62382	1.527805
38	H	-3.88948	2.951221	0.292178	38	H	-0.93099	2.864422	-1.80735
39	H	-4.60902	2.32139	-1.2888	39	H	-2.68119	2.880278	-2.37508
40	H	1.261092	1.426858	2.749566	40	H	1.140038	-0.10821	2.988671
41	H	1.539938	2.983099	1.956251	41	H	1.526396	1.617452	3.050003
42	H	-0.08814	2.539365	2.477953	42	H	-0.15305	1.087004	3.174401
43	H	-4.39124	-1.55833	2.096267	43	H	-5.11901	-1.14273	0.980972
44	H	-3.81763	0.062305	1.680745	44	H	-4.32941	-2.571	1.659614
45	H	-4.87787	-0.81162	0.569034	45	H	-3.76294	-0.95907	2.104317
46	H	2.26062	1.964193	-1.76549	46	H	2.601574	2.490698	-0.57447
47	H	4.232934	-2.39183	-0.64747	47	H	4.426109	-1.97967	-1.58755
48	H	3.22085	-2.56111	0.810206	48	H	3.217031	-2.74769	-0.53909
49	H	5.696176	-0.61453	0.494125	49	H	5.756186	-1.06569	0.408675
50	H	4.885906	0.719038	1.331761	50	H	4.865496	-0.29936	1.733952
51	H	4.737234	-0.93511	1.957157	51	H	4.64055	-2.02871	1.403213
52	H	2.050363	-3.07943	-1.13846	52	H	2.725294	-0.94335	-2.63726

MPW1PW91/6-31G(d) Energy / Hartree =-1080.08985727 a.u. MPW1PW91/6-31G(d) Energy / Hartree =-1080.09019775 a.u.

Population = 6.530% Population = 5.586%

**3** Conf. 5		Standard Orientation (Ångstroms)			**3** Conf. 6		Standard Orientation (Ångstroms)		
I	atom	X	Y	Z	I	atom	X	Y	Z
1	C	-0.58639	0.707453	-0.86571	1	C	0.632021	0.145032	0.972203
2	C	0.016123	1.991568	-0.20969	2	C	0.105875	1.603294	1.133725
3	H	0.751458	-0.43371	0.3738	3	H	-0.62137	-0.07907	-0.79488
4	H	1.71116	1.756591	-1.52948	4	H	-1.79027	0.847363	1.869736
5	C	2.700795	1.241933	1.798115	5	C	-3.80547	1.796005	0.417628
6	C	2.574428	1.831701	0.400563	6	C	-2.31409	1.9839	0.145574
7	C	3.718969	0.076071	1.893791	7	C	-4.56695	1.050178	-0.7044
8	C	3.206903	-1.11767	1.133939	8	C	-3.79799	-0.17726	-1.12148
9	C	3.462236	-1.41331	-0.28749	9	C	-3.68521	-1.39825	-0.31
10	C	2.321319	-2.05576	-1.05414	10	C	-2.37578	-2.15791	-0.39118
11	C	1.324149	-1.02409	-1.63122	11	C	-1.28794	-1.63036	0.564708
12	C	0.797748	0.009512	-0.62251	12	C	-0.72946	-0.22769	0.283734
13	C	1.438635	1.438302	-0.51962	13	C	-1.34171	1.098481	0.903671
14	C	3.451112	2.754729	-0.00841	14	C	-1.91486	2.926403	-0.71356
15	C	-0.95545	0.928954	-2.34403	15	C	0.88048	-0.52774	2.335697
16	C	-1.75714	0.118333	-0.15123	16	C	1.856425	0.007371	0.129662
17	O	4.061089	-2.21877	0.767877	17	O	-4.47515	-1.38223	-1.53339

18	C	4.482969	-0.67361	-1.12225	18	C	-4.50744	-1.62493	0.940101
19	H	2.249368	-1.46964	1.51847	19	H	-2.93958	0.046324	-1.75236
20	O	-2.84027	0.920515	-0.08167	20	O	2.936642	0.671064	0.588514
21	C	-3.90841	0.198175	0.606204	21	C	4.056731	0.41387	-0.31297
22	C	-3.26882	-1.1678	0.940002	22	C	3.454631	-0.51662	-1.38496
23	C	-1.92165	-1.09863	0.433752	23	C	2.072297	-0.68643	-1.02176
24	C	-5.08222	0.058128	-0.35413	24	C	5.158923	-0.28467	0.491262
25	O	-3.85899	-2.06348	1.527392	25	O	4.104635	-0.98389	-2.31179
26	C	-4.28918	0.964869	1.866234	26	C	4.531635	1.734655	-0.90416
27	O	-4.65694	-0.67166	-1.49822	27	O	4.740672	-1.50834	1.078502
28	H	-0.18168	2.026624	0.863897	28	H	0.458453	2.247276	0.328509
29	H	-0.25273	2.95015	-0.65657	29	H	0.311286	2.089071	2.089076
30	H	1.727307	0.896381	2.161886	30	H	-4.27173	2.774281	0.563648
31	H	3.023128	2.02825	2.484955	31	H	-3.93337	1.245052	1.352764
32	H	4.691953	0.401386	1.520552	32	H	-5.56782	0.781693	-0.35879
33	H	3.849536	-0.19697	2.946874	33	H	-4.69223	1.708879	-1.56955
34	H	1.793289	-2.7313	-0.37422	34	H	-2.01305	-2.11036	-1.42284
35	H	2.715732	-2.66934	-1.87188	35	H	-2.55582	-3.21572	-0.16649
36	H	1.776069	-0.50581	-2.483	36	H	-1.64266	-1.68832	1.598592
37	H	0.478223	-1.58656	-2.03936	37	H	-0.44827	-2.33158	0.503701
38	H	3.408598	3.178768	-1.0065	38	H	-0.87336	3.107121	-0.95036
39	H	4.241189	3.118083	0.641702	39	H	-2.63634	3.557875	-1.2228
40	H	-1.19968	-0.01473	-2.83773	40	H	1.079168	-1.59637	2.228109
41	H	-1.82602	1.583632	-2.41537	41	H	1.745543	-0.06916	2.818571
42	H	-0.13154	1.39586	-2.88675	42	H	0.018007	-0.40218	2.993231
43	H	5.245788	-0.20095	-0.50525	43	H	-5.41549	-1.02259	0.943198
44	H	4.977143	-1.38773	-1.78901	44	H	-4.79965	-2.67832	0.99377
45	H	4.015327	0.095075	-1.73957	45	H	-3.93624	-1.39301	1.842366
46	H	-1.18628	-1.88468	0.493249	46	H	1.348846	-1.28069	-1.55623
47	H	-5.88314	-0.46604	0.181661	47	H	6.019481	-0.42655	-0.17158
48	H	-5.43744	1.058722	-0.62794	48	H	5.45835	0.369849	1.312247
49	H	-5.01025	0.380538	2.440985	49	H	5.291388	1.538005	-1.66317
50	H	-3.40813	1.133772	2.487825	50	H	3.700712	2.262709	-1.3754
51	H	-4.73219	1.930738	1.615016	51	H	4.960028	2.374105	-0.12936
52	H	-5.40204	-0.73312	-2.10422	52	H	4.758821	-2.19251	0.400699

MPW1PW91/6-31G(d) Energy / Hartree =-1080.09145094 a.u. MPW1PW91/6-31G(d) Energy / Hartree =-1080.09180283 a.u.

Population = 3.340% Population = 2.759%

**3** Conf. 7		Standard Orientation (Ångstroms)			**3** Conf. 8		Standard Orientation (Ångstroms)		
I	atom	X	Y	Z	I	atom	X	Y	Z
1	C	0.672261	0.983315	1.12797	1	C	0.585108	1.513301	0.675696
2	C	-0.04853	2.291772	0.71885	2	C	-0.24325	2.423306	-0.27207
3	H	-0.3084	-0.02714	-0.54341	3	H	-0.41645	-0.16927	-0.22493
4	H	-1.91131	1.529387	1.538376	4	H	-1.91875	2.158699	1.071153
5	C	-3.83771	1.577885	-0.29863	5	C	-2.58879	0.645161	-2.02454
6	C	-2.36524	1.929043	-0.50308	6	C	-2.67822	1.55936	-0.81103

7	C	-4.33344	0.389171	-1.15723	7	C	-3.31716	-0.71188	-1.83938
8	C	-3.35636	-0.75477	-1.06729	8	C	-2.60061	-1.54433	-0.81007
9	C	-3.19954	-1.61597	0.114147	9	C	-2.8674	-1.54409	0.640125
10	C	-1.8018	-2.12101	0.412782	10	C	-1.66016	-1.70738	1.544727
11	C	-0.9383	-1.12686	1.214209	11	C	-0.95783	-0.36618	1.85948
12	C	-0.54133	0.17518	0.50704	12	C	-0.61662	0.483162	0.626984
13	C	-1.38416	1.515612	0.579559	13	C	-1.53316	1.669804	0.172548
14	C	-1.99477	2.607529	-1.59301	14	C	-3.76569	2.316856	-0.63383
15	C	0.815017	0.849851	2.654661	15	C	0.831586	2.154435	2.051285
16	C	1.978378	0.695381	0.468041	16	C	1.852957	0.944962	0.134592
17	O	-3.80256	-2.12575	-1.10927	17	O	-3.21163	-2.6916	-0.18819
18	C	-4.15346	-1.57097	1.287739	18	C	-4.07461	-0.88148	1.265058
19	H	-2.4545	-0.59885	-1.65661	19	H	-1.5685	-1.75109	-1.09384
20	O	2.451883	-0.5583	0.668188	20	O	2.38689	-0.04954	0.873712
21	C	3.723434	-0.69283	-0.04275	21	C	3.57025	-0.55593	0.183873
22	C	3.909413	0.680688	-0.71707	22	C	3.663956	0.328705	-1.07537
23	C	2.775005	1.47263	-0.31199	23	C	2.550744	1.237569	-0.99722
24	C	3.572989	-1.82146	-1.06703	24	C	3.31564	-2.02645	-0.16998
25	O	4.863853	0.958351	-1.42928	25	O	4.534963	0.197175	-1.92625
26	C	4.829419	-0.97788	0.964975	26	C	4.783015	-0.39074	1.089949
27	O	2.493041	-1.61652	-1.96518	27	O	2.16437	-2.22559	-0.97861
28	H	0.307209	2.669539	-0.23858	28	H	-0.01428	2.237665	-1.32303
29	H	-0.02936	3.104759	1.446878	29	H	-0.21058	3.497575	-0.08224
30	H	-4.45198	2.452664	-0.52917	30	H	-1.54323	0.455374	-2.28898
31	H	-4.00944	1.343828	0.755231	31	H	-3.03521	1.153002	-2.88293
32	H	-5.3257	0.078026	-0.82257	32	H	-4.35819	-0.5391	-1.55997
33	H	-4.43011	0.700656	-2.2021	33	H	-3.32142	-1.24675	-2.79563
34	H	-1.30821	-2.35102	-0.5368	34	H	-0.95204	-2.37829	1.048902
35	H	-1.86589	-3.06074	0.973523	35	H	-1.95511	-2.18904	2.483815
36	H	-1.42158	-0.89834	2.169348	36	H	-1.5661	0.221767	2.554289
37	H	-0.00913	-1.64746	1.46659	37	H	-0.0321	-0.60168	2.39234
38	H	-0.96732	2.883702	-1.79585	38	H	-3.87761	2.961346	0.232087
39	H	-2.72612	2.910941	-2.33565	39	H	-4.58191	2.316744	-1.34982
40	H	1.153054	-0.1457	2.944676	40	H	1.249475	1.435351	2.757368
41	H	1.543736	1.578301	3.020839	41	H	1.535244	2.98584	1.954828
42	H	-0.1359	1.050424	3.151788	42	H	-0.09657	2.54791	2.469934
43	H	-5.12034	-1.15372	1.008387	43	H	-4.4317	-1.50657	2.090003
44	H	-4.31724	-2.58756	1.65885	44	H	-3.83102	0.102597	1.669132
45	H	-3.74624	-0.9793	2.111103	45	H	-4.88832	-0.76581	0.550449
46	H	2.592188	2.496638	-0.59427	46	H	2.303805	1.995557	-1.7227
47	H	4.48473	-1.84485	-1.66746	47	H	4.219067	-2.41688	-0.65094
48	H	3.479469	-2.77723	-0.53873	48	H	3.146894	-2.58252	0.754087
49	H	5.790142	-1.00075	0.446319	49	H	5.685156	-0.66435	0.539322
50	H	4.867533	-0.19637	1.725967	50	H	4.877925	0.64697	1.414791
51	H	4.667608	-1.93969	1.455869	51	H	4.69761	-1.02849	1.972357
52	H	1.675601	-1.77027	-1.47957	52	H	2.388526	-2.00915	-1.89013

MPW1PW91/6-31G(d) Energy / Hartree =-1080.08921730 a.u. MPW1PW91/6-31G(d) Energy / Hartree =-1080.09014067 a.u.

Population = 2.519% Population = 2.456%

**3** Conf. 9		Standard Orientation (Ångstroms)			**3** Conf. 10		Standard Orientation (Ångstroms)		
I	atom	X	Y	Z	I	atom	X	Y	Z
1	C	0.599282	-1.02927	-1.08163	1	C	-0.67176	-1.00891	1.150425
2	C	-0.18672	-2.31887	-0.74034	2	C	0.043606	-2.30963	0.707675
3	H	-0.35755	-0.07104	0.632626	3	H	0.287215	0.046093	-0.50353
4	H	-1.98384	-1.42501	-1.57071	4	H	1.920823	-1.56798	1.513242
5	C	-3.9718	-1.48746	0.191805	5	C	3.815009	-1.5696	-0.3596
6	C	-2.52288	-1.90931	0.429849	6	C	2.339308	-1.91824	-0.54511
7	C	-4.45192	-0.32249	1.091003	7	C	4.293624	-0.36097	-1.19996
8	C	-3.42156	0.777106	1.104159	8	C	3.319252	0.780475	-1.06328
9	C	-3.17291	1.692031	-0.01918	9	C	3.185662	1.61233	0.141787
10	C	-1.74212	2.146463	-0.22938	10	C	1.794041	2.109288	0.479886
11	C	-0.8922	1.166873	-1.06234	11	C	0.946006	1.094018	1.271541
12	C	-0.58162	-0.19635	-0.43175	12	C	0.535911	-0.18805	0.536105
13	C	-1.48787	-1.48663	-0.5972	13	C	1.37758	-1.53084	0.563985
14	C	-2.22044	-2.65708	1.495285	14	C	1.950255	-2.5714	-1.64401
15	C	0.759814	-0.83059	-2.59957	15	C	-0.80638	-0.90748	2.679961
16	C	1.911989	-0.8277	-0.40312	16	C	-1.97804	-0.6999	0.499964
17	O	-3.80619	2.163652	1.204633	17	O	3.765715	2.152046	-1.08011
18	C	-4.0746	1.757552	-1.23254	18	C	4.162238	1.537762	1.295184
19	H	-2.55423	0.547463	1.720484	19	H	2.40625	0.639215	-1.63875
20	O	2.446631	0.401233	-0.56498	20	O	-2.41927	0.56219	0.691825
21	C	3.712143	0.459046	0.167317	21	C	-3.67101	0.732799	-0.04317
22	C	3.83619	-0.94747	0.790227	22	C	-3.90886	-0.65244	-0.68358
23	C	2.661421	-1.66533	0.364593	23	C	-2.79564	-1.46737	-0.26995
24	C	3.587144	1.508786	1.265889	24	C	-3.46512	1.818974	-1.09095
25	O	4.776375	-1.29662	1.491277	25	O	-4.8803	-0.91598	-1.37845
26	C	4.834069	0.742017	-0.82353	26	C	-4.7758	1.094171	0.941547
27	O	3.489034	2.79735	0.672342	27	O	-2.43405	1.412655	-1.98298
28	H	0.130952	-2.74528	0.210201	28	H	-0.32631	-2.67118	-0.25066
29	H	-0.18735	-3.10493	-1.49764	29	H	0.03563	-3.13531	1.421618
30	H	-4.62864	-2.34613	0.355888	30	H	4.4259	-2.43801	-0.62127
31	H	-4.09399	-1.19519	-0.85441	31	H	4.005613	-1.35926	0.696054
32	H	-5.41344	0.051357	0.731772	32	H	5.292631	-0.05766	-0.87861
33	H	-4.60855	-0.68259	2.112769	33	H	4.368997	-0.64795	-2.25359
34	H	-1.2789	2.291026	0.751545	34	H	1.281366	2.362829	-0.45342
35	H	-1.7403	3.122463	-0.72872	35	H	1.869403	3.034236	1.063434
36	H	-1.34496	1.025873	-2.04873	36	H	1.446802	0.841435	2.211615
37	H	0.070464	1.657054	-1.24061	37	H	0.021635	1.60747	1.553526
38	H	-1.21316	-2.98721	1.718387	38	H	0.919568	-2.84393	-1.83497
39	H	-2.98987	-2.96565	2.196244	39	H	2.668604	-2.85612	-2.40652
40	H	1.140951	0.162617	-2.83955	40	H	-1.1451	0.081298	2.992318
41	H	1.461387	-1.57046	-2.99486	41	H	-1.53145	-1.64498	3.035232
42	H	-0.19366	-0.96804	-3.11289	42	H	0.147917	-1.11557	3.167729

43	H	-5.07601	1.387964	-1.01398	43	H	4.337907	2.545352	1.684989
44	H	-4.16212	2.797736	-1.56147	44	H	3.76802	0.930189	2.113308
45	H	-3.66715	1.17984	-2.06558	45	H	5.121694	1.122433	0.988558
46	H	2.427682	-2.6877	0.613627	46	H	-2.63836	-2.4989	-0.54016
47	H	2.70241	1.283482	1.87398	47	H	-4.41879	1.952265	-1.61618
48	H	4.475727	1.420081	1.902757	48	H	-3.2082	2.756463	-0.58393
49	H	5.792224	0.682132	-0.30279	49	H	-5.73327	1.114338	0.417442
50	H	4.828678	0.002838	-1.62676	50	H	-4.83402	0.349793	1.737548
51	H	4.721768	1.739152	-1.24783	51	H	-4.5936	2.073364	1.389154
52	H	3.442142	3.445271	1.38273	52	H	-2.29301	2.122846	-2.61718

MPW1PW91/6-31G(d) Energy / Hartree =-1080.09087644 a.u. MPW1PW91/6-31G(d) Energy / Hartree =-1080.08852885 a.u.

Population = 2.124% Population = 2.070%

**3** Conf. 11		Standard Orientation (Ångstroms)			**3** Conf. 12		Standard Orientation (Ångstroms)		
I	atom	X	Y	Z	I	atom	X	Y	Z
1	C	0.637331	0.104903	0.978527	1	C	0.602315	0.192038	0.912225
2	C	0.125378	1.566716	1.15756	2	C	0.134715	1.678995	0.852234
3	H	-0.6178	-0.08099	-0.79101	3	H	-0.69158	-0.24037	-0.78708
4	H	-1.7762	0.818378	1.887158	4	H	-1.76793	1.112606	1.728344
5	C	-3.7855	1.801452	0.44843	5	C	-3.78602	1.910527	0.193115
6	C	-2.29272	1.983548	0.180377	6	C	-2.29643	2.002628	-0.13262
7	C	-4.5512	1.080945	-0.687	7	C	-4.5995	1.029197	-0.786
8	C	-3.79109	-0.14519	-1.12372	8	C	-3.87636	-0.26902	-1.03559
9	C	-3.69181	-1.38147	-0.33391	9	C	-3.78871	-1.36782	-0.06304
10	C	-2.38838	-2.15045	-0.42316	10	C	-2.50495	-2.17393	-0.05386
11	C	-1.29947	-1.64649	0.544223	11	C	-1.38249	-1.54826	0.797503
12	C	-0.72772	-0.24483	0.28507	12	C	-0.78325	-0.22433	0.302853
13	C	-1.32709	1.078421	0.923658	13	C	-1.33516	1.201323	0.727334
14	C	-1.88645	2.938904	-0.66108	14	C	-1.88505	2.798538	-1.12401
15	C	0.874604	-0.58428	2.335409	15	C	0.840782	-0.26665	2.363406
16	C	1.858888	-0.03038	0.131014	16	C	1.807971	-0.12245	0.088721
17	O	-4.47684	-1.33684	-1.55958	17	O	-4.59908	-1.49587	-1.26628
18	C	-4.52046	-1.62288	0.909156	18	C	-4.59563	-1.38982	1.216848
19	H	-2.92853	0.082614	-1.74743	19	H	-3.02231	-0.16561	-1.70232
20	O	2.956029	0.600844	0.613109	20	O	2.943951	0.483233	0.493282
21	C	4.059002	0.395642	-0.32647	21	C	4.032945	0.070955	-0.39381
22	C	3.443842	-0.50278	-1.41769	22	C	3.346997	-0.88544	-1.39157
23	C	2.06186	-0.67964	-1.04546	23	C	1.96071	-0.92458	-1.00037
24	C	5.190046	-0.30844	0.427786	24	C	5.064561	-0.6926	0.430026
25	O	4.072902	-0.92523	-2.3772	25	O	3.938055	-1.45542	-2.29874
26	C	4.490582	1.744544	-0.88658	26	C	4.594862	1.307441	-1.08266
27	O	4.781055	-1.52982	1.023221	27	O	5.709975	0.206068	1.323011
28	H	0.483184	2.216059	0.359037	28	H	0.493052	2.17512	-0.04943
29	H	0.337181	2.039346	2.118065	29	H	0.379985	2.29826	1.716829
30	H	-4.24491	2.780332	0.611132	30	H	-4.21719	2.915409	0.19511
31	H	-3.91807	1.235341	1.373845	31	H	-3.909	1.515402	1.204931

32	H	-5.55474	0.814669	-0.34751	32	H	-5.59602	0.845595	-0.37818
33	H	-4.66976	1.755281	-1.54096	33	H	-4.73268	1.55634	-1.73605
34	H	-2.02199	-2.08998	-1.45282	34	H	-2.16267	-2.2868	-1.08722
35	H	-2.57755	-3.2101	-0.2154	35	H	-2.71153	-3.18175	0.324707
36	H	-1.65856	-1.7162	1.575869	36	H	-1.71911	-1.43852	1.83311
37	H	-0.46578	-2.35412	0.47577	37	H	-0.56724	-2.27953	0.83028
38	H	-0.84368	3.116959	-0.89414	38	H	-0.84345	2.903519	-1.40219
39	H	-2.60331	3.584629	-1.15886	39	H	-2.59496	3.380192	-1.70391
40	H	1.070064	-1.65261	2.217855	40	H	0.996336	-1.34612	2.426329
41	H	1.734786	-0.13116	2.83258	41	H	1.726744	0.229721	2.763583
42	H	0.008236	-0.46442	2.988752	42	H	-0.0091	-0.00592	2.996972
43	H	-5.42306	-1.01254	0.920108	43	H	-5.48507	-0.7646	1.145601
44	H	-4.82222	-2.6744	0.943301	44	H	-4.91828	-2.41527	1.422472
45	H	-3.95008	-1.41209	1.817066	45	H	-4.00248	-1.05171	2.069938
46	H	1.329355	-1.24339	-1.60014	46	H	1.188812	-1.49553	-1.49023
47	H	5.968458	-0.55421	-0.29729	47	H	4.557501	-1.49862	0.974752
48	H	5.610059	0.379802	1.170619	48	H	5.772555	-1.1452	-0.27514
49	H	5.254642	1.589239	-1.65113	49	H	5.335585	0.996753	-1.82268
50	H	3.643457	2.257757	-1.3454	50	H	3.800266	1.853647	-1.5944
51	H	4.900748	2.377979	-0.09744	51	H	5.073096	1.961286	-0.35423
52	H	4.193073	-1.31346	1.755027	52	H	6.369559	-0.29287	1.815541

MPW1PW91/6-31G(d) Energy / Hartree =-1080.09083900 a.u.

Population = 1.901%

**3** Conf. 13		Standard Orientation (Ångstroms)			**3** Conf. 13		Standard Orientation (Ångstroms)		
I	atom	X	Y	Z	I	atom	X	Y	Z
1	C	-0.58355	0.711462	-0.87027	27	O	-4.70848	-0.72254	-1.55097
2	C	0.02728	2.000627	-0.23024	28	H	-0.16932	2.050212	0.842981
3	H	0.75043	-0.42178	0.381634	29	H	-0.23712	2.954697	-0.6892
4	H	1.71987	1.740327	-1.54828	30	H	1.734597	0.922189	2.153333
5	C	2.709712	1.257323	1.784194	31	H	3.037959	2.049352	2.4616
6	C	2.585032	1.832209	0.380296	32	H	4.695447	0.401617	1.514193
7	C	3.720858	0.08638	1.891937	33	H	3.850867	-0.17558	2.947893
8	C	3.200944	-1.11279	1.146097	34	H	1.776428	-2.73507	-0.34235
9	C	3.452908	-1.42575	-0.27225	35	H	2.697176	-2.6949	-1.8418
10	C	2.307428	-2.07012	-1.03035	36	H	1.769282	-0.53322	-2.47573
11	C	1.315542	-1.0391	-1.61752	37	H	0.465604	-1.60099	-2.01807
12	C	0.796922	0.00917	-0.61996	38	H	3.425197	3.159073	-1.04237
13	C	1.446214	1.435226	-0.53468	39	H	4.259214	3.111782	0.605527
14	C	3.466406	2.74576	-0.03975	40	H	-1.21026	-0.03008	-2.83081
15	C	-0.95227	0.917031	-2.35078	41	H	-1.81337	1.582963	-2.43062
16	C	-1.75561	0.1392	-0.14457	42	H	-0.12242	1.364195	-2.90087
17	O	4.048442	-2.22275	0.791302	43	H	5.243807	-0.22766	-0.50454
18	C	4.476311	-0.69999	-1.11594	44	H	4.963897	-1.42256	-1.77841
19	H	2.241937	-1.45498	1.53574	45	H	4.012089	0.06714	-1.73776
20	O	-2.84805	0.929064	-0.11675	46	H	-1.16942	-1.83131	0.584974

21	C	-3.91399	0.221629	0.58833	47	H	-5.89558	-0.45843	0.14913
22	C	-3.26213	-1.11827	0.983745	48	H	-5.40807	0.996386	-0.74402
23	C	-1.91135	-1.05499	0.490798	49	H	-5.04677	0.459709	2.39617
24	C	-5.07269	0.017826	-0.39503	50	H	-3.46782	1.261926	2.430362
25	O	-3.85928	-2.00744	1.577559	51	H	-4.8005	1.975669	1.500509
26	C	-4.3332	1.036527	1.804501	52	H	-4.68876	-1.65751	-1.31987

2.2.4 ECD calculation for (1*S*,4*S*,9*R*,11*S*)-6

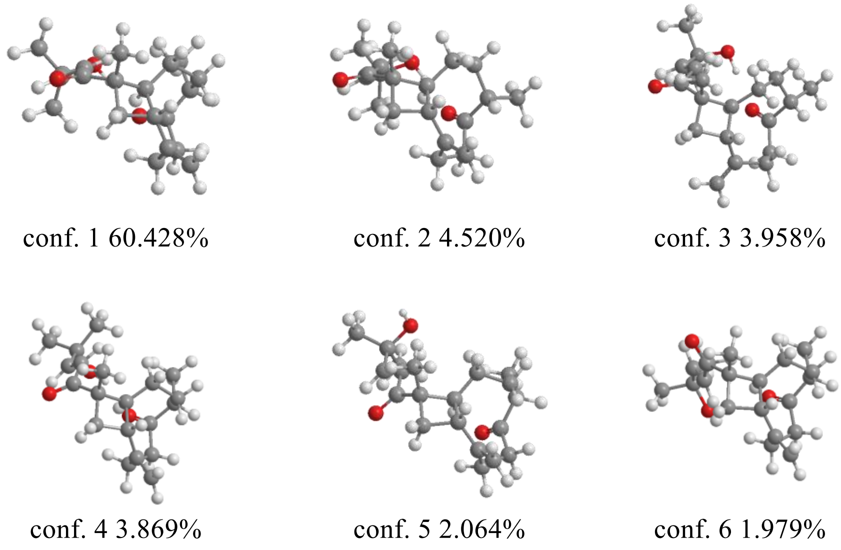

Figure S102. Re-optimized conformers above 1% population (OPLS_2005) of (1*S*,4*S*,9*R*,11*S*)-**6** calculated at the MPW1PW91/6-31G(d) level with IEFPCM solvent model for acetonitrile.

Table S11. Cartesian coordinates for the re-optimized conformers of (1*S*,4*S*,9*R*,11*S*)-**6** at the MPW1PW91/6-31G(d) level with IEFPCM solvent model for acetonitrile.

MPW1PW91/6-31G(d) Energy / Hartree =-1005.59606370 a.u. MPW1PW91/6-31G(d) Energy / Hartree =-1005.59299943 a.u.

Population = 60.428% Population = 4.520%

**6** Conf. 1		Standard Orientation (Ångstroms)			**6** Conf. 2		Standard Orientation (Ångstroms)		
I	atom	X	Y	Z	I	atom	X	Y	Z
1	C	-0.71925	2.141646	0.481729	1	C	-0.39403	2.31079	0.412763
2	C	-1.68873	2.295241	-0.72629	2	C	-1.39791	2.513585	-0.75683
3	C	-2.08788	-0.82657	-1.99589	3	C	-2.08279	-0.45288	-2.04093
4	C	-3.02889	0.078937	-1.22867	4	C	-2.93678	0.390012	-1.1138
5	C	-1.81382	-2.19199	-1.31473	5	C	-1.85011	-1.91777	-1.58819
6	C	-0.68008	-2.2055	-0.30503	6	C	-0.98107	-2.02225	-0.35754
7	C	-1.00947	-2.50531	1.144477	7	C	-1.66416	-2.0544	1.001716
8	C	-1.95946	-1.45525	1.772988	8	C	-0.92259	-1.23419	2.062693
9	C	-1.39895	-0.03704	1.931449	9	C	-1.20076	0.273047	2.01002
10	C	-1.22055	0.672626	0.601911	10	C	-1.02406	0.901756	0.626904
11	C	-2.46844	1.049867	-0.22722	11	C	-2.26165	1.358913	-0.18404
12	C	-4.34666	0.012197	-1.44739	12	C	-4.26881	0.271833	-1.13101
13	C	-1.0784	3.058426	1.648426	13	C	-0.59772	3.309294	1.548812
14	C	0.742404	2.336058	0.099668	14	C	1.064931	2.312117	-0.02509
15	C	1.648635	1.184837	0.295566	15	C	1.846481	1.099808	0.302993

16	C	2.743874	0.998601	-0.44789	16	C	2.859248	0.669896	-0.45582
17	C	3.60241	-0.24194	-0.39616	17	C	3.543713	-0.66433	-0.27404
18	C	3.758403	-0.80015	-1.81661	18	C	3.582789	-1.39332	-1.62266
19	C	4.967318	0.112826	0.195303	19	C	4.958421	-0.44014	0.260901
20	O	3.04755	-1.23018	0.456057	20	O	2.877857	-1.46568	0.689962
21	O	0.46802	-2.02761	-0.69301	21	O	0.234563	-2.11681	-0.46966
22	C	0.236274	-2.76532	1.981581	22	C	-1.75625	-3.53783	1.401813
23	O	1.111332	3.388607	-0.40434	23	O	1.523266	3.246009	-0.66854
24	H	-0.5621	0.083547	-0.04082	24	H	-0.44319	0.227806	-0.00818
25	H	-3.27183	1.378996	0.440312	25	H	-3.01069	1.776785	0.496841
26	H	-1.18348	2.112774	-1.67941	26	H	-0.95138	2.268932	-1.72474
27	H	-2.24691	3.232198	-0.79755	27	H	-1.87682	3.493193	-0.83147
28	H	-1.12891	-0.33597	-2.19869	28	H	-1.10701	0.015064	-2.20931
29	H	-2.53625	-1.04122	-2.96948	29	H	-2.56972	-0.50859	-3.01882
30	H	-2.73763	-2.57387	-0.87341	30	H	-2.82013	-2.39072	-1.40757
31	H	-1.50566	-2.89734	-2.09435	31	H	-1.3439	-2.44767	-2.39959
32	H	-1.59856	-3.43393	1.102178	32	H	-2.68303	-1.66831	0.880265
33	H	-2.23787	-1.84088	2.759626	33	H	0.15141	-1.426	1.957102
34	H	-2.88956	-1.41043	1.194033	34	H	-1.20447	-1.60717	3.053493
35	H	-2.09086	0.535271	2.560578	35	H	-2.22224	0.462599	2.362324
36	H	-0.44883	-0.06675	2.479507	36	H	-0.54024	0.764906	2.734037
37	H	-5.0479	0.627157	-0.88996	37	H	-4.9004	0.853372	-0.46545
38	H	-4.77078	-0.65712	-2.19124	38	H	-4.77536	-0.40769	-1.81175
39	H	-0.52828	2.790234	2.556152	39	H	-0.00753	3.047424	2.432652
40	H	-0.83238	4.092725	1.392158	40	H	-0.29231	4.307301	1.221343
41	H	-2.14753	3.00903	1.875504	41	H	-1.64962	3.354125	1.847035
42	H	1.354216	0.417083	1.001995	42	H	1.498683	0.475558	1.119221
43	H	3.025822	1.759077	-1.17438	43	H	3.192071	1.28235	-1.29253
44	H	4.390149	-1.69244	-1.78863	44	H	4.091609	-2.35392	-1.50221
45	H	2.782886	-1.07753	-2.22798	45	H	2.566673	-1.58086	-1.98327
46	H	4.217592	-0.06806	-2.48719	46	H	4.11423	-0.80941	-2.37956
47	H	5.606206	-0.77505	0.201035	47	H	5.472634	-1.40103	0.354369
48	H	5.459614	0.892919	-0.39097	48	H	5.535428	0.200555	-0.41087
49	H	4.851994	0.46817	1.222564	49	H	4.917905	0.033468	1.245219
50	H	2.204556	-1.53315	0.067607	50	H	2.00545	-1.70817	0.328189
51	H	0.818755	-3.59188	1.566543	51	H	-2.25552	-4.13897	0.635606
52	H	0.89451	-1.89464	2.027129	52	H	-0.75563	-3.95036	1.56048
53	H	-0.04859	-3.02858	3.004121	53	H	-2.32366	-3.63647	2.330961

MPW1PW91/6-31G(d) Energy / Hartree =-1005.59127526 a.u. MPW1PW91/6-31G(d) Energy / Hartree =-1005.59075441 a.u.

Population = 3.958% Population = 3.869%

**6** Conf. 3		Standard Orientation (Ångstroms)			**6** Conf. 4		Standard Orientation (Ångstroms)		
I	atom	X	Y	Z	I	atom	X	Y	Z
1	C	-0.62248	2.222839	0.680693	1	C	-0.65842	2.131339	0.512896
2	C	-1.6834	2.582719	-0.39319	2	C	-1.85879	2.371216	-0.43428
3	C	-3.21137	-1.05127	-0.97372	3	C	-2.43917	-0.66631	-1.87683

4	C	-2.86754	0.395318	-1.27512	4	C	-3.23236	0.168538	-0.89367
5	C	-2.10642	-2.07608	-1.32383	5	C	-2.07544	-2.08272	-1.36211
6	C	-0.92	-2.13575	-0.39291	6	C	-0.80453	-2.17785	-0.53526
7	C	-1.17191	-2.4788	1.067717	7	C	-0.92531	-2.58263	0.921437
8	C	-0.62625	-1.40545	2.021475	8	C	-1.7107	-1.54639	1.763911
9	C	-1.33182	-0.04644	1.967251	9	C	-1.07383	-0.15869	1.916838
10	C	-1.15369	0.750305	0.66712	10	C	-1.12089	0.640179	0.626288
11	C	-2.39729	1.242125	-0.12524	11	C	-2.50527	1.057193	0.077766
12	C	-3.00803	0.873221	-2.51432	12	C	-4.56874	0.11433	-0.89535
13	C	-0.80734	2.996518	1.983265	13	C	-0.71922	2.941483	1.806076
14	C	0.811431	2.39198	0.191624	14	C	0.69462	2.303576	-0.15642
15	C	1.69683	1.219771	0.358884	15	C	1.775185	1.396711	0.303755
16	C	2.701478	0.951247	-0.48041	16	C	2.65061	0.897271	-0.57573
17	C	3.471938	-0.34914	-0.48548	17	C	3.656535	-0.20443	-0.34302
18	C	3.548987	-0.87644	-1.92317	18	C	4.981617	0.171512	-1.00065
19	C	4.873657	-0.11533	0.078583	19	C	3.876993	-0.54017	1.128724
20	O	2.863655	-1.31891	0.354758	20	O	3.189009	-1.35291	-1.05771
21	O	0.220095	-1.98305	-0.81376	21	O	0.27695	-1.97372	-1.0697
22	C	-0.51971	-3.83895	1.352622	22	C	0.42123	-2.94762	1.529384
23	O	1.167546	3.423053	-0.36132	23	O	0.855273	3.088788	-1.07845
24	H	-0.5179	0.197922	-0.02779	24	H	-0.57825	0.100145	-0.1528
25	H	-3.23418	1.385753	0.572181	25	H	-3.17363	1.292171	0.913456
26	H	-1.24598	2.642513	-1.39236	26	H	-1.55666	2.312901	-1.48307
27	H	-2.25926	3.493292	-0.21014	27	H	-2.43682	3.287008	-0.28599
28	H	-4.08991	-1.33299	-1.56126	28	H	-1.52101	-0.15746	-2.19163
29	H	-3.50114	-1.16166	0.076665	29	H	-3.04204	-0.8012	-2.77882
30	H	-2.56357	-3.07494	-1.29221	30	H	-2.927	-2.50322	-0.82116
31	H	-1.74231	-1.90676	-2.33976	31	H	-1.89342	-2.71827	-2.23575
32	H	-2.25184	-2.57197	1.227117	32	H	-1.55837	-3.4822	0.909606
33	H	0.4478	-1.29064	1.833967	33	H	-1.84066	-1.98985	2.756949
34	H	-0.71619	-1.80164	3.039402	34	H	-2.71973	-1.43159	1.350542
35	H	-2.4018	-0.19745	2.159886	35	H	-1.62058	0.379549	2.700032
36	H	-0.96934	0.547232	2.813495	36	H	-0.04283	-0.25399	2.277187
37	H	-2.76963	1.900435	-2.77025	37	H	-5.16216	0.676338	-0.17926
38	H	-3.3751	0.246401	-3.32288	38	H	-5.11651	-0.49092	-1.61283
39	H	-0.13328	2.648076	2.772491	39	H	0.038069	2.618664	2.527811
40	H	-0.6017	4.056804	1.808925	40	H	-0.55809	4.003118	1.594941
41	H	-1.83439	2.90418	2.350395	41	H	-1.69925	2.839481	2.281688
42	H	1.426369	0.485211	1.108698	42	H	1.744897	1.051898	1.333321
43	H	2.957216	1.677172	-1.25072	43	H	2.585024	1.239465	-1.6079
44	H	4.107094	-1.81684	-1.93636	44	H	5.680885	-0.66595	-0.9224
45	H	2.545192	-1.06236	-2.31782	45	H	4.832331	0.398052	-2.05987
46	H	4.0502	-0.16348	-2.58419	46	H	5.426166	1.043781	-0.51434
47	H	5.448477	-1.04483	0.03159	47	H	4.600239	-1.35601	1.206566
48	H	5.403564	0.651759	-0.49193	48	H	4.265698	0.320989	1.680128
49	H	4.810565	0.20652	1.121319	49	H	2.95326	-0.86675	1.613945
50	H	2.002285	-1.56156	-0.03554	50	H	2.267795	-1.53191	-0.79706

51	H	-0.90414	-4.61906	0.688676	51	H	0.904522	-3.74753	0.962707
52	H	0.56344	-3.77816	1.216489	52	H	1.103063	-2.09509	1.537876
53	H	-0.72332	-4.14192	2.383326	53	H	0.290423	-3.28812	2.560367

MPW1PW91/6-31G(d) Energy / Hartree =-1005.59296974 a.u. MPW1PW91/6-31G(d) Energy / Hartree =-1005.59664473 a.u.

Population = 2.064% Population = 1.979%

**6** Conf. 5		Standard Orientation (Ångstroms)			**6** Conf. 6		Standard Orientation (Ångstroms)		
I	atom	X	Y	Z	I	atom	X	Y	Z
1	C	-0.21467	1.94942	0.400811	1	C	-0.67647	2.217975	0.265068
2	C	-1.4209	2.522955	-0.37335	2	C	-1.22699	2.160367	-1.19375
3	C	-3.2079	-0.1315	-1.62691	3	C	-1.55796	-1.11226	-2.052
4	C	-3.46627	0.857215	-0.51187	4	C	-2.58191	-0.049	-1.71477
5	C	-3.18722	-1.61021	-1.1657	5	C	-1.57046	-2.33976	-1.10746
6	C	-1.8305	-2.10756	-0.695	6	C	-0.73036	-2.20292	0.148607
7	C	-1.67678	-2.54069	0.7529	7	C	-1.4264	-2.23527	1.496658
8	C	-1.98299	-1.40612	1.759101	8	C	-2.459	-1.09396	1.674884
9	C	-0.9837	-0.24354	1.8003	9	C	-1.89483	0.328961	1.768125
10	C	-1.02891	0.616015	0.549263	10	C	-1.30425	0.793579	0.451425
11	C	-2.31524	1.431187	0.267115	11	C	-2.22795	1.056169	-0.75951
12	C	-4.72003	1.224354	-0.22374	12	C	-3.79623	-0.09492	-2.27392
13	C	0.063742	2.680548	1.714966	13	C	-1.33554	3.31362	1.087343
14	C	1.069641	1.833772	-0.39669	14	C	0.83643	2.244869	0.429093
15	C	2.073408	0.861207	0.094288	15	C	1.601448	1.181964	-0.26494
16	C	3.186367	0.596083	-0.59821	16	C	2.819869	0.799514	0.127751
17	C	4.239988	-0.40396	-0.20225	17	C	3.567872	-0.37108	-0.46357
18	C	4.224165	-1.56443	-1.19811	18	C	4.79523	0.142642	-1.21703
19	C	5.613156	0.277009	-0.1866	19	C	3.985946	-1.31911	0.667358
20	O	3.965544	-0.98382	1.066692	20	O	2.783753	-1.06771	-1.41862
21	O	-0.91177	-2.18982	-1.49345	21	O	0.488135	-2.1227	0.062561
22	C	-0.33203	-3.2119	1.001284	22	C	-0.43288	-2.32594	2.647907
23	O	1.254252	2.505328	-1.40288	23	O	1.388123	3.066917	1.148583
24	H	-0.78767	0.008803	-0.32851	24	H	-0.53355	0.084607	0.145791
25	H	-2.70055	1.815024	1.218436	25	H	-3.1525	1.533672	-0.41815
26	H	-1.2962	2.380916	-1.45015	26	H	-0.49155	1.792245	-1.91371
27	H	-1.69873	3.562867	-0.18195	27	H	-1.65402	3.089818	-1.57955
28	H	-2.26998	0.084635	-2.1511	28	H	-0.54166	-0.70334	-2.08292
29	H	-4.00679	-0.03233	-2.36679	29	H	-1.76725	-1.48193	-3.0592
30	H	-3.96131	-1.77415	-0.41161	30	H	-2.60168	-2.60788	-0.86552
31	H	-3.43405	-2.23485	-2.03124	31	H	-1.13019	-3.18338	-1.65059
32	H	-2.47466	-3.28364	0.90546	32	H	-2.02411	-3.15931	1.472718
33	H	-2.02404	-1.86802	2.751675	33	H	-3.01382	-1.31746	2.592372
34	H	-2.98718	-1.00844	1.570158	34	H	-3.19288	-1.13332	0.861398
35	H	-1.22111	0.374662	2.674149	35	H	-2.70712	1.000445	2.069894
36	H	0.027974	-0.63118	1.96725	36	H	-1.1378	0.386603	2.559486
37	H	-4.94091	1.904949	0.594123	37	H	-4.56321	0.634909	-2.02963
38	H	-5.56903	0.860645	-0.79656	38	H	-4.0659	-0.86089	-2.99617

39	H	0.786338	2.144226	2.337721	39	H	-1.10989	3.20273	2.151085
40	H	0.464301	3.679121	1.513642	40	H	-0.97277	4.295594	0.771677
41	H	-0.85415	2.80192	2.297594	41	H	-2.42208	3.298442	0.963988
42	H	1.868578	0.3317	1.019101	42	H	1.129214	0.636283	-1.07362
43	H	3.353191	1.130095	-1.53243	43	H	3.304411	1.32923	0.946469
44	H	5.005872	-2.28255	-0.93504	44	H	5.361419	-0.70428	-1.61539
45	H	3.256216	-2.07131	-1.17006	45	H	4.486395	0.78084	-2.04903
46	H	4.403355	-1.2091	-2.21569	46	H	5.448495	0.719752	-0.55761
47	H	6.379072	-0.4531	0.088805	47	H	4.546406	-2.15977	0.24855
48	H	5.862478	0.691908	-1.16702	48	H	4.616133	-0.81193	1.403566
49	H	5.633265	1.095117	0.541013	49	H	3.102883	-1.71051	1.181466
50	H	4.066636	-0.2887	1.732547	50	H	2.032121	-1.47845	-0.95293
51	H	-0.21238	-4.09145	0.363848	51	H	0.190182	-3.21902	2.556483
52	H	0.501417	-2.53884	0.785441	52	H	0.236042	-1.46234	2.673225
53	H	-0.25417	-3.53144	2.044653	53	H	-0.96518	-2.37613	3.602059

2.2.5 ECD calculation for (1*S*,4*S*,5*S*,9*R*,11*S*)-8

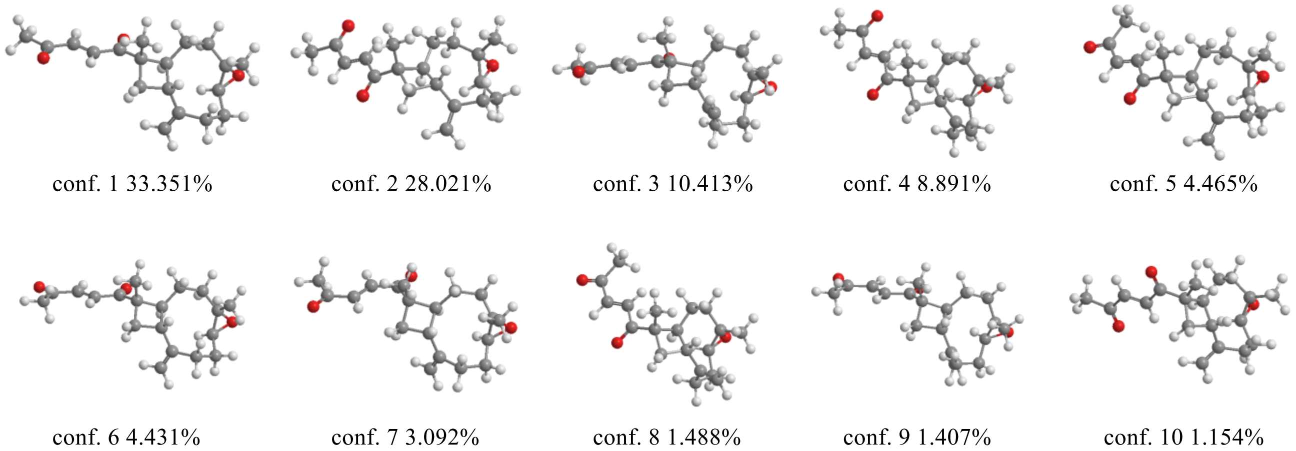

Figure S103. Re-optimized conformers above 1% population (OPLS_2005) of (1*S*,4*S*,5*S*,9*R*,11*S*)-**8** calculated at the MPW1PW91/6-31G(d) level with IEFPCM solvent model for acetonitrile.

Table S12. Cartesian coordinates for the re-optimized conformers of (1*S*,4*S*,5*S*,9*R*,11*S*)-**8** at the MPW1PW91/6-31G(d) level with IEFPCM solvent model for acetonitrile.

MPW1PW91/6-31G(d) Energy / Hartree =-965.04730410 a.u. MPW1PW91/6-31G(d) Energy / Hartree =-965.04802623 a.u.

Population = 33.351% Population = 28.021%

**8** Conf. 1		Standard Orientation (Ångstroms)			**8** Conf. 2		Standard Orientation (Ångstroms)		
I	atom	X	Y	Z	I	atom	X	Y	Z
1	C	0.863527	-0.27391	0.719954	1	C	0.763806	1.30165	0.913161
2	C	0.582662	1.23968	0.904857	2	C	-0.21663	2.44676	0.616016
3	C	-3.27466	2.027346	0.47613	3	C	-3.83138	1.131726	-0.30289
4	C	-1.80623	1.974749	0.082647	4	C	-2.43362	1.670908	-0.56707
5	C	-4.21763	1.421865	-0.57862	5	C	-4.17366	-0.12777	-1.11852
6	C	-3.66809	0.105624	-1.05343	6	C	-3.02383	-1.09481	-1.06435
7	C	-3.67916	-1.12589	-0.25712	7	C	-2.66753	-1.88257	0.120289
8	C	-2.51457	-2.07013	-0.44592	8	C	-1.19872	-2.16542	0.337757
9	C	-1.29572	-1.73581	0.42189	9	C	-0.45295	-1.05075	1.08199

10	C	-0.56075	-0.43145	0.116456	10	C	-0.30351	0.284832	0.352028
11	C	-0.92896	0.951808	0.770645	11	C	-1.37202	1.433759	0.48535
12	C	-1.33852	2.828767	-0.83356	12	C	-2.18771	2.342554	-1.69672
13	C	1.08723	-0.98064	2.061086	13	C	1.073709	1.15684	2.405287
14	C	1.999893	-0.61346	-0.21637	14	C	2.054438	1.349672	0.123895
15	C	3.338534	-0.05207	0.129661	15	C	2.902598	0.122537	0.126115
16	C	4.434513	-0.36025	-0.57283	16	C	4.095706	0.090009	-0.47843
17	C	5.769542	0.205883	-0.22381	17	C	4.939152	-1.13998	-0.47423
18	C	6.946308	-0.20091	-1.0762	18	C	6.25726	-1.04596	-1.19053
19	O	5.907839	0.967992	0.736188	19	O	4.568516	-2.1597	0.083276
20	C	-4.41781	-1.2369	1.053188	20	C	-3.5431	-1.94232	1.347054
21	O	1.843954	-1.31049	-1.21099	21	O	2.398669	2.341187	-0.49963
22	H	-0.47926	-0.29842	-0.9684	22	H	-0.10097	0.111828	-0.71266
23	H	-1.34072	0.757842	1.767794	23	H	-1.86549	1.321772	1.457585
24	O	-4.53987	-0.9722	-1.40619	24	O	-3.24742	-2.50715	-1.04483
25	H	-2.83019	0.210628	-1.7436	25	H	-2.19035	-0.81125	-1.70782
26	H	0.968899	1.830295	0.070729	26	H	0.034838	2.937741	-0.32474
27	H	0.917057	1.694932	1.841081	27	H	-0.32718	3.211343	1.389655
28	H	-3.56869	3.066521	0.656907	28	H	-4.57137	1.907212	-0.52638
29	H	-3.41684	1.493066	1.420929	29	H	-3.93434	0.898793	0.761825
30	H	-5.21949	1.29606	-0.15875	30	H	-5.08756	-0.58806	-0.7326
31	H	-4.31097	2.103691	-1.43118	31	H	-4.36833	0.144558	-2.16179
32	H	-2.22567	-2.05216	-1.503	32	H	-0.73104	-2.32938	-0.63974
33	H	-2.83644	-3.0952	-0.22261	33	H	-1.08789	-3.09947	0.902704
34	H	-1.57044	-1.76977	1.482859	34	H	-0.91208	-0.88342	2.063131
35	H	-0.57211	-2.54717	0.276917	35	H	0.550565	-1.43692	1.295818
36	H	-0.30545	2.835286	-1.1652	36	H	-1.21014	2.74243	-1.94521
37	H	-1.99114	3.563438	-1.29807	37	H	-2.97244	2.513123	-2.42921
38	H	1.10252	-2.06844	1.949854	38	H	1.629554	0.244055	2.638039
39	H	2.037733	-0.67359	2.508398	39	H	1.66858	2.010137	2.745965
40	H	0.29489	-0.72177	2.769249	40	H	0.149426	1.143106	2.989684
41	H	3.420647	0.625547	0.974998	41	H	2.545414	-0.76886	0.632855
42	H	4.357876	-1.03669	-1.41902	42	H	4.468717	0.974945	-0.9862
43	H	7.584368	-0.87457	-0.49397	43	H	6.095804	-0.78065	-2.24108
44	H	6.651792	-0.71208	-1.99374	44	H	6.865544	-0.24628	-0.75396
45	H	7.54271	0.681445	-1.31979	45	H	6.79252	-1.99314	-1.12728
46	H	-5.23131	-0.51308	1.122594	46	H	-4.58194	-1.70331	1.114462
47	H	-4.84842	-2.23956	1.147438	47	H	-3.51427	-2.95287	1.768268
48	H	-3.74742	-1.08456	1.903632	48	H	-3.19573	-1.25212	2.120956

MPW1PW91/6-31G(d) Energy / Hartree =-965.05009588 a.u. MPW1PW91/6-31G(d) Energy / Hartree =-965.04892492 a.u.

Population = 10.413% Population = 8.891%

**8** Conf. 3		Standard Orientation (Ångstroms)			**8** Conf. 4		Standard Orientation (Ångstroms)		
I	atom	X	Y	Z	I	atom	X	Y	Z
1	C	0.801372	0.014711	0.953068	1	C	0.618288	1.583017	0.583608
2	C	0.472864	1.490082	0.590972	2	C	-0.46055	2.495181	-0.02846

3	C	-2.17192	1.695428	-1.54391	3	C	-2.75666	0.710658	-1.80769
4	C	-2.03322	1.922321	-0.05168	4	C	-2.78715	1.352642	-0.43483
5	C	-3.36679	0.794147	-1.91402	5	C	-3.27229	-0.74266	-1.80998
6	C	-3.11426	-0.60472	-1.4316	6	C	-2.3145	-1.62021	-1.05773
7	C	-3.48401	-1.14106	-0.11321	7	C	-2.36091	-1.90226	0.384987
8	C	-2.52136	-2.13695	0.495047	8	C	-1.02551	-2.04093	1.082587
9	C	-1.39546	-1.46349	1.297188	9	C	-0.46365	-0.69177	1.560874
10	C	-0.65532	-0.3507	0.553707	10	C	-0.40989	0.394214	0.482989
11	C	-1.02965	1.14769	0.764445	11	C	-1.53672	1.469023	0.399556
12	C	-2.77795	2.859062	0.545981	12	C	-3.93053	1.871471	0.026486
13	C	1.133421	-0.16384	2.437288	13	C	1.004321	1.976969	2.010849
14	C	1.880462	-0.6319	0.116739	14	C	1.864711	1.413715	-0.25665
15	C	3.222973	0.017122	0.144376	15	C	2.777825	0.287301	0.092491
16	C	4.270766	-0.50279	-0.50478	16	C	3.936771	0.099971	-0.54914
17	C	5.61035	0.151872	-0.47973	17	C	4.848283	-1.02906	-0.20566
18	C	6.702953	-0.53884	-1.24714	18	C	6.115175	-1.12875	-1.00858
19	O	5.796445	1.189874	0.133388	19	O	4.569587	-1.82102	0.679249
20	C	-4.37896	-0.41633	0.858892	20	C	-3.52601	-1.53251	1.267133
21	O	1.67527	-1.6297	-0.55614	21	O	2.121564	2.156742	-1.19057
22	H	-0.62524	-0.57918	-0.5156	22	H	-0.26709	-0.0633	-0.50186
23	H	-1.28474	1.286499	1.820912	23	H	-1.84277	1.705762	1.424747
24	O	-4.16472	-1.56633	-1.3136	24	O	-2.67996	-2.91943	-0.58864
25	H	-2.21726	-1.03877	-1.87947	25	H	-1.31729	-1.61974	-1.50397
26	H	0.716393	1.717633	-0.4521	26	H	-0.34914	2.553062	-1.1144
27	H	0.883918	2.273715	1.232992	27	H	-0.55483	3.507388	0.373573
28	H	-1.25367	1.26115	-1.95796	28	H	-1.7426	0.733299	-2.2254
29	H	-2.3056	2.660452	-2.04214	29	H	-3.38152	1.294369	-2.49045
30	H	-4.28744	1.2038	-1.48977	30	H	-4.27398	-0.78028	-1.37445
31	H	-3.48791	0.785024	-3.00387	31	H	-3.35345	-1.09609	-2.84479
32	H	-2.08351	-2.72666	-0.31817	32	H	-0.3199	-2.50174	0.382147
33	H	-3.06047	-2.83839	1.143593	33	H	-1.11407	-2.7195	1.939645
34	H	-1.7888	-1.07538	2.244194	34	H	-1.04501	-0.32534	2.414706
35	H	-0.67692	-2.24546	1.568241	35	H	0.543228	-0.8781	1.951675
36	H	-2.72608	3.034974	1.617026	36	H	-4.00009	2.315738	1.015762
37	H	-3.46763	3.48415	-0.01569	37	H	-4.8395	1.870561	-0.56983
38	H	1.166193	-1.2217	2.71337	38	H	1.633755	1.2247	2.495521
39	H	2.103834	0.278875	2.682007	39	H	1.554063	2.923337	2.005826
40	H	0.382401	0.325747	3.063361	40	H	0.112951	2.112875	2.629673
41	H	3.346968	0.939225	0.705347	41	H	2.495593	-0.39832	0.886002
42	H	4.15328	-1.42479	-1.0675	42	H	4.229677	0.78198	-1.34249
43	H	7.635247	0.019924	-1.16797	43	H	5.877922	-1.23254	-2.07307
44	H	6.845999	-1.55492	-0.86334	44	H	6.698284	-0.20718	-0.90509
45	H	6.417917	-0.63789	-2.30024	45	H	6.709163	-1.98068	-0.67837
46	H	-5.00949	0.320023	0.359804	46	H	-4.44773	-1.42055	0.695149
47	H	-5.03083	-1.14514	1.353129	47	H	-3.67818	-2.32628	2.006855
48	H	-3.80007	0.095362	1.630752	48	H	-3.34317	-0.59993	1.805464

MPW1PW91/6-31G(d) Energy / Hartree =-965.04745022 a.u. MPW1PW91/6-31G(d) Energy / Hartree =-965.04861355 a.u.

Population = 4.465% Population = 4.431%

**8** Conf. 5		Standard Orientation (Ångstroms)			**8** Conf. 6		Standard Orientation (Ångstroms)		
I	atom	X	Y	Z	I	atom	X	Y	Z
1	C	-0.79044	-1.2866	0.930065	1	C	0.883159	-0.30809	0.697825
2	C	0.202254	-2.43156	0.674903	2	C	0.623994	1.209618	0.880838
3	C	3.808377	-1.16197	-0.29975	3	C	-3.21971	2.060981	0.47311
4	C	2.398306	-1.67298	-0.55271	4	C	-1.75703	1.974165	0.06464
5	C	4.164318	0.097233	-1.11067	5	C	-4.18566	1.459155	-0.56277
6	C	3.022546	1.075007	-1.06558	6	C	-3.66407	0.12871	-1.02971
7	C	2.657704	1.861209	0.117691	7	C	-3.68254	-1.09332	-0.2187
8	C	1.19005	2.166011	0.312737	8	C	-2.53736	-2.05962	-0.41502
9	C	0.417294	1.071915	1.059342	9	C	-1.3002	-1.74015	0.432134
10	C	0.272856	-0.27538	0.350961	10	C	-0.54939	-0.44938	0.108889
11	C	1.349156	-1.41342	0.507453	11	C	-0.89252	0.943025	0.756771
12	C	2.127735	-2.33673	-1.68139	12	C	-1.28294	2.805118	-0.86929
13	C	-1.11937	-1.1053	2.414173	13	C	1.110771	-1.01449	2.038584
14	C	-2.07149	-1.36935	0.128123	14	C	2.004342	-0.66727	-0.24911
15	C	-2.92767	-0.14565	0.078282	15	C	3.353631	-0.11078	0.07122
16	C	-4.11065	-0.15546	-0.54925	16	C	4.424092	-0.42148	-0.67066
17	C	-5.03512	1.000328	-0.65639	17	C	5.803176	0.074629	-0.43901
18	C	-4.65282	2.317098	-0.03298	18	C	6.073312	1.004236	0.714928
19	O	-6.09083	0.853622	-1.25263	19	O	6.691701	-0.28684	-1.19498
20	C	3.515299	1.901822	1.357912	20	C	-4.4036	-1.17754	1.103415
21	O	-2.40379	-2.38268	-0.46476	21	O	1.831085	-1.37336	-1.22899
22	H	0.072389	-0.1199	-0.7168	22	H	-0.47731	-0.32587	-0.97769
23	H	1.852509	-1.27208	1.470493	23	H	-1.30233	0.762282	1.75713
24	O	3.262274	2.484588	-1.03524	24	O	-4.55835	-0.93876	-1.35612
25	H	2.195256	0.804166	-1.72241	25	H	-2.83548	0.212103	-1.73391
26	H	-0.04126	-2.9626	-0.24592	26	H	1.013387	1.794363	0.044012
27	H	0.319602	-3.164	1.478033	27	H	0.969506	1.661451	1.81475
28	H	4.531964	-1.94878	-0.53673	28	H	-3.4916	3.108093	0.642307
29	H	3.926753	-0.93918	0.7655	29	H	-3.3619	1.542639	1.426734
30	H	5.079203	0.550409	-0.71866	30	H	-5.18406	1.354404	-0.12903
31	H	4.363643	-0.17489	-2.1531	31	H	-4.2788	2.132721	-1.42186
32	H	0.738143	2.326149	-0.67279	32	H	-2.26371	-2.05633	-1.47627
33	H	1.085773	3.107857	0.865947	33	H	-2.87413	-3.07674	-0.17771
34	H	0.853158	0.918456	2.053234	34	H	-1.55932	-1.76425	1.497258
35	H	-0.58703	1.471435	1.244306	35	H	-0.59196	-2.56381	0.280661
36	H	1.138909	-2.71366	-1.92187	36	H	-0.25389	2.78548	-1.2128
37	H	2.901577	-2.52301	-2.42157	37	H	-1.92611	3.545764	-1.33745
38	H	-1.68464	-0.19124	2.617569	38	H	1.108156	-2.10262	1.930327
39	H	-1.71174	-1.95422	2.769657	39	H	2.070355	-0.7213	2.475733
40	H	-0.20199	-1.0696	3.008422	40	H	0.329822	-0.74126	2.753948
41	H	-2.56199	0.750942	0.568002	41	H	3.43247	0.555815	0.924649
42	H	-4.45442	-1.06835	-1.02804	42	H	4.309653	-1.0906	-1.5194

43	H	-4.50345	2.207195	1.045598	43	H	7.132389	1.260835	0.72832
44	H	-3.71475	2.68931	-0.45632	44	H	5.479825	1.919219	0.623897
45	H	-5.4445	3.043508	-0.21523	45	H	5.802401	0.532905	1.664777
46	H	4.555924	1.659732	1.13668	46	H	-5.19231	-0.42794	1.184145
47	H	3.486163	2.907141	1.791421	47	H	-4.86422	-2.16584	1.207012
48	H	3.153559	1.203633	2.118065	48	H	-3.71517	-1.04494	1.942677

MPW1PW91/6-31G(d) Energy / Hartree =-965.04595914 a.u. MPW1PW91/6-31G(d) Energy / Hartree =-965.04840612 a.u.

Population = 3.092% Population = 1.488%

**8** Conf. 7		Standard Orientation (Ångstroms)			**8** Conf. 8		Standard Orientation (Ångstroms)		
I	atom	X	Y	Z	I	atom	X	Y	Z
1	C	0.983827	-0.53309	0.43793	1	C	0.642983	1.576257	0.602653
2	C	0.868757	1.005629	0.584406	2	C	-0.44947	2.502959	0.038295
3	C	-2.90735	2.188484	0.515948	3	C	-2.77156	0.768833	-1.75722
4	C	-1.50346	1.960678	-0.0239	4	C	-2.7793	1.365718	-0.36378
5	C	-4.01569	1.652985	-0.40781	5	C	-3.28352	-0.68513	-1.7978
6	C	-3.65719	0.271947	-0.88119	6	C	-2.30907	-1.583	-1.09261
7	C	-3.70534	-0.92612	-0.03677	7	C	-2.32818	-1.91272	0.340681
8	C	-2.6704	-1.99368	-0.30826	8	C	-0.97998	-2.06896	1.009307
9	C	-1.33821	-1.76935	0.416761	9	C	-0.4153	-0.73392	1.522624
10	C	-0.50953	-0.55871	-0.01058	10	C	-0.38272	0.386999	0.48006
11	C	-0.66808	0.874538	0.617627	11	C	-1.51448	1.459444	0.451597
12	C	-1.05061	2.724942	-1.02311	12	C	-3.91587	1.86458	0.134828
13	C	1.254361	-1.22636	1.779006	13	C	1.049727	1.925966	2.035701
14	C	1.938794	-1.07591	-0.60749	14	C	1.875326	1.440486	-0.2642
15	C	3.379059	-0.70066	-0.62132	15	C	2.8036	0.308674	0.035561
16	C	3.989401	0.169993	0.19444	16	C	3.941067	0.150013	-0.65311
17	C	5.43325	0.517162	0.126114	17	C	4.930065	-0.93602	-0.44275
18	C	6.305082	-0.12738	-0.91903	18	C	4.682724	-1.96961	0.624629
19	O	5.878132	1.323542	0.927687	19	O	5.928199	-0.96403	-1.14594
20	C	-4.3069	-0.92098	1.346402	20	C	-3.47837	-1.57637	1.255015
21	O	1.551176	-1.85454	-1.4669	21	O	2.110588	2.211025	-1.18051
22	H	-0.52977	-0.4668	-1.10237	22	H	-0.25494	-0.03721	-0.52159
23	H	-0.99106	0.759335	1.658792	23	H	-1.80263	1.6613	1.489358
24	O	-4.66618	-0.71884	-1.09459	24	O	-2.66123	-2.89846	-0.65996
25	H	-2.89256	0.26599	-1.65881	25	H	-1.3203	-1.56344	-1.55691
26	H	1.224866	1.526572	-0.30685	26	H	-0.35692	2.5953	-1.04698
27	H	1.334751	1.458791	1.464095	27	H	-0.54018	3.501625	0.473543
28	H	-3.06923	3.259542	0.676014	28	H	-1.76505	0.807882	-2.19162
29	H	-3.00341	1.708326	1.495101	29	H	-3.40966	1.372742	-2.40948
30	H	-4.97404	1.647004	0.119068	30	H	-4.27709	-0.74062	-1.34575
31	H	-4.12964	2.313793	-1.27421	31	H	-3.38245	-1.00472	-2.84202
32	H	-2.49565	-2.03466	-1.38943	32	H	-0.28527	-2.50295	0.281298
33	H	-3.07094	-2.97223	-0.01448	33	H	-1.05063	-2.7762	1.844596
34	H	-1.50255	-1.74764	1.500461	34	H	-0.98494	-0.3982	2.396711
35	H	-0.72106	-2.65524	0.226296	35	H	0.597781	-0.92926	1.892711

36	H	-0.0647	2.606255	-1.46021	36	H	-3.96923	2.275898	1.13924
37	H	-1.6692	3.509212	-1.45173	37	H	-4.83532	1.879862	-0.445
38	H	1.133999	-2.31072	1.704139	38	H	1.686325	1.160179	2.488773
39	H	2.271112	-1.02555	2.126547	39	H	1.598303	2.872817	2.052437
40	H	0.564025	-0.86054	2.54431	40	H	0.166925	2.04123	2.670743
41	H	3.935818	-1.20946	-1.4038	41	H	2.525793	-0.38035	0.826618
42	H	3.45238	0.694451	0.977974	42	H	4.19233	0.857965	-1.4383
43	H	6.310736	-1.21549	-0.80286	43	H	4.611289	-1.49916	1.610235
44	H	5.933854	0.091497	-1.92487	44	H	3.740502	-2.49662	0.445404
45	H	7.321791	0.251624	-0.81856	45	H	5.503193	-2.68685	0.626019
46	H	-5.02101	-0.10616	1.475152	46	H	-4.40955	-1.44556	0.702771
47	H	-4.83585	-1.86449	1.518475	47	H	-3.61738	-2.39605	1.968609
48	H	-3.53637	-0.82951	2.117028	48	H	-3.2875	-0.66316	1.822956

MPW1PW91/6-31G(d) Energy / Hartree =-965.04961758 a.u. MPW1PW91/6-31G(d) Energy / Hartree =-965.04752578 a.u.

Population = 1.407% Population = 1.154%

**8** Conf. 9		Standard Orientation (Ångstroms)			**8** Conf. 10		Standard Orientation (Ångstroms)		
I	atom	X	Y	Z	I	atom	X	Y	Z
1	C	0.823312	-0.025	0.935277	1	C	-0.75103	1.677969	0.668167
2	C	0.517422	1.457132	0.580158	2	C	-0.41009	0.942992	1.990977
3	C	-2.14398	1.715199	-1.52753	3	C	3.051709	-0.98163	1.705446
4	C	-1.98795	1.934261	-0.03588	4	C	1.54279	-0.81559	1.806109
5	C	-3.35606	0.834252	-1.89054	5	C	3.506226	-1.84161	0.513264
6	C	-3.12148	-0.57028	-1.41563	6	C	2.789193	-1.40061	-0.73211
7	C	-3.48978	-1.10628	-0.09666	7	C	3.076342	-0.15054	-1.44386
8	C	-2.53803	-2.11873	0.501416	8	C	1.907174	0.542979	-2.10457
9	C	-1.39747	-1.464	1.298231	9	C	1.121006	1.463347	-1.16297
10	C	-0.64398	-0.36315	0.550353	10	C	0.354331	0.794126	-0.02325
11	C	-0.98917	1.140935	0.768089	11	C	0.951733	0.517129	1.406055
12	C	-2.71245	2.880065	0.572303	12	C	0.799218	-1.82659	2.267826
13	C	1.168025	-0.21461	2.415163	13	C	-0.40581	3.159505	0.746701
14	C	1.879707	-0.69014	0.08452	14	C	-2.14726	1.494232	0.112385
15	C	3.236546	-0.06463	0.093324	15	C	-2.71414	0.113828	0.052788
16	C	4.251221	-0.60586	-0.59255	16	C	-3.94087	-0.11116	-0.43162
17	C	5.631012	-0.06402	-0.65731	17	C	-4.51746	-1.4845	-0.4928
18	C	5.967131	1.199765	0.089813	18	C	-5.89744	-1.5995	-1.07797
19	O	6.465367	-0.66073	-1.32019	19	O	-3.89453	-2.4525	-0.08872
20	C	-4.36657	-0.37188	0.884828	20	C	4.258972	0.722371	-1.10525
21	O	1.648242	-1.68271	-0.58646	21	O	-2.80453	2.440066	-0.2956
22	H	-0.62828	-0.58985	-0.51958	22	H	-0.1064	-0.13235	-0.38609
23	H	-1.2309	1.281711	1.82744	23	H	1.691304	1.297447	1.619265
24	O	-4.18563	-1.51632	-1.29362	24	O	3.435166	-1.42999	-2.0076
25	H	-2.23473	-1.01627	-1.87209	25	H	1.75466	-1.74273	-0.7796
26	H	0.754523	1.683426	-0.46465	26	H	-1.06871	0.093145	2.176966
27	H	0.948434	2.231596	1.220302	27	H	-0.3927	1.566431	2.888968
28	H	-1.23643	1.268363	-1.95171	28	H	3.4306	-1.43315	2.628159

29	H	-2.26708	2.684142	-2.02086	29	H	3.520273	0.004533	1.624757
30	H	-4.26653	1.256604	-1.45693	30	H	4.589617	-1.76269	0.386482
31	H	-3.48642	0.831427	-2.97934	31	H	3.282076	-2.8964	0.707227
32	H	-2.11407	-2.71182	-0.31665	32	H	1.237706	-0.22327	-2.51195
33	H	-3.08311	-2.81435	1.15121	33	H	2.266442	1.134927	-2.95573
34	H	-1.78023	-1.06946	2.246932	34	H	1.77922	2.24136	-0.75976
35	H	-0.69039	-2.25743	1.565978	35	H	0.383271	1.99356	-1.77834
36	H	-2.64789	3.050953	1.643474	36	H	-0.28003	-1.77533	2.366203
37	H	-3.39773	3.517842	0.019604	37	H	1.257998	-2.76451	2.569855
38	H	1.184229	-1.27375	2.687506	38	H	-0.45071	3.641694	-0.23225
39	H	2.148857	0.209261	2.651693	39	H	-1.11793	3.671663	1.399714
40	H	0.432827	0.286685	3.050624	40	H	0.59575	3.307979	1.158792
41	H	3.365723	0.846123	0.670405	41	H	-2.13213	-0.73093	0.407147
42	H	4.085546	-1.51792	-1.15985	42	H	-4.53876	0.720743	-0.79312
43	H	5.342555	2.030611	-0.25266	43	H	-5.90412	-1.20869	-2.10138
44	H	5.786251	1.076149	1.162028	44	H	-6.22637	-2.63847	-1.07753
45	H	7.016266	1.445119	-0.07378	45	H	-6.60094	-0.98718	-0.50331
46	H	-4.98947	0.376031	0.393443	46	H	5.047892	0.159559	-0.60407
47	H	-5.02601	-1.0928	1.380586	47	H	4.676856	1.144174	-2.02553
48	H	-3.77451	0.127466	1.654806	48	H	3.968738	1.557329	-0.46146

2.3 Specific rotation calculation for compounds 9 and 10

The specific optical rotation calculations for compounds were carried out by using Information Gaussian 09. The conformers were further optimized at the B3LYP/6-311G(d,p) level with the IEFPCM solvent model for chloroform, all of which were subjected to specific optical rotation calculations at the B3LYP/6-311+G(d) level in chloroform with SMD model. The calculated specific optical rotation data of these conformers were averaged according to the Boltzmann distribution theory and their relative Gibbs free energy. The predicted ORD curves were generated from specific rotations calculated at four different wavelengths.

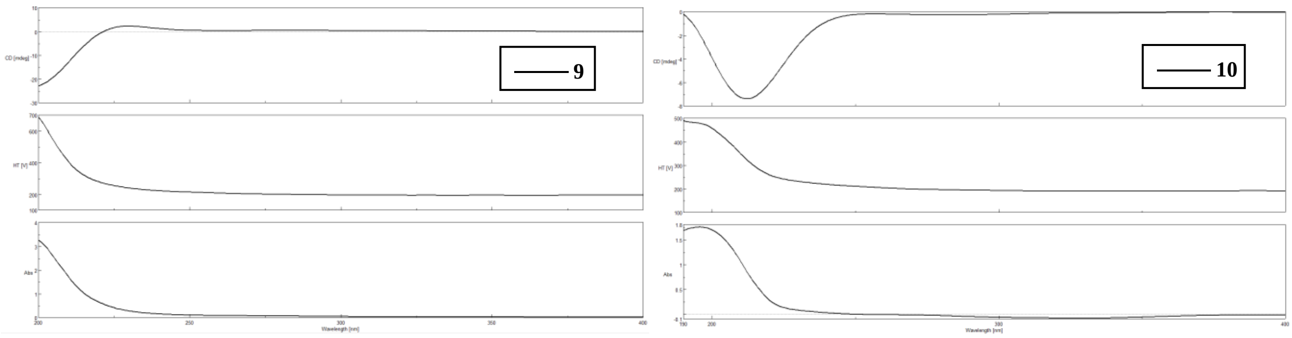

Figure S104. Experimental CD spectrum of **9** and **10** observed at the region of 200–400 nm and 190–400 nm in CH_3CN, respectively. No obvious cotton effects were detected. The negative cotton effects at 200 nm were not informative for ECD calculation.

n	Average	Std.Dev.	Maximum	Minimum
6	-40.500	0.5000	-40.000	-41.000

S.No	Sample ID	Time	Result	Scale	OR ° Arc	WLG	Lg.mm	Conc.	Temp.	Comment
1	EEG-6	04:51:56 PM	-40.000	SR	-0.040	589	100.00	0.100	19.9	
2	EEG-6	04:52:06 PM	-40.000	SR	-0.040	589	100.00	0.100	19.9	
3	EEG-6	04:52:16 PM	-41.000	SR	-0.041	589	100.00	0.100	19.9	
4	EEG-6	04:52:26 PM	-41.000	SR	-0.041	589	100.00	0.100	19.9	
5	EEG-6	04:52:36 PM	-41.000	SR	-0.041	589	100.00	0.100	19.9	
6	EEG-6	04:52:45 PM	-40.000	SR	-0.040	589	100.00	0.100	19.9	

n	Average	Std.Dev.	Maximum	Minimum
6	-97.334	0.3849	-96.667	-97.667

S.No	Sample ID	Time	Result	Scale	OR ° Arc	WLG	Lg.mm	Conc.	Temp.	Comment
1	LBAAD-11	11:39:59 AM	-97.667	SR	-0.293	589	100.00	0.300	18.9	
2	LBAAD-11	11:40:05 AM	-97.667	SR	-0.293	589	100.00	0.300	18.9	
3	LBAAD-11	11:40:12 AM	-97.667	SR	-0.293	589	100.00	0.300	18.9	
4	LBAAD-11	11:40:18 AM	-97.333	SR	-0.292	589	100.00	0.300	18.9	
5	LBAAD-11	11:40:24 AM	-97.000	SR	-0.291	589	100.00	0.300	18.9	
6	LBAAD-11	11:40:34 AM	-96.667	SR	-0.290	589	100.00	0.300	18.9	

Figure S105. Specific optical rotation value of **9** and **10**.

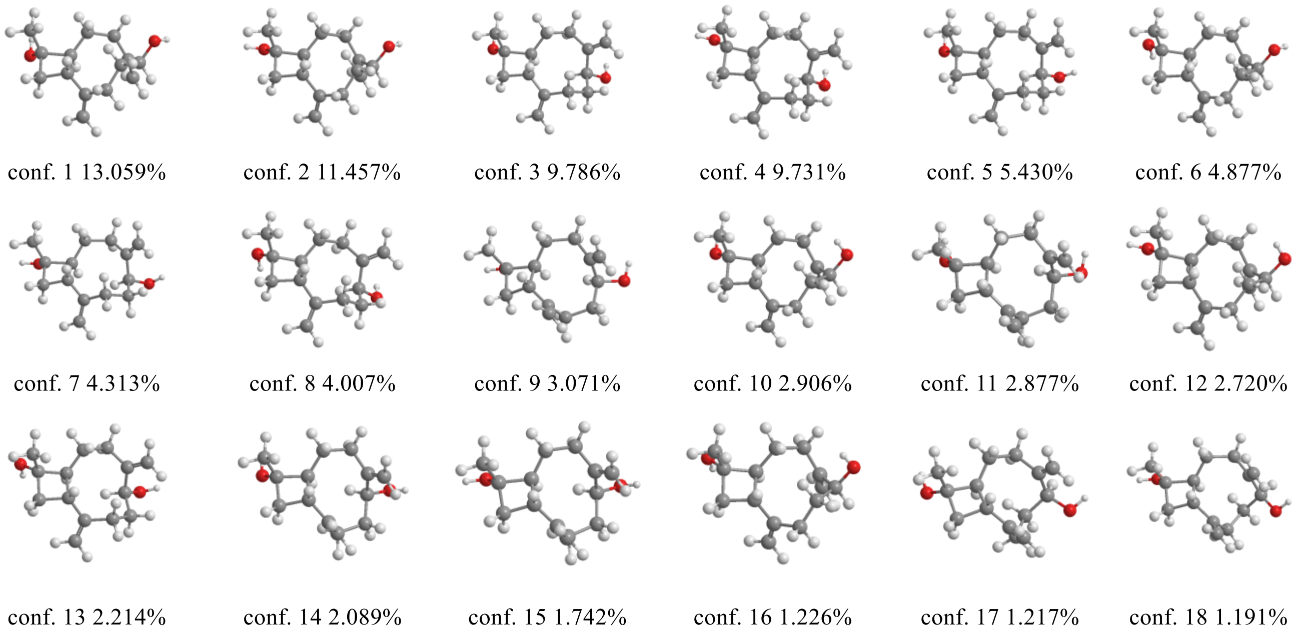

Figure S106. Re-optimized conformers above 1% population (OPLS_2005) of (1*S*,5*S*,9*R*,11*S*)-**9** calculated at the B3LYP/6-311+G* level with SMD solvent model for methanol.

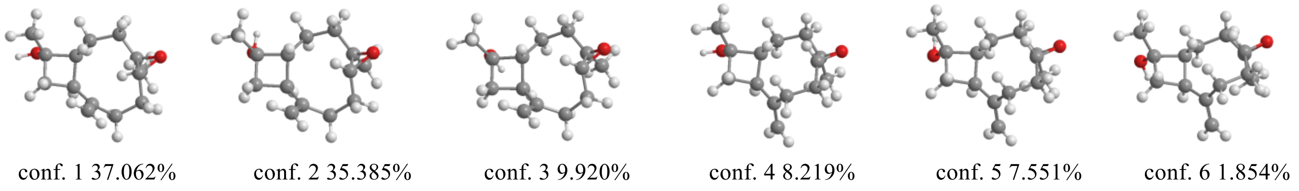

Figure S107. Re-optimized conformers above 1% population (OPLS_2005) of (1*R*,4*R*,5*R*,9*S*,11*S*)-**9** calculated at the B3LYP/6-311+G* level with SMD solvent model for methanol.

Table S13. Specific rotation calculation for compounds **9** and **10**.

Compounds	Conformers	$[\alpha]_D$ calc.	Boltzmann distribution	$[\alpha]_D$
**9**	**C1**	+7.01	0.13059	+0.915436
	**C2**	+41.81	0.11457	+4.790172
	**C3**	−100.15	0.09786	−9.80068
	**C4**	−63.21	0.09731	−6.15097
	**C5**	−92.43	0.05430	−5.01895
	**C6**	+46.41	0.04877	+2.263416
	**C7**	−52.67	0.04313	−2.27166
	**C8**	−65.36	0.04007	−2.61898
	**C9**	−216.17	0.03071	−6.63858
	**C10**	+25.94	0.02906	+0.753816
	**C11**	−254.87	0.02877	−7.33261
	**C12**	+60.66	0.02720	+1.649952
	**C13**	−56.69	0.02214	−1.25512
	**C14**	−222.77	0.02089	−4.65367
	**C15**	−184.55	0.01742	−3.21486
	**C16**	+65.64	0.01226	+0.804746
	**C17**	−208.14	0.01217	−2.53306
	**C18**	−171.21	0.01191	−2.03911
				−50.5
**10**	**C1**	−214.88	0.37062	−79.6388
	**C2**	−242.71	0.35385	−85.8829
	**C3**	−208.75	0.09920	−20.708
	**C4**	+79.67	0.08219	+6.548077
	**C5**	+44.43	0.07551	+3.354909
	**C6**	+70.24	0.01854	+1.30225
				−175.0
